# Zegocractin for acute pancreatitis with systemic inflammatory response syndrome: a randomized, controlled, dose-ranging, phase 2b trial

**DOI:** 10.1016/j.eclinm.2026.103757

**Published:** 2026-02-23

**Authors:** Robert Sutton, Pramod K. Garg, Joseph Miller, S. Suresh Kumar, James L. Buxbaum, Mathew Philip, Jeffrey Zhang, Kenneth Stauderman, Sudarshan Hebbar, Bechien U. Wu, W. Frank Peacock, Timothy B. Gardner

**Affiliations:** a University of Liverpool and Liverpool University Hospitals NHS Foundation Trust, Liverpool, UK; b Department of Gastroenterology, All India Institute of Medical Sciences, New Delhi, 110029, India; c Department of Emergency Medicine, Henry Ford Hospital, Detroit, USA; d Department of Surgery, Jawaharlal Institute of Postgraduate Medicine and Research, Puducherry, 605006, India; e Keck School of Medicine, University of Southern California, Los Angeles, USA; f Department of Gastroenterology, Lisie Hospital, Kochi, 682018, India; g Princeton Pharmatech, Princeton, USA; h CalciMedica, La Jolla, USA; i Southern California Permanente Medical Group, Los Angeles, USA; j Henry J.N. Taub Department of Emergency Medicine, Baylor College of Medicine, Houston, USA; k Section of Gastroenterology and Hepatology, Dartmouth Hitchcock Medical Center, Lebanon, USA

**Keywords:** Acute pancreatitis, Organ failure, Calcium release-activated calcium channel inhibition, Zegocractin, Randomised clinical trial

## Abstract

**Background:**

Acute pancreatitis (AP) is without specific drug therapy. We conducted a phase 2b trial of the calcium release-activated calcium channel inhibitor zegocractin, previously found to accelerate recovery of food intake in AP, to determine dose–response, target population, endpoints, safety and tolerability in AP with systemic inflammatory response syndrome.

**Methods:**

This double-blind, randomised, placebo-controlled, phase 2b trial enrolled adults (aged ≥18 years) with AP and systemic inflammatory response syndrome at 37 centres in the US and India. Patients were randomly assigned (1:1:1:1) to receive placebo or 0.5 (low), 1.0 (medium) or 2.0 (high) mg per kilogramme intravenous zegocractin once daily for 3 days. The primary outcome was time to solid food tolerance, and all outcomes were specified a priori. This trial is registered with ClinicalTrials.gov (NCT04681066) and is complete.

**Findings:**

Between 30th March, 2021 and 16th April, 2024, 216 patients were assigned to placebo (N = 53), low (N = 53), medium (N = 56) or high (N = 54) dose zegocractin. The primary outcome of median time to solid food tolerance was 66, 78, 64, and 67 h in these groups respectively (n.s.). Dose–response was observed in patients with a high haematocrit (n = 92), the median times being 113.5, 78, 64 and 67 h, and in those with Balthazar D or E computed tomography at presentation (n = 145), at 112, 68.5, 68.5, and 66 h respectively. Overall, there were dose-dependent responses for the secondary outcomes new-onset severe respiratory failure (4, 4, 0, and 0 patients), new-onset necrotising pancreatitis (17, 17, 20 and 11 patients) and time to medically indicated discharge (104, 109.5, 104.5 and 89 median hours), reflected in an exploratory win ratio for high dose zegocractin compared to placebo of 1.640 (95% CI 1.030–2.612; p = 0.04).

**Interpretation:**

This trial was negative for the primary endpoint of time to solid food tolerance in the whole trial population but improvement with zegocractin was seen in patients with a high haematocrit or Balthazar score. Multiple secondary endpoints improved consistently with zegocractin compared to placebo, most notably in preventing new-onset severe respiratory failure. These findings identified a suitable dose, a potential patient population, and endpoints for a phase 3 trial.

**Funding:**

CalciMedica.

**Research in context**

**Evidence before this study**

Acute pancreatitis (AP) has no licenced specific, targeted drug to reduce its impact and complications, notably organ failure and pancreatic necrosis, despite six decades of randomised clinical trials testing many therapies. The most recent systematic and technical reviews, updates and guidelines identify that among reasons for this failure are insufficient understanding of pathophysiology, inappropriate drug choice, uncertain patient selection and unsuitable endpoints. We proposed and obtained preclinical evidence for a novel mechanism of cellular calcium release-activated calcium (CRAC) entry (also known as store-operated calcium entry, SOCE) and overload as critical to the onset and progression of AP, then developed the drug candidate zegocractin to block ORAI channels, the principal CRAC channel in pancreatic parenchymal cells, leukocytes and endothelia. Our phase 2a randomised trial of low- or high-dose zegocractin versus standard of care in patients with AP and accompanying systemic inflammatory response syndrome (SIRS) found a favourable safety profile and earlier resumption of solid food consumption, improvements of pancreatic injury on computed tomography (CT), and earlier hospital discharge with zegocractin, justifying a larger, double-blind, placebo-controlled, dose-ranging phase 2b trial.

**Added value of this study**

The CARPO trial was a phase 2b, multinational, double-blind, randomised, placebo-controlled trial of low, medium or high dose zegocractin in patients with AP and SIRS (randomly assigned 1:1:1:1) designed to determine dose–response, efficacy, safety, and guide selection of patients and endpoints for phase 3 trials. The primary endpoint of time to solid food tolerance was not altered across the whole trial population but demonstrated dose–response in patients at higher risk of organ failure (high haematocrit and/or higher levels of pancreatic injury on screening CT). Dose–response was also seen for the development of severe respiratory failure, with consistent reductions in further secondary outcomes contributing to a statistically significant exploratory hierarchical composite win ratio for high dose zegocractin compared to placebo. The trial added to data indicating acceptable safety and tolerability for zegocractin.

**Implications of all the available evidence**

Although zegocractin did not alter the primary outcome across the whole trial population, the CARPO trial of zegocractin in patients with AP and SIRS met its objectives, identifying a dose–response, with efficacy greatest at the high dose, and an acceptable safety and tolerability profile. The trial has identified patients with AP at higher risk of organ failure as likely to benefit most from zegocractin and developed a win ratio as a suitable endpoint to evaluate effectiveness. These data support targeting CRAC channels as a novel therapeutic approach, which may have applications in other conditions of severe inflammatory stress.

## Introduction

Acute pancreatitis (AP) is a common gastrointestinal condition of increasing global incidence without approved pharmaceutical treatment, management largely being supportive.^1^ Randomised trials of drugs for AP have previously been repeatedly disappointing,^2^ frustrating drug development for a major unmet need, and suggesting the right mechanisms have yet to be identified effectively and targeted. In patients with AP, injury to pancreatic exocrine cells releases prematurely activated zymogens, cytokines and inflammatory mediators, inducing the systemic inflammatory response syndrome (SIRS) in the majority.^1^^,^^3^^,^^4^ When SIRS persists, the risk of organ failure increases, with the lungs being most commonly affected, increasing the risk of death.^1^^,^^4^^,^^5^ Persistent SIRS also increases the risk of necrotising pancreatitis, increasing morbidity and further increasing the risk of organ failure.^4^ There is, therefore, an obvious need for a safe, rapidly acting therapeutic to reduce the inflammatory response in AP, prevent organ failure and protect the pancreas,^1^^,^^3^, ^4^, ^5^ thereby reducing morbidity and mortality.

Calcium ion overload in pancreatic acinar and ductal cells triggers injury and inflammation in AP regardless of aetiology, including gallstones, alcohol and hypertriglyceridaemia.^3^^,^^6^, ^7^, ^8^ This calcium overload follows abnormal sustained release from the endoplasmic reticulum calcium store which, when depleted, activates the plasma membrane calcium release-activated calcium (CRAC) channel ORAI1, replenishing the store.^8^ As ORAI1-mediated store-operated calcium entry is also integral to immune cell function,^9^ inhibition of ORAI1 has the potential both to reduce pancreatic injury and decrease the inflammatory response. Zegocractin is a potent and selective ORAI1 CRAC channel inhibitor that reduces pancreatic and lung injury in preclinical AP,^10^, ^11^, ^12^ and was well tolerated in a previous phase 2a open label trial in patients with AP and SIRS that showed promising signs of efficacy, warranting further evaluation.^13^ The development of zegocractin for AP depends on determination of an appropriate dose, appropriate patients to target and, with no prior successful drug developed and approved for AP, appropriate outcome measures. Here, we report the results of a phase 2b trial, designed to determine the dose–response relationship and efficacy of zegocractin in patients with AP and SIRS (the CARPO trial), identify patients to target, and establish endpoints for future phase 3 trials. We also assessed safety and tolerability.

## Methods

### Study design and participants

CARPO was a phase 2b, double-blind, randomised, placebo-controlled, parallel-group, phase 2 trial conducted at 37 centres in the US and India. The trial was done in compliance with the International Council on Harmonisation Good Clinical Practice guidelines and in accordance with the principles set forth in the declaration of Helsinki. The trial was registered on December 15th, 2020, in ClinicalTrials.gov (NCT04681066), and the protocol (see Appendix p 1 for version 2.0 prespecified before the start and version 4.0 finalised during recruitment before any data were available for analysis, other than safety data reviewed confidentially by the Independent Data Monitoring Committee; the summary of changes and rationale are also provided) was approved by central and local institutional review boards and ethics committees for all participating centres. All participating centres worked to provide care consistent with the 2018 American Gastroenterological Association Institute Technical Review of the Initial Medical Management of Acute Pancreatitis.^14^

Adult patients with AP and SIRS were enrolled in the trial. The diagnosis of AP required characteristic abdominal pain, with a serum lipase >3 times the upper limit of normal or characteristic findings on contrast-enhanced computed tomography (CECT) or magnetic resonance imaging (MRI).^1^ SIRS was established by the presence of two or more of the following criteria: temperature <36 °C or >38 °C, heart rate >90 beats per minute, respiratory rate >20 breaths/minute or arterial carbon dioxide tension <32 mmHg, white blood cell count >12,000 mm^3^, or <4000 mm^3^.^15^ In addition to SIRS, patients were also required to have one of the following: a high haematocrit (≥44% for men or ≥ 40% for women), a peripancreatic fluid collection (Balthazar D or E score) or pleural effusion on imaging, or guarding or rebound tenderness on abdominal examination. A full list of exclusion criteria is given in the protocol. All patients, or their legally authorised representatives, gave written informed consent.

### Randomisation and masking

Patients were assigned in a 1:1:1:1 ratio to placebo, or low (0.5 mg/kg; 0.3125 mL/kg), medium (1.0 mg/kg; 0.625 mL/kg) or high dose (2.0 mg/kg; 1.25 mL/kg) zegocractin (as Auxora™ from CalciMedica), infused intravenously over 4 h once daily for 3 days. Placebo infusions each comprised 1 of 3 random volumes corresponding to each zegocractin dose. Trial products containing placebo or zegocractin were visually identical and packaged in a manner that maintained masking, including all notation and approved labelling. Randomisation was performed centrally for each country using a computer-generated randomisation scheme with a block size of 8 that was accessed through an interactive voice/web response system (IXRS). Randomisation was stratified first by sex, then by the presence of an elevated haematocrit (≥44% for men or ≥ 40% for women) and hypoxaemia (PaO_2_/FiO_2_ ≤ 360 mmHg). CARPO was a double-blind trial with both participants and study teams (sponsor, investigators, study coordinators, and pharmacists) masked to treatment.

### Procedures

Trial treatment was begun within 8 h of informed consent. After the first trial infusion, patients were offered a low fat, ≥500 calorie solid meal at each mealtime until discharge, if not nil per os for a medical reason, recording whether they ate ≥50% of the meal, vomited, or experienced increased abdominal pain in the 2 h following the meal. If solid food was not tolerated, liquid food was offered. Throughout the trial, including daily after discharge, patients completed a modified American Neurogastroenterology and Motility Society Gastroparesis Cardinal Symptom Index Daily Diary (mGCSI-DD)^16^ worksheet (Table S1 in the Appendix p 9), and daily while hospitalised, a pain 0–10 numeric rating scale.^17^ CECT or MRI was performed before randomisation, from which the Balthazar CT severity score (A to E)^18^ was determined, on day 30, and as required for standard care. All imaging was interpreted centrally by two independent, experienced radiologists blinded to treatment assignments, with differences resolved by consensus. The peripheral oxygen saturation (SpO_2_) and fraction of inspired oxygen (FiO_2_) were assessed daily for the first 10 days, and all organ support assessed daily during hospitalisation.

### Outcomes

All endpoints were defined a priori in the protocol. The primary endpoint and secondary endpoint to determine dose–response was time to solid food tolerance,^19^ and the development of (new-onset) severe respiratory failure, respectively. Time to solid food tolerance was recorded from the start of trial treatment until the patient could eat ≥50% of a low fat, ≥500-calorie solid meal, without increasing abdominal pain or vomiting within 2 h of that and all subsequent meals. For patients discharged not tolerating solid food, this time was determined using the mGCSI-DD worksheet as 8 am on the first of 3 consecutive days when there was no vomiting, no or mild nausea or abdominal pain, and patients finished a normal-sized meal.

Severe respiratory failure was defined as the use of invasive mechanical ventilation, or ≥48 h of either high flow nasal cannula or non-invasive mechanical ventilation, excluding for a procedure or obstructive sleep apnoea. Severe cardiovascular failure was defined as receiving vasopressors or inotropes for ≥48 h, and severe renal failure as the initiation of renal replacement therapy. Organ failure was defined as the development of severe respiratory failure and/or severe renal failure and/or severe cardiac failure.

The development of necrotising pancreatitis was determined from the day 30 CECT, or if unavailable, the last CECT following trial treatment. Time to medically indicated discharge was taken from the start of trial treatment to when the patient tolerated solid food, abdominal pain was controlled or resolved (≥50% pain scale reduction from peak level in the first 24 h and no opioids), and the patient was without infection requiring continued hospitalisation. For patients discharged before meeting these criteria, this time was determined using the mGCSI-DD worksheet as for solid food tolerance.

The Independent Data Monitoring Committee evaluated the progress of the trial and examined safety end points sequentially after 20, 60, 120, and 140 patients were enrolled and had completed the trial. Safety was evaluated by treatment-emergent adverse events coded per the Medical Dictionary for Regulatory Activities (MedDRA) version 26.0.

### Statistical analysis

The statistical analysis was conducted adhering to the statistical analysis plan version 1.0 (see Appendix p 1), which although not prespecified before the trial began, was written, reviewed by the US Food and Drug Administration (FDA) and Steering Committee members, then finalised a priori (10th June 2024), before any unblinded data were available (database lock 18th June 2024; unblinded data and analyses available 22nd June 2024).

Sample size was based on the PYTHON trial, which found the median time to full oral diet in the on demand feeding group was 6 days,^20^ and a phase 2a trial of zegocractin, which found 7 of 14 (50%) patients receiving zegocractin tolerated solid food at 72 h versus 1 of 7 (14%) receiving standard care.^13^ We calculated a sample size of 216 patients randomised in a 1:1:1:1 ratio to 4 groups of 54 patients each would provide 86% power for testing the difference in two populations having a median length of time of 72 and 144 h, respectively, to tolerating solid food. In addition, it would provide 80% power with a two-sided alpha of 0.05 to detect a 45% response rate for tolerating solid food in a zegocractin group versus 20% in the placebo group at 72 h.

All statistics were compiled from the modified intention-to-treat (mITT) population, comprising all randomised patients receiving any trial treatment. The generalised multiple comparisons and modeling (gMCP-Mod) analysis^21^^,^^22^ as described in the statistical analysis plan was the primary statistical method used for determining a dose–response relationship for both time to solid food tolerance and severe respiratory failure, as approved by the FDA and European Medicines Agency (EMA) (see Appendix), with alpha at 0.15 that allows for false positive results at a higher rate appropriate to a phase 2 dose-ranging trial. The time to solid food analysis was performed in the whole mITT population and in patients at higher risk of organ failure, identified by a high haematocrit or high Balthazar CT severity score (D or E). The analysis of severe respiratory failure was only performed in the whole mITT population.

The results of the additional secondary analyses were not adjusted for multiplicity and therefore should not be interpreted to inform definitive treatment effects but characterise a potential composite phase 3 endpoint. An exploratory win-ratio with the potential to serve as a phase 3 endpoint was calculated for each zegocractin group versus placebo using a hierarchical composite endpoint of mortality, new-onset severe respiratory failure, new-onset necrotising pancreatitis, and time to medically indicated discharge, in that order.^23^^,^^24^ Patients were included in the statistical analyses if they received any amount of study drug. Missing data in the response variable after early termination prior to Day 30 were not imputed in the win ratio. Statistical analyses were conducted using SAS software, version 9.4 (for further detail on statistical considerations see the Appendix p 5).

### Role of the funding source

The trial was designed by the sponsor (CalciMedica) together with the Steering Committee and conducted under US and India Investigational New Drug Applications with oversight from an independent data monitoring committee. The investigators gathered the data, and the trial funder (CalciMedica) undertook site monitoring and data collation, with leadership and scientific supervision from the Steering Committee. The statistical analysis was undertaken by a contract research organisation (Princeton Pharmatech), in collaboration with the sponsor and Steering Committee. The authors were responsible for data interpretation for the manuscript, which was drafted by the first author and all coauthors contributed to its content.

## Results

**Fig. 1:**
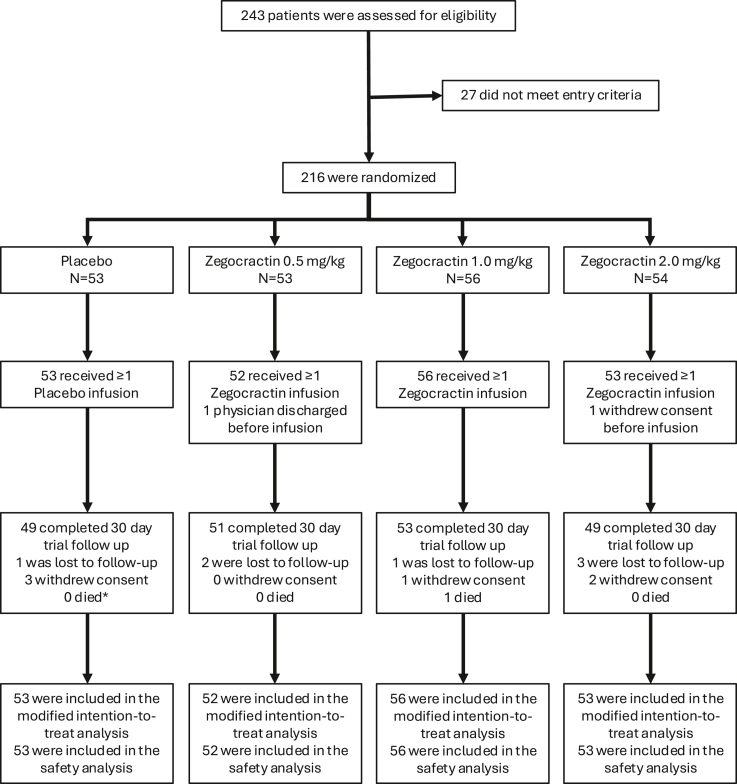
Trial profile. ∗1 patient died after consent was withdrawn.

From March 2021 through May 2024, 243 consecutive patients with AP and SIRS were fully screened at 58 sites in the US and India, of whom 216 were enrolled across 37 centres. In the US, 152 patients were randomised, and in India, 64; 53 were assigned to placebo and 163 to either low (N = 53), medium (N = 56), or high dose (N = 54) zegocractin (Fig. 1). The median (interquartile range; IQR) patient age was 43 (34, 57) years and median (IQR) time from the onset of abdominal pain to randomisation was 3 (2, 4) days. Overall, the 4 trial groups were balanced except for statistically fewer patients ≥65 years of age in the high dose and placebo groups compared to the low and medium groups (Table 1).

**Table 1:** Characteristics of the patients at baseline.^a^

Characteristics	Placebo group (N = 53)	Zegocractin, 0.5 mg (N = 52)	Zegocractin, 1.0 mg (N = 56)	Zegocractin, 2.0 mg (N = 53)
Median age (IQR)—yr	42 (32, 56)	48.5 (35.5, 63.5)	43.5 (34, 62.5)	42 (32, 51)
Age group—no. (%)				
18–39 yr	23 (43.4)	19 (36.5)	22 (39.3)	25 (47.2)
40–64 yr	24 (45.3)	21 (40.4)	22 (39.3)	23 (43.4)
≥65 yr	6 (11.3)	12 (23.1)	12 (21.4)	5 (9.4)
Geographic region—no. (%)				
US	36 (67.9)	37 (71.2)	40 (71.4)	37 (69.8)
India	17 (32.1)	15 (28.8)	16 (28.6)	16 (30.2)
Female sex—no. (%)	20 (37.7)	20 (38.5)	23 (41.1)	20 (37.7)
Race or ethnic group—no. (%)				
American Indian Native	1 (1.9)	0	0	0
Asian	20 (37.7)	16 (30.8)	17 (30.4)	16 (30.2)
Black	8 (15.1)	8 (15.4)	10 (17.9)	13 (24.5)
Hawaiian Native	1 (1.9)	0	0	0
White	15 (28.3)	22 (42.3)	26 (46.4)	21 (39.6)
Other	8 (15.1)	6 (11.5)	3 (5.4)	3 (5.7)
Hispanic or Latino ethnic group (%)	13 (24.5)	14 (26.9)	8 (14.3)	10 (18.9)
Median (IQR) BMI	28.9 (23.3, 37.0)	28.4 (24.2, 31.0)	27.8 (25.3, 34.3)	28.3 (24.7, 34.2)
Consume alcohol—no. (%)	28 (52.8)	26 (50)	30 (53.6)	28 (52.8)
Median (IQR) drinks/week	10 (3.5, 14.0)	13.0 (3.0, 30.0)	6.5 (3.0, 16.0)	7 (2.0, 18.0)
Median (IQR) onset of pain to first infusion—days	3.0 (2.0, 4.0)	2.0 (2.0, 3.0)	3.0 (2.0, 5.0)	2.0 (2.0, 4.0)
SIRS criteria—no. (%)				
2	42 (79)	41 (79)	41 (73)	39 (74)
≥3	11 (21)	11 (21)	15 (27)	14 (26)
Abdominal guarding or rebound—no. (%)	37 (69.8)	35 (67.3)	44 (78.6)	38 (71.7)
Balthazar Score				
A–C	18 (34.0)	13 (25)	20 (35.7)	16 (30.2)
D	5 (9.4)	11 (21.2)	9 (16.1)	8 (15.1)
E	30 (56.6)	27 (51.9)	27 (48.2)	28 (52.8)
High haematocrit—no. (%)^b^	20 (37.7)	24 (46.2)	25 (44.6)	23 (43.4)
Organ failure at screening^c^				
Respiratory failure	6 (11.3)	4 (7.7)	4 (7.1)	3 (5.7)
Cardiovascular failure	0	0	0	0
Renal failure	3 (5.7)	3 (5.8)	2 (3.6)	2 (3.8)
Etiology—no. (%)				
Alcohol	18 (34.0)	22 (42.3)	25 (44.6)	26 (49.1)
Biliary	14 (26.4)	10 (19.2)	11 (19.6)	8 (15.1)
HTG	9 (17.0)	5 (9.6)	4 (7.1)	4 (7.5)
Other	4 (7.5)	5 (9.6)	5 (8.9)	5 (9.4)
Unknown	8 (15.1)	10 (19.2)	10 (17.9)	10 (18.9)

a Data are for the modified intention-to-treat population. The abbreviation BMI denotes body mass index, ED Emergency Department, HTG hypertriglyceridaemia, IQR interquartile range, N number of patients, SIRS systemic inflammatory response syndrome, and US United States.

b ≥44% for men, ≥40% for women.

c Defined by a score of ≥2 on the modified Marshall scoring system.^25^

At least 1 trial infusion was started in 53 patients in the placebo group and in 52 patients in the low, 56 in the medium, 53 in the high dose zegocractin groups, resulting in a mITT population of 214 patients. 34 patients did not receive all three doses of study drug (Table S2 in the Appendix p 10). Trial treatment was stopped early because of adverse events in 3 (5.7%) of 53 patients in the placebo group and in 2 (3.8%) of 52, 1 (1.8%) of 56 and 2 (3.7%) of 53 patients in the low, medium, and high dose zegocractin groups, respectively. Physician discharge led to early discontinuation of study drug in 2 (3.8%) patients in the placebo group and in 2 (3.8%), 6 (10.7%) and 4 (7.4%) patients in the low, medium, high dose zegocractin groups, respectively (Table S2).

In the mITT population, the median time to solid food tolerance was similar following treatment with placebo compared to all doses of zegocractin (Table 2). In the subgroup of 92 (43.0%) patients with a high haematocrit, all doses of zegocractin resulted in a shorter time to solid food tolerance, with medians of 78.0, 64.0 and 67.0 h for low, medium and high doses, respectively, versus 113.5 h for placebo; in these patients a dose–response relationship was demonstrated by gMCP-Mod analysis (p = 0.057) (Fig. 1A). In the subgroup of 145 (67.8%) patients with screening Balthazar scores of D or E, all doses of zegocractin resulted in a shorter time to solid food tolerance, with medians of 68.5, 68.5, and 66.0 h for low, medium, and high doses, respectively, versus 112.0 h for placebo (Table 2). Patients with a low haematocrit, or screening Balthazar scores of A, B, or C, had similar solid food tolerance following zegocractin versus placebo (Table S3 in the Appendix p 12).

**Table 2:** Trial endpoints.^a^

Outcome	Placebo group (N = 53)	Zegocractin, 0.5 mg (N = 52)	Zegocractin, 1.0 mg (N = 56)	Zegocractin, 2.0 mg (N = 53)
Median (IQR) hours to solid food tolerance				
mITT population	66.0 (38.0, 156.0)	78.0 (25.5, 184.0)	64.0 (21.5, 133.5)	67.0 (25.0, 117.0)
High haematocrit at baseline^b^	N = 20 (37.7)	N = 24 (46.2)	N = 25 (44.6)	N = 23 (43.4)
	113.5 (41.5, 187.0)	78.0 (37.0, 187.5)	64.0 (20.0, 113.0)	67.0 (13.0, 117.0)
Hazard ratio (95% CI)^c^		1.306 (0.703, 2.426)	1.927 (1.016, 3.655)	1.595 (0.847, 3.004)
Balthazar D or E at baseline^d^	N = 35 (66)	N = 38 (73.1)	N = 36 (64.3)	N = 36 (67.9)
	112.0 (45.0, 210.0)	68.5 (27.0, 181.0)	68.5 (20.0, 133.5)	66.0 (25.5, 120.0)
Hazard ratio (95% CI)^c^		1.330 (0.806, 2.194)	1.339 (0.810, 2.214)	1.412 (0.860, 2.319)
Organ failure—no. (%)^e^				
Any severe organ failure	5 (9.4%)	5 (9.6%)	2 (3.6%)	2 (3.8%)
Percent difference (95% CI)^f^		−0.1 (−11.4, 11.1)	−5.9 (−15.3, 3.4)	−5.9 (−15.3, 3.5)
Severe respiratory failure	5 (9.4%)	5 (9.6%)	2 (3.6%)	2 (3.8%)
Severe cardiovascular failure	1 (1.9%)	3 (5.8%)	1 (1.8%)	1 (1.9%)
Severe renal failure	1 (1.9%)	2 (3.8%)	1 (1.8%)	0
New-onset severe respiratory failure	4 (8.5)	4 (8.3)	0	0
Percent difference (95% CI)^f^		−1.3 (−12.1, 9.5)	−8.4 (−16.4, −0.4)	−8.4 (−16.4, −0.4)
Necrotising pancreatitis^g^	N = 47	N = 47	N = 52	N = 41
Necrotising pancreatitis at baseline	1 (2.1)	3 (6.4)	3 (5.8)	4 (9.8)
New-onset necrotising pancreatitis	17 (37.0)	17 (38.6)	20 (40.8)	11 (29.7)
Percent difference (95% CI)^f^		1.1 (−17.9, 20.1)	2.2 (−16.9, 21.3)	8.0 (−27.6, 11.6)
Infected pancreatic necrosis at Day 30	1	0	0	1
Time to medically indicated discharge				
Median (IQR) hours	104.0 (50.0, 210.0)	109.5 (53.5, 280.0)	104.5 (50.5, N/A^h^)	89.0 (42.0, 204.0)
Hazard ratio (95% CI)^c^		0.916 (0.604, 1.389)	0.760 (0.498, 1.159)	1.243 (0.826, 1.869)
Indicated at 96 h—no. (%)	26 (49.1)	25 (48.1)	27 (48.2)	29 (54.7)
Length of hospital stay				
Median (IQR) days	5.0 (4.0, 8.0)	5.5 (4.0, 8.5)	5.0 (3.0, 7.0)	4.0 (4.0, 7.0)
Least squares mean (95% CI)^i^	7.20 (5.75, 8.66)	7.64 (6.18, 9.10)	5.92 (4.52, 7.32)	5.94 (4.48, 7.40)
Hospital stay >21 days—no. (%)	3 (5.7)	3 (5.8)	1 (1.8)	0 (0.0)

a Data are median values or number of patients. All analyses were performed in the modified intention-to-treat (mITT) population. IQR denotes interquartile ranges. N and no. denote number of patients.

b ≥44% for men, ≥40% for women. One patient in the 2.0 mg zegocractin group did not have a screening haematocrit.

c Stratified Cox proportional hazard model with treatment as the independent variable and stratified by sex and haematocrit.

d One patient in the 0.5 mg zegocractin group and one in the 2.0 mg zegocractin group were without a screening Balthazar score.

e Number and percentage of patients with new-onset organ failure among all patients who did not have organ failure at presentation.

f Cochran-Mantel-Haenszel test zegocractin versus placebo stratified analysis.

g Number of patients with CECT/MRI of sufficient quality to be read accurately by the central panel at both baseline and Day 30, or if unavailable, post-trial treatment standard care CECT/MRI. Patients with new-onset necrotising pancreatic were the number and percentage of patients who did not have necrotising pancreatitis at screening determined by the central panel.

h Upper limit not applicable as 1.0 mg zegocractin group included patients who did not tolerate solid food throughout the 30-day period of the trial.

i ANOVA model.

**Fig. 2:**
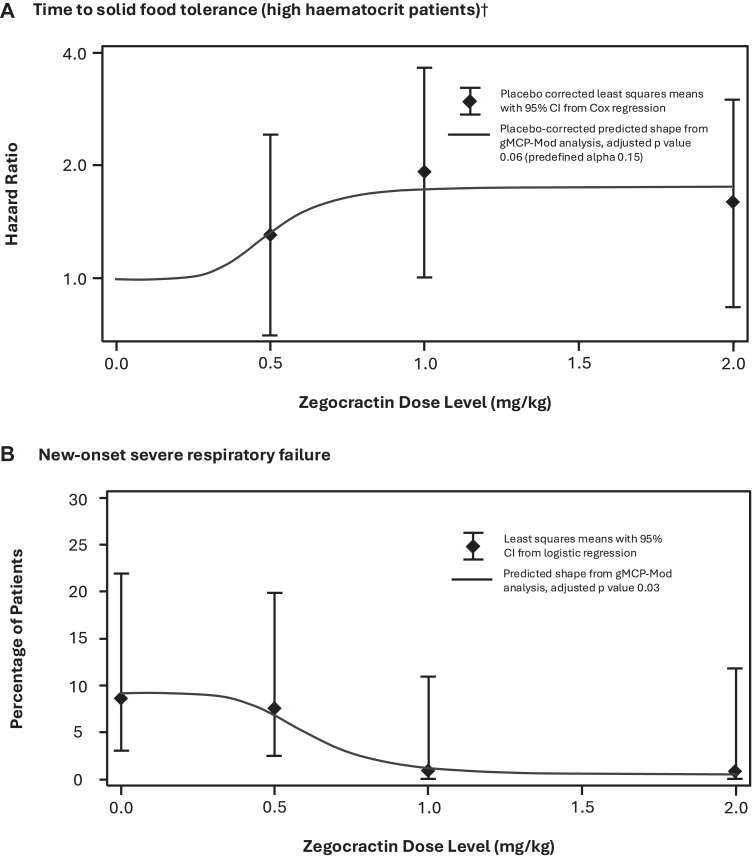
Dose–response relationship of zegocractin to outcome∗. A. Relationship between zegocractin and time to solid food tolerance in patients with a high haematocrit at presentation. B. Relationship between zegocractin and new-onset severe respiratory failure in the whole mITT population. ∗Analysis using the generalised multiple comparisons and modeling (gMCP-Mod) approach as qualified by regulatory agencies (displayed as sigEmax, see the statistical analysis plan in the Appendix p 1), by which a dose–response is confirmed with the maximum contrast test if statistically significant at a defined level. †The hazard ratios in A increase as solid food tolerance increases (i.e. time to solid food tolerance is shortened) with increasing doses of zegocractin.

In patients without respiratory failure at screening, none developed severe respiratory failure in the medium (52 patients) and high (50 patients) dose groups, versus 4 (8.5% of 47 patients) and 4 (8.3% of 48 patients) in the placebo and low dose groups, respectively. A dose–response relationship was demonstrated for zegocractin by gMCP-Mod analysis of new-onset severe respiratory failure (p = 0.029) (Fig. 2B).

Although the statistical analysis plan allowed for analyses of 11 subgroups, because dose–response was demonstrated in the entire mITT population for severe respiratory failure and for solid food tolerance in those at higher risk of organ failure with a high haematocrit or Balthazar D or E, further subgroup analyses (age, race, sex, aetiology, SIRS, hypoxaemia, white cell count, country of enrolment) were considered unlikely to be informative and therefore not performed.

Patients receiving medium or high dose zegocractin had less frequent organ failure, predominantly severe respiratory failure (Table 2). All organ failure occurred in patients who had either a high haematocrit or peripancreatic fluid at screening, except in one. One patient treated with placebo died from multiorgan failure and one patient treated with medium dose zegocractin died secondary to acute coronary syndrome. There were no deaths in the low or high dose groups.

By day 30, new-onset necrotising pancreatitis in 11 of 37 (29.7%) patients in the high dose group was lower than in 17 of 46 (37%), 17 of 44 (38.6%) and 20 of 49 (40.8%) in the placebo, low dose and medium dose groups, respectively. One patient in both the placebo and high dose groups was found to have imaging consistent with infected pancreatic necrosis at day 30 (Table 2).

The median time to medically indicated discharge of 89.0 h in the high dose group was lower than 104.0, 109.5 and 104.5 h in the placebo, low dose and medium dose groups, respectively (Table 2). The median length of hospital stay of 4.0 days for the high dose group was lower than 5.0, 5.5 and 5.0 days for the placebo, low dose and medium dose groups, respectively (Table 2). The number of patients discharged by days 7, 14 and 21 was consistently higher in the medium and high dose groups versus the low dose and placebo groups (Table S4 in the Appendix p 13). No patient in the high dose group was hospitalised more than 21 days.

**Fig. 3:**
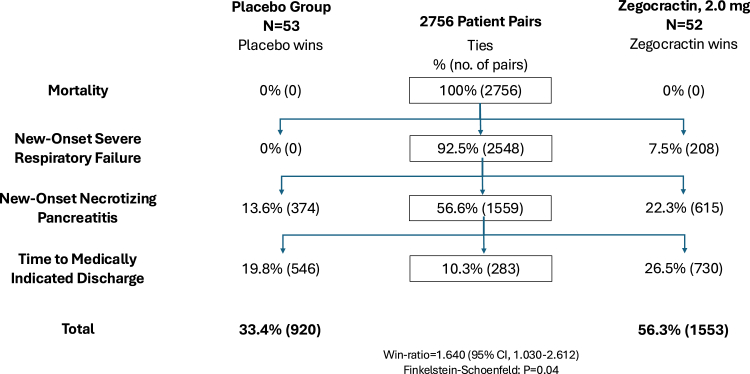
Win-ratio for placebo versus 2.0 mg zegocractin∗. ∗1 patient in the placebo group died after consent had been withdrawn, and 1 patient in the zegocractin group did not have a baseline haematocrit and has been excluded.

In the exploratory stratified win-ratio analysis of the hierarchical composite endpoint, there were 1553 total wins for the high dose group versus 920 for placebo (win-ratio 1.64 [95% CI 1.030–2.612], p = 0.04, Fig. 3), whereas neither the low (win-ratio 1.123 [95% CI 0.708–1.781], p = 0.62) nor the medium dose (win-ratio 1.177 [95% CI 0.740–1.871], p = 0.50) group won statistically against placebo.

The incidence of adverse and serious adverse events was higher in the zegocractin groups overall versus placebo but was similar across the high dose and placebo groups, without a pattern suggestive of a dose–response relationship (Table 3). The low and medium dose groups, among whom adverse events were commoner, were older (Table 1) and pharmacokinetic analysis did not show serious adverse events to be increased by increasing drug exposure (Figure S1, see statistical considerations in the Appendix p 6). An adverse event led to discontinuation of trial drug in 3 patients in the placebo group and in 2 patients in each zegocractin group. An increase in alanine aminotransferase, aspartate aminotransferase and total bilirubin classified as three serious adverse events in 1 (1.9%) patient in the low dose group were believed by the investigator to be related to trial treatment. Metabolic acidosis and multiorgan failure preceded death in 1 patient in the placebo group, and acute coronary syndrome preceded death in 1 patient in the medium dose group, not considered related to the trial treatment.

**Table 3:** Summary of adverse events.^a^

Characteristics	Placebo group (N = 53)	Zegocractin, 0.5 mg (N = 52)	Zegocractin, 1.0 mg (N = 56)	Zegocractin, 2.0 mg (N = 53)
	number of patients (percent)			
Any adverse event	25 (47.2)	28 (53.8)	36 (64.3)	23 (43.4)
Adverse event related to zegocractin or placebo	5 (9.4)	9 (17.3)	6 (10.7)	4 (7.5)
Serious adverse event	6 (11.3)	13 (25.0)	12 (21.4)	8 (15.1)
Serious adverse event related to zegocractin or placebo	0	1 (1.9)	0	0
Adverse event leading to discontinuation of trial treatment	3 (5.7)	2 (3.8)	2 (3.6)	2 (3.8)
Adverse events that resulted in death	1 (1.9)	0	1 (1.8)	0
Adverse events with an incidence ≥5% in the pooled zegocractin groups				
Blood and lymphatic system disorders	5 (9.4)	5 (9.6)	4 (7.1)	4 (7.5)
Anaemia	3 (5.7)	2 (3.8)	3 (5.4)	3 (5.7)
Cardiac disorders	1 (1.9)	5 (9.6)	5 (8.9)	2 (3.8)
Gastrointestinal disorders	15 (28.3)	13 (25.0)	18 (32.1)	11 (20.8)
Constipation	6 (11.3)	3 (5.8)	5 (8.9)	1 (1.9)
Acute pancreatitis	0	2 (3.8)	3 (5.4)	4 (7.5)
Diarrhoea	3 (5.7)	2 (3.8)	3 (5.4)	3 (5.7)
Abdominal pain	1 (1.9)	2 (3.8)	2 (3.6)	4 (7.5)
General disorders	5 (9.4)	6 (11.5)	9 (16.1)	9 (17.0)
Pyrexia	2 (3.8)	3 (5.8)	3 (5.4)	3 (5.7)
Infections	4 (7.5)	5 (9.6)	8 (14.3)	2 (3.8)
Metabolic and nutritional disorders	6 (11.3)	5 (9.6)	5 (8.9)	7 (13.2)
Hypokalaemia	0	1 (1.9)	4 (7.1)	3 (5.7)
Psychiatric disorders	4 (7.5)	5 (9.6)	2 (3.6)	5 (9.4)
Renal and urinary disorders	2 (3.8)	4 (7.7)	3 (5.4)	2 (3.8)
Respiratory disorders	6 (11.3)	8 (15.4)	8 (14.3)	4 (7.5)
Pleural effusion	4 (7.5)	5 (9.6)	3 (5.4)	3 (5.7)
Skin disorders	1 (1.9)	3 (5.8)	2 (3.6)	3 (5.7)

a Data compiled from the modified intention-to-treat (mITT) population. N denotes number of patients in each group.

## Discussion

In this phase 2 trial gMCP-MOD analysis of the mITT population did not demonstrate a dose–response relationship for the primary outcome of time to solid food tolerance but demonstrated a dose–response relationship for the secondary outcome of severe respiratory failure, and although a secondary outcome, severe respiratory failure carries major clinical significance. An analysis of subgroups defined a priori by the additional inclusion criteria of a high haematocrit or peripancreatic fluid collection (Balthazar D or E score on CECT or MRI at screening) found zegocractin to be associated with a reduction in time to solid food tolerance. All patients but one who developed organ failure had either a high haematocrit or peripancreatic fluid at screening and all organ failure was found to be less frequent in the medium and high dose groups. These findings suggest that the benefit from zegocractin in patients with AP and SIRS is most apparent in those who have additional clinical evidence of severe inflammation and are at risk for organ failure. Organ failure, most frequently respiratory, is a major complication of AP that is a principal risk factor for, contributes to, and precedes most mortality from AP.^1^^,^^4^^,^^5^ No drug has been shown previously to reduce the development of organ failure in patients with AP.^1^, ^2^, ^3^, ^4^, ^5^^,^^7^^,^^8^^,^^14^ A combination of the cyclooxygenase inhibitors parecoxib and imrecoxib reduced the occurrence and duration of severe AP but did not reduce the development of organ failure or use of mechanical ventilation compared to placebo in patients with predicted severe AP.^26^ Use of these two medicines is not an option in many countries as neither the FDA nor EMA has approved imrecoxib and the FDA has not approved parecoxib.^27^

The 2.0 mg/kg dose of zegocractin was associated with a reduction in necrotising pancreatitis at Day 30, length of stay notably prolonged hospital stays >21 days, and time to medically indicated discharge, compared to other dose groups and placebo. Time to medically indicated discharge is an endpoint designed to standardise discharge criteria for patients with AP across different sites and was first proposed in the PYTHON trial.^20^ An exploratory composite endpoint integrating patient outcomes, specifically mortality, new-onset respiratory failure, new-onset necrotising pancreatitis, and time to medically indicated discharge, was developed in CARPO as a potential confirmatory trial endpoint and tested using the win ratio method.^23^^,^^24^ The comparison of drug doses to placebo was consistent with an advantage of the 2.0 mg/kg dose over the 1.0 and 0.5 mg/kg doses. Additionally, the 2.0 mg/kg dose had a similar safety and tolerability profile to placebo. These safety data add to those of the phase 2 CARDEA trial of zegocractin in COVID-19 pneumonia, which in addition to significant beneficial effects on mortality over 30 days, found fewer adverse events in 130 patients receiving zegocractin at 2.0 mg/kg then 1.6 mg/kg (each given once daily for 3 days) than in 131 patients receiving placebo.^28^

The CARPO trial has limitations. First, time to solid food tolerance did not demonstrate a dose–response relationship for zegocractin in the whole mITT population. A dose–response was however seen in data for time to solid food tolerance in patients with a high haematocrit, as in data for new-onset severe respiratory failure in the mITT population. Time to solid food tolerance was chosen as the primary endpoint to assess dose–response because every patient enrolled would contribute to the analysis but may have lacked discriminative ability compared to the exploratory secondary end points of organ failure and necrotising pancreatitis, both of which were improved across the whole mITT population following zegocractin. In addition, time to solid food tolerance may have been subject to the benefits of an enhanced recovery strategy promoting early tolerance in patients treated with placebo.^14^ Alternatively, zegocractin may have greater efficacy in patients who have a more markedly increased inflammatory state, as indicated by an elevated haematocrit or high (D or E) Balthazar score. Second, trial treatment was incomplete in 34 of 216 patients, although early efficacy of medium and high dose zegocractin, begun within a median of 3 or 2 days from the onset of pain respectively, might have contributed to this, and so to early discharge. Third, the absence of adjustments for multiplicity requires caution in the interpretation of differences between the four trial treatment arms, as without such adjustments, the secondary endpoints are exploratory. The direction of improvement of all outcomes, however, was associated with the administration of zegocractin, most consistently at high dose, reflected in the win-ratio, and indicative of proportionality assumed in the use of hazard ratios in the gMCP-Mod analysis. As hypothesis generating, these data provide justification for a pivotal phase 3 trial of zegocractin. Fourth, global generalisability is undetermined, with short lengths of stay typical of the US health system, despite recruitment of patients with more severe AP. Nevertheless consistent results from patients with different ethnicities from the US and India point to a greater impact of ORAI1 inhibition common to patients with more severe AP, the clinical features of which are similar throughout the world.^1^^,^^4^^,^^5^

From 1965, three decades of negative randomised trials of drugs in AP prompted search for a mechanism fundamental in AP to target,^2^^,^^6^^,^^29^ leading to discovery of a critical role for abnormal pancreatic parenchymal cell overload with calcium,^3^^,^^7^^,^^8^ normal signaling of which is essential to exocrine secretion.^8^ Since discovery of the role of CRAC channel calcium entry in AP, three further decades of randomised trials of drugs targeting other mechanisms have not resulted in a licenced therapeutic for AP.^1^, ^2^, ^3^, ^4^, ^5^ The findings from CARPO provide credence for the essential role of cellular calcium entry and overload in the development of AP, and point to this mechanism as a tractable target, demonstrating a dose–response for zegocractin in patients with AP and SIRS. Furthermore, this trial has earmarked patients with AP at higher risk of organ failure who are likely to benefit most from zegocractin, suggesting an effective dose at 2 mg/kg and suitable endpoints with a win ratio to evaluate effectiveness,^30^ which if established would overcome hurdles that have hindered drug development for AP over so many years.

## Contributors

Conceptualisation: RS, PKG, KS, SH, BUW, WFP, TBG; data curation: JZ, KS, SH; formal analysis: JZ, SH; funding acquisition: KS, SH; investigation: JM, SSK, JLB, MP, SH; methodology: JZ, SH; project administration: RS, PKG, SH, BUW, WFP, TBG; resources: JM, SSK, JLB, MP, JZ, KS, SH; software: JZ; supervision: RS, PKG, SH, BUW, WFP, TBG; validation: RS, PKG, JZ, SH, BUW, WFP, TBG; visualisation: RS, SH; writing—original draft: RS; writing—review & editing: RS, PKG, JM, SSK, JLB, MP, JZ, KS, SH, BUW, WFP, TBG.

## Data sharing statement

We do not plan to share the source datasets as these have been submitted for regulatory review.

## Declaration of interests

RS: support from MRC/NIHR EME 15/20/01 (funding for the RAPID-I Trial (NCT03684278) from the EME Programme (infliximab supplied by Merck/MSD until 30 September 2024, since when funded by NIHR via Excess Treatment Costs) 2018–2027); Innovative Medicines Initiative 2 (Translational Safety Biomarker Pipeline Consortium 2019–2026, membership of which includes academic medical centres, small sized enterprises, and Janssen, Lilly, Merck/MSD, Novartis, Pfizer, Roche, and Sanofi-Aventis; for this work the University of Liverpool received supplementary funding from Lilly, Merck/MSD and Pfizer for technical support); NIHR (Senior Investigator Award (Second Term) 2022–2025); LAP Research (Drug discovery to inhibit cyclophilin D as a treatment for acute pancreatitis 2022–2024); CalciMedica (funding for CARPO and the CARPO Investigators; Chair of Steering Committee for CARPO trial, work for which payments have been made to the University of Liverpool for time, payments which have been used to support medical research; support for presentation of results of CARPO trial at the American College of Gastroenterology, Philadelphia, October 2024). PKG: Indian Council of Medical Research (research funding); Department of Biotechnology, Government of India (research funding); past secretary, International Association of Pancreatology (honorary, unpaid). JZ: CalciMedica (employed by Princeton Pharmatech LLC, who received consulting fee from Calcimedica for conducting statistical analysis for this manuscript; also stock options). KS: CalciMedica (employment salary and stock options; travel expenses; patents and pending applications related to the treatment of acute pancreatitis with CRAC channel inhibitors). SH: CalciMedica (patent applications files and granted; stock and stock options).
