# Supplementary material for: Zegocractin for acute pancreatitis with systemic inflammatory response syndrome: a randomized, controlled, dose-ranging, phase 2b trial
Source: eClinicalMedicine. 2026 Feb 23;93:103757. doi: 10.1016/j.eclinm.2026.103757 (PMC13043320; doi:10.1016/j.eclinm.2026.103757)
Supplement: Appendix [file mmc1.pdf]

Zegocractin for acute pancreatitis with systemic inflammatory response syndrome: a randomised, controlled, dose-ranging, phase 2b trial

Appendix

Contents

CARPO Investigators…………………………………………………………………………….. 2

CARPO Radiology Panel………………………………………………………………………… 3

CARPO Steering Committee……………………………………………………………….. 4

CARPO Senior Research Personnel………………………………………………………….. 4

Statistical Considerations……………………………………………………………………... 5

Table S1………………………………………………………………………………………. 9

Table S2………………………………………………………………………………………. 10

Table S3………………………………………………………………………………………. 12

Table S4………………………………………………………………………………………. 13

Subsequent documents displayed in the order below are paginated separately, with each page identifying to which document each belongs

CARPO Protocol Version 2.0………………………………………………………………… 79 pages

CARPO Protocol Version 4.0………………………………………………………………… 87 pages

Summary of Changes with Rationale V2.0 to V4.0 ……………………………………… 20 pages

Statistical Analysis Plan Version 1.0…………………………………………………… 31 pages

INVESTIGATORS	
**USA**	
Jason Bischof	Ohio State University Wexner Medical Center, Columbus, OH
Charles Bruen	Regions Hospital, St. Paul, MN
Harleen Chela	CAMC Memorial Hospital, Charleston, WV
Gerald Dryden	Gerald Dryden, Robley Rex Va Medical Center, Louisville, KY
James D'Etienne	John Peter Smith Hospital, Fort Worth, TX
Thomas Hartka	University Medical Center, Charlottesville, VA
Stacey House	Barnes Jewish Hospital, St. Louis, MO
Raj Keswani	Northwestern Memorial Hospital, Chicago, IL
Paul Sanchez Leon	Houston Methodist Hospital, Houston, TX
Maher Madhoun	Stamford Hospital, Stamford, CT
James McKinnell	Torrance Memorial Medical Center, Torrance, CA
Karen Miller	St. Luke's Boise Medical Center, Boise, ID
Syed H. Naqvi	University Hospital, Columbia, MO
Stephen Pandol	Cedars-Sinai Medical Center, Los Angeles, CA
Brant Putnam	Harbor-UCLA Medical Center, Torrance, CA
David Robinson	Memorial Hermann-Texas Medical Center, Houston, TX
Daniel Rolston	North Shore University Hospital, Manhasset, NY
Ibrahim Saad	Sarasota Memorial Hospital, Sarasota, FL
Jason Samarasena	UCI Medical Center, Orange, CA
Jonathan Schimmel	The Mount Sinai Hospital, New York, NY
Michael Schnaus	Methodist Hospital, St. Louis Park, MN
Amber Thacker	Regional Medical Center, Memphis, TN
David Wein	Tampa General Hospital, Tampa, FL
Gentry Wilkerson	University of Maryland Medical Center, Baltimore, MD

Fady Youssef	Memorial Care Long Beach Medical Center, Long Beach, CA
**INDIA**	
Sunil Kumar Dadhich	Mathuradas Mathur Hospital, Jodhpur, Rajasthan
Ashish Joshi	Sardar Patel Medical College and Associated Group of Hospitals, Bikaner, Rajasthan
Kiran Josy	Lisie Hospital, Kochi, Kerala
Bahar Kulkarni	Sanjeevan Hospital, Pune, Maharashtra
P. Kranthi Kumar	Malla Reddy Narayana Multispeciality Hospital, Hyderabad, Telangana
Prashant Rahate	Seven Star Hospital, Nagpur, Maharashtra
Brij Sharma	Indira Gandhi Medical College and Hospital, Shimla, Himachal Pradesh
Saroj K. Sinha	Postgraduate Institute of Medical Education and Research, Nehru Hospital, Chandigarh
Pratibha Sonawane	Vasantrao Pawar Medical College, Hospital and Research Center, Nashik, Maharashtra
Devang Tank	Shree Giriraj Multispeciality Hospital, Rajkot, Gujarat

**CENTRAL RADIOLOGY PANEL**	
**Name**	**Affiliation**
Thomas L. Bollen	Department of Radiology, St Antonius Hospital, Nieuwegein, Netherlands
Erik J.R.J. van der Hoeven	Department of Radiology, St. Antonius Hospital, Nieuwegein, Netherlands

STEERING COMMITTEE	
**Name**	**Affiliation**
Timothy B. Gardner	Section of Gastroenterology and Hepatology, Dartmouth Hitchcock Medical Center, Lebanon, NH, USA
Pramod K. Garg	Department of Gastroenterology, All India Institute of Medical Sciences, New Delhi 110029, India
Sudarshan Hebbar	CalciMedica, La Jolla, CA, USA
W. Frank Peacock	Henry J.N. Taub Department of Emergency Medicine, Baylor College of Medicine, Houston, TX, USA
Robert Sutton	University of Liverpool and Liverpool University Hospitals NHS Foundation Trust, Liverpool, UK
Bechien Wu	Southern California Permanente Medical Group, Los Angeles, CA, USA

SENIOR RESEARCH PERSONNEL	
**Name**	**Affiliation**
Andrew Cunningham	Senior Vice-President, Clinical Development, CalciMedica, La Jolla, CA
Liisa Tingue	Senior Director, Clinical Operations, CalciMedica, La Jolla, CA
Katherine Randolph	Senior Director, Clinical Operations, CalciMedica, La Jolla, CA
Jen Otto	Executive Vice-President, Clinical Development, Bionical Emas, Paulsboro, NJ
Ramprasath TR	Senior Project Manager, JSS Medical Research, Quebec H7V 0A3, Canada
Arumuga Rajan	Assistant Project Manager, JSS Medical Research, Quebec H7V 0A3, Canada
Mary Dymond	President, Safety Sphere, Pearland, TX
Ashley Hehr	Senior Drug Safety Specialist, Safety Sphere, Houston, TX
Harvey Yuan	Senior Programmer, Princeton Pharmatech, Princeton, TX

STATISTICAL CONSIDERATIONS

The trial sample size calculation was based on the nQuery software STT0-1 function of log-rank test of survival (time to solid food tolerance) for pairwise two treatment group comparison without multiplicity adjustment. For randomisation a stratified Cox regression of the time to solid food tolerance at each dose level was used, stratified by the recorded sex (male or female) and then by risk for organ failure based on data in the sex subgroups (higher or lower). A lower risk of organ failure was defined as by the absence of either or both an elevated haematocrit and hypoxaemia.

To demonstrate the dose-response relationship between treatment with zegocractin compared to placebo for the primary endpoint of time to solid food tolerance for the modified intention to treat (mITT) population or predefined subgroup with high haematocrit on presentation, the analysis was based on generalized the Multiple Comparisons and Modeling (gMCP-Mod) method.[1,2] This procedure tests for a dose-response relationship, allowing for uncertainty in the dose-response relationship through inclusion of contrasts from multiple pre-specified candidate models assessing dose-response. This method is qualified by the European Medicines Agency (EMA)[3] and US Food and Drug Administration (FDA)[4] for use in phase 2 clinical trial analysis to satisfy regulatory requirements for demonstrating dose-response. The target dose(s) suggested by gMCP-Mod serves as an important consideration in dose selection, also considering the totality of efficacy data and safety endpoints including adverse events. The gMCP-Mod procedure extends the MCP-Mod methodology to non-continuous endpoints. This is accomplished by decoupling the dose-response model from the expected response using an ANOVA-style parameterisation of the dose-response parameter.

For the time-to-event endpoint used in this study, a Cox regression of the time to solid food tolerance at each dose level was used, stratified by the recorded sex (male or female) and then by risk for organ failure based on data in the sex subgroups (higher or lower). The mean estimates of log hazard ratio from the Cox regression model and the corresponding covariance matrix, both of which are on the log scale, were then used during the rest of the gMCP-Mod procedure, based on the assumption of proportional hazards. Point estimates from the selected models were displayed on the ratio scale in statistical outputs. The gMCP-Mod method was not used in the sample size calculation.

The gMCP-Mod method was employed in a three-step approach:

Step 1: A conventional step was used to determine the hazard ratio between each dose versus placebo. The data were analysed using stratified Cox regression to obtain treatment effects and a corresponding variance covariance

matrix. The estimated treatment hazard ratio and associated 95% confidence intervals were determined for the treatment contrast of each dose of zegocractin versus placebo.

Step 2: A proof-of-concept step was applied as a candidate models and multiple testing procedure. The null hypothesis of a flat dose-response relationship as compared to placebo for the primary efficacy endpoint was tested at a significance level of one-sided 15% against the alternative hypothesis of a non-constant dose-response curve using a multiple contrast test as described in gMCP-Mod methodology. The contrast test statistic is a linear combination of the optimal contrast coefficients corresponding to the candidate dose-response curve with the hazard ratio at each individual dose obtained from Step 1. An alpha of 0.15, as set in CARPO, has been used in other phase 2 trials (e.g. NCT02671461) for the demonstration of dose response. Proof of concept was established if at least one dose-response model was statistically significant, thereby rejecting the null hypothesis of a flat dose-response curve and indicating a benefit of zegocractin over placebo.

Step 3: A dose-finding step was undertaken with dose-response model fitting. Once the significance of a dose-response signal was established, the dose-response profile and target dose were estimated using the best-fitting dose-response model. The dose-response curve was plotted graphically (Figure 2A), including the mean responses from the Cox regression and association 95% confidence intervals for each of the dose groups.

The predefined gMCP-Mod analysis of severe respiratory failure was conducted in the same manner as described for time to solid food tolerance, using odds ratios between each dose versus placebo (Figure 2B). Logistic regression was undertaken with treatment, sex and the risk of organ failure as co-variates to obtain treatment effects and the corresponding covariance matrix for Step 1, undertaking Steps 2 and 3 as for solid food tolerance.

As the incidence of serious adverse events was higher in the low and medium dose groups, pharmacokinetic analysis was undertaken to determine whether drug exposure related to the development of any serious adverse event after the start of trial treatment (treatment emergent serious adverse events, TESAEs). This analysis showed that serious adverse events were not increased by increasing zegocractin exposure over 72 hours from the start of the first of three daily zegocractin infusions, assessed by zegocractin area under the curve (AUC as ng·h/mL, see Figure S1). The high AUC group had a slightly lower incidence of serious adverse events.

Figure S1 **Zegocractin exposure-response relationship to any serious adverse event**

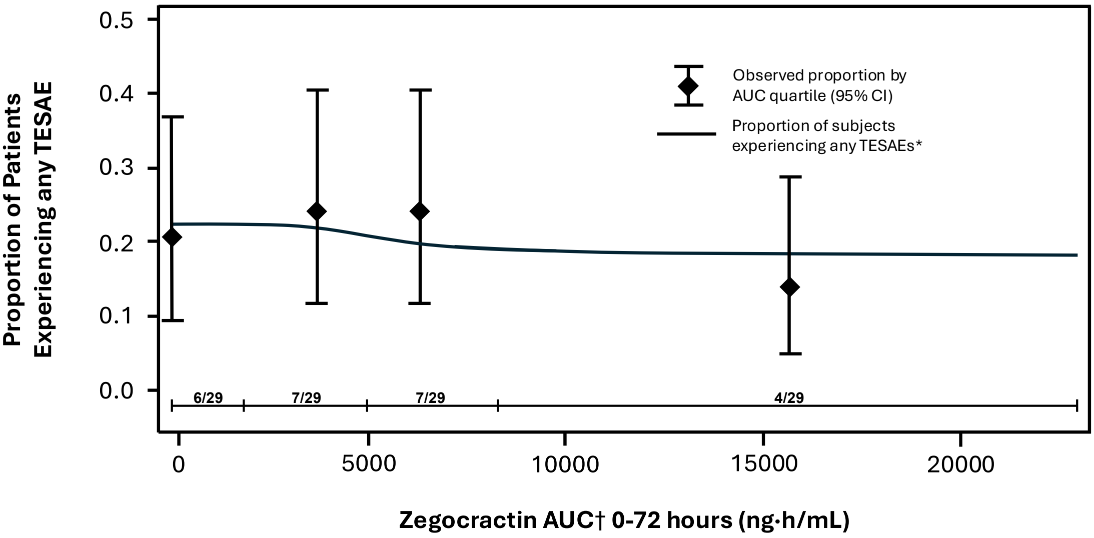

*The abbreviation TESAEs denotes treatment emergent serious adverse events, with the number of patients who developed serious adverse events after the start of trial treatment and from whom pharmacokinetic analysis was undertaken, given in quartiles. †AUC denotes area under the curve. The exposure-response relationship is displayed using Emax logistic regression.

REFERENCES

1. Bretz F, Pinheiro JC, Branson M. Combining multiple comparisons and modeling techniques in dose-response studies. *Biometrics* 2005; **61**(3): 738-48.

2. Pinheiro J, Bornkamp B, Glimm E, Bretz F. Model-based dose finding under model uncertainty using general parametric models. *Stat Med* 2014; **33**(10): 1646-61.

3. EMA. Qualification Opinion of MCP-Mod as an efficient statistical methodology for model-based design and analysis of Phase II dose finding studies under model uncertainty. 2014; https://www.ema.europa.eu/en/documents/regulatory-procedural-guideline/qualification-opinion-mcp-mod-efficient-statistical-methodology-model-based-design-and-analysis-phase-ii-dose-finding-studies-under-model-uncertainty_en.pdf. Retrieved 18 September 2025.

4. FDA Qualification of the MCP-Mod Procedure. 2016; https://www.fda.gov/media/99313/download. Retrieved 18 September 2025.

Table S1 **Modified American Neurogastroenterology and Motility Society Gastroparesis Cardinal Symptom Index Daily Diary (mGCSI-DD)**

	None	Mild	Moderate	Severe	Very Severe
Nausea (feeling sick to your stomach as if you are going to vomit or throw up)	☐	☐	☐	☐	☐
Not able to finish a normal sized meal (for a healthy person)	☐	☐	☐	☐	☐
Feeling excessively full after meals	☐	☐	☐	☐	☐
Abdominal Pain (around or above the navel)	☐	☐	☐	☐	☐
The next question asks you to record the number of times vomiting occurred in the last 24 hours. Please record the number of vomits (throwing up with food or liquid coming out) that occurred in the last 24 hours. Record zero, if you have not vomited during the past 24 hours. If you vomited, write down the number of all vomits. If you vomited once, record one. If you vomited three times during the day, record three. If you vomited three times, whether it was during the same trip to the bathroom or three separate trips, record three as the number of episodes of vomiting.<br><br>During the past 24 hours, how many episodes of vomiting did you have: ____					
In thinking about your pancreatitis, what was the overall severity of your symptoms over the past 24 hours	☐	☐	☐	☐	☐

Table S2 **Number of Trial Infusions Received by Each Patient Group.**

Outcome	Placebo Group (N=53)	Zegocractin, 0.5 mg (N=53)	Zegocractin, 1.0 mg (N=56)	Zegocractin, 2.0 mg (N=54)
No infusion administered (%)	0	1 (1.9)	0	1 (1.9)
Discharged by physician	0	1	0	0
Withdrawal of consent	0	0	0	1
At least 1 infusion (%)*	53 (100)	52 (98.1)	56 (100)	53 (98.1)
All 3 infusions (%)	47 (88.7)	48 (90.6)	42 (75.0)	43 (79.6)
At least 1 but <3 infusions (%)	6 (11.3)	4 (7.5)	14 (25.0)	10 (18.5)
Primary reason for at least 1 but <3 infusions:				
Patient discharged by physician	2	2	6	4
Patient request	0	0	3	3
Withdrawal of consent	1	0	0	0
Adverse event	3	2	1	2
Death	0	0	1	0
Other	0	0	3†	1‡

* Modified intention-to-treat population.
† All three patients left hospital against medical advice.
‡ Attending physician decision to remove one patient from trial.

Table S3 **Time to Solid Food Tolerance in Patients Presenting with Low Haematocrit and with Balthazar A, B, and C on CECT.**

Patient Group	Placebo Group (N=53)	Zegocractin, 0.5 mg (N=52)*	Zegocractin, 1.0 mg (N=56)	Zegocractin, 2.0 mg (N=53)†
Low hematocrit (N=131)	N=33	N=28	N=31	N=29
Hours to solid food tolerance (IQR)	62.0 (36.0, 137.0)	67.0 (19.0, 184.0)	68.0 (28.0, 353.0)	65.0 (25.0, 100.0)
Hazard ratio (95% CI)		0.898 (0.514, 1.570)	0.779 (0.455, 1.331)	1.033 (0.610, 1.751)
Balthazar A, B, C (N=67)	N=18	N=13	N=20	N=16
Hours to solid food tolerance (IQR)	41.5 (17.0, 72.0)	167 (20, 327)	63.5 (25.5, N/A‡)	74.0 (25.5, 118.5)
Hazard ratio (95% CI)		0.726 (0.300, 1.755)	0.954 (0.451, 2.017)	1.040 (0.464, 2.332)

CECT denotes contrast enhanced computer tomography scan, N number of patients, and N/A not applicable.

* One patient in 0.5 mg zegocractin group was without a screening Balthazar score

† One patient in the 2.0 mg zegocractin group did not have a screening hematocrit and one patient was without a screening Balthazar score

‡ Upper limit not applicable as 1.0 mg zegocractin group included patients who did not tolerate solid food throughout the 30-day period of the trial.

Table S4 **Days to discharge of mITT patients.***

Patient Group	Placebo Group (N=53)	Zegocractin, 0.5 mg (N=52)	Zegocractin, 1.0 mg (N=56)	Zegocractin, 2.0 mg (N=53)
Discharged in 1-7 Days (%)	39 (73.6%)	39 (73.6%)	47 (83.9%)	42 (79.2%)
Discharged in 8-14 Days (%)	9 (17.0%)	9 (17.0%)	8 (14.3%)	7 (13.2%)
Discharged in 15-21 Days (%)	2 (3.8%)	2 (3.8%)	0	4 (7.5%)
Discharged in 22-30 Days (%)	3 (5.7%)	3 (5.7%)	1 (1.8%)	0

*mITT denotes modified intention to treat population.

CM4620-203

A Randomized, Double-Blind, Placebo Controlled Dose-Ranging Study of Auxora in Patients with Acute Pancreatitis and Accompanying Systemic Inflammatory Response Syndrome (CARPO)

Sponsor: CalciMedica, Inc.
505 Coast Boulevard South, Suite 307
La Jolla, CA 92037

Sponsor Medical Contact: Sudarshan Hebbar, MD
(816) 838-7105 – Mobile
sudarshan@calcimedica.com – Email

FOR QUALIFIED INVESTIGATORS AND THEIR IRB/EC ONLY

Version: 2.0

Issue Date: December 14, 2020

Confidentiality Statement

The information contained in this document is confidential and proprietary property of CalciMedica, Inc. Any distribution, copying, or disclosure is strictly prohibited unless federal regulations or state law requires such disclosure. Prior written authorization of CalciMedica, Inc. is required for any disclosure or use of the information contained herein. Persons to whom the information is disclosed must know that it is confidential and that it may not be further disclosed by them.

SPONSOR APPROVAL AND SIGNATURE PAGE

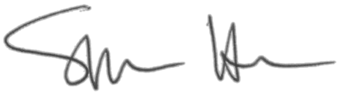

15-Dec-2020

Sudarshan Hebbar MD
Chief Medical Officer

Date

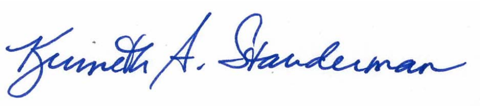

15-Dec-2020

Kenneth A. Stauderman Ph.D.
Chief Scientific Officer

Date

SYNOPSIS

**Protocol Number:**	CM4620-203
**Protocol Title:**	A Randomized, Double-Blind, Placebo Controlled Dose Ranging Study of Auxora in Patients with Acute Pancreatitis and Accompanying Systemic Inflammatory Response Syndrome (CARPO)
**Sponsor:**	CalciMedica, Inc.<br>505 Coast Blvd. South, Suite 307<br>La Jolla, CA 92037 USA
**Study Phase:**	2b
**Number of Patients and Sites:**	Approximately 216 patients with acute pancreatitis and accompanying SIRS will be randomized at approximately 30 sites.
**CM4620-IE Dose and Route of Administration:**	Patients randomized to receive Auxora will receive one of three dose levels, 2.0 mg/kg (1.25 mL/kg), 1.0 mg/kg (0.625 mL/kg), and 0.5 mg/kg (0.3125 mL/kg), every 24 hours (±1 hour) for three consecutive days for a total of three infusions. Auxora will be administered intravenously as a continuous infusion over 4 hours via a bag and tubing compatible with lipid emulsions and using a 1.2-micron filter.
**Placebo Dose and Route of Administration**	Placebo will consist of the emulsion without any active pharmaceutical ingredient CM4620. Patients randomized to receive Placebo will receive one of three dose volumes, 1.25 mL/kg, 0.625 mL/kg, and 0.3125 mL/kg, every 24 hours (±1 hour) for three consecutive days for a total of three infusions. Placebo will be administered intravenously as a continuous infusion over 4 hours via a bag and tubing compatible with lipid emulsions and using a 1.2-micron filter.
**Objectives:**	*Primary*:<br>• To assess the dose response and efficacy of three different dose levels of Auxora in patients with acute pancreatitis and accompanying SIRS;<br>• To assess the time to medically indicated discharge in patients who are responders to early tolerance of solid food intake versus non-responders.<br>*Secondary*:<br>• To assess the safety and tolerability of varying doses of Auxora in patients with acute pancreatitis and accompanying SIRS.

**Inclusion Criteria:**	All of the following must be met for a patient to be randomized into the study:<br>1. The diagnosis of acute pancreatitis has been established by the presence of abdominal pain consistent with acute pancreatitis together with at least 1 of the following 2 criteria:<br>a. Serum lipase > 3 times the upper limit of normal (ULN);<br>b. Characteristic findings of acute pancreatitis on abdominal imaging;<br>2. The diagnosis of SIRS has been established by the presence of at least two of the following four criteria:<br>a. Temperature < 36°C or > 38°C;<br>b. Heart rate > 90 beats/minute;<br>c. Respiratory rate >20 breaths/minute or arterial carbon dioxide tension ($PaCO_2$) <32 mmHg;<br>d. White blood cell count (WBC) >12,000 mm^3, or <4,000 mm^3, or > 10% immature (band) forms;<br>3. At least one of the following criteria is also present:<br>a. A peripancreatic fluid collection or a pleural effusion on a contrast-enhanced computed tomography (CECT) performed in the 12 hours before Consent or after Consent and before Randomization;<br>b. Abdominal examination documenting either abdominal guarding or rebound tenderness;<br>c. Hematocrit ≥44% for men or ≥40% for women;<br>4. The patient is ≥ 18 years of age;<br>5. Lack of pancreatic necrosis, pancreatic calcifications, pancreatic pseudocysts and no evidence for previous necrosectomy or pancreatic surgery identified by CECT performed in the 12 hours before Consent or after Consent and before Randomization;<br>6. A female patient of childbearing potential who is sexually active with a male partner is willing to practice acceptable methods of birth control for 180 days after the last dose of study drug. A female patient must not attempt to become pregnant for 180 days;<br>7. A male patient who is sexually active with a female partner of childbearing potential is willing to practice acceptable methods of birth control for 180 days after the last dose of study drug. A male patient must not donate sperm for 180 days;<br>8. The patient is willing and able to, or has a legal authorized representative (LAR) who is willing and able to, provide informed consent to participate, and to cooperate with all aspects of the protocol.

**Exclusion Criteria:**	Patients with any of the following conditions or characteristics must be excluded from randomizing:<br>1. Expected survival <6 months;<br>2. Suspected presence of cholangitis in the judgment of the treating physician;<br>3. The patient has a known history of:<br>a. Organ or hematologic transplant;<br>b. HIV, hepatitis B, or hepatitis C infection;<br>c. Chronic pancreatitis;<br>4. Current treatment with:<br>a. Chemotherapy;<br>b. Immunosuppressive medications or immunotherapy (Section 5.4 for list of prohibited immunosuppressive medications and immunotherapy);<br>c. Pancreatic enzyme replacement therapy;<br>d. Hemodialysis or Peritoneal Dialysis;<br>5. The patient is known to be pregnant or is nursing;<br>6. The patient has participated in another study of an investigational drug or therapeutic medical device in the 30 days before randomization;<br>7. Allergy to eggs or known hypersensitivity to any components of study drug.
**Study Design:**	This double blind, randomized, placebo-controlled study will evaluate the efficacy, safety, and tolerability of three different dose levels of Auxora in patients with acute pancreatitis and accompanying SIRS.<br><br>Approximately 216 patients will be randomized 1:1:1:1 into one of 4 groups using a computer generated randomization scheme accessed through an interactive voice/web response system (IXRS). Randomization will be first stratified by gender (male or female) and then by risk for organ failure in the gender subgroups (higher or lower). Higher risk for organ failure is defined by the presence of both an elevated hematocrit (HCT ≥44% for men or ≥40% for women) and hypoxemia (imputed $PaO_2/FiO_2 \leq 360$). Lower risk for organ failure is defined by the absence of either or both an elevated hematocrit and hypoxemia (Figure 4). The PaO_2/FiO_2 will be determined using an arterial blood gas or imputed using pulse oximetry (Appendix 5).

	All patients will have received a Screening CECT of the abdomen/pancreas before being randomized into the study. CECTs performed as standard of care may be used as the Screening CECT but must have been performed in the 12 hours before Consent or after Consent and before Randomization.<br><br>The Start of First Infusion of Study Drug (SFISD) should occur within 8 hours of the patient or LAR providing informed consent. Patients randomized to Group 1 will receive 2.0 mg/kg of Auxora intravenously every 24 hours (±1 hour) for a total of three doses. Patients randomized to Group 2 will receive 1.0 mg/kg of Auxora intravenously every 24 hours (±1 hour) for a total of three doses. Patients randomized to Group 3 will receive 0.5 mg/kg of Auxora intravenously every 24 hours (±1 hour) for a total of three doses. Patients randomized to Group 4 will receive emulsion without any active pharmaceutical ingredient. Patients in Group 4 will receive one of three randomly assigned dose volumes, 1.25 mL/kg, 0.625 mL/kg, or 0.3125 mL/kg, which will be administered intravenously every 24 hours (±1 hour) for a total of three doses. The dosing will be based on actual body weight obtained at the time of hospitalization or screening for the study. As described in the pharmacy manual, the upper limit of the volume of Auxora and volume of Placebo that will be administered will be 156.25 mL. The sponsor, investigators and patients will be blinded to the assigned group. In the event of a medical emergency, investigators will be able to receive the treatment assignment if required to provide optimal care of the patient.<br><br>For all 4 groups, a study physician or appropriately trained delegate will perform assessments at screening, at the baseline assessment, immediately prior to the SFISD, and then every 24 hours until 240 hours after the SFISD, or until discharge if earlier. If patients remain hospitalized at Day 12, assessments will then be performed every 48 hours starting on Day 12 until Day 28, or until discharge if earlier. Patients discharged from the hospital before Day 25 will return at Day 30 (+5 days) to perform the Day 30 assessments. If patients are discharged on Days 25-29, the Day 30 assessments may be performed prior to discharge.<br><br>Patients will receive another CECT of the abdomen/pancreas at the Day 30 (±5 days) visit. All CECTs performed as standard of care after randomization and before the Day 30 CECT will also be captured. A blinded central reader will read the Screening, Day 30, and any standard of care CECTs obtained between randomization and 30 days.<br><br>Patients will complete the modified American Neurogastroenterology and Motility Society Gastrointestinal Cardinal Symptom Index Daily Diary (mGCSI-DD) worksheet at the baseline assessment, at 96 hours, 168 hours, Day 14 and Day 21 (for patients who remain hospitalized on these days), on

	the day of discharge, and daily at bedtime after discharge until the Day 30 visit. Patients who are discharged on Days 25-29 will not complete the mGCSI worksheet after discharge.<br><br>It is recommended that all patients randomized in the study should receive care consistent with the 2018 American Gastroenterological Association (AGA) Institute Technical Review of the Initial Medical Management of Acute Pancreatitis. Patients should receive local standard of care (SOC) for the management of other medical conditions.<br><br>In patients with acute pancreatitis, the AGA strongly recommends early oral feeding (within 24 hours) rather than keeping the patient nil per mouth (Nil per Os, NPO). Patients randomized into the study, therefore, will be offered a low fat, ≥500-calorie solid meal at each mealtime after the infusion of the first dose of study drug if alert and not on mechanical ventilation. If the patient does not wish to eat the solid meal when offered or is unable to tolerate the solid meal, they should then be offered a liquid meal. The same approach should occur at each subsequent mealtime. When patients eat a solid meal, it should be recorded if they ate ≥50% of the meal and if they either vomited or experienced an increase in abdominal pain in the two hours after eating a meal.<br><br>It is also recommended that all patients randomized in the study should not be discharged from the hospital until solid food is tolerated, abdominal pain has resolved or been adequately controlled, and there is no clinical evidence of infection. Tolerating solid food is defined as eating ≥50% of a low fat, ≥500-calorie solid meal without an increase in abdominal pain or vomiting. If the patient is not tolerating either solid or liquid meals, tube feedings should be considered.<br><br>All protocol required laboratory testing, except biomarker and PK samples, will be performed at the local laboratory. Results from the biomarkers and PK blood samples collected as part of the protocol and being tested at a central lab will not be available to assist the PI or treating physician in managing the patient.

**Efficacy Assessments:**	Efficacy assessments will include the following:<br>• Enhanced Recovery Strategy for Tolerance of Solid Food<br>• Modified Gastrointestinal Cardinal Symptom Index Score<br>• CECT of the Pancreas<br>• Pain Numeric Rating Scale (PNRS) score<br>• Organ Failure scores<br>• Vital sign measurements<br>• Laboratory measurements<br>• Concomitant medications
**Safety Assessments:**	Safety assessments will include the following:<br>• Vital signs measurements<br>• Laboratory measurements<br>• Concomitant medications<br>• Treatment-emergent adverse events (TEAEs) and serious adverse events (SAEs)
**Efficacy Endpoints:**	**Primary endpoint**<br>• Time to solid food tolerance<br>**Secondary endpoints:**<br>• Solid food tolerance at 48 hours, 72 hours, and 96 hours after the SFISD and at discharge<br>• Time to medically indicated discharge<br>• Length of stay in the hospital<br>• Length of stay in the ICU for patients admitted to the ICU<br>• Re-hospitalization for acute pancreatitis by Day 30<br>• Change in severity of acute pancreatitis by CTSI score from screening to Day 30<br>• Development of pancreatic necrosis ≥30% and >50%<br>• The persistence of SIRS ≥48 hours after the SFISD<br>• Incidence, severity, and duration of organ failure<br>• Mortality by Day 30<br>• Change in pain score and opioid use<br>**Exploratory endpoints:**<br>• Development of infected pancreatic necrosis<br>• Development of sepsis<br>• Hospital procedures for the management of pancreatic necrosis<br>• Change in GCSI-DD score<br>• Change in albumin<br>• Change in ANC/ALC ratio and IL-6 levels<br>• Change in urine NGAL

**Safety Endpoints**	• The incidence of TEAEs and SAEs<br>• The intensity and relationship of TEAEs and SAEs<br>• Clinically significant changes in vital signs and safety laboratory results
**Pharmacokinetic Assessments:**	In patients at selected sites, sparse sampling of plasma will be employed for pharmacokinetic assessment after finishing the first and third doses of study drug.
**Statistical Considerations**	**Sample Size Calculations**<br>A sample size of 216 patients will be randomized into four groups on a 1:1:1:1 basis, resulting in 54 patients randomized into each group. This sample size will provide 86% power for testing the difference in two populations having a median length of time of 70 and 100 hours, respectively, to tolerating solid food. This sample size will provide 80% power with a two-sided alpha of 0.05 to detect a 45% response rate for tolerating solid food in a Auxora dose group versus a 20% response rate in the Placebo group in the 72 hours after completion of the first study drug infusion.<br><br>**Treatment Populations**<br>• Modified Intent to Treat Population will consist of all randomized patients who receive any amount of study drug<br>• The Per-Protocol Population will consist of all randomized patients who complete the study and have no major protocol violations<br>• The PK population will consist of all randomized patients who receive any amount of active study drug and who have at least one post baseline PK sample analyzed<br><br>A full Statistical Analysis Plan (SAP) detailing the primary and secondary efficacy analyses and the safety analyses, will be developed. Data evaluation will include all primary and secondary endpoints, and will be based on descriptive, exploratory, and inferential statistical methods. The number of patients per group, means, standard deviations, medians, quartiles, minimum and maximum values will be determined for continuous variables. Absolute and percentage frequencies will be determined for categorical variables. Handling of missing data and additional sensitivity analyses will be described in the statistical plan.

Table of Contents

Sponsor Approval and Signature Page 2
Synopsis 3
List of Tables 14
List of Figures 14
List of Terms and Abbreviations 15
1 INTRODUCTION 17
1.1 Acute Pancreatitis 17
1.2 Pathophysiology of Acute Pancreatitis 18
1.3 Overview of CM4620 20
1.4 Pre-Clinical Development of CM4620 20
1.4.1 Pre-Clinical Safety and Toxicology Studies 20
1.4.2 Preclinical Efficacy Studies 21
1.5 Clinical Development of Auxora 23
1.5.1 Single Ascending Dose and Multiple Ascending Dose Studies 23
1.5.2 Open Label Study in Patients with AP and SIRS 25
1.5.3 Pharmacodynamic and Pharmacokinetic Study in Patients with AP 28
1.6 Rationale for the Study and Selected Doses 29
2 OBJECTIVES AND ENDPOINTS 31
2.1 Primary Objectives 31
2.2 Secondary Objective 31
2.3 Efficacy Endpoints 31
2.4 Safety Endpoints 31
3 INVESTIGATIONAL PLAN 32
3.1 Study Design 32
3.2 End of Study 34
3.3 Sponsor Termination of the Study 34
4 SELECTION OF PATIENTS 35
4.1 Inclusion Criteria 35
4.2 Exclusion Criteria 36
4.3 Re-Screening 36

5 TREATMENT OF PATIENTS 37
5.1 Overview 37
5.2 Enhanced Recovery Strategy for Tolerance of Solid Food 37
5.3 Discharge Criteria 37
5.4 Prohibited Medications 38
5.5 Compliance 38
6 PROCEDURES 39
6.1 Randomization Procedures 39
6.2 Discontinuation and Withdrawal 39
7 STUDY DRUG MATERIALS AND MANAGEMENT 40
7.1 Auxora Product Description 40
7.2 Placebo Product Description 40
7.3 Auxora and Placebo Storage 40
7.4 Auxora and Placebo Preparation 41
7.5 Auxora and Placebo Administration 41
7.6 Packaging and Labeling 41
7.7 Accountability, Handling and Disposal 42
8 VISITS AND ASSESSMENTS 43
8.1 Screening 43
8.2 Baseline Assessment 44
8.3 Randomization using IXRS system 44
8.4 Start of First Infusion of Study Drug (SFISD) 44
8.5 24 hours 45
8.6 48 hours 45
8.7 72 hours 46
8.8 96, 120, 144, 168, 192, 216, 240 hours (±4 hours) 47
8.9 Days 12 to 28 (±4 hours) 47
8.10 Day 30 (±5 days) 48
8.11 Discharge Date 48
8.12 Study Assessments 49
8.12.1 Demographics, Medical History and Medications 49
8.12.2 Concomitant Medications 49

8.12.3 Abdominal Examination 49
8.12.4 Vital Signs 49
8.12.5 Arterial Blood Gas 49
8.12.6 SpO_2/FiO_2 50
8.12.7 Laboratory Analyses 50
8.12.8 PK analysis 51
8.12.9 Enhanced Recovery Strategy for Tolerance of Solid Food 51
8.12.10 Modified ANMS GCSI-Daily Diary (mGCSI-DD) 52
8.12.11 Pain Numeric Rating Scale (PNRS) 52
8.12.12 Contrast-Enhanced Computed Tomography (CECT) of the Abdomen/Pancreas 52
8.12.13 Systemic Inflammatory Response Syndrome (SIRS) 53
9 ADVERSE EVENTS 54
9.1 Definition of Adverse Event 54
9.2 Definition of Serious Adverse Event 54
9.3 Eliciting Adverse Event Information 55
9.4 Recording Adverse Events 55
9.5 Assessment of Relationship to Study Drug 56
9.6 Assessment of Severity 56
9.7 Reporting of Serious Adverse Events 57
9.8 Suspected Pregnancy in a Woman of Childbearing Potential 58
10 STATISTICAL METHODS 60
10.1 General Considerations 60
10.2 Sample Size 60
10.3 Study Assessments 60
10.4 Analysis Sets 61
10.4.1 Efficacy and Safety 61
10.4.2 Pharmacokinetics 61
10.5 Disposition 61
10.6 Analysis of Demographic and Baseline Data 61
10.7 Efficacy Analysis 62
10.8 Safety Analysis 63

10.9 Independent Data Monitoring Committee ... 63
11 ADMINISTRATIVE CONSIDERATIONS ... 64
11.1 Electronic Case Report Forms ... 64
11.2 Monitoring of the Study ... 64
11.3 Inspection of Records ... 64
11.4 Study Record Retention ... 64
11.5 Study Conduct: Good Clinical Practice and Declaration of Helsinki ... 64
11.6 Responsibilities of the Investigator and the IRB/EC ... 65
11.7 Confidentiality ... 65
11.8 Modification of the Protocol ... 65
11.9 Informed Consent ... 66
11.10 Protocol Violations and Deviations ... 66
11.11 Financial Disclosure ... 67
11.12 Sponsor Obligations ... 67
11.13 Investigator Documentation ... 67
11.14 Clinical Study Insurance ... 67
11.15 Use of Information ... 68
11.16 Publications ... 68
12 REFERENCES ... 69
Appendix 1. Definition of Organ Failure and Sepsis ... 71
Appendix 2. American Gastroenterological Association Institute Guideline on the Initial Management of Acute Pancreatitis ... 73
Appendix 3. CTSI Score ... 74
Appendix 4. Pain Numeric Rating Scale (PNRS) ... 75
Appendix 5. Imputed PaO_2/FiO_2 ... 76
Appendix 6. Modified American Neurogastroenterology and Motility Society Gastrointestinal Cardinal Symptom Index Daily Dairy (mGCSI-DD) ... 77
Appendix 7. Schedule of Events ... 78

List of Tables

Table 1. SAD (CM4620-101) 23
Table 2. MAD (CM4620-102) 24
Table 3. Demographics and Baseline Characteristics of Patients in CM4620-201 26
Table 4. Auxora Product Information 40
Table 5. Conversion of O_2 Flow to FiO_2 50

List of Figures

Figure 1. Schema Showing the Activation of CRAC Channels Found on Pancreatic Acinar Cells Triggering a Series of Events Which Lead to Acute Pancreatitis..... 19
Figure 2. Sites of action of CM4620 in treating acute pancreatitis 22
Figure 3. Stimulated IL-2 production by WBCs in response to administration of Auxora 29
Figure 4. Higher versus Lower Risk of Organ Failure 32

List of Terms and Abbreviations

Abbreviation	Definition
ABG	arterial blood gas
AE	adverse event
AGA	American Gastroenterological Association
ALT	alanine transaminase
ANMS	American Neurogastroenterology and Motility Society
AP	acute pancreatitis
AST	aspartate transaminase
ATP	adenosine triphosphate
Ca^{2+}	Calcium ion
CCK	cholecystokinin
CECT	contrast enhanced computed tomography
CFR	Code of Federal Regulations
CMH	Cochran Mantel-Haenszel
CRAC	calcium release-activated calcium
CRF	case report form
CRP	C-Reactive Protein
CTSI	computed tomography severity index
CV	Cardiovascular
EC	Ethics Committee
ECG	electrocardiogram
EDC	Electronic Data Capture
EDTA	edetate disodium salt dehydrate
ER	endoplasmic reticulum
ERCP	endoscopic retrograde cholangiopancreatography
FAEE	fatty acid ethyl ester
FiO_2	fraction of inspired oxygen
GFR	glomerular filtration rate
GMP	Good Manufacturing Practice of Medicinal Products
Hr(s)	hour(s)
ICH	International Conference on Harmonization
IDMC	Independent Data Monitoring Committee
IP	intraperitoneal
IP_3R	inositol 1,4,5-trisphosphate (IP3) receptor
IRB	Institutional Review Board
IXRS	interactive voice/web response system
Kg	kilogram
L	liter

Abbreviation	Definition
LAR	legal authorized representative
LDH	lactate dehydrogenase
MAD	multiple ascending dose
MedDRA	Medical Dictionary for Regulatory Activities
mGCSI-DD	Modified American Neurogastroenterology and Motility Society Gastrointestinal Cardinal Symptom Index Daily Dairy
MITT	Modified Intent-to-treat Population
MPO	myeloperoxidase
NGAL	Neutrophil gelatinase-associated lipocalin
NOAEL	no observable adverse effect level
NPO	Nil per Os (Nil per Mouth)
OR	odds ratio
Orai1	Calcium release-activated calcium channel protein 1
PaO_2/FiO_2	P/F Ratio
PI	Principal Investigator
PK	pharmacokinetic
PNRS	pain numeric rating scale
popPK	population PK
PP	Per-Protocol Population
PT	preferred Term
QTcF	QT corrected for HR using Fridericia's method
SAD	single ascending dose
SAE	Serious adverse event
SaO_2	arterial blood oxygen saturation measured directly
SAP	Statistical analysis plan
SC	Supportive Care
SFISD	Start of First Infusion of Study Drug
SIRS	Systemic inflammatory response syndrome
SOC	Standard of care
SOC	System Organ Class
SOCE	Store-operated calcium entry
SpO_2	Oxyhemoglobin percent saturation measured by pulse oximetry
STIM1	Stromal interaction molecule 1
TEAE	Treatment-emergent adverse event
ULN	upper limit of normal
VTBI	Volume to be infused
WBC	White blood cell

1 INTRODUCTION

1.1 Acute Pancreatitis

Acute pancreatitis (AP) is an acute inflammatory process of the pancreas with varying involvement of local tissues and/or more remote organ systems. Due to the dynamic nature of the disease, it leads to wide-ranging outcomes that evolve rapidly in any given patient with little predictability. There is no prescribed order of events that the disease course follows, beyond the basic concept of an early (usually <1-2 weeks) and a late (>1-2 weeks) phase, with the former characterised by varying degrees of pancreatic and systemic inflammation, and the latter by an anergic phase that can make patients susceptible to infection.

Due to the evolving nature of AP, the severity may change during the course of the disease. The Atlanta classification of 2012 defines three degrees of severity: mild acute pancreatitis, moderately severe AP, and severe acute pancreatitis (Banks 2013). Terminology that is important in this classification includes transient organ failure, persistent organ failure, and local or systemic complications. Transient organ failure is organ failure that is present for < 48 hours. Persistent organ failure is defined as organ failure that persists for ≥ 48 hours. Local complications include peripancreatic fluid collections and acute necrotic collections, while systemic complications can be related to exacerbations of underlying co-morbidities. A new international classification, referred to as the 'determinant-based classification of severity', has been proposed as an alternative to the Atlanta classification (Dellinger 2012). This classification is based on the actual local and systemic determinants of severity, rather than a description of events that are correlated with severity. The derivation of a classification based on the above principles results in four categories of severity: mild, moderate, severe, and critical. The determinant-based classification approach to severity has elicited a great deal of interest; nevertheless, the Atlanta classification remains the more commonly used standard against which severity of AP is assessed.

AP can be subdivided into two histopathological subtypes: interstitial oedematous pancreatitis and necrotising pancreatitis. In the majority of cases, AP comprises clinically of a mild transitory form of oedematous-interstitial inflammation, which is self-limiting and resolves spontaneously. However, 15–20% of patients with AP will develop the more severe form of the disease (Whitcomb 2006). Necrotising pancreatitis most commonly manifests as necrosis involving both the pancreas and peripancreatic tissues and, less commonly, as necrosis of only the peripancreatic tissue or pancreatic parenchyma alone. Patients with peripancreatic necrosis alone have increased morbidity and intervention rates compared to patients with interstitial oedematous pancreatitis (Singh 2011). Repeated attacks of AP can result in chronic pancreatitis, which increases the risk of developing pancreatic cancer (Yadav 2013).

Gallstones and alcohol misuse are long-established risk factors for AP. The next most common cause is thought to be endoscopic procedures, e.g. endoscopic retrograde cholangiopancreatography (ERCP). Other causes include surgery or trauma, metabolic problems, infections, hereditary factors and drugs. A small residual group has no obvious cause and is labelled ''idiopathic.'' However, many of these may be due to unidentified microlithiasis (cholesterol crystals, biliary

sludge, or small stones). Smoking is an independent risk factor, and its effects could be synergistic with those of alcohol (Lindkvist 2008; Tolstrup 2009).

There are currently two primary aims in the initial treatment of patients with AP. The first aim is to provide supportive therapy, and to treat specific complications that may occur. The second aim is to limit the severity of pancreatic inflammation, necrosis, and SIRS (Werner 2005).

There is currently no approved pharmaceutical treatment for AP, regardless of the severity of the disease (Lankisch 2015). Fluid resuscitation is at the cornerstone of early treatment of AP (Lankisch 2015). Analgesics, usually opioids, are administered initially to manage the abdominal pain. Enteral feeding can also be used and is preferred over exclusive parenteral nutrition. Thus, patients are generally managed with supportive care, including fluid replacement, painkillers, oxygen, feeding via a tube or into a vein, and antibiotics. Severely ill patients with organ failure are managed aggressively using a variety of different treatment strategies.

1.2 Pathophysiology of Acute Pancreatitis

The exocrine pancreas is a highly specialised secretory organ capable of synthesising, storing and releasing large quantities of digestive enzyme precursors into the small intestine, essential for the breakdown of food. Homeostasis of the functional cellular unit, the acinar cell, is therefore paramount for the smooth running of physiological processes; disruption can lead to severe damage of the pancreas, resulting in premature activation of zymogens, vacuolization and necrotic cell death, features typical of AP.

The pancreatic acinar cells are responsible for synthesising digestive proenzymes such as trypsinogen, which, when secreted with pancreatic fluids, become activated in the gut to help digest food. This secretory process is regulated through cytosolic calcium levels in the pancreatic acinar cells. At physiological concentrations, the neurotransmitter acetylcholine and the hormone cholecystokinin (CCK) evoke repetitive short-lasting increases in cytosolic calcium ions (Ca^{2+}) in pancreatic acinar cells, provided by Ca^{2+} release from internal stores such as the endoplasmic reticulum (ER) (Petersen 2006). Repetitive release of Ca^{2+} from internal stores triggers activation of store operated calcium entry (SOCE) through Calcium Release-Activated Calcium (CRAC) channels to replenish the stores from interstitial fluid and activates mitochondria to produce adenosine triphosphate (ATP) (Petersen 2011; Gerasimenko 2013). Both Ca^{2+} and ATP are required for the secretion of the digestive proenzymes and the accompanying fluid needed to wash the secreted proteases out of the duct system and into the gut (Petersen 2011).

The triggers of AP, such as gallstones and alcohol, lead to massive and sustained release of ER Ca^{2+} stores, resulting in maintenance of high cytosolic Ca^{2+} levels and a Ca^{2+} depleted state of the stores (Muallem 1988). The sustained cytosolic Ca^{2+} elevation is due to excessive Ca^{2+} entry into the cells from the interstitial fluid following depletion of ER Ca^{2+} stores. Mechanistically, when the ER stores are depleted of Ca^{2+} the acinar cell attempts to refill the stores with Ca^{2+} entering through a plasma membrane CRAC channel, with Orai1 being the key Ca^{2+}-conducting component of the channel complex (Lur 2009). The opening of the Orai1 channel is activated by STIM1 in response to ER Ca^{2+} store depletion (Gerasimenko 2013).

The massive release of ER Ca^{2+} followed by the sustained elevation in cytosolic Ca^{2+} causes the digestive enzymes to be activated inappropriately within the acinar cells, resulting in autodigestion (i.e., digestion of the pancreas and surrounding tissue), necrosis (Phillip 2014), and the characteristic features of AP. Elevated cytosolic Ca^{2+} also results in lower production of ATP by mitochondria, which limits the cell's ability to handle stress and further promotes necrosis (Criddle 2016). Figure 1 shows the sequence of events leading to AP following activation of CRAC channels.

Figure 1. Schema Showing the Activation of CRAC Channels Found on Pancreatic Acinar Cells Triggering a Series of Events Which Lead to Acute Pancreatitis.

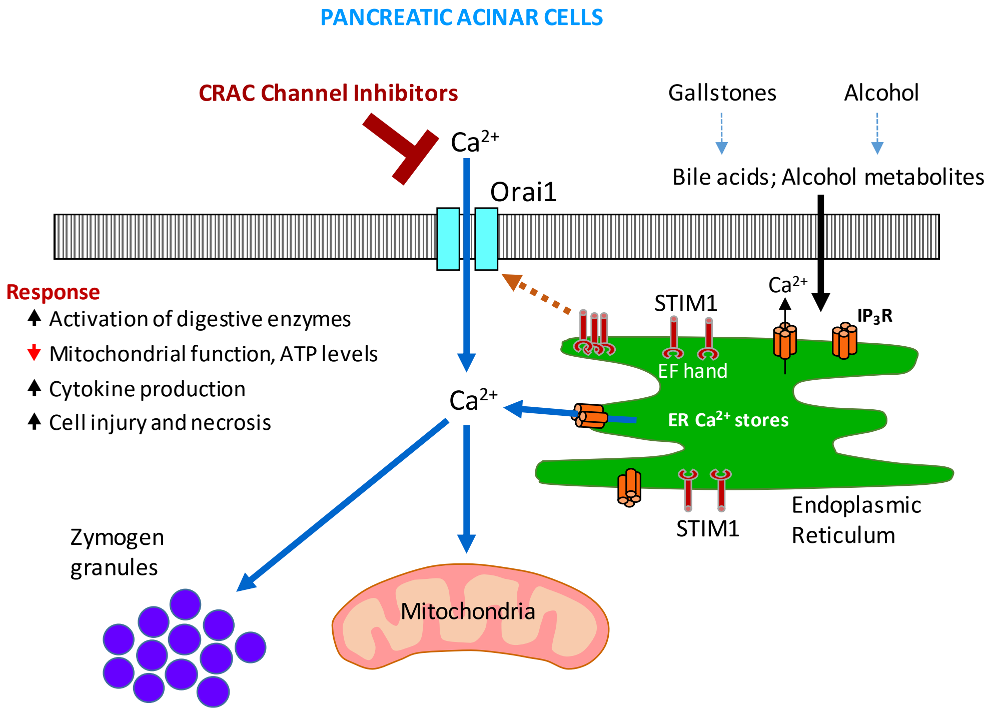

1.3 Overview of CM4620

CM4620 is a potent and selective inhibitor of CRAC channels. CRAC channels are composed of the pore-forming plasma membrane protein Orai1 and the calcium-sensing ER gating-protein STIM1. Low levels of calcium within the ER cause the STIM1 protein to oligomerize and move to locations closely apposed to Orai1. When STIM1 binds to Orai1, the Orai1 Ca^{2+} pore opens, permitting entry of extracellular calcium into the cell through the CRAC channel. This process is referred to as SOCE and evidence suggests that SOCE through CRAC channels plays a critical role in the degradation and necrosis of pancreatic acinar cells in patients with AP.

The potential for CM4620 to inhibit CRAC channels was investigated by measuring the electrophysiological current (I_{CRAC}) associated with calcium entry through CRAC channels in HEK293 cells stably expressing recombinant human Orai1/STIM1 (Wen 2015). Cellular recordings were performed using the whole-cell patch clamp method. Measurements of I_{CRAC} were made after the addition of extracellular 10 mM calcium chloride and subsequent administration of CM4620 at concentrations of 0.001, 0.01, 0.1, and 1 µM. CM4620 was able to inhibit I_{CRAC} in a concentration-dependent manner, producing a mean 50% inhibition (IC_{50}) value of 119 nM. Rapid and complete inhibition was achieved at 1 µM of CM4620. Evaluations were performed to further elucidate the site of action of CM4620. A mutation in Orai1 (Orai1-V102C) is known to produce constitutively active CRAC channels without the need for STIM1. In this evaluation, successive concentrations of CM4620 produced nearly complete inhibition of the STIM1-independent I_{CRAC}, indicating that Orai1 is a major site of action of the compound.

1.4 Pre-Clinical Development of CM4620

1.4.1 Pre-Clinical Safety and Toxicology Studies

Safety pharmacology studies conducted in rats indicated no CM4620-induced adverse effects on central nervous or respiratory systems. Dose-limiting adverse clinical and cardiovascular effects were noted in a single telemetered cynomolgus monkey dosed at 25 mg/kg IV with CM4620-IE. Cardiovascular data at lower doses (1, 3 and 10 mg/kg) showed transient, non-dose-related, slight-to-moderate increases in systolic/diastolic arterial blood pressures and negative chronotropic effects (mild and non-adverse) at all doses and in placebo treated animals.

Repeat-dose toxicity studies conducted in both rats and monkeys showed no observable adverse effect levels (NOAELs) of 25 mg/kg/day and 3 mg/kg/day, respectively. *In vitro* genetic toxicity studies were negative in the Ames bacterial reverse mutation assay and weakly positive/equivocal in a micronucleus assay conducted in human peripheral blood lymphocytes. A subsequent *in vivo* micronucleus study conducted in rats involving two different endpoints (bone marrow micronucleus and liver Comet assays) showed no evidence of DNA reactivity. Based on the results of the complete battery of genotoxicity testing, the weight of evidence indicates that CM4620 is neither mutagenic nor clastogenic. Hemolysis testing concluded that CM4620 placebo was compatible with human plasma and non-hemolytic in human blood. Specific local tolerance studies to examine irritation/inflammation at the injection site were not performed, but no evidence of compound-related or vehicle-related local irritation was observed in the repeat-dose toxicity

studies in rat and monkey. Finally, *in vitro* 3T3 results indicated that CM4620 is potentially phototoxic, so appropriate precautions are being taken in clinical trials.

1.4.2 Preclinical Efficacy Studies

Non-clinical studies conducted in murine and human pancreatic acinar cells to date have demonstrated that by acting on CRAC channels in pancreatic acinar cells, CM4620 can protect these cells from pancreatitis-induced cell death and can reduce biochemical, immunological and histopathologic consequences of experimental AP when administered early in the course of the disease (Wen 2015; Waldron 2019). CM4620 caused marked reductions in serum amylase activity, pancreatic trypsin activity, myeloperoxidase (MPO) activity (a marker of neutrophil infiltration) in the pancreas and lung, and led to a significant reduction in pancreatic histopathology scores. Importantly, the non-clinical findings provide proof of concept that CM4620 has the potential to be effective in the treatment of AP regardless of the cause and support the administration of CM4620 as an IV infusion to humans. Additional non-clinical evidence supports the position that the effects of CM4620 on the pancreas are complimented by its ability to reduce inflammatory responses that can manifest clinically as SIRS.

The protection offered by CM4620 *in vitro* has been confirmed in three diverse *in vivo* models representative of gallstone-, alcohol-, or hyper-stimulation–induced AP (taurolithocholate acid sulphate [TLCS]-induced, fatty acid ethyl ester [FAEE]-induced and cerulein-induced pancreatitis models, respectively). Trypsin activity within pancreatic tissue, myeloperoxidase activity within pancreatic and lung tissue, and histopathological indices of pancreatic damage, such as edema, inflammatory cell infiltration, vacuolization, and necrosis, were all markedly reduced following single 5-20 mg/kg intraperitoneal (IP) doses of CM4620 in the mouse (cerulein-induced AP), two 5 or 20 mg/kg IP doses of CM4620 in the mouse (TLCS-induced and FAEE-induced APs models) or one 4-hour IV infusion of CM4620 Nano-emulsion (the intended clinical dosage form, route of administration and infusion duration) at doses of 5-20 mg/kg in the rat (cerulein-induced AP model) (Wen 2015; Waldron 2019). The timing of CM4620 administration relative to induction of pancreatitis was investigated in the TLCS-induced and FAEE-induced AP models and the results suggest that CM4620 may be more effective in minimizing pancreatic injury and subsequent downstream events if it is administered early in the course of disease, although later administration retains effectiveness in halting disease progression (Wen 2015).

The potential for CM4620 to reduce SIRS through an inhibitory effect on immune cells has been investigated *in vitro*, *ex vivo* and as part of *in vivo* pancreatitis models. *In vitro*, CM4620 inhibited human neutrophil function and inhibited release of key inflammatory cytokines (IL-2, IFNγ and IL-17) from human lymphocytes (IC_{50} 4-138 nM). Results from *ex vivo* pharmacodynamic (PD) analyses in blood demonstrated that CM4620 was able to significantly reduce stimulated IL-2 secretion from T cells, and presumably other lymphocytes, in a dose-dependent manner in Sprague Dawley rats and cynomolgus monkeys after 14 days of oral dosing. Further, the effects of CM4620 on myeloperoxidase activity in the lung of mice in TLCS-induced and FAEE-induced AP models and rats in the caerulein-induced AP model were assessed. Myeloperoxidase activity is a measure of neutrophil infiltration and when seen in the lung in these models it is indicative of a systemic inflammatory process analogous to SIRS in humans. Two IP doses of CM4620 at

20 mg/kg in mice (Wen 2015) or one 4-hour infusion of CM4620 Nano-emulsion at 5 mg/kg in rats (Waldron 2019) were able to substantially reduce lung myeloperoxidase activity (80% reduction in the mouse TLCS model, 73% reduction in the rat caerulein model). Lastly, in the IV infusion study in rats, CM4620 significantly reduced IL-6 and TNFα mRNA levels in both pancreas and lung, as well as that of myeloperoxidase.

In addition to the above, *in vitro* treatment with CM4620 protected mouse, rat and human pancreatic acinar cells from TLCS-induced necrosis or trypsinogen activation, blocked cholecystokinin (CCK)-induced NFAT translocation in isolated mouse acinar cells, CCK-induced NF-κB translocation in a rat acinar cell line, and lipopolysaccharide-induced expression of fibro-inflammatory mRNAs in mouse pancreatic stellate cells Furthermore, a single therapeutic IV infusion of CM4620 Nano-emulsion in rat was shown to reduce markers of ER stress-associated cell death and apoptosis in rat pancreas (Waldron 2019).

Figure 2 highlights the potential role of CM4620 in treating AP through its effects on both the acinar cell and the immune system:

Figure 2. Sites of action of CM4620 in treating acute pancreatitis

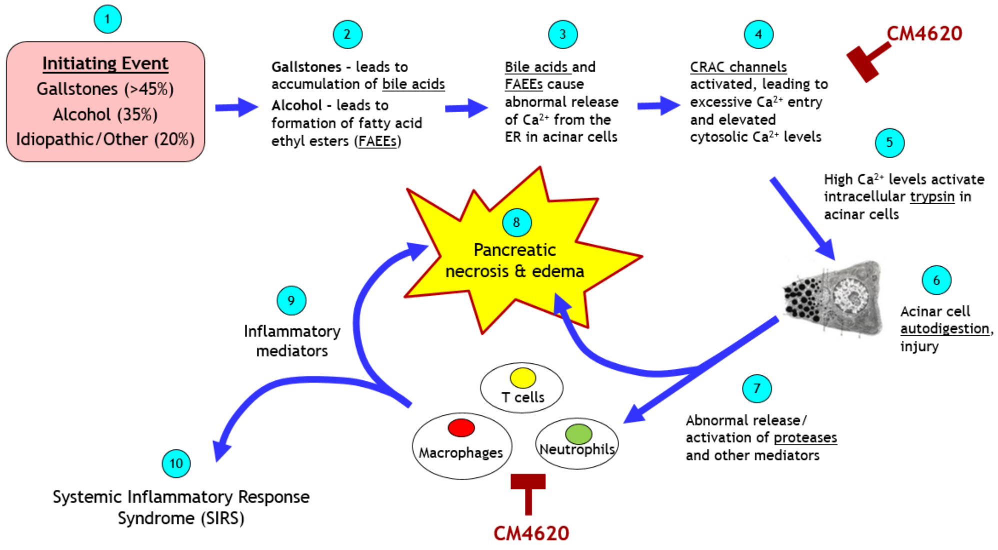

1.5 Clinical Development of Auxora

1.5.1 Single Ascending Dose and Multiple Ascending Dose Studies

CalciMedica has conducted two Phase 1 studies of Auxora in healthy subjects: a single ascending dose (SAD) study (CM4620-101) and a multiple ascending dose (MAD) study (CM4620-102). In CM4620-101 (Table 1), 32 healthy subjects were enrolled in five groups and randomized in a 3:1 ratio to receive a single dose of active versus placebo. The dose levels for each group are noted in Table 1. The dose volume of the emulsion was fixed at 1.3 mL/kg for all subjects in the SAD study groups, and Aurora or placebo was administered via a 4-hour IV infusion.

Table 1. SAD (CM4620-101)

Group	Active Treatment	Number of Active Treatment Subjects	Number of Placebo Treatment Subjects	IV Dose Volume (mL/kg)
1	0.1 mg/kg	6	2	1.3
2	0.24 mg/kg	3	1	1.3
3	0.48 mg/kg	3	1	1.3
4	1.0 mg/kg	6	2	1.3
5	2.1 mg/kg	6	2	1.3

Of the 32 enrolled subjects, there were no serious adverse events (SAE) or adverse events (AE) classified as moderate or severe in intensity. There were three clinical AEs that were all classified as mild in intensity. Two of the AEs were considered possibly related and one was considered unlikely or unrelated to study treatment. In each case, no action was taken with respect to study treatment because of the AEs. No laboratory abnormalities were observed that were considered clinically significant. There were vehicle-related increases in serum triglyceride and cholesterol levels noted in some subjects that returned to baseline within 24 hours. There was no evidence of any sustained treatment related increase in systolic or diastolic blood pressure. In addition, cardiac function, monitored by continuous electrocardiographic recording and serial biomarker testing, showed no evidence of any treatment related effect on heart rate, QTcF, cardiac troponin-T or B-type natriuretic peptide levels.

In the SAD study (CM4620-101), interim non-compartmental pharmacokinetic (PK) analysis indicates that Auxora likely distributes to three compartments. Plasma concentrations of compound rise steadily during the 4-hour infusion, with T_{max} achieved at the end of infusion (4 hours). After the end of infusion, there is a rapid and prominent distribution phase followed by a prolonged period of residual drug levels. The terminal elimination phase has not yet been fully characterized as it appears to be much longer than was anticipated based on pre-clinical PK data in mouse, rat, dog and monkey. Plasma concentrations during the terminal phase are approximately 5% of C_{max} values and, as indicated above, to date there have been no clinically significant AEs reported during this phase. Plasma exposures, defined by AUC_{0-24h}, appear to be dose-proportional

and reached a maximum of 6710 ng*h/mL in Group 5, which is 4.3-fold below the mean AUC_{24h} in monkey at the NOAEL (29,000 ng*hr/mL).

In the MAD study (Table 2) of Auxora (CM4620-102), subjects in the first group were randomized to receive a single dose of active treatment, 0.50 mg/kg, versus placebo for seven consecutive days. Eight healthy subjects were enrolled in the first group, with five receiving active treatment and three receiving placebo. One of the subjects received placebo at the maximum dose volume of emulsion, 1.3 mL/kg, for 7 days, whereas all others were dosed on a weight-based adjustment of dose volume. There were no SAEs and no AEs classified as moderate or severe in intensity. There were 15 clinical AEs that were all classified as mild. In each case, no action was taken with respect to study treatment because of the AEs. No laboratory abnormalities were observed that were considered clinically significant.

Table 2. MAD (CM4620-102)

Group	Active Treatment Daily for 7 days	Number of Active Treatment Subjects	Number of Placebo Treatment Subjects	IV Dose Volume (mL/kg)
1	0.5 mg/kg	5	3	0.3125a
2	1.0 mg/kg	6	2	0.625

[a] one placebo patient received maximum dose volume of 1.3 mL/kg

Subjects in the second group of CM4620-102 were randomized to receive a single dose of active treatment, 1.0 mg/kg, versus placebo for seven consecutive days. Eight healthy subjects were enrolled in the second group, with six receiving active treatment and two receiving placebo for seven consecutive days. There were no SAEs and no AEs classified as moderate or severe in intensity. There were three AEs that were all classified as mild in intensity. In each case, no action was taken with respect to study treatment because of the AEs. No laboratory abnormalities were observed that were considered clinically significant.

There were vehicle-related increases in serum triglyceride noted in some subjects in both groups with levels returning to baseline within 24 hours. Cholesterol levels accumulated in some subjects in both groups with daily dosing but the increases were not considered clinically significant and were related to the vehicle. Thus, the largest rise in cholesterol levels was in the subject who received placebo at the maximum dose volume of emulsion. The rise in cholesterol is believed to be due to the release of tissue cholesterol induced by the lecithin in the emulsion (Byers 1962), was noted in the pre-clinical studies in monkeys, and was reversible with cessation of dosing. There was no evidence of any sustained treatment related increase in systolic or diastolic blood pressure. In addition, cardiac function, monitored by continuous electrocardiographic recording and serial biomarker testing, showed no evidence of any sustained treatment related effect on heart rate, QTcF or B-type natriuretic peptide levels.

Non-compartmental PK analysis of Group 1 in CM4620-102 (0.5 mg/kg) indicates that CM4620 accumulated in plasma, with a 2.6-fold increase in systemic exposure (AUC_{24h}) on Day 7 compared to Day 1 of dosing, consistent with modeling simulations. C_{max} accumulated 1.6-fold (geometric mean of 363 ng/mL on Day 7). The geometric mean of the AUC_{24h} on Day 7 was

3190 ng*hr/mL, which is 9.1-fold below the NOAEL AUC_{24h} in monkey (29,000 ng*hr/mL). PK analysis of Group 2 in CM4620-102 (1.0 mg/kg) indicates that CM4620 accumulated in plasma, with a 2.6-fold increase in AUC_{24h} on Day 7 compared to Day 1 of dosing, consistent with modeling simulations. C_{max} accumulated 1.4-fold (geometric mean 637 ng/mL on Day 7). The geometric mean of the AUC_{24h} on Day 7 was 6830 ng*hr/mL, which is 4.2-fold below the NOAEL AUC_{24h} in monkey (29,000 ng*hr/mL). After the end of 7 days of infusion, there remained a prolonged period of residual drug levels in both MAD groups that remained significantly lower than the C_{max} on Day 7. A 3rd group of healthy subjects in the MAD was not dosed, despite the benign safety profile, because of the prolonged period of residual drug levels noted in the previous groups of healthy subjects.

Subjects in Groups 4 and 5 of the SAD study and Groups 1 and 2 of the MAD study who received CM4620 were followed for 1 year in a long-term extension study to assess for adverse events and serious adverse events. In addition, PK levels were drawn in all 4 groups on Day 270 to further characterize the terminal phase and the prolonged period of minimal residual drug level. There were no serious adverse events and no adverse events rated moderate or severe in intensity in subjects followed for 365 days.

A population PK model was built using the data from the SAD and MAD studies. The model suggested three compartments for distribution as well as gender and body weight-dependent differences in exposures. The model showed that females have a higher volume of distribution compared to males, resulting in lower plasma AUC_{24h} values versus males, and that patients with higher body weights will have a lower AUC. The model was then used to identify the dosing regimens for the first and second phases of the open-label study described below.

1.5.2 Open Label Study in Patients with AP and SIRS

CalciMedica has conducted a Phase 2a, open-label, dose-response, multi-center study of Auxora in patients with AP and accompanying SIRS and hypoxemia (CM4620-201). One patient was randomized having SIRS alone at Screening. The primary objective of the study was to evaluate safety and tolerability; the secondary objective was to evaluate efficacy and the PK profile of CM4620-IE.

The study consisted of 2 phases; the Initial Phase consisted of 2 concurrently enrolled cohorts and the Second Phase consisted of 2 concurrently enrolled cohorts. In total, it was planned to have 4 Cohorts containing 24 adult male and female patients with AP and accompanying SIRS and hypoxemia. In the Initial Phase, 4 female patients were to be randomized in a 3:1 ratio to receive CM4620-IE + Supportive Care (SC) or SC alone (Cohort 1). Concurrently, 4 male patients were to be randomized in a 3:1 ratio to receive CM4620-IE + SC or SC alone (Cohort 2). Doses were to be 1.0 mg/kg on Day1 and 1.4 mg/kg daily on Days 2, 3 and 4 (low dose regimen). In the Second Phase, 8 female patients were to be randomized in a 3:1 ratio to receive CM4620-IE + SC or SC alone (Cohort 3). Concurrently, 8 male patients were to be randomized in a 3:1 ratio to receive CM4620-IE + SC or SC alone (Cohort 4). Planned doses for both Cohorts 3 and 4 were to be 2.08 mg/kg daily on Days 1 and 2 and 1.6 mg/kg daily on Days 3 and 4 (high dose regimen).

The decision to start Cohort 3 in the Second Phase was made after CalciMedica reviewed the available efficacy, safety and tolerability data from Cohort 1 and discussed this with the Principal Investigator (PI). At this point, a decision was made to administer patients in Cohort 3 with the same dose level and schedule as in Cohort 1, as efficacy was observed in Cohort 1. Cohort 3, therefore, received the same dose level and schedule as Cohort 1, 1.0 mg.kg on Day1 and 1.4 mg/kg daily on Days 2, 3 and 4. The decision to start Cohort 4 in the Second Phase of the study was made after CalciMedica reviewed the available efficacy, safety and tolerability data from Cohort 2 and discussed this with the Principal Investigator. Cohort 4, therefore, received the original planned dose level and schedule; 2.08 mg/kg daily on Days 1 and 2 and 1.6 mg/kg daily on Days 3 and 4.

The first infusion of CM4620-IE was started within 6 (up to 8) hours of the patient or LAR providing informed consent and was administered as a continuous IV infusion over 4 hours. Subsequent infusions were to be started every 24 hours (± 1 hour) from the start of the first infusion. In patients receiving CM4620-IE+SC (all doses), there were 9 patients of 14 patients (64%) who did not receive all 4 scheduled doses, 7 of 9 patients because of rapid clinical improvement leading to early discharge and 2 of 9 patients because of study drug discontinuation. Five of 8 patients (63%) receiving the low dose regimen+SC and 4 of 6 patients (67%) receiving the high dose regimen+SC did not receive all 4 doses of CM4620-IE.

The demographic information and baseline characteristics for the patients enrolled in the study are noted in Table 3.

Table 3. Demographics and Baseline Characteristics of Patients in CM4620-201

**Treatment**	**CM4620-IE+SC low dose regimen (N = 8)**	**CM4620-IE+SC high dose regimen (N = 6)**	**CM4620-IE+SC TOTAL (N = 14)**	**SC Alone (N = 7)**
**Median Age (years)**<br>**Min, Max**	55<br>26, 66	43.5<br>37, 55	50.5<br>26, 66	54<br>40, 72
**Gender, n%**	Female 5 (63%)<br>Male 3 (38%)	Female 0<br>Male 6 (100%)	Female 5 (36%)<br>Male 9 (64%	Female 4 (57%)<br>Male 3 (43%)
**Race, n%**	Asian 1 (13%)<br>Black 1 (13%)<br>White 6 (75%)	Asian 0<br>Black 2 (33%)<br>White 4 (67%)	Asian 1 (7%)<br>Black 3 (21%)<br>White 10 (71%)	Asian 0<br>Black 3 (43%)<br>White 4 (57%)
**Median Weight (kg)**<br>**Min, Max**	86<br>56.2, 108.9	92.8<br>84.8, 113.8	87.5<br>56.2, 113.8	93.1<br>59, 108.9
**BMI (kg/m^2)**<br>**Min, Max**	31.6<br>22, 44.4	28.9<br>25, 38.2	30.3<br>22, 44.4	34<br>23.8, 41.6
**Hx Type 2 Diabetes Mellitus**	2 (25%)	1 (17%)	3 (21%)	1 (14%)
**Hx Hypertension**	4 (50%)	4 (67%)	8 (57%)	6 (86%)

The primary objective of this study was to assess the safety and tolerability of CM4620-IE in patients with AP and accompanying SIRS and Hypoxemia. In this study, the low dose regimen+SC and the high dose regimen+SC were well tolerated in patients with AP and SIRS, with no evidence of untoward safety or tolerability findings.

Treatment-emergent AEs (TEAEs) were reported in 7 of 8 patients (88%) receiving the low dose regimen+SC, 5 of 6 patients (83%) receiving the high dose regimen+SC, and 3 of 7 patients (43%) receiving SC alone. Severe TEAEs were reported in 0 of 8 (0%) patients receiving the low dose regimen+SC, 2 of 6 (33%) receiving the high dose regimen+SC, and 2 of 7 (29%) receiving SC alone. There were 3 TEAEs in 2 patients leading to discontinuation of the study drug. Both patients received the high dose regimen+SC.

Two different TEAE preferred terms were reported in 2 or more patients receiving the low dose regimen+SC: Hypokalemia in 2 of 8 patients (25%) and Headache in 2 of 8 patients (25%). Three different TEAE preferred terms were reported in 2 or more patients receiving the high dose regimen+SC: Malnutrition, Confusional State and Acute Respiratory Distress Syndrome were each reported in 2 of 6 patients (33%). There were no TEAE preferred terms reported in 2 or more patients receiving SC alone.

There was 1 TEAE of Chromaturia in a patient receiving the high dose regimen+SC for which the causality was considered Possible. There were no other TEAEs, for which the causality was considered Possible, Probable or Definite.

SAEs were reported in 2 of 8 patients (25%) receiving the low dose regimen+SC, 1 of 6 patients (17%) receiving the high dose regimen+SC and 2 of 7 patients (29%) receiving SC alone. There was 1 death during the study. This patient, who received the high dose regimen+SC, experienced an SAE of Hypoxic-Ischemic Encephalopathy for which the outcome was fatal. The SAE was considered severe and the outcome was designated recovered/resolved with sequelae. Causality was considered to be Unrelated.

There were no untoward changes in vital signs, oxygenation, and laboratory values associated with treatment with either the low dose or high dose regimen of CM4620-IE.

The secondary objective of the study was to evaluate efficacy. Treatment with CM4620-IE (all doses) resulted in more rapid restoration of gut function, less persistent SIRS, a greater percentage of patients with a reduction in the severity of AP by CTSI scoring, and a decreased length of hospital stay compared to SC alone.

At 72 hours or Discharge (if earlier), 7 of 14 (50%) of patients receiving CM4620-IE+SC (all doses) were able to tolerate solid food, defined as eating ≥ 50% of a solid meal without vomiting or an increase in pain, compared to 1 of 7 (14%) patients receiving SC alone. At Day 10 (216 hours or Discharge), 13 of 14 (92.9%) patients receiving CM4620-IE+SC (all doses) were able to tolerate solid food compared to 3 of 7 (43%) patients receiving SC alone. The median (min, max) time to tolerating solid food for patients receiving the low dose regimen+SC was 57 hours (17, 121), for patients receiving the high dose regimen, 78.5 hours (39, 440) and for patients receiving only SC, 100 hours (22, 720).

Five of 14 patients (35.7%) randomized to CM4620-IE+SC (all doses) had persistent SIRS, defined as SIRS lasting continuously for ≥ 48 hours starting on or after the Day 1 (0 hour) assessment, compared to 5 of 7 patients (71.4%) in the SC alone group.

A blinded central reader read all Screening, Day 5 or Discharge, and Unscheduled CECT scans and gave a score for the severity of AP using the CTSI scoring system. One patient treated with the high dose regimen+SC did not receive a CTSI score at either the Screening or Day 5 or Discharge CECTs. One patient treated with SC alone did not receive a CTSI score at Screening because contrast was not given. On the Screening CECT, 8 patients treated with CM4620-IE+SC (all doses) had moderate or severe AP based on the CTSI score. Three of the 8 (37.5%) patients with moderate or severe AP improved with treatment to have mild AP based on the CTSI score on the Day 5 or Discharge CECT. On the Screening CECT, 4 patients treated with SC alone had moderate or severe AP. None of the 4 (0%) patients improved to have mild AP by the Day 5 or Discharge CECT.

The median length of stay (min, max) for patients randomized to CM4620-IE+SC (all doses) and SC alone were 3.70 (1.5, 18.3) and 6.02 (1.1, 30.0) days, respectively.

1.5.3 Pharmacodynamic and Pharmacokinetic Study in Patients with AP

In Study CM4620-202, A Pharmacodynamic and Pharmacokinetic Study of Auxora in Patients with Acute Pancreatitis, patients with AP (regardless of the presence of SIRS and/or hypoxemia) were administered a single IV infusion of 2.08 mg/kg Auxora and blood, plasma and serum were collected for analysis. It was planned to initially enroll 5 patients and then to enroll an additional 4 patients as needed. Ultimately, 7 patients were screened for the study, and all 7 enrolled in and completed the study. On Days 1 and 2, blood and plasma samples for PD and PK analyses, respectively, were obtained 30 minutes after completing the administration of Auxora and 24 hours from the start of the administration of Auxora. In patients hospitalized at Day 5 and 10, blood and plasma samples were obtained; if discharged earlier, samples were obtained at the time of discharge. After discharge, patients returned to the hospital on Day 30 to provide final blood and plasma samples.

Of the 7 patients, 5 (71%) were male and 2 (29%) were female. The median (min, max) age in all 7 patients was 42 (29, 54) years. The age range was 38 to 54 years in males and 29 to 35 years in females. The weight range was 49.4 to 102.1 kg and the BMI range was 19.3 to 32.2. Of the 7 patients, 4 (57%) were black or African and 3 (43%) were white. There were no (0%) Hispanic or Latino patients enrolled in the study. The cause of AP was alcohol in 5 of the 7 patients, hypertriglyceridemia in 1 of the 7, and unknown in the other.

A total of 3 patients experienced 7 TEAEs during the study. One (1) patient experienced a TEAE of Melena and a TEAE of bursitis, 1 patient had a TEAE of Pancreatitis Acute (which was also an SAE), and 1 patient experienced TEAEs of Pneumonia, Alcohol Withdrawal Syndrome, Pyrexia and Respiratory Distress (which was also an SAE). Of the 7 TEAEs, there were 3 mild, 2 moderate and 2 severe TEAEs. The 2 severe TEAEs (Pancreatitis Acute and Respiratory Distress) were also SAEs. The causality of the 7 TEAEs to Auxora was Unrelated for 5 TEAEs and Unlikely for 2 TEAEs.

The results of the study showed a rapid decrease in IL-2 production by stimulated white blood cells with infusion of Auxora with rapid return after discontinuation (Figure 3). The study suggested that there would be no long-term immunomodulatory effects from the administration of Auxora.

Figure 3. Stimulated IL-2 production by WBCs in response to administration of Auxora

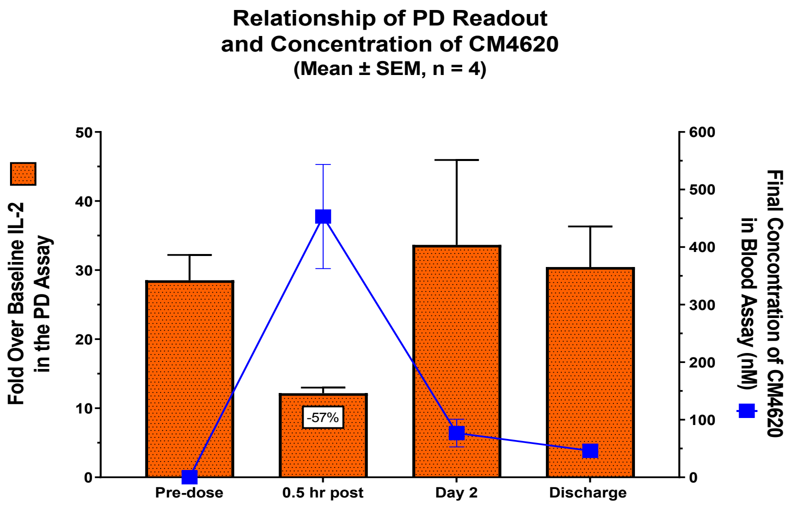

1.6 Rationale for the Study and Selected Doses

Auxora has been well tolerated in healthy volunteers and in patients with AP, SIRS, and hypoxemia with no untoward safety findings. In addition, an array of efficacy signals was noted in the open label study. The safety of the compound and the noted efficacy signals warrant continued development of Auxora for patients with AP given that there are no approved products for the treatment of the disease. A randomized, double blind, placebo controlled, study would establish a clear proof of concept in patients with AP and SIRS, provide dose ranging data to support Phase 3 dosing, and evaluate a number of efficacy endpoints to inform Phase 3 endpoints.

The dosing regimen for the current study includes three consecutive days of dosing at 0.5, 1.0 or 2.0 mg/kg, each administered by 4-hour IV infusion The infusion duration and doses of 1.0 and 2.0 mg/kg were selected based on the results from previous Phase 1 and 2a studies that suggested these doses would have beneficial effects in patients with AP and SIRS. The 0.5 mg/kg dose was included to determine if this dose could define the minimally effective dose of Auxora.

From Phase 2a study CM4620-201, early tolerance of solid food was displayed in patients assigned to both the low and high dose regimens of Auxora (low dose regimen: 1.0 mg/kg on Day 1 and 1.4 mg/kg on Days 2-4; high dose regimen: 2.08 mg/kg on Days 1-2, 1.6 mg/kg on Days 3-4), although not all patients received the full 4 days of dosing because of early discharge. Additionally, no patients (0/6) in the high dose regimen group showed a worsening in their

Balthazar scores, indicative of increased pancreatic inflammation, whereas 2/8 patients (25%) in the low dose regimen group showed an increase in their scores. These data suggest that doses or systemic exposures associated with the high dose regimen provide the greatest opportunity for efficacy with acceptable tolerability. Since 75% of patients receiving the low dose regimen showed no worsening in their Balthazar scores, these doses may also be efficacious, albeit slightly less than those in the high dose regimen. The specific dose levels of 2.0 and 1.0 mg/kg were selected for the current study to closely match the Day 1 doses of the high and low dose regimens in the Phase 2a study, respectively. Doses notably higher than 2.0 mg/kg (1.25 mL/kg) were not selected as they carry increased risk of intolerance to either CM4620 or lipid load from the emulsion vehicle. The third dose level of 0.5 mg/kg was chosen to help define a minimum efficacious dose level. Adjustment of the CM4620 dose for body weight is based on covariate analysis from a population PK (popPK) model that was developed from previous Phase 1 and Phase 2a studies. Dose adjustment is not being made for gender or race based on the absence of substantial covariate effects on the popPK model. The 3 dose levels selected are expected to achieve significant separation of plasma exposures on Day 1 (C_{max} and AUC_{0-24}) based on popPK modeling. Furthermore, 3 dose levels are considered adequate for dose-ranging studies, as described in ICH guidance E4.

The choice of the lower dose level (0.5 mg/kg) is also supported by non-clinical and clinical PK/PD data. Studies in mouse and rat models of AP indicated that achieving a C_{max} blood level of CM4620 *in vivo* close to or greater than the IC_{50} value in an *in vitro* whole blood assay of T lymphocyte function predicted efficacy in the animal model. Consistent with this idea, data from a small PK/PD study in AP patients (CM4620-202) showed that T lymphocyte function in an *ex vivo* whole blood assay was inhibited greater than 50% after a single dose of 2.08 mg/kg CM4620-IE, a dose level linked to signs of efficacy in the open-label study (CM4620-201). Analysis and modeling of clinical PK in patients receiving CM4620-IE indicates that C_{max} values of CM4620 after dosing at 0.5 mg/kg would fall close to the range of the IC_{50} value in an *in vitro* human whole blood assay of T lymphocyte function. Thus, if the animal models are predictive of human disease, the dose of 0.5 mg/kg CM4620-IE should mark the lower limit of the efficacious range.

The dosing regimen for the current study includes 3 consecutive days of dosing at the same dose level. The derivation of this regimen comes from both the Phase 1 MAD and Phase 2a studies, in that the dosing regimens for the Phase 2a open-label study were chosen based on maximum systemic exposures observed in the Phase 1 MAD study that were well tolerated. Thus, the low dose regimen in Phase 2a study CM4620-201 (1.0 mg/kg on Day 1, 1.4 mg/kg on Days 2-4) was designed to achieve plasma exposures on Day 4 (AUC_{0-24}) equivalent to those achieved on Day 7 from the high dose group in the MAD study that received 7 daily doses of 1.0 mg/kg. Over an abundance of caution, the high dose regimen in the Phase 2a study (2.08 mg/kg on Days 1-2, 1.6 mg/kg on Days 3-4) was designed to achieve plasma exposures on Day 4 that were only 30% higher than those achieved with the low dose regimen. Using this approach, no drug-related adverse events were noted in the Phase 2a study. Although well-conceived for safety reasons, these forced titration regimens complicated the interpretation of PK exposures. Therefore, simplified repeat fixed-dose regimens are being utilized in the current study, whereby the same dose level is administered on three consecutive days. Three days of dosing was chosen because it matches the median number of doses administered in the Phase 2a study, where evidence of efficacy signals was established.

2 OBJECTIVES AND ENDPOINTS

2.1 Primary Objectives

- To assess the dose response and efficacy of three different dose levels of Auxora in patients with AP and accompanying SIRS;
- To assess the time to medically indicated discharge in patients who are responders to early tolerance of solid food intake versus non-responders.

2.2 Secondary Objective

- To assess the safety and tolerability of varying doses of Auxora in patients with AP and accompanying SIRS.

2.3 Efficacy Endpoints

- Primary endpoint
  - Time to solid food tolerance
- Secondary endpoints:
  - Solid food tolerance at 48 hours, 72 hours, and 96 hours after the SFISD and at discharge
  - Time to medically indicated discharge
  - Length of stay in the hospital
  - Length of stay in the ICU for patients admitted to the ICU
  - Re-hospitalization for AP by Day 30
  - Change in severity of AP by CTSI score from screening to Day 30
  - Development of pancreatic necrosis ≥30% and >50%
  - The persistence of SIRS ≥48 hours after the SFISD
  - Incidence, severity, and duration of organ failure
  - Mortality by Day 30
  - Change in pain score and opioid use
- Exploratory endpoints:
  - Development of infected pancreatic necrosis
  - Development of sepsis
  - Hospital procedures for the management of pancreatic necrosis
  - Change in GCSI-DD score
  - Change in albumin
  - Change in ANC/ALC ratio and IL-6 levels
  - Change in urine NGAL levels

2.4 Safety Endpoints

- The incidence of TEAEs and SAEs
- The intensity and relationship of TEAEs and SAEs
- Clinically significant changes in vital signs and safety laboratory results

3 INVESTIGATIONAL PLAN

3.1 Study Design

This double blind, randomized, placebo-controlled study will evaluate the efficacy, safety, and tolerability of three different dose levels of Auxora in patients with AP and accompanying SIRS.

Approximately 216 patients will be randomized 1:1:1:1 into one of 4 groups using a computer generated randomization scheme accessed through an interactive voice/web response system (IXRS). Randomization will be first stratified by gender (male or female) and then by risk for organ failure in the gender subgroups (higher or lower). Higher risk for organ failure is defined by the presence of both an elevated hematocrit (HCT ≥44% for men or ≥40% for women) and hypoxemia (imputed $PaO_2/FiO_2 \leq 360$). Lower risk for organ failure is defined by the absence of either or both an elevated hematocrit and hypoxemia (Figure 4). The PaO_2/FiO_2 will be determined using an arterial blood gas or imputed using pulse oximetry (Appendix 5).

Figure 4. Higher versus Lower Risk of Organ Failure

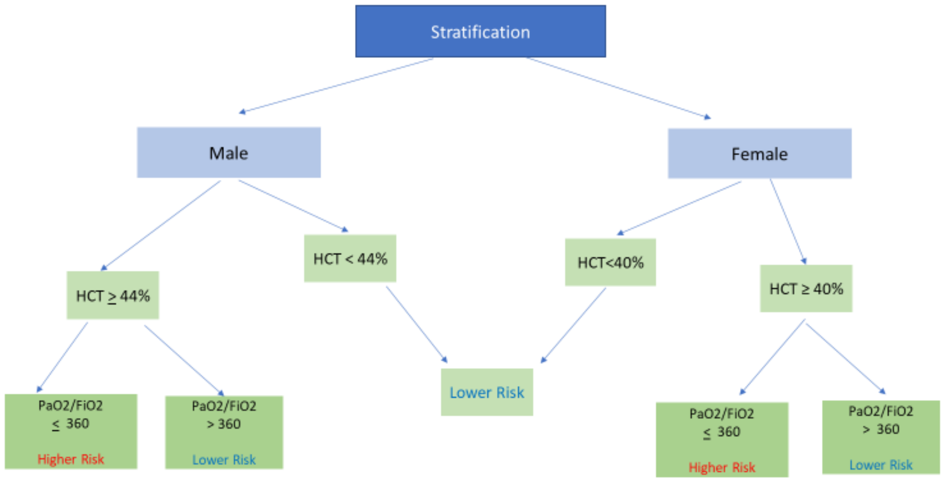

All patients will have received a Screening CECT of the abdomen/pancreas before being randomized into the study. CECTs performed as standard of care may be used as the Screening CECT but must have been performed in the 12 hours before Consent or after Consent and before Randomization.

The Start of First Infusion of Study Drug (SFISD) should occur within 8 hours of the patient or LAR providing informed consent. Patients randomized to Group 1 will receive 2.0 mg/kg of

Auxora intravenously every 24 hours (±1 hour) for a total of three doses. Patients randomized to Group 2 will receive 1.0 mg/kg of Auxora intravenously every 24 hours (±1 hour) for a total of three doses. Patients randomized to Group 3 will receive 0.5 mg/kg of Auxora intravenously every 24 hours (±1 hour) for a total of three doses. Patients randomized to Group 4 will receive emulsion without any active pharmaceutical ingredient. Patients in Group 4 will receive one of three randomly assigned dose volumes, 1.25 mL/kg, 0.625 mL/kg, or 0.3125 mL/kg, which will be administered intravenously every 24 hours (±1 hour) for a total of three doses. The dosing will be based on actual body weight obtained at the time of hospitalization or screening for the study. As described in the pharmacy manual, the upper limit of the volume of Auxora and volume of Placebo that will be administered will be 156.25 mL. The sponsor, investigators and patients will be blinded to the assigned group. In the event of a medical emergency, investigators will be able to receive the treatment assignment if required to provide optimal care of the patient.

For all 4 groups, a study physician or appropriately trained delegate will perform assessments at screening, at the baseline assessment, immediately prior to the SFISD, and then every 24 hours until 240 hours after the SFISD, or until discharge if earlier. If patients remain hospitalized at Day 12, assessments will then be performed every 48 hours starting on Day 12 until Day 28, or until discharge if earlier. Patients discharged from the hospital before Day 25 will return at Day 30 (+5 days) to perform the Day 30 assessments. If patients are discharged on Days 25-29, the Day 30 assessments may be performed prior to discharge.

Patients will receive another CECT of the abdomen/pancreas at the Day 30 (±5 days) visit. All CECTs performed as standard of care after randomization and before the Day 30 CECT will also be captured. A blinded central reader will read the Screening, Day 30, and any standard of care CECTs obtained between randomization and 30 days.

Patients will complete the modified American Neurogastroenterology and Motility Society (ANMS) Gastrointestinal Cardinal Symptom Index Daily Diary (mGCSI-DD) worksheet at the baseline assessment, at 96 hours, 168 hours, Day 14 and Day 21 (for patients who remain hospitalized on these days), on the day of discharge, and daily at bedtime after discharge until the Day 30 visit. Patients who are discharged on Days 25-29 will not complete the mGCSI worksheet after discharge.

It is recommended that all patients randomized in the study should receive care consistent with the 2018 American Gastroenterological Association (AGA) Institute Technical Review of the Initial Medical Management of Acute Pancreatitis. Patients should receive local standard of care (SOC) for the management of other medical conditions.

In patients with acute pancreatitis, the AGA strongly recommends early oral feeding (within 24 hours) rather than keeping the patient nil per mouth (Nil per Os, NPO). Patients randomized into the study, therefore, will be offered a low fat, ≥500-calorie solid meal at each mealtime after the infusion of the first dose of study drug if alert and not on mechanical ventilation. If the patient does not wish to eat the solid meal when offered or is unable to tolerate the solid meal, they should then be offered a liquid meal. The same approach should occur at each subsequent mealtime. When patients eat a solid meal, it should be recorded if they ate ≥50% of the meal and if they either vomited or experienced an increase in abdominal pain in the two hours after the meal.

It is also recommended that all patients randomized in the study should not be discharged from the hospital until solid food is tolerated, abdominal pain has resolved or been adequately controlled, and there is no clinical evidence of infection. Tolerating solid food is defined as eating ≥50% of a low fat, ≥500-calorie solid meal without an increase in abdominal pain or vomiting. If the patient is not tolerating either solid or liquid meals, tube feedings should be considered.

All protocol required laboratory testing, except biomarker and PK samples, will be performed at the local laboratory. Results from the biomarkers and PK blood samples collected as part of the protocol and being tested at a central lab will not be available to assist the PI or treating physician in managing the patient.

CalciMedica may submit amendments to the protocol to modify the planned doses, the dosing schedule, the infusion time, the number of sites in the study, the total number of patients randomized in the study, the number of patients randomized in each group, and the number of groups in the study.

3.2 End of Study

The End of Study is considered the date on which the last patient randomized completes the visit on Day 30, unless CalciMedica terminates the study early.

3.3 Sponsor Termination of the Study

CalciMedica intends to complete the study as outlined. CalciMedica reserves the right, however, to terminate the study at any time because of:

- A recommendation from the IDMC
- A directive from the FDA
- A lack of enrollment

4 SELECTION OF PATIENTS

4.1 Inclusion Criteria

All of the following must be met for a patient to be randomized into the study:

1. The diagnosis of acute pancreatitis has been established by the presence of abdominal pain consistent with acute pancreatitis together with at least 1 of the following 2 criteria:
    a. Serum lipase > 3 times the upper limit of normal (ULN);
    b. Characteristic findings of acute pancreatitis on abdominal imaging;
2. The diagnosis of SIRS has been established by the presence of at least two of the following four criteria:
    a. Temperature < 36°C or > 38°C;
    b. Heart rate > 90 beats/minute;
    c. Respiratory rate >20 breaths/minute or arterial carbon dioxide tension ($PaCO_2$) <32 mmHg;
    d. White blood cell count (WBC) >12,000 mm^3, or <4,000 mm^3, or > 10% immature (band) forms;
3. At least one of the following criteria is present:
    a. A peripancreatic fluid collection or a pleural effusion on a contrast-enhanced computed tomography (CECT) performed in the 12 hours before Consent or after Consent and before Randomization;
    b. Abdominal examination documenting either abdominal guarding or rebound tenderness;
    c. Hematocrit ≥44% for men or ≥40% for women;
4. The patient is ≥ 18 years of age;
5. Lack of pancreatic necrosis, pancreatic calcifications, pancreatic pseudocysts and no evidence for previous necrosectomy or pancreatic surgery identified by CECT performed in the 12 hours before Consent or after Consent and before Randomization;
6. A female patient of childbearing potential who is sexually active with a male partner is willing to practice acceptable methods of birth control for 180 days after the last dose of study drug. A female patient must not attempt to become pregnant for 180 days;
7. A male patient who is sexually active with a female partner of childbearing potential is willing to practice acceptable methods of birth control for 180 days after the last dose of study drug. A male patient must not donate sperm for 180 days;
8. The patient is willing and able to, or has a legal authorized representative (LAR) who is willing and able to, provide informed consent to participate, and to cooperate with all aspects of the protocol.

4.2 Exclusion Criteria

Patients with any of the following conditions or characteristics must be excluded from randomizing:

1. Expected survival <6 months;
2. Suspected presence of cholangitis in the judgment of the treating physician;
3. The patient has a known history of:
   a. Organ or hematologic transplant;
   b. HIV, hepatitis B, or hepatitis C infection;
   c. Chronic pancreatitis;
4. Current treatment with:
   a. Chemotherapy;
   b. Immunosuppressive medications or immunotherapy (Section 5.4 for list of prohibited immunosuppressive medications and immunotherapy);
   c. Pancreatic enzyme replacement therapy;
   d. Hemodialysis or Peritoneal Dialysis;
5. The patient is known to be pregnant or is nursing;
6. The patient has participated in another study of an investigational drug or therapeutic medical device in the 30 days before randomization;
7. Allergy to eggs or known hypersensitivity to any components of study drug.

4.3 Re-Screening

A patient who fails the initial screening because SIRS was not present, or either guarding or rebound were not present, or HCT was not ≥44% for a man or ≥40% for a woman, may be rescreened once again within 6 hours of the original consent. The CECT used to evaluate for inclusion and exclusion criteria must be within 12 hours of the rescreening or must be performed after rescreening and before randomization.

5 TREATMENT OF PATIENTS

5.1 Overview

All patients randomized in the study should receive care consistent with the 2018 American Gastroenterological Association Institute Technical Review of the Initial Medical Management of Acute Pancreatitis and local SOC for the management of other medical conditions. All protocol required laboratory testing, except biomarker and PK samples, will be performed at the local laboratory. Results from the biomarkers and PK blood samples collected as part of the protocol and being tested at a central lab will not be available to assist the PI or treating physician in managing the patient.

All CECTs performed as part of the study from Screening to 30 days after SFISD will be read by a blinded central reader; these scans may also be read locally as necessary and used by the PI or treating physician for patient management.

5.2 Enhanced Recovery Strategy for Tolerance of Solid Food

Poor tolerance of solid food is proportional to severity in patients with AP, identified through an international Delphi process and validated in a study of 439 patients to be second to organ failure in the assessment of AP severity (Buxbaum 2018). Furthermore, as the Phase 2a study CM4620-201 showed a marked improvement in tolerance of solid food in association with CM4620 administration compared to placebo, this parameter will be measured as the primary efficacy endpoint. Patients randomized into the study will be offered a low fat, ≥500-calorie solid meal at each mealtime after the infusion of the first dose of study drug if alert and not on mechanical ventilation. If the patient does not wish to eat the solid meal when offered or is unable to tolerate the solid meal, they should then be offered a liquid meal. The same approach should occur at each subsequent mealtime. When patients eat a solid meal, it should be recorded if they ate ≥50% of the meal and if they either vomited or experienced an increase in abdominal pain in the two hours after the meal. Tolerating solid food is defined by the consumption of ≥50% of a low fat, ≥500-calorie solid meal without an increase in abdominal pain or vomiting in the two hours after the meal. The time when the patient receives a solid meal that is then tolerated shall be recorded. If the patient is not tolerating either solid or liquid meals, tube feedings should be considered as per the AGA Institute Guideline on Initial Management of Acute Pancreatitis.

5.3 Discharge Criteria

The patient randomized to receive study drug should remain in the hospital until all 3 doses of study drug have been administered. The criteria for medically indicated discharge from the hospital are:

- Abdominal pain has resolved or is controlled with medications;
- Solid food is tolerated, which is defined as eating ≥50% of a low fat, ≥500 calorie solid meal without an increase in abdominal pain or vomiting;
- There is no clinical evidence of an infection.

If the patient is ready to be discharged before all 3 doses of study drug have been administered, the PI or treating physician should contact the Medical Monitor prior to discharging the patient.

5.4 Prohibited Medications

Any medication, with the exception of those listed below, may be given at the discretion of the PI. Immunosuppressive medications/immunotherapy that should not be administered during the study include:

- Chemotherapy
- Cyclosporine, Tacrolimus
- Sirolimus, Everolimus
- Azathioprine
- Cyclosphosphamide
- Methotrexate
- Mycophenolate
- Leflunomide
- Biologics/Monoclonals: abatacept, adalimumab, alemtuzumab, anakinra, basilizimab, belimumab, bevacizumab, brodalumab, canakinumab, certolizumab, cetuximab, daclizumab, eculizumab, etanercept, golimumab, infliximab, interferon, ixekizumab, muromonab, natalizumab, omalizumab, rituximab, secukinumab, tocilizumab, trastuzumab, ustekinumab, vedolizumab

5.5 Compliance

Only the PI or his/her appropriately trained study staff will administer study drug to patients randomized in the trial in accordance with the protocol. Study drug must not be used for any reasons other than that described in the protocol.

6 PROCEDURES

6.1 Randomization Procedures

Approximately 216 patients will be randomized 1:1:1:1 into one of 4 groups using a computer generated randomization scheme accessed through an interactive voice/web response system (IXRS). Randomization will be first stratified by gender (male or female) and then by risk for organ failure in the gender subgroups (higher or lower). High risker for organ failure is defined by the presence of both an elevated hematocrit (HCT ≥44% for men or ≥40% for women) and hypoxemia (imputed $PaO_2/FiO_2 \leq 360$). Lower risk for organ failure is defined by the absence of either or both an elevated hematocrit and hypoxemia (Figure 4). The PaO_2/FiO_2 will be determined using an arterial blood gas or imputed using pulse oximetry (Appendix 5).

6.2 Discontinuation and Withdrawal

The term discontinuation refers to a patient or PI discontinuing the administration of study drug before all 3 doses are administered despite the patient remaining in the hospital. Patients who do not receive all 3 doses because the treating physician discharged them from the hospital will not be considered to have discontinued study medication. Patients have the right to discontinue the administration of study drug at any time for any reason, without prejudice to their medical care. The PI may discontinue the administration of study drug because of an adverse event or change in medical status that raises a safety concern about the patient receiving additional doses of study drug. The PI **must** discontinue the administration of study drug if the patient is diagnosed with a new or recurrent malignancy, or if the patient was concomitantly administered a prohibited medication. If possible, the PI should contact the Medical Monitor to review the reasons for a patient's discontinuation from study drug. The PI should also record the reason for the discontinuation in the eCRF and appropriate source documents at the site. Even if the patient discontinues receiving study drug, diligence should occur to ensure that all study visits and assessments are completed.

Withdrawal refers only to the complete withdrawal of the patient from the study because of the withdrawal of consent. The PI should inform the Medical Monitor of the withdrawal of consent and record the withdrawal of consent in the eCRF and appropriate source documents at the site.

7 STUDY DRUG MATERIALS AND MANAGEMENT

7.1 Auxora Product Description

Auxora is to be administered as an IV infusion and is supplied as a translucent, white to yellowish colored, sterile, non-pyrogenic emulsion containing 1.6 mg/mL of the active pharmaceutical ingredient CM4620. CM4620-IE is supplied as an 80 mL fill in a 100 mL, single-use glass vial. The drug product is formulated as an emulsion due to the low solubility of CM4620 in aqueous solution. CM4620-IE contains egg phospholipids, medium chain triglycerides, glycerin, edetate disodium salt dehydrate (EDTA), sodium hydroxide (as needed to adjust pH), and sterile water for injection (Table 4)

Table 4. Auxora Product Information

**Product Name:**	CM4620 Injectable Emulsion
**Dosage Form:**	Injectable Emulsion (Liquid)
**Concentration**	1.6 mg/mL
**Route of Administration**	IV
**Physical Description**	Translucent, non-separated, white to yellowish emulsion
**Inactive Ingredients**	Sterile Water for Injection USP, Egg Phospholipid NF (80% Phosphatidylcholine), Medium Chain Triglycerides NF, Glycerin USP, and Edetate Disodium Salt Dihydrate (EDTA) USP. Sodium Hydroxide and Hydrochloric Acid may be added to adjust the pH.
**Manufacturer**	Bioserv Corporation<br>San Diego, CA 92121

7.2 Placebo Product Description

Matching Placebo is to be administered as an IV infusion and is supplied as a translucent, white to yellowish, sterile, non-pyrogenic emulsion carrier containing no active pharmaceutical ingredient. Placebo is supplied as an 80 mL fill in a 100 mL single-use vial. Placebo contains the same ingredients as Auxora except that it does not contain CM4620.

7.3 Auxora and Placebo Storage

Auxora and Placebo must be maintained in a secure location with refrigerated temperature conditions of 2 to 8°C (36 to 46°F). Precaution should be taken to ensure that the Auxora and Placebo do not freeze. Temperature logs should be maintained and available at each monitoring or remote review visit. When a temperature is noted outside the range of 2°C to 8°C lasting for 24 hours or more, or if the temperature exceeds 20°C (68°F), or is below 0°C (32°F), CalciMedica or its designee must be notified. The stability of Auxora and Placebo has been demonstrated to 24 months and is being evaluated for longer periods in ongoing studies.

7.4 Auxora and Placebo Preparation

The study pharmacist and/or designee will be responsible for the preparation and dispensation of Auxora and Placebo. Prior to administration, Auxora and Placebo must be transferred to a sterile container a using sterile technique. Specific details on how to calculate and prepare Auxora and Placebo, as well as the specific components that will be used to administer Auxora and Placebo, will be provided in a Pharmacy Manual. The Pharmacy Manual will also contain tables detailing the selected dose level, and the concentration of Auxora and Placebo.

7.5 Auxora and Placebo Administration

Both Auxora and Placebo will be administered intravenously over 4 hours at a constant rate of infusion. They will be administered every 24 hours (±1 hours) for three consecutive days for a total of 3 doses. The dose and volume of Auxora, and the volume of Placebo, that will be administered will be calculated using the patient weight obtained at the time of hospitalization or during screening. A line into a peripheral or central vein may be used for the infusion. The peripheral IV should be 20 gauge in size or larger. If a smaller gauge IV is required, please contact the medical monitor. The peripheral IV or central line port should be dedicated when administering Auxora or Placebo other than 0.9% normal saline. Auxora and Placebo are compatible with 0.9% normal saline. The IV tubing used to administer Auxora and Placebo must contain a 1.2 micron filter. The Pharmacy Manual will contain a recommended procedure to prime the IV tubing and flush the tubing, but this may be adapted to local nursing standards. 0.9% normal saline may be used to clear the line to ensure that the volume to be infused (VTBI) is completely administered. If the administration of Auxora or Placebo is stopped because of a technical reason, such as failure of the IV site, or IV pump malfunction, the administration of Auxora or Placebo should be resumed when the technical reason is resolved, and continued at the same rate until the infusion is completed. The total amount of time for the start of infusion to end of infusion of Auxora or Placebo should be recorded.

CalciMedica may modify at any time the administered doses of Auxora or volumes of Placebo, the days of infusion, the timing of the infusion and the rate of infusion based on its review of the safety and tolerability data. CalciMedica will discuss with the PIs before implementation. If the administration of Auxora or Placebo is stopped because of a serious adverse event that is considered to be probably or definitely related to Auxora or Placebo, the Medical Monitor must be immediately contacted.

7.6 Packaging and Labeling

Preparation, packaging and labeling of Auxora or Placebo will be in accordance with current Good Manufacturing Practice of Medicinal Products (GMP) guidelines. Medication labels will comply with legal requirements for labeling of investigational products in the United States.

7.7 Accountability, Handling and Disposal

The PI or designee will ensure that deliveries of Auxora or Placebo from CalciMedica or its designee are received by a responsible person, and such deliveries are recorded; that Auxora or Placebo is handled and stored safely and properly; that Auxora or Placebo is only dispensed to study patients in accordance with the protocol; and that unused Auxora or Placebo is returned to CalciMedica or its designee or disposed of using standard procedures approved of in advance by CalciMedica or its designee. Appropriately trained study staff will administer all doses of Auxora or Placebo. The pharmacy will maintain a record of Auxora or Placebo accountability. The procedure for blinding the administration of study drug, and maintain the blind during the study, will be developed and adapted for all sites.

8 VISITS AND ASSESSMENTS

8.1 Screening

The PI or designee must provide informed consent to the patient, or LAR, allowing the patient or LAR adequate time to consider, ask questions and receive answers, prior to agreeing to participate.

- Record the time of the patient or LAR provides informed consent

After informed consent is obtained, the following procedures are to be performed:

- Record the time of the onset of abdominal pain that sent patient to ER
- Record demographics and medical history
- Record list of medications being taken prior to hospitalization
- Record vital signs closest to the time of informed consent
- Perform an abdominal examination specifically assessing for guarding and rebound tenderness (see Section 8.12.3)
- Draw serum pregnancy test if the patient is a female of childbearing potential – analyze at local lab
  - If already performed as part of standard of care, record results. Testing may be performed on blood drawn in the previous 6 hours prior to Consent
- Draw a blood sample for serum lipase–analyze at local lab
  - If already performed at local lab as part of standard of care, record results. Testing may be performed on blood drawn in the previous 6 hours prior to Consent
- Draw a blood sample for CBC, differential, and platelets–analyze at local lab
  - If already performed at local lab as part of standard of care, record results. Testing may be performed on blood drawn in the previous 6 hours prior to Consent
- Determine if SIRS is present
- Obtain CECT of the abdomen/pancreas and determine if there is evidence of pleural effusion, peripancreatic fluid collection, pancreatic necrosis, pancreatic calcifications, pancreatic pseudocysts, and previous necrosectomy/previous pancreatic surgery are present on a CECT
  - If CECT already performed in the 12 hours prior to the patient or LAR providing informed consent, use results from that CECT

If the patient satisfies all of the inclusion criteria and none of the exclusion criteria, immediately proceed to Baseline Assessment.

8.2 Baseline Assessment

Perform the following prior to randomization (-2 hours):

- Record height and weight
- Record SpO_2 and the FiO_2 closest but prior to the time of randomization
- Record the patient's pain using the PNRS scale closest but prior to the time of randomization
- Record the patient's symptoms using the mGCSI-DD worksheet closest but prior to the time of randomization
- Draw blood samples for Serum Procalcitonin and Serum Chemistries- analyze at local lab.
    - If already performed at local lab as standard of care, record results. Testing may be performed on blood drawn in the previous 6 hours of randomization.
- Draw blood sample for Serum IL-6 –send to central lab. May be performed on blood drawn in the previous 6 hours
- Obtain urine sample for Urine NGAL-send to central lab. May be performed on urine collected in the previous 6 hours

8.3 Randomization using IXRS system

8.4 Start of First Infusion of Study Drug (SFISD)

<u>The SFISD should begin within 8 hours of the patient or LAR providing informed consent.</u>

Perform the following procedures immediately prior to the SFISD:

- Record concomitant medications
- **Infuse the first dose of study drug**
    - Record the time of the SFISD
    - Record the time when the infusion is finished
    - Draw blood sample for PK as soon as possible after completion of the first infusion of study drug (+2 hours)
- <u>Begin Enhanced Recovery Strategy for Tolerance of Solid Food (see Section 5.2) and continue daily until the patient is tolerating solid food.</u>

8.5 24 hours

24 hours from the SFISD:

- Record concomitant medications
- Record vital signs closest to the scheduled time of assessment
- Record the patient's pain using the PNRS closest to the scheduled time of assessment
- Record SpO_2 and the FiO_2 closest to the scheduled time of assessment
- Determine if, and when the patient tolerated solid food in the previous 24 hours (see Section 5.2)
- Perform AE/SAE assessment
- Prior to the infusion of the second dose of study drug:
    - Draw blood samples for CBC, differential, platelets, Serum Procalcitonin and Serum Chemistries- analyze at local lab. Testing may be performed on blood drawn in the previous 12 hours.
    - Draw blood sample for Serum IL-6 –send to central lab. Testing may be performed on blood drawn in the previous 12 hours.
    - Draw blood sample for PK prior to the infusion of the second dose of study drug (-30 min)-send to central lab.
    - Obtain urine sample for Urine NGAL-send to central lab. Testing may be performed on urine collected in the previous 12 hours.
- **Start the infusion of second dose of study drug 24 hours (±1 hour) from the SFISD**
    - Record the time when the infusion starts and finishes

8.6 48 hours

48 hours from the SFISD:

- Record concomitant medications
- Record vital signs closest to the scheduled time of assessment
- Record the patient's pain using the PNRS closest to the scheduled time of assessment
- Record SpO_2 and the FiO_2 closest to the scheduled time of assessment
- Determine if, and when the patient tolerated solid food in the previous 24 hours (see Section 5.2)
- Perform AE/SAE assessment
- Prior to the infusion of the third dose of study drug:

  - Draw blood samples for CBC, differential, platelets, Serum Procalcitonin and and Serum Chemistries – analyze at local lab. Testing may be performed on blood drawn in the previous 12 hours.
  - Draw blood sample for Serum IL-6 –send to central lab. Testing may be performed on blood drawn in the previous 12 hours.
  - Obtain urine sample for Urine NGAL-send to central lab. Testing may be performed on urine collected in the previous 12 hours.
- **Start the infusion of third dose of study drug 48 hours (±1 hour) from the SFISD**
  - Record the time that the infusion starts and finishes
- Assess whether patient discharge is upcoming and whether mGCSI-DD worksheets need to be dispensed per Section 8.12.10.

8.7 72 hours

For patients who remain hospitalized 72 hours from the SFISD:

- Record concomitant medications
- Record weight closest to the scheduled time of assessment
- Record vital signs closest to the scheduled time of assessment
- Record the patient's pain using the PNRS closest to the scheduled time of assessment
- Record SpO_2 and the FiO_2 closest to the scheduled time of assessment
- Determine if, and when the patient tolerated solid food in the previous 24 hours (see Section 5.2)
- Perform AE/SAE assessment
- Draw blood samples for CBC, differential, platelets, Serum Procalcitonin and Serum Chemistries – analyze at local lab. Testing may be performed on blood drawn in the previous 12 hours.
- Draw blood sample for Serum IL-6 –send to central lab. Testing may be performed on blood drawn in the previous 12 hours.
- Draw blood sample for PK ± 2 hours– send to central lab
- Obtain urine sample for Urine NGAL-send to central lab. Testing may be performed on urine collected in the previous 12 hours.
- Assess whether patient discharge is upcoming and whether mGCSI-DD worksheets need to be dispensed per Section 8.12.10.

8.8 96, 120, 144, 168, 192, 216, 240 hours (±4 hours)

For patients who remain hospitalized 96, 120, 144, 168, 192, 216, and 240 hours from the SFISD:

- Record concomitant medications
- Record vital signs closest to the scheduled time of assessment
- Record the patient's pain using the PNRS closest to the scheduled time of assessment
- Record SpO_2 and the FiO_2 closest to the scheduled time of assessment
- Determine if, and when the patient tolerated solid food in the previous 24 hours (see Section 5.2
- Perform AE/SAE assessment
- Assess whether patient discharge is upcoming and whether mGCSI-DD worksheets need to be dispensed per Section 8.12.10.
- If the patient remains hospitalized 96 hours from the SFISD, record the patient's symptoms using the mGCSI-DD worksheet
- If the patient remains hospitalized 168 hours (Day 7) from the SFISD, record the patient's symptoms using the mGCSI-DD worksheet

In addition, for patients who remain hospitalized 120, 168, and 216 hours from the SFISD:

- Draw blood samples for CBC, differential, platelets, Serum Procalcitonin, and Serum Chemistries – analyze at local lab. Testing may be performed on blood drawn in the previous 12 hours.
- Draw blood sample for Serum IL-6 –send to central lab. Testing may be performed on blood drawn in the previous 12 hours.
- Obtain sample for Urine NGAL-send to central lab. Testing may be performed on urine collected in the previous 12 hours.

8.9 Days 12 to 28 (±4 hours)

For patients who remain hospitalized 12, 14, 16, 18, 20, 22, 24, 26, and 28 days from the SFISD:

- Determine if, and when the patient tolerated solid food in the previous 48 hours
- Perform AE/SAE assessment
- Assess whether patient discharge is upcoming and whether mGCSI-DD worksheets need to be dispensed per Section 8.12.10.
- If the patient remains hospitalized at Day 14 and Day 21, request the patient record their symptoms at bedtime using the mGCSI-DD worksheet (see Section 8.12.10)

8.10 Day 30 (±5 days)

Patients who had been discharged from the hospital before Day 25, should return on Day 30 for the following assessments:

- Record the investigator's opinion of etiology of the acute pancreatitis episode
- Record weight
- Perform CECT of the pancreas. May be performed in the 5 days before or after the visit.
- Collect mGCSI-DD worksheets from the patient
- Record concomitant medications
- Record vital signs
- Record the patient's pain using the PNRS
- Record SpO_2 and the FiO_2
- Perform AE/SAE assessment
- Draw blood samples for CBC, differential, platelets, Serum Procalcitonin, and Serum Chemistries – analyze at local lab
- Draw blood sample for Serum IL-6 –send to central lab
- Draw a blood sample for PK -send to central lab
- Obtain sample for Urine NGAL-send to central lab

For patients discharged from the hospital on Days 25-29, the Day 30 assessments may be performed on the Day of discharge.

- Patients who are discharged from the hospital on Days 25-29 will not complete the mGCSI-DD after discharge. They will only complete it on the day of discharge.

For patients who remain hospitalized on Day 30, the following additional assessment will be performed:

- Determine if, and when the patient tolerated solid food in the previous 48 hours

8.11 Discharge Date

The time and date of discharge will be recorded as well as if the patient meets criteria for medically indicated discharge. All patients will be trained to complete the mGCSI-DD and will complete the mGCSI-DD before leaving the hospital. If patients are discharged on Days 25-29, they may complete the Day 30 assessments on the day of discharge.

8.12 Study Assessments

8.12.1 Demographics, Medical History and Medications

To the extent possible given the patient's medical condition, a medical history including family history of pancreatitis and smoking history will be collected at Screening. Demographic data will be collected and will include self-reported race, ethnicity and gender. Patients must be specifically questioned if they have had previous episodes of AP and if they have chronic pancreatitis, diabetes mellitus, hypertension, coronary or cerebrovascular disease, and chronic obstructive pulmonary disease, as well as those conditions noted in the exclusion criteria. The extent of alcohol consumption will be collected. Selected medications that were being taken prior to coming to the emergency department will be collected as will, if available, a baseline serum creatinine.

8.12.2 Concomitant Medications

The name, dose and frequency of selected medications that were administered will be recorded. The time, dose, and route of administration of opioid pain medications that are actually administered will be recorded. Generic names should be used when possible.

8.12.3 Abdominal Examination

An abdominal examination will be performed and recorded at Screening to assess if the patient has abdominal guarding or rebound tenderness. Guarding is the voluntary contraction or involuntary spasm of the abdominal wall musculature to avoid pain from palpation of the abdomen and/or protect underlying inflamed viscera. Rebound tenderness is the elicitation of tenderness by rapidly and smoothly removing the examining hand. In addition, the presence or absence of the following symptoms should also be recorded at screening: bruising in the subcutaneous fatty tissue around the umbilicus and bruising in the flanks.

8.12.4 Vital Signs

The patient's temperature, heart rate (beats per minute), systolic and diastolic blood pressures and respiratory rate (breaths per minute) closest to the time of assessment should be recorded. If intra-abdominal pressures are being monitored, the pressure closest to the time of assessment should be recorded.

8.12.5 Arterial Blood Gas

If an arterial blood gas (ABG) has been performed as supportive care, the time of the draw and following results will be recorded: the pH, PaO_2, $PaCO_2$, SaO_2 and FiO_2.

8.12.6 SpO_2/FiO_2

The SpO_2 will be determined using a hospital approved pulse oximeter. The PI or appropriately trained delegate should review the pulse oximetry waveform determining the SpO_2 to attest to its adequacy. The FiO_2 at the time of the SpO_2 measurement will be recorded. For patients with assisted breathing, the FiO_2 is read from the controlled oxygen source e.g. Venturi masks, ventilator and CPAP/BIPAP systems with calibrated oxygen blenders. For patients breathing unassisted i.e. room air, the FiO_2 is recorded as 0.21. If a patient is on an uncontrolled oxygen source, the following table provides the estimated FiO_2:

Table 5. Conversion of O_2 Flow to FiO_2

Supplemental Oxygen L/min	Estimated FiO_2 (%)		
	Nasal Cannula	Face Mask	Face Mask with Reservoir
Room Air	21		
1	24		
2	28		
3	32		
4	36		
5	40	40	
6	44	50	60
7		50	70
8		60	80
9		60	90
10		60	95

Adapted from Vincent 2009

8.12.7 Laboratory Analyses

At Screening, a lipase, CBC, differential, and platelets, will be performed at the local lab. The results of the CBC will be used to determine the presence of SIRS.

CBC with Differential and Platelets will be performed at 24, 48 and 72 hours, and if the patient remains hospitalized, at 120, 168, and 216 hours following SFISD as well as at Day 30 assessment. These samples will be sent to the local laboratory for analysis.

Serum Chemistries and Serum Procalcitonin will be performed at Baseline, 24, 48 and 72 hours, and if the patient remains hospitalized, at 120, 168, and 216 hours following SFISD as well as at Day 30 assessment. These samples will be sent to the local laboratory for analysis.

CBC results that should be reported in the eCRFs include absolute or percent neutrophil and lymphocyte counts. Serum chemistries reported in CRFs should include sodium, potassium,

chloride, bicarbonate, blood urea nitrogen, creatinine, glucose, calcium, phosphorus, as well as magnesium, total protein, albumin, alkaline phosphatase, alanine transaminase (ALT), aspartate transaminase (AST), total bilirubin, lactate dehydrogenase (LDH), total cholesterol, triglycerides, and creatine kinase (CPK).

If a site is unable to perform any of the laboratory tests because it is not offered at the site or is not part of the standard of care testing, it will not be performed on patients randomized at the site. The only exception to this would be absolute and/or percent neutrophil and lymphocyte counts.

Blood samples for IL-6 and urine samples for urine NGAL will be collected at baseline, 24, 48, and 72 hours, and if the patient remains hospitalized, at 120, 168, 216 hours following SFISD as well as at Day 30 assessment. A reference laboratory will analyze these blood and urine samples IL-6 and urine NGAL. The results of these assays will not be available to the PI or treating physician in managing the patient. It is the expectation that all sites participate in biomarker collection; however, if a site is unable to provide blood or urine samples for the biomarker analysis, this will be determined at study start and not considered a protocol deviation for that site. In addition, if a site is participating in biomarker collection but a patient does not want to provide biomarker samples this will be documented in the informed consent and not considered a protocol deviation.

8.12.8 PK analysis

Blood samples for PK analysis will be drawn as soon as possible after the completion of the first infusion of study drug (+2 hours), prior to the start of second infusion of study drug (-30 min), and at 72 hours (±2 hours) from the SFISD. If the patient is discharged after the third infusion of study drug and prior to 72 hours from the SFISD, the blood sample will be drawn prior to discharge. A PK sample will also be drawn at Day 30. The date and time that the sample for PK analysis is drawn shall be recorded. All samples for PK analysis should be stored at either -20°C or -80°C until shipment on dry ice to the central laboratory. As with the biomarkers, it is the expectation that all sites participate in the PK sample collection; however, if a site is unable to provide blood for the PK analysis, this will be determined at study start and not considered a protocol deviation for that site. In addition, if a site is participating in PK collection but a patient does not want to provide PK samples this will be documented in the informed consent and not considered a protocol deviation.

8.12.9 Enhanced Recovery Strategy for Tolerance of Solid Food

Patients randomized into the study will be offered a low fat, ≥500-calorie solid meal at each mealtime after the infusion of the first dose of study drug if alert and not on mechanical ventilation. See Section 5.2 for specific assessment procedures

8.12.10 Modified ANMS GCSI-Daily Diary (mGCSI-DD)

The ANMS GCSI Daily Dairy was designed to assess gastrointestinal symptoms associated with idiopathic and diabetic gastroparesis. It has been shown to be a reliable tool to evaluate dysmotility in patients with AP (Ma 2016). It will be used to assess gastrointestinal symptoms associated with AP in an exploratory manner. The mGCSI-DD has a 24-hour recall; thus all items are designed to be self-administered on a daily basis.

The patient training for completing the daily diary is provided by the PI or appropriately trained delegate. Patients should understand their disorder: AP, where there is an abnormally delayed emptying of food from the stomach because of inflammation in the pancreas. The mGCSI-DD recording sheet and instructions are reviewed with the patient prior to the patient starting the recording of daily symptoms in the diary. The symptom severity response items are reviewed: "For each symptom listed below, please mark with an X the box that best describes the worst severity of each symptom during the past 24 hours. The next question asks you to record the number of times vomiting occurred in the last 24 hours. Please record the number of vomits (throwing up with food or liquid coming out) that occurred in the last 24 hours. Record zero, if you have not vomited during the past 24 hours. If you vomited, write down the number of all vomits. If you vomited once, record one. If you vomited three times during the day, record three. If you vomited three times, whether it was during the same trip to the bathroom or three separate trips, record three as the number of episodes of vomiting."

Patients discharged before Day 25 will complete the daily diary on the day of discharge, and then after discharge, daily at bedtime until the Day 30 visit.

8.12.11 Pain Numeric Rating Scale (PNRS)

In patients who are able to self-report their pain, the Pain Numeric Rating Scale (Appendix 4) will be used to grade the severity of the abdominal pain. It should be recorded if an opioid analgesic had been given in the 2 hours prior to the PNRS determination.

8.12.12 Contrast-Enhanced Computed Tomography (CECT) of the Abdomen/Pancreas

The recommended CECT protocol will consist of:

- A non-ionic contrast bolus of 100-150cc will be administered at 3-5cc/second
- Slice thicknesses will be 5 mm or less and will be obtained through the pancreas during the pancreatic phase (35 seconds after the start of the non-ionic contrast bolus) and through the entire abdomen during the portal venous phase (50 to 70 seconds after the start of the non-ionic contrast bolus) to visualize the pancreas and surrounding tissue

The lack of perfusion of the pancreas on the CECT will be exclusionary. The CECT protocol may be altered to local standard of care. The PI or treating physician may also elect to delay the Day 30 CECT (±5 days) CECT due to patient-specific safety considerations but must inform the

Medical Monitor of the decision. The PI or treating physician may also elect not to perform the CECT because of futility considerations, e.g., the patient is unlikely to survive in the next 48 hours, but must discuss the decision with the Medical Monitor.

The Screening and Day 30 CECTs will read by a blinded central reader; they will also be read locally and may be used by the PI or treating physician, as needed, for patient management.

If the patient undergoes additional CECTs at the discretion of the PI or treating physician from randomization to Day 30, the date of the CECT will be recorded and a blinded central reader will read the images.

8.12.13 Systemic Inflammatory Response Syndrome (SIRS)

The physician, or appropriately trained designee will determine the presence of SIRS at Screening. The most extreme value for each criterion in the 6 hours before Screening may be used. SIRS is defined as the presence of at least 2 of the 4 criteria:

- Temperature < 36°C or > 38°C
- Heart rate > 90 beats/minute
- Respiratory rate > 20 breaths/minute or arterial carbon dioxide tension (PaCO2) < 32 mmHg
- White blood cell count (WBC) >12,000 mm3, or < 4,000 mm3, or > 10% immature (band) forms

9 ADVERSE EVENTS

9.1 Definition of Adverse Event

An adverse event (AE) is defined as any untoward medical event in a patient regardless of its causal relationship to study treatment. An AE can be any unfavorable and unintended sign (including any clinically significant medical test abnormality), symptom, or disease temporally associated with the use of study drug, whether or not it is considered related to study drug administration. Included in this definition is any newly occurring event or previous condition that has increased in severity or frequency since the administration of study drug.

A medical test abnormality (e.g., laboratory test value, vital sign recording, ECG finding, physical examination finding) will be considered clinically significant and consequently recorded as an AE only if it meets one of the following criteria:

- Induces clinical signs or symptoms
- Requires active intervention
- Requires interruption or discontinuation of study medication

9.2 Definition of Serious Adverse Event

A serious adverse event (SAE) is any AE occurring at any dose and regardless of causality that:

- Results in death
- Is life-threatening
- Requires hospitalization or prolongation of existing hospitalization
- Results in persistent or significant incapacity or substantial disruption of the ability to conduct normal life function
- Is a congenital anomaly or birth defect in an offspring of a patient receiving study drug
- Is an important medical event

The term “life-threatening” refers to an event in which the patient was at immediate risk of death at the time of the event. It does not refer to an event that hypothetically might have caused death if it were more severe.

Important medical events are those that may not need any of the criteria defined above; however, they may be considered serious when, based upon appropriate medical judgment, they may jeopardize the patient and may require medical or surgical intervention to prevent one of the other outcomes listed in the SAE definition.

Pregnancy is not considered an AE; however, information will be collected for any pregnancies that occur during study drug administration or the thirty days thereafter. Certain pregnancy outcomes will require submission as an SAE.

9.3 Eliciting Adverse Event Information

At every AE/SAE assessment, the patient must be asked a standard, non-directed question, such as, “how have you been feeling since your last visit?” to elicit any medically related changes in their well-being. In addition, the hospital chart and other documents relevant to patient safety must be reviewed when the patient is in the hospital.

9.4 Recording Adverse Events

Recording of AEs must begin after randomization. All conditions present before randomization, including untoward medical events during Screening, should be documented as medical history. Documentation shall continue until the patient dies, the patient withdraws consent, or the patient’s participation in the study ends. Information to be collected includes:

- Type of event
- Date of onset
- Date of resolution
- Investigator-specified relationship to study drug and assessment of severity
- Seriousness
- Any action taken

While an AE is ongoing, changes in the severity (e.g., worsening and improving) should be noted in the source documents, but when documenting the AE, only the total duration and the greatest severity should be recorded in the case report form. AEs characterized as intermittent require documentation of onset and duration.

All AEs reported or observed during the study must be followed to resolution. Or, if not fully resolved, until the condition has stabilized, the patient dies, withdraws consent, or CalciMedica ends the trial whichever is first.

Adverse events resulting from concurrent illnesses, reactions to concurrent medications, or progression of disease states must also be reported. Pre-existing conditions (present before the start of the AE collection period) are considered concurrent medical conditions and should NOT be recorded as AEs. However, if the patient experiences a worsening or complication of such a concurrent condition, the worsening or complication should be recorded as an AE. Investigators should ensure that the AE term recorded captures the change in condition (e.g., “worsening of…”).

Each AE should be recorded to represent a single diagnosis. Accompanying signs (including abnormal laboratory test values or ECG findings) or symptoms should NOT be recorded as additional AEs. If a diagnosis is unknown, sign(s) or symptom(s) should be recorded as an AE(s).

Elective procedures (surgeries or therapies) performed to manage/treat conditions that existed prior to the patient enrolling in the trial should not be recorded as AEs but should be documented in the patient’s source documents. If a planned procedure is performed early (e.g., as an emergency) because the pre-existing condition worsens, the worsening condition should be captured as an AE.

9.5 Assessment of Relationship to Study Drug

The Investigator must use the following classification and criteria to characterize the relationship or association of study drug in causing or contributing to the AE:

- **Unrelated**: This relationship suggests that there is no association between study drug and the reported event
- **Unlikely**: This relationship suggests that there is an unlikely association between study drug and the reported event
- **Possible**: This relationship suggests that treatment with study drug caused or contributed to the AE. That is, the event follows a reasonable temporal sequence from the time of study drug administration, and/or follows a known response pattern to the study drug, but could have been produced by other factors
- **Probable**: This relationship suggests that a reasonable temporal sequence of the event with study drug administration exists and, based upon the known pharmacological action of the drug, known or previously reported adverse reactions to the drug or class of drugs, or judgment based on the Investigator's clinical experience, the association of the event with study drug administration seems likely
- **Definite**: This relationship suggests a definite causal relationship exists between study drug administration and the AE, and other conditions (concurrent illness, progression/expression of disease state, or concurrent medication reaction) do not appear to explain the event

9.6 Assessment of Severity

The Investigator must use the following criteria to rate the intensity of the AE:

- **Mild**: Symptoms causing no or minimal interference with usual social and functional activities
- **Moderate**: Symptoms causing greater than minimal interference with usual social and functional activities
- **Severe**: Symptoms causing inability to perform usual social and functional activities

9.7 Reporting of Serious Adverse Events

The Investigator is responsible for reporting to CalciMedica or designee within 24 hours from the time when site personnel learn about the event, all SAEs that are observed or reported by the patient during the study (from randomization until the patient dies, the patient withdraws consent, or the patient's participation in the study ends) regardless of the relationship to study drug or clinical significance. Any additional information that becomes available later should be submitted within 1 working day of receipt. All SAEs reported or observed during the study must be followed to resolution or until the Investigator deems the event to be chronic or the patient to be stable. CalciMedica or its designee may contact the Investigator to obtain additional information on any SAE that has not resolved at the time the patient completes the study. SAEs ongoing at database lock will be noted as such. The PIs are also responsible for informing their IRB/EC of any SAEs at their site. SAE correspondence with IRBs/ECs must be submitted to CalciMedica or its designee for filing.

A study manual will contain the details needed to report SAEs. If any questions on SAEs, contact information is as follows:

- **SAE reporting email address**: CalciMedicaSafety@safety-sphere.com
- **SAE phone number**: 844-965-1070 (toll free)
- **SAE Fax number**: 1 (833) 292-6393

CalciMedica will notify the FDA in a written safety report of any suspected adverse reaction or adverse reaction associated with the use of Auxora that is serious and unexpected.

- Suspected adverse reaction means any adverse event for which there is a reasonable possibility that the drug caused the adverse event. For the purposes of IND safety reporting, 'reasonable possibility' means CalciMedica determines that there is evidence to suggest a causal relationship between the drug and the adverse event (definite, probable, possible) regardless of the investigator's causality assessment
- Adverse reaction means any adverse event caused by a drug. Adverse reactions are a subset of all suspected adverse reactions where there is reason to conclude that the drug caused the event
- Serious, as defined in Section 9.2
- Unexpected adverse event or suspected adverse reaction refers to an event or reaction that is not listed in the investigator's brochure or is not listed at the specificity or severity that has been observed

CalciMedica or its designee will notify the FDA of any unexpected serious suspected adverse reactions associated with the use of Auxora that are fatal or life threatening as soon as possible but no later than seven calendar days after the initial receipt of the information. Initial notification will be followed by a written report within fifteen calendar days.

CalciMedica or its designee will notify the FDA of any unexpected serious suspected adverse reactions associated with the use of Auxora that are not fatal or life threatening fifteen calendar days.

CalciMedica or its designee will provide copies of any reports to regulatory agencies regarding unexpected serious suspected adverse reactions associated with the use of Auxora to the Investigators for review and submission to the IRB/EC.

In addition, the following serious adverse events that are commonly observed in this patient population will not be reported to the regulatory authority as individual expedited reports except in unusual circumstances:

- Hypoxemia and acute respiratory distress syndrome
- Oliguria and acute kidney injury
- Hypotension and shock
- Bacteremia
- Pneumonia
- Obtundation
- Splanchnic venous thrombosis
- Abdominal compartment syndrome
- Gastroparesis and ileus
- Local complications of the pancreas and peripancreatic tissue
- Percutaneous insertion of abdominal drains
- Endoscopic necrosectomy
- Endoscopic retrograde cholangiopancreatography
- Abdominal surgery including but not limited to cholecystectomy, pancreatic necrosectomy, and placement of pancreatic drains

9.8 Suspected Pregnancy in a Woman of Childbearing Potential

A female patient of childbearing potential is a female who is not surgically sterile (no history of a bilateral salpingo-oophorectomy) and is not postmenopausal for at least 1 year.

A female patient of childbearing potential who receives study drug and is sexually active with a male partner, and a male patient who receives study drug and is sexually active with a female of childbearing potential, must be willing to use two highly effective methods of contraception (e.g., barrier methods, spermicidals, intrauterine devices, and/or hormonal contraception) for 180 days after last dose of study drug. No contraception is required if a female patient or partner has undergone a bilateral salpingo-oophorectomy.

Two of the following methods of birth control must be practiced unless a sexually active female patient or partner of childbearing potential has undergone a bilateral salpingo-oophorectomy:

- Male partner has a vasectomy for at least six months duration
- Use of an intrauterine device
- Use of hormonal contraceptives (oral, parenteral, vaginal or transdermal)
- Double barrier contraception with the male partner using a condom and the female using a contraceptive sponge, spermicidal jelly or cream or diaphragm plus spermicidal jelly or cream

The Investigator should be immediately informed if a female patient or partner of childbearing potential suspects she is pregnant up to 180 days after last dose of study drug. If the female patient is receiving study drug when discovered to be pregnant, the study drug should be immediately discontinued. If a pregnancy is confirmed, the Investigator must immediately report a pregnancy and record the event using a Pregnancy Report Form. Pregnancy is not considered an AE but the Investigator must follow a pregnant patient or partner. The Investigator must report follow-up information regarding the course of the pregnancy, including perinatal or neonatal outcome. Infants resulting from such pregnancies should be assessed for normality at birth and should be followed for 6 months to assess for development milestones. CalciMedica or its designee may contact the Investigator to request additional information throughout the course of the pregnancy.

The following pregnancy outcomes must be considered SAEs and will require additional reporting in the eCRF and on an SAE form:

- Congenital anomaly/birth defect
- Stillbirth
- Spontaneous miscarriage

10 STATISTICAL METHODS

10.1 General Considerations

Data summaries and listings will be generated using SAS version 9.3 or a more recent version (SAS Institute Inc., Cary, NC, USA).

The statistical analysis plan and/or the clinical study report will provide additional details of the analysis, which may include details of missing and, if applicable, unused data, as well as additional sensitivity analyses of the primary and secondary variables. The clinical study report will describe deviations from the statistical analysis plan, if any.

The statistical analysis approach will be descriptive, exploratory and inferential to determine the relationship between dose response and endpoints in identifying the optimal dose and primary endpoint(s); also, in identifying the priority order of sequential testing for secondary endpoints.

10.2 Sample Size

A sample size of 216 patients will be randomized into four groups on a 1:1:1:1 basis, resulting in 54 patients randomized into each group. This sample size will provide 86% power for testing the difference in two populations having a median length of time of 70 and 100 hours, respectively, to tolerating solid food. This sample size will provide 80% power with a two-sided alpha of 0.05 to detect a 45% response rate for tolerating solid food in a Auxora dose group versus a 20% response rate in the Placebo group in the 72 hours after completion of the first study drug infusion.

10.3 Study Assessments

Efficacy assessments will include:

- Enhanced Recovery Strategy for Tolerance of Solid Food
- Modified Gastrointestinal Cardinal Symptom Index Score
- CECT of the Pancreas
- Pain Numeric Rating Scale (PNRS) score
- Organ Failure scores
- Vital sign measurements
- Laboratory measurements
- Concomitant medications

Safety assessments will include:

- Vital signs measurements
- Laboratory measurements
- Concomitant medications
- Treatment-emergent adverse events (TEAEs) and serious adverse events (SAEs)

Pharmacokinetic assessments and analyses will include:

- Adding the plasma concentration data to the current population PK model to refine the model and covariate analysis
- Using the revised population PK model to determine if AP disease severity alters PK properties of CM4620
- Determining whether a relationship exists between plasma trough levels of CM4620 and patient response to Auxora (i.e., an exposure-response relationship)

10.4 Analysis Sets

10.4.1 Efficacy and Safety

The efficacy analyses will be performed on the Modified Intent-to-treat Population (MITT) consisting of all randomized patients who receive any amount of study drug.

As the sensitive analysis, the primary efficacy analyses will also be performed on the Per-Protocol Population (PP) consisting of all randomized patients who complete the study and have no major protocol violations.

The safety analyses will be performed on modified intent-to-treat (MITT) data set.

10.4.2 Pharmacokinetics

The PK population will consist of all randomized patients who receive any amount of active study drug and who have at least one post baseline PK sample analyzed.

10.5 Disposition

The number and percentage (n, %) of patients enrolled, screen failed, treated, completed study, and discontinued (with reason) will be summarized. Sample size for each analysis population will be identified for each treatment group. All screened patients will be included in the disposition analysis.

10.6 Analysis of Demographic and Baseline Data

Patient demographic and baseline characteristics will be summarized by mean, standard deviation, median, minimum, and maximum for continuous variables; and by counts and percentages for categorical variables. Summaries will be provided separately for each treatment group.

10.7 Efficacy Analysis

Time to solid food tolerance is the number of hours from the date and time of the SFISD for the patient to the date and time the patient receives a solid meal that is tolerated, which is defined as eating ≥50% of a low fat, ≥500-calorie solid meal without an increase in abdominal pain or vomiting in the two hours after completing the meal. Time to medically indicated discharge is the number of days from the SFISD to the date of meeting discharge criteria in Section 5.3.

Time to solid food tolerance for patients who could not tolerate solid food during the study will be censored on the latest date and time of food tolerance measurement. Time to medically indicated discharge for patients who do not have documented medically indicated discharge per discharge criteria in Section 5.3 during the study will be censored on the earliest date of hospital discharge or study withdrawal or study completion.

Time-to-event endpoints will be summarized using Kaplan-Meier analyses. The medians and quartiles of time-to-events will be summarized. Time to solid food tolerance will be compared between the treatment groups and the placebo group using the stratified log-rank test. The test will be at an alpha level of 0.05. The stratification variables will include gender (male vs. female) and disease severity status (higher risk for organ failure defined by the presence of both a HCT ≥44% for men, or ≥40% for women, and a PaO_2/FiO_2 ≤360 before randomization (Figure 4).

In addition, the hazard ratio (HR) and the 95% CI will be estimated using a Cox proportional-hazard model with treatment group as the independent variable and stratified by the same randomization stratification factors as used for the log-rank test.

Percentage of patients who tolerated solid food at 48, 72 and 96 hours from the SFISD will be summarized and plotted by treatment groups across time points. The solid food tolerance rate at 72 hours will be compared between the treatment groups and the placebo group using a Cochran-Mantel-Haenszel (CMH) test at an alpha level of 0.05 stratified by gender (male vs. female) and disease severity status (higher risk for organ failure defined by the presence of both a HCT ≥44% for men, or ≥40% for women, and a PaO_2/FiO_2 ≤360 before randomization (see Figure 4). The Mantel-Haenszel weighted difference in complete response (CR) rate between the treatment groups and the placebo group and its 2-sided 95% CI will be provided.

In addition, the odds ratio (OR) and the 95% CI will be estimated using a logistic regression model with treatment group as the independent variable and stratified by the same randomization stratification factors as used for the CMH test.

Time to medically indicated discharge will be compared between patients who could tolerate solid food and who could not tolerate solid food at 48, 72, and 96 hours using Kaplan-Meier curve and log-rank test.

Other secondary and exploratory efficacy endpoints will be summarized without profile.

10.8 Safety Analysis

Safety will be assessed by subject reported and Investigator observed AEs along with clinical laboratory tests (hematology and chemistries), and vital signs. Safety variables will be tabulated by treatment groups and presented for all treatment patients. Exposure to study treatments, reasons for discontinuation, deaths and causes of deaths will be tabulated. Treatment-emergent AEs (TEAEs) are defined as events that first occurred or worsened after the first dose of study drug. TEAEs will be mapped to the appropriate System Organ Class (SOC) and Preferred Term (PT) according to the Medical Dictionary for Regulatory Activities (MedDRA).

Summaries will be provided for all AEs, AEs considered related to study treatment, SAEs, and related SAEs as follows:

- By maximum severity
- Incidence by SOC (by severity grade and overall)
- Incidence by PT (by severity grade and overall)

The percentage of 30 day all-cause mortality will be calculated based on each subject's date of death relative to first dose of study treatment and tabulated by treatment groups.

Laboratory test results will be used in the summary. The lower and upper reference range values for blood tests from the laboratories will be used to grade the lab results to low, normal and high. Shift tables will display (1) shift from baseline grade to the worst grade, and (2) shift from baseline grade to the last grade.Vital signs will be summarized by visit using proportion of patients with each vital sign being too high or too low according to conventionally accepted vital sign normal ranges.

Concomitant medication will be coded by the WHO Drug Dictionary and summarized by Therapeutic subgroup and preferred terms, using counts and percentages. Concomitant medications are the medications taken with a start date on or after the start of the administration of study treatment, or those with a start date before the start of the administration of study treatment and a stop date on or after the start of the administration of study treatment.

10.9 Independent Data Monitoring Committee

An IDMC will be convened and will monitor safety for this study on an ongoing basis. An IDMC charter will govern the IDMC and will describe the scope of responsibilities of the IDMC. If the IDMC recommends alteration of the dosing regimen because of safety issues, the FDA and other regulatory agencies will be notified as appropriate. The IDMC responsibilities include protecting the safety of the study patients and making recommendations to CalciMedica concerning the conduct of the study.

11 ADMINISTRATIVE CONSIDERATIONS

11.1 Electronic Case Report Forms

The study will use an Electronic Data Capture (EDC) system that is 21 CFR Part 11 compliant to electronically capture data for all screened and randomized patients.

11.2 Monitoring of the Study

The site monitor, as a representative of CalciMedica, will closely follow the conduct of the study. The site monitor will visit or remotely review the site's study activities periodically and maintain necessary telephone and letter contact with the PI and his/her study staff. The site monitor will maintain current knowledge of each site's study activity by observing the conduct of the study at the site, reviewing study records and source documentation, and discussing the conduct of the study with the PI and his/her study staff.

11.3 Inspection of Records

The PI, his/her study staff and the study site will provide direct access to all study records to assist study-related monitoring and audits, Institutional Review Board/Ethics Committee/Research Ethics Board (IRB/EC) reviews, and regulatory inspections. In the event of an audit, the PI agrees to allow CalciMedica or its representatives and relevant regulatory authorities access to all study records.

If any regulatory agency schedules an audit, the PI should promptly notify CalciMedica or its representatives and promptly forward to CalciMedica copies of any audit reports he/she receives.

11.4 Study Record Retention

The PI or his/her study staff must retain essential documents for at least 2 years after the last approval of a marketing application in an ICH region. They should retain these documents longer if required because of regulatory requirements or because of an agreement with CalciMedica.

11.5 Study Conduct: Good Clinical Practice and Declaration of Helsinki

CalciMedica will design the clinical study, shall implement it, and report it in accordance with the ICH Harmonized Guideline for Good Clinical Practice, with applicable local regulations (e.g., European Directive 2001/83/EC and US Code of Federal Regulations Title 21), and with the ethical principles laid down in the Declaration of Helsinki.

The PI agrees to conduct the study in accordance with the ICH Guideline for Good Clinical Practice, with applicable local regulations (e.g., European Directive 2001/83/EC and US Code of Federal Regulations Title 21) and with the principles of the Declaration of Helsinki. The PI must conduct all aspects of this study in accordance with all national, state and local laws or regulations.

11.6 Responsibilities of the Investigator and the IRB/EC

A properly constituted IRB/EC must review and approve the protocol and the proposed informed consent form before the start of the study at the site. The PI or his/her study staff must provide CalciMedica or its designee a signed and dated statement that the IRB/EC has approved the protocol and the informed consent form before consenting patients for the study. Prior to starting the study, the PI will sign a protocol signature page confirming that he/she will conduct the study in accordance with this protocol and he/she will give CalciMedica or its designee and regulatory authorities access to all relevant data and records.

The IRB/EC chairperson or designee must sign all IRB/EC approvals and must identify the IRB/EC by name and address, the clinical protocol, and the date of approval.

The PI is responsible for obtaining reviews of the clinical research at intervals specified by the IRB/EC. The specified intervals should not exceed 1 year. The PI must supply CalciMedica or its designee written documentation of the reviews of the clinical research.

11.7 Confidentiality

All laboratory specimens, evaluation forms, reports, and other records will be identified in a manner designed to maintain patient confidentiality. All records will be kept in a secure storage area with limited access. Clinical information will not be released without the written permission of the patient (or the patient's legal representative) except as necessary for monitoring and auditing by CalciMedica or its designee, inspections by relevant regulatory authorities, or reviews by the IRB/EC.

The PI, all study staff and all co-workers involved in the study may not disclose or use for any purpose other than the conduct of the study any data, record or other unpublished confidential information disclosed to them for the purpose of the study. They must obtain prior written agreements from CalciMedica or its designee for the disclosure of any said confidential information to other parties.

11.8 Modification of the Protocol

Any changes that arise after the approval of the protocol must be documented as protocol amendments. Amendments (substantial/non-substantial) require regulatory approval and IRB/EC approval or notification. Only after approval by CalciMedica, the PI, the IRB/EC, and if applicable the regulatory authorities, will the protocol amendments become effective. In cases when the protocol is amended to enhance patient safety, the amendment may be implemented but must be immediately submitted to the IRB/EC and regulatory authorities.

The revision number and date of the amendment will be recorded on the title page of the protocol.

The PI is responsible for informing the IRB/EC of all problems involving risks to patients. In case of urgent safety measures, CalciMedica or its designee will immediately notify the PIs and relevant regulatory authorities.

11.9 Informed Consent

Because the study will be conducted under a United States Investigational New Drug Application, informed consent that is in compliance with Title 21 of the United States Code of Federal Regulations (CFR) Part 50 will be obtained from each patient or LAR before the patient enters the study or before any unusual or non-routine procedure is performed. For sites outside the United States, the signed informed consent form will be obtained in compliance with local regulations, ICH E6 (R2) and the principles of the Declaration of Helsinki.

CalciMedica or it designee may provide to the PI or his/her study staff an informed consent form template. The informed consent form must be reviewed by CalciMedica or its designee before the PI or his/her study staff submits it to the IRB/EC. After CalciMedica or its designee review the informed consent form, the PI or his/her study staff will submit it to the IRB/EC for review and approval. If the informed consent form is revised during the course of the study, CalciMedica or its designee must agree with revisions before the PI or his/her study staff submits it to the IRB/EC. The study staff must provide CalciMedica or its designee a copy of the revised informed consent form after IRB/EC approves it. All patients or LARs affected by the revision must sign the revised informed consent form after the IRB/EC approves it.

Before enrolling in the study, each prospective patient or LAR will receive a full explanation of the study and review the approved informed consent form. Once the PI or designee is assured that the patient or LAR understands the implications of participating in the study, he/she will ask the patient or LAR to give consent for the patient to participate in the study by signing the informed consent form.

A patient may participate in the study only after providing consent using an IRB/EC approved informed consent form. A LAR of the patient may provide informed consent on behalf of the patient under conditions authorized by local laws and regulations. The patient or LAR must provide informed consent before the patient undergoes any study-specific procedures described in the protocol. The PI or designee will provide a copy of the informed consent form to the patient and/or LAR. The process of obtaining informed consent must also be documented in the patient source documents.

11.10 Protocol Violations and Deviations

The PI or designee must document any protocol deviation or violation. Reporting of protocol deviations and violations to the appropriate IRB/EC is the responsibility of the PI and must follow the applicable IRB/EC guidelines.

Major protocol deviations are a subset of protocol deviations that might significantly affect the completeness, accuracy, and/or reliability of the study data or that might significantly affect a subject's rights, safety, or well-being.

If there is an immediate hazard to the patient, the PI may deviate from the protocol without prior approval from CalciMedica or its designee and the IRB/EC but must notify CalciMedica or its designee and IRB/EC of the deviation as soon as he/she is able to do so.

11.11 Financial Disclosure

PIs and sub-investigators are required to provide financial disclosure information prior to starting the study. In addition, the PIs and sub-investigators must provide CalciMedica or its designee with updated information if any relevant changes occur during the course of the study and for one year following the completion of the study.

Any PIs, sub-Investigators or study staff with a vested financial interest in the success of the study may not participate in the study.

11.12 Sponsor Obligations

CalciMedica or designee is not financially responsible for further testing/treatment of any medical condition that may be detected during Screening. In addition, CalciMedica is not financially responsible for the treatment of the patient's underlying disease.

11.13 Investigator Documentation

Before beginning the study, the PI will be asked to comply with ICH E6 (R2) 8.2 and title 21 CFR by providing to CalciMedica or designee the following documents:

- The IRB/EC approval of the protocol
- The IRB/EC approved informed consent form
- Any written information regarding the study that will be provided to the patient or LAR
- A Form FDA 1572, fully executed, and all updates on new fully executed Form FDA 1572 (Unless granted an exemption by the FDA and in compliance with local regulations)
- A current Curriculum Vitae for the PI and each sub-Investigator listed on Form FDA 1572. Evidence of licensure must be noted on the Curriculum Vitae or a copy of the license must be provided.
- Completed financial disclosure forms to allow CalciMedica or designee to submit complete and accurate certification or disclosure statements required under US Title 21 CFR 54. In addition, the PI and sub-Investigators must provide to CalciMedica or designee a commitment to update this information promptly if any relevant changes occur during the course of the study and for 1 year following completion of the study
- Laboratory certifications and normal ranges for any laboratories used by the site for the conduct of the study

11.14 Clinical Study Insurance

CalciMedica has subscribed to an insurance policy, covering in its terms and provisions its legal liability for injuries caused to participating persons and arising out of this research that is performed strictly in accordance with the scientific protocol as well as with applicable law and professional standards.

11.15 Use of Information

All information supplied by CalciMedica to the PI and his/her study staff is privileged and confidential. The PI and his/her study staff agree to use this information to accomplish the study and not to use it for other purposes without consent from CalciMedica. Furthermore, the PI and his/her study staff are obligated to provide CalciMedica with complete data obtained during the study. The information obtained from the clinical study will be used towards the development of Auxora, and may be disclosed to regulatory authorities, other Investigators, corporate partners or consultants as required.

11.16 Publications

CalciMedica Inc. reserves the right to review all planned communications and manuscripts based on the results of this study. This reservation of the right is not intended to restrict or hinder publication or any other dissemination of study results, but rather to allow CalciMedica to confirm the accuracy of the data, to protect proprietary information, and to provide comments based on information that may not yet be available to the study investigators. CalciMedica Inc. supports communication and publication of study results whatever the findings of the study. CalciMedica Inc. also encourages disclosure of any conflict of interest from all authors or investigators when manuscripts are submitted for publication.

12 REFERENCES

Balthazar EJ. Acute pancreatitis: assessment of severity with clinical and CT evaluation. Radiology. 2002;223(3):603-13. doi: 10.1148/radiol.2233010680.

Banks PA, Bollen TL, Dervenis C, Gooszen HG, Johnson CD, Sarr MG, Tsiotos GG, Vege SS, Acute Pancreatitis Classification Working G. Classification of acute pancreatitis--2012: revision of the Atlanta classification and definitions by international consensus. Gut. 2013;62(1):102-11. doi: 10.1136/gutjnl-2012-302779.

Buxbaum J, Quezada M, Chong B, Gupta N, Yu CY, Lane C, Da B, Leung K, Shulman I, Pandol S, Wu B. The Pancreatitis Activity Scoring System predicts clinical outcomes in acute pancreatitis: findings from a prospective cohort study. Am J Gastroenterol. 2018;113(5):755-64. doi: 10.1038/s41395-018-0048-1.

Byers SO, Friedman M, Sugiyama T. Mechanism underlying phosphatide-induced hypercholesterolemia. J Biol Chem. 1962;237:3375-80.

Criddle DN. Reactive oxygen species, Ca(2+) stores and acute pancreatitis; a step closer to therapy? Cell Calcium. 2016;60(3):180-9. doi: 10.1016/j.ceca.2016.04.007.

Crockett SD, Wani S, Gardner TB, Falck-Ytter Y, Barkun AN, American Gastroenterological Association Institute Clinical Guidelines C. American Gastroenterological Association Institute Guideline on Initial Management of Acute Pancreatitis. Gastroenterology. 2018;154(4):1096-101. doi: 10.1053/j.gastro.2018.01.032.

Dellinger EP, Forsmark CE, Layer P, Levy P, Maravi-Poma E, Petrov MS, Shimosegawa T, Siriwardena AK, Uomo G, Whitcomb DC, Windsor JA, Pancreatitis Across Nations Clinical R, Education A. Determinant-based classification of acute pancreatitis severity: an international multidisciplinary consultation. Ann Surg. 2012;256(6):875-80. doi: 10.1097/SLA.0b013e318256f778.

Fowler AA, III, Truwit JD, Hite RD, Morris PE, DeWilde C, Priday A, Fisher B, Thacker LR, 2nd, Natarajan R, Brophy DF, Sculthorpe R, Nanchal R, Syed A, Sturgill J, Martin GS, Sevransky J, Kashiouris M, Hamman S, Egan KF, Hastings A, Spencer W, Tench S, Mehkri O, Bindas J, Duggal A, Graf J, Zellner S, Yanny L, McPolin C, Hollrith T, Kramer D, Ojielo C, Damm T, Cassity E, Wieliczko A, Halquist M. Effect of Vitamin C Infusion on Organ Failure and Biomarkers of Inflammation and Vascular Injury in Patients With Sepsis and Severe Acute Respiratory Failure: The CITRIS-ALI Randomized Clinical Trial. JAMA. 2019;322(13):1261-70. doi: 10.1001/jama.2019.11825.

Gerasimenko JV, Gryshchenko O, Ferdek PE, Stapleton E, Hebert TO, Bychkova S, Peng S, Begg M, Gerasimenko OV, Petersen OH. Ca2+ release-activated Ca2+ channel blockade as a potential tool in antipancreatitis therapy. Proc Natl Acad Sci U S A. 2013;110(32):13186-91. doi: 10.1073/pnas.1300910110.

Lankisch PG, Apte M, Banks PA. Acute pancreatitis. Lancet. 2015;386(9988):85-96. doi: 10.1016/S0140-6736(14)60649-8.

Lindkvist B, Appelros S, Manjer J, Berglund G, Borgstrom A. A prospective cohort study of smoking in acute pancreatitis. Pancreatology. 2008;8(1):63-70. doi: 10.1159/000114868.

Lur G, Haynes LP, Prior IA, Gerasimenko OV, Feske S, Petersen OH, Burgoyne RD, Tepikin AV. Ribosome-free terminals of rough ER allow formation of STIM1 puncta and segregation of STIM1 from IP(3) receptors. Curr Biol. 2009;19(19):1648-53. doi: 10.1016/j.cub.2009.07.072.

Ma J, Pendharkar SA, O'Grady G, Windsor JA, Petrov MS. Effect of Nasogastric Tube Feeding vs Nil per Os on Dysmotility in Acute Pancreatitis: Results of a Randomized Controlled Trial. Nutr Clin Pract. 2016;31(1):99-104. doi: 10.1177/0884533615603967.

Muallem S, Schoeffield MS, Fimmel CJ, Pandol SJ. Agonist-sensitive calcium pool in the pancreatic acinar cell. I. Permeability properties. Am J Physiol. 1988;255(2 Pt 1):G221-8. doi: 10.1152/ajpgi.1988.255.2.G221.

Petersen OH, Gerasimenko OV, Gerasimenko JV. Pathobiology of acute pancreatitis: focus on intracellular calcium and calmodulin. F1000 Med Rep. 2011;3:15. doi: 10.3410/M3-15.

Petersen OH, Sutton R. Ca2+ signalling and pancreatitis: effects of alcohol, bile and coffee. Trends Pharmacol Sci. 2006;27(2):113-20. doi: 10.1016/j.tips.2005.12.006.

Phillip V, Steiner JM, Algul H. Early phase of acute pancreatitis: Assessment and management. World J Gastrointest Pathophysiol. 2014;5(3):158-68. doi: 10.4291/wjgp.v5.i3.158.

Singh VK, Bollen TL, Wu BU, Repas K, Maurer R, Yu S, Mortele KJ, Conwell DL, Banks PA. An assessment of the severity of interstitial pancreatitis. Clin Gastroenterol Hepatol. 2011;9(12):1098-103. doi: 10.1016/j.cgh.2011.08.026.

Tolstrup JS, Kristiansen L, Becker U, Gronbaek M. Smoking and risk of acute and chronic pancreatitis among women and men: a population-based cohort study. Arch Intern Med. 2009;169(6):603-9. doi: 10.1001/archinternmed.2008.601.

Vincent JL, Rello J, Marshall J, Silva E, Anzueto A, Martin CD, Moreno R, Lipman J, Gomersall C, Sakr Y, Reinhart K, Investigators EIGo. International study of the prevalence and outcomes of infection in intensive care units. JAMA. 2009;302(21):2323-9.

Waldron RT, Chen Y, Pham H, Go A, Su HY, Hu C, Wen L, Husain SZ, Sugar CA, Roos J, Ramos S, Lugea A, Dunn M, Stauderman K, Pandol SJ. The Orai Ca(2+) channel inhibitor CM4620 targets both parenchymal and immune cells to reduce inflammation in experimental acute pancreatitis. J Physiol. 2019;597(12):3085-105. doi: 10.1113/jp277856.

Wen L, Voronina S, Javed MA, Awais M, Szatmary P, Latawiec D, Chvanov M, Collier D, Huang W, Barrett J, Begg M, Stauderman K, Roos J, Grigoryev S, Ramos S, Rogers E, Whitten J, Velicelebi G, Dunn M, Tepikin AV, Criddle DN, Sutton R. Inhibitors of ORAI1 Prevent Cytosolic Calcium-Associated Injury of Human Pancreatic Acinar Cells and Acute Pancreatitis in 3 Mouse Models. Gastroenterology. 2015;149(2):481-92 e7.

Werner J, Feuerbach S, Uhl W, Buchler MW. Management of acute pancreatitis: from surgery to interventional intensive care. Gut. 2005;54(3):426-36. doi: 10.1136/gut.2003.035907.

Whitcomb DC. Clinical practice. Acute pancreatitis. N Engl J Med. 2006;354(20):2142-50. doi: 10.1056/NEJMcp054958.

Yadav D, Lowenfels AB. The epidemiology of pancreatitis and pancreatic cancer. Gastroenterology. 2013;144(6):1252-61. doi: 10.1053/j.gastro.2013.01.068.

Appendix 1. Definition of Organ Failure and Sepsis

The definition of organ failure encompasses three organ systems, respiratory system, renal system and cardiovascular system.

For the respiratory system, organ failure will be defined as:

- $PaO_2/FiO_2 \leq 300$ determined by arterial blood gas or imputed using pulse oximetry, or
- The use of noninvasive or invasive mechanical ventilation

For the renal system, in patients without known chronic kidney disease (baseline eGFR ≥60 mL/min/1.73 m^2), organ failure will be defined as:

- An increase in serum creatinine ≥300% if the baseline creatinine is known from prior to the hospitalization for AP, or
- A serum creatinine ≥1.9 mg/dL, or
- The initiation of renal replacement therapy

For patients with pre-existing chronic kidney disease (baseline GFR <60 mL/min/1.73 m^2), organ failure will be defined as:

- An increase in serum creatinine ≥300% if the baseline creatinine is known from prior to the hospitalization for AP, or
- An increase of serum creatinine to a level >4.0 mg/dl, or
- The initiation of renal replacement therapy

For the cardiovascular system, organ failure will be defined as:

- A systolic blood pressure <90 mmHg that is not fluid responsive, or
- The use of either vasopressor or inotropic support

Sepsis is the life threatening organ dysfunction caused by a dysregulated host response to infection. For this study, organ dysfunction in the setting of infection will be defined by the presence of one of the following criteria:

- $PaO_2/FiO_2 \leq 300$ determined by arterial blood gas or imputed using pulse oximetry
- The use of noninvasive or invasive mechanical ventilation
- Platelet count <100 x10^3/uL
- Total bilirubin ≥2.0 mg/dL
- The use of vasopressor or inotropic support
- Serum creatinine ≥1.9 mg/dL in patients without pre-existing chronic kidney disease
- Serum creatinine >4.0 mg/dL in patients with pre-existing chronic kidney disease

- An increase in serum creatinine ≥300% if the baseline creatinine is known from prior to the hospitalization for acute pancreatitis
- The initiation of renal replacement therapy

Modified Marshall Scoring System: Organ Dysfunction in Acute Pancreatitis

		**0 (None)**	**1 (None)**	**2 (Mild)**	**3 (Moderate)**	**4 (Severe)**
Respiratory	PaO_2/FiO_2	>400	301-400	201-300	101-200	≤100
Renal	Serum Cr mg/dL	<1.4	1.4-1.8	1.9-3.6	3.7-4.9	>4.9
CV no inotrope	SBP mmHg	≥90	<90 fluid responsive	<90 not fluid responsive	<90, pH <7.3	<90, pH <7.2

Adapted from Banks 2013

Appendix 2. American Gastroenterological Association Institute Guideline on the Initial Management of Acute Pancreatitis

	Recommendation	Strength	Quality
1a.	In patients with AP, the AGA suggests using goal-directed therapy for fluid management.<br>*Comment: The AGA makes no recommendation whether normal saline or Ringer's lactate is used.*	Conditional	Very Low
1b.	In patients with AP, the AGA suggests against the use of HES fluids	Conditional	Very Low
2	In patients with predicted severe AP and necrotizing AP, the AGA suggests against the use of prophylactic antibiotics	Conditional	Low
3	In patients with acute biliary pancreatitis and no cholangitis, the AGA suggests against the routine use of urgent ERCP	Conditional	Low
4	In patients with AP, the AGA recommends early (within 24 h) oral feeding as tolerated, rather than keeping the patient nil per os	Strong	Moderate
5	In patients with AP and inability to feed orally, the AGA recommends enteral rather than parenteral nutrition	Strong	Moderate
6	In patients with predicted severe or necrotizing pancreatitis requiring enteral tube feeding, the AGA suggests either NG or NJ route	Conditional	Low
7	In patients with acute biliary pancreatitis, the AGA recommends cholecystectomy during the initial admission rather than after discharge	Strong	Moderate
8	In patients with acute alcoholic pancreatitis, the AGA recommends brief alcohol intervention during admission	Strong	Moderate

Adapted from Crockett 2018

Appendix 3. CTSI Score

Grade	CT Finding	Points
A	Normal pancreas	0
B	Enlargement of pancreas	1
C	Inflammatory changes in pancreas and peripancreatic fat	2
D	Ill-defined single peripancreatic fluid collection	3
E	Two or more poorly defined peripancreatic fluid collections	4

Pancreatic Necrosis	Points
None	0
<30%	2
30-50%	4
>50%	6

Adapted from Balthazar 2002

The CTSI score is the sum of the Balthazar and Pancreatic Necrosis scores:

- 0-3: mild acute pancreatitis
- 4-6: moderate acute pancreatitis
- 7-10: severe acute pancreatitis

Appendix 4. Pain Numeric Rating Scale (PNRS)

Pain Numeric Rating Scale

1. On a scale of 0 to 10, with 0 being no pain at all and 10 being the worst pain imaginable, how would you rate your pain RIGHT NOW.

0	1	2	3	4	5	6	7	8	9	10
No Pain										Worst Pain Imaginable

Appendix 5. Imputed PaO_2/FiO_2

FiO_2 \ SpO_2	0.7	0.75	0.8	0.81	0.82	0.83	0.84	0.85	0.86	0.87	0.88	0.89	0.9	0.91	0.92	0.93	0.94	0.95	0.96
**0.21**	174	191	211	216	221	226	232	238	245	252	260	269	279	291	304	319	337	360	~~390~~
**0.24**	153	167	185	189	193	198	203	208	214	221	228	236	244	254	266	279	295	315	341
**0.27**	136	148	164	168	172	176	180	185	190	196	202	209	217	226	236	248	262	280	303
**0.3**	122	133	148	151	155	158	162	167	171	177	182	189	196	203	213	223	236	252	273
**0.35**	105	114	127	129	132	136	139	143	147	151	156	162	168	174	182	191	202	216	234
**0.4**	92	100	111	113	116	119	122	125	129	132	137	141	147	153	159	168	177	189	205
**0.45**	81	89	98	101	103	106	108	111	114	118	121	126	130	136	142	149	157	168	182
**0.5**	73	80	89	91	93	95	97	100	103	106	109	113	117	122	128	134	142	151	164
**0.55**	67	73	81	82	84	86	89	91	94	96	99	103	107	111	116	122	129	138	149
**0.6**	61	67	74	76	77	79	81	83	86	88	91	94	98	102	106	112	118	126	136
**0.65**	56	62	68	70	71	73	75	77	79	81	84	87	90	94	98	103	109	116	126
**0.7**	52	57	63	65	66	68	70	71	73	76	78	81	84	87	91	96	101	108	117
**0.75**	49	53	59	60	62	63	65	67	69	71	73	75	78	81	85	89	94	101	109
**0.8**	46	50	55	57	58	59	61	63	64	66	68	71	73	76	80	84	89	95	102
**0.85**	43	47	52	53	55	56	57	59	61	62	64	67	69	72	75	79	83	89	96
**0.9**	41	44	49	50	52	53	54	56	57	59	61	63	65	68	71	74	79	84	91
**0.95**	39	42	47	48	49	50	51	53	54	56	58	60	62	64	67	71	75	80	86
**1**	37	40	44	45	46	47	49	50	51	53	55	57	59	61	64	67	71	76	82

Adapted from Fowler 2019

Appendix 6. Modified American Neurogastroenterology and Motility Society Gastrointestinal Cardinal Symptom Index Daily Dairy (mGCSI-DD)

	None	Mild	Moderate	Severe	Very Severe
Nausea (feeling sick to your stomach as if you are going to vomit or throw up)	☐	☐	☐	☐	☐
Not able to finish a normal sized meal (for a healthy person)	☐	☐	☐	☐	☐
Feeling excessively full after meals	☐	☐	☐	☐	☐
Abdominal Pain (around or above the navel)	☐	☐	☐	☐	☐
The next question asks you to record the number of times vomiting occurred in the last 24 hours. Please record the number of vomits (throwing up with food or liquid coming out) that occurred in the last 24 hours. Record zero, if you have not vomited during the past 24 hours. If you vomited, write down the number of all vomits. If you vomited once, record one. If you vomited three times during the day, record three. If you vomited three times, whether it was during the same trip to the bathroom or three separate trips, record three as the number of episodes of vomiting.<br><br>During the past 24 hours, how many episodes of vomiting did you have: ______________________					
In thinking about your pancreatitis, what was the overall severity of your symptoms over the past 24 hours	☐	☐	☐	☐	☐

Appendix 7. Schedule of Events

	Screening	If Eligible, Baseline Assessment	R	SFISD	24 (±1) Hours	48 (±1) Hours	72 (±2) Hours	96, 144, 192, 240 (±4) Hours[e,g]	120, 168, 216 (±4) Hours[e,g]	Days 12-28 Q48 (±4) Hours[e,g]	Day 30 (±5) Days
Informed Consent	X										
Demographics	X										X[h]
Medical History	X										
List of Medications	X										
Concomitant Meds				X	X	X	X	X	X		X
Height		X									
Weight		X					X				X
Vital Signs	X				X	X	X	X	X		X
Abdominal Exam	X										
PNRS		X			X	X	X	X	X		X
Serum Lipase	X[a]										
CBC+diff+Platelets	X[a]				X[a]	X[a]	X[a]		X[a]		X[a]
Presence of SIRS	X										
SpO_2+FiO_2		X			X	X	X	X	X		X
CECT of Pancreas[b]	X										X
Serum Pregnancy Test	X[a]										
Randomize Patient			X								
Serum Chemistries		X[a]			X[a]	X[a]	X[a]		X[a]		X[a]
Serum Procalcitonin		X[a]			X[a]	X[a]	X[a]		X[a]		X[a]
Serum IL-6[c]		X			X	X	X		X		X
Urine NGAL[c]		X			X	X	X		X		X
Administer Study Drug				X	X	X					
Serum PK[c]				X[f]	X[f]		X[f]				X[f]
Enhanced Recovery Strategy[d]				X	X	X	X	X	X	X	X[i]
mGCSI-DD[e]		X[e]					X[e]			X[e]	X[e]
AE/SAE Evaluation			X	X	X	X	X	X	X	X	X

a. Blood sample for serum pregnancy test, serum lipase and CBC+diff+Platelets at Screening will be analyzed at local laboratory. May use results obtained as standard of care if blood drawn in the previous 6 hours prior to consent. Following screening, all protocol-required blood samples, except as noted in Footnote “c”, will be analyzed at local laboratory. Baseline blood samples including biomarkers may be performed on blood drawn in the previous 6 hours of randomization. For all later assessment times, blood drawn in the previous 12 hours maybe used (except PK).

b. A CECT already performed in the 12 hours prior to the patient or LAR providing informed consent may be used as the Screening CECT. In addition to the Screening and Day 30 CECTs, CECTs performed as standard of care after randomization and to 25 days after SFISD will be captured and submitted for central reading. If the CECT is performed 25 to 29 days after SFISD, it will substitute for the 30-day CECT scan and will be captured.

c. Blood and urine samples for IL-6, Urine NGAL and serum PK will be analyzed at central laboratory. If site or patient does not participate in these assessments this will be documented at start of study and will not be considered a protocol deviation.

d. Patients will be offered a low fat, ≥500-calorie solid meal at each mealtime after the infusion of the first dose of study drug if alert and not on mechanical ventilation. If the patient does not wish to eat the solid meal when offered or is unable to tolerate the solid meal, they should then be offered a liquid meal. Determine for each meal if the patient ate ≥50% of the meal without vomiting or having abdominal pain in the two hours after the meal.

e. The mGCSI-DD score will be completed by all eligible patients at the baseline assessment and on the day of discharge, which will vary by patient as shown in schedule above. In addition, if the patient remains hospitalized at 96 and/or 168 hours and/or at Day 14 and/or Day 21 patient should be requested to record their symptoms at bedtime using the mGCSI-DD worksheet. Patients will be trained on completing the mGCSI-DD worksheet. Upon discharge, patient will record the mGCSI-DD score daily before bedtime until the Day 30 visit.

f. Blood samples for PK analysis will be drawn as soon as possible after the completion of the first infusion of study drug (+2 hours), prior to the start of second infusion of study drug (-30 min), at 72 hours (±2 hours) from the SFISD, and at Day 30. If the patient is discharged after the third infusion of study drug and prior to 72 hours from the SFISD, the blood sample will be drawn prior to discharge.

g. Study assessments continue daily, or every other day, as noted in schedule of events until discharge or Day 30. All patients return for Day 30 assessments if discharged earlier than Day 25.

h. At the Day 30 visit (or at the time of death if before Day 30), record the Investigator’s opinion on the etiology of the episode of AP.

i. Only patients who remain hospitalized at Day 30.

CM4620-203

A Randomized, Double-Blind, Placebo Controlled Dose-Ranging Study of Auxora in Patients with Acute Pancreatitis and Accompanying Systemic Inflammatory Response Syndrome (CARPO)

Sponsor: CalciMedica, Inc.
505 Coast Boulevard South, Suite 307
La Jolla, CA 92037

Sponsor Medical Contact: Sudarshan Hebbar, MD
(816) 838-7105 – Mobile
sudarshan@calcimedica.com – Email

FOR QUALIFIED INVESTIGATORS AND THEIR IRB/EC ONLY

Version: 4.0

Issue Date: October 05, 2023

IND 123533

Confidentiality Statement

The information contained in this document is confidential and proprietary property of CalciMedica, Inc. Any distribution, copying, or disclosure is strictly prohibited unless federal regulations or state law requires such disclosure. Prior written authorization of CalciMedica, Inc. is required for any disclosure or use of the information contained herein. Persons to whom the information is disclosed must know that it is confidential and that it may not be further disclosed by them.

SPONSOR APPROVAL AND SIGNATURE PAGE

DocuSigned by:
Sudarshan Hebbar
Signer Name: Sudarshan Hebbar
Signing Reason: I approve this document
Signing Time: 10/9/2023 | 5:23:17 PM PDT
732B16E369D74416B6B6AE164D8A9C32

10/9/2023

Sudarshan Hebbar, MD
Chief Medical Officer

Date

DocuSigned by:
Andy Cunningham, MD
Signer Name: Andy Cunningham, MD
Signing Reason: I approve this document
Signing Time: 10/9/2023 | 5:20:59 PM PDT
BE2370D8E006454AB92C665D175B6585

10/9/2023

Andrew Cunningham, MD
Senior Vice President, Clinical Development

Date

SYNOPSIS

**Protocol Number:**	CM4620-203
**Protocol Title:**	A Randomized, Double-Blind, Placebo Controlled Dose Ranging Study of Auxora in Patients with Acute Pancreatitis and Accompanying Systemic Inflammatory Response Syndrome (CARPO)
**Sponsor:**	CalciMedica, Inc.<br>505 Coast Blvd. South, Suite 307<br>La Jolla, CA 92037 USA
**Study Phase:**	2b
**Number of Patients and Sites:**	Approximately 216 patients with acute pancreatitis (AP) and accompanying systemic inflammatory response syndrome (SIRS) will be randomized at approximately 45 sites.
**CM4620-IE Dose and Route of Administration:**	Patients randomized to receive Auxora (also known as CM4620 (zegocractin) Intravenous Emulsion) will receive one of three dose levels, 2.0 mg/kg (1.25 mL/kg), 1.0 mg/kg (0.625 mL/kg), and 0.5 mg/kg (0.3125 mL/kg), every 24 hours (±1 hour) for three consecutive days for a total of three infusions (Section 5.3). Auxora will be administered intravenously as a continuous infusion over 4 hours via a bag and tubing compatible with lipid emulsions and using a 1.2-micron filter.
**Placebo Dose and Route of Administration**	Placebo will consist of the emulsion without any active pharmaceutical ingredient CM4620. Patients randomized to receive Placebo will receive one of three dose volumes, 1.25 mL/kg, 0.625 mL/kg, and 0.3125 mL/kg, every 24 hours (±1 hour) for three consecutive days for a total of three infusions (Section 5.3). Placebo will be administered intravenously as a continuous infusion over 4 hours via a bag and tubing compatible with lipid emulsions and using a 1.2-micron filter.
**Objectives:**	*Primary*:<br>• To assess the dose response and efficacy of three different dose levels of Auxora in patients with AP and accompanying SIRS;<br>• To assess the time to medically indicated discharge in patients who are responders to early tolerance of solid food intake versus non-responders.<br>*Secondary*:<br>• To assess the safety and tolerability of varying doses of Auxora in patients with AP and accompanying SIRS.

**Inclusion Criteria:**	All of the following must be met for a patient to be randomized into the study:<br>1. The diagnosis of AP has been established by the presence of abdominal pain consistent with AP together with at least 1 of the following 2 criteria:<br>a. Serum lipase > 3 times the upper limit of normal (ULN);<br>b. Characteristic findings of AP on abdominal imaging;<br>2. The diagnosis of SIRS has been established by the presence of at least two of the following four criteria:<br>a. Temperature < 36°C or > 38°C;<br>b. Heart rate > 90 beats/minute;<br>c. Respiratory rate >20 breaths/minute or arterial carbon dioxide tension ($PaCO_2$) <32 mmHg;<br>d. White blood cell count (WBC) >12,000 mm^3, or <4,000 mm^3, or > 10% immature (band) forms;<br>3. At least one of the following criteria is also present:<br>a. A peripancreatic fluid collection or a pleural effusion on a contrast-enhanced computed tomography (CECT) performed in the 24 hours before Consent or after Consent and before Randomization;<br>b. Abdominal examination documenting either abdominal guarding or rebound tenderness;<br>c. Hematocrit ≥44% for men or ≥40% for women;<br>4. The patient is ≥ 18 years of age;<br>5. Lack of pancreatic necrosis, pancreatic calcifications, pancreatic pseudocysts and no evidence for previous necrosectomy or pancreatic surgery identified by CECT performed in the 24 hours before Consent or after Consent and before Randomization;<br>6. A female patient of childbearing potential who is sexually active with a male partner is willing to practice acceptable methods of birth control for 180 days after the last dose of study drug. A female patient must not attempt to become pregnant for 180 days;<br>7. A male patient who is sexually active with a female partner of childbearing potential is willing to practice acceptable methods of birth control for 180 days after the last dose of study drug. A male patient must not donate sperm for 180 days;<br>8. The patient is willing and able to, or has a legal authorized representative (LAR) who is willing and able to, provide informed consent to participate, and to cooperate with all aspects of the protocol.

<table>
<tr><td>Exclusion Criteria:</td><td>Patients with any of the following conditions or characteristics must be excluded from randomizing:
1. Expected survival <6 months;
2. Suspected presence of cholangitis in the judgment of the treating physician;
3. The patient has a known history of:
a. Organ or hematologic transplant;
b. HIV, hepatitis B, or hepatitis C infection;
c. Chronic pancreatitis (Appendix 7);
4. Current treatment with:
a. Chemotherapy;
b. Immunosuppressive medications or immunotherapy (Section 5.4 for list of prohibited immunosuppressive medications and immunotherapy);
c. Pancreatic enzyme replacement therapy;
d. Hemodialysis or Peritoneal Dialysis
5. The patient is known to be pregnant or is nursing;
6. The patient has participated in another study of an investigational drug or therapeutic medical device in the 30 days before randomization;
7. Allergy to eggs or known hypersensitivity to any components of study drug.</td></tr>
<tr><td>Study Design:</td><td>This double blind, randomized, placebo-controlled study will evaluate the efficacy, safety, and tolerability of three different dose levels of Auxora in patients with AP and accompanying SIRS.

Approximately 216 patients will be randomized 1:1:1:1 into one of 4 groups using a computer-generated randomization scheme accessed through an interactive voice/web response system (IXRS). Randomization will be first stratified by gender (male or female) and then by risk for organ failure in the gender subgroups (higher or lower). Higher risk for organ failure is defined by the presence of both an elevated hematocrit (HCT ≥44% for men or ≥40% for women) and hypoxemia (imputed $PaO_2/FiO_2 \leq 360$). Lower risk for organ failure is defined by the absence of either or both an elevated hematocrit and hypoxemia (Figure 4). The PaO_2/FiO_2 will be determined using an arterial blood gas or imputed using pulse oximetry (Appendix 5).</td></tr>
</table>

	All patients will have received a Screening CECT of the abdomen/pancreas before being randomized into the study. CECTs performed as standard of care (SOC) may be used as the Screening CECT but must have been performed in the 24 hours before Consent or after Consent and before Randomization (Section 8.12.12)<br><br>The Start of First Infusion of Study Drug (SFISD) should occur within 8 hours of the patient or LAR providing informed consent. Patients randomized to Group 1 will receive 2.0 mg/kg of Auxora intravenously every 24 hours (±1 hour) for a total of three doses. Patients randomized to Group 2 will receive 1.0 mg/kg of Auxora intravenously every 24 hours (±1 hour) for a total of three doses. Patients randomized to Group 3 will receive 0.5 mg/kg of Auxora intravenously every 24 hours (±1 hour) for a total of three doses. Patients randomized to Group 4 will receive emulsion without any active pharmaceutical ingredient. Patients in Group 4 will receive one of three randomly assigned dose volumes, 1.25 mL/kg, 0.625 mL/kg, or 0.3125 mL/kg, which will be administered intravenously every 24 hours (±1 hour) for a total of three doses. (Section 5.3) The dosing will be based on actual body weight obtained at the time of hospitalization or screening for the study. As described in the pharmacy manual, the upper limit of the volume of Auxora and volume of Placebo that will be administered will be 156.25 mL. The sponsor, investigators, pharmacists, and patients will be blinded to the assigned group. In the event of a medical emergency, investigators will be able to receive the treatment assignment if required to provide optimal care of the patient.<br><br>For all 4 groups, a study physician or appropriately trained delegate will perform study-specific assessments at screening, at the baseline assessment, immediately prior to the SFISD, and then every 24 hours until 240 hours after the SFISD, or until discharge if earlier. If patients remain hospitalized at Day 12, assessments will then be performed every 48 hours starting on Day 12 until Day 28, or until discharge if earlier. Patients discharged from the hospital before Day 25 will return at Day 30 (+5 days) to perform the Day 30 assessments. If patients are discharged on Days 25-29, the Day 30 assessments may be performed prior to discharge.<br><br>Patients will receive another CECT of the abdomen/pancreas at the Day 30 (±5 days) visit. All CECTs performed as SOC after randomization and before the Day 30 CECT will also be captured. A blinded central reader will read the Screening, Day 30, and any SOC CECTs obtained between randomization and the Day 30 visit.<br><br>Patients will complete the modified American Neurogastroenterology and Motility Society Gastrointestinal Cardinal Symptom Index Daily Diary

	(mGCSI-DD) worksheet at the baseline assessment, at 96 hours, 168 hours, Day 14 and Day 21 (for patients who remain hospitalized on these days), on the day of discharge, and daily at bedtime after discharge until the Day 30 visit. Patients who are discharged on Days 25-29 will not complete the mGCSI worksheet after discharge.<br><br>It is recommended that all patients randomized in the study should receive care consistent with the 2018 American Gastroenterological Association (AGA) Institute Technical Review of the Initial Medical Management of Acute Pancreatitis. Patients should receive local SOC for the management of other medical conditions.<br><br>In patients with AP, the AGA strongly recommends early oral feeding (within 24 hours) rather than keeping the patient nil per mouth (Nil per Os, NPO). Patients randomized into the study, therefore, will be offered a low fat, ≥500-calorie solid meal at each mealtime after the infusion of the first dose of study drug if alert and not on mechanical ventilation, or if not NPO for a planned surgery/medical procedure, or if not NPO because of an acute medical condition. If the patient does not wish to eat the solid meal when offered or is unable to tolerate the solid meal, they should then be offered a liquid meal. The same approach should occur at each subsequent mealtime until patient discharge. When patients eat a solid meal, it should be recorded if they ate ≥50% of the meal and if they either vomited or experienced an increase in abdominal pain in the two hours after eating a meal.<br><br>It is also recommended that all patients randomized in the study should not be discharged from the hospital until solid food is tolerated, abdominal pain has resolved or been adequately controlled, and there is no clinical evidence of infection that would necessitate continued hospitalization. Tolerating solid food is defined as eating ≥50% of a low fat, ≥500-calorie solid meal without an increase in abdominal pain or vomiting. If the patient is not tolerating either solid or liquid meals, tube feedings should be considered.<br><br>All protocol required laboratory testing, except biomarker and pharmacokinetic (PK) samples, will be performed at the local laboratory. Results from the biomarkers and PK blood samples collected as part of the protocol and being tested at a central lab will not be available to assist the Principal Investigator (PI) or treating physician in managing the patient.

**Efficacy Assessments:**	Efficacy assessments will include the following:<br>• Enhanced Recovery Strategy for Tolerance of Solid Food<br>• Modified Gastrointestinal Cardinal Symptom Index Score<br>• CECT of the Pancreas<br>• Pain Numeric Rating Scale (PNRS) score<br>• Organ Failure scores<br>• Vital sign measurements<br>• Laboratory measurements<br>• Concomitant medications
**Safety Assessments:**	Safety assessments will include the following:<br>• Vital signs measurements<br>• Laboratory measurements<br>• Concomitant medications<br>• Treatment-emergent adverse events (TEAEs) and serious adverse events (SAEs)

**Efficacy Endpoints:**	**Primary endpoint**<br>• Time to solid food tolerance<br>**Secondary endpoints:**<br>• Solid food tolerance at 48 hours, 72 hours, and 96 hours after the SFISD and at discharge<br>• Time to medically indicated discharge<br>• Length of stay in the hospital<br>• Length of stay in the intensive care unit (ICU) for patients admitted to the ICU<br>• Re-hospitalization for AP by Day 30<br>• Change in severity of AP by CTSI score from screening to Day 30<br>• Development of pancreatic necrosis ≥30% and >50%<br>• The persistence of SIRS ≥48 hours after the SFISD<br>• Incidence, severity, and duration of organ failure<br>• Mortality by Day 30<br>• Change in pain score and opioid use<br>**Exploratory endpoints:**<br>• Development of infected pancreatic necrosis<br>• Development of sepsis<br>• Hospital procedures for the management of pancreatic necrosis<br>• Change in GCSI-DD score<br>• Change in albumin<br>• Change in absolute neutrophil count (ANC)/absolute lymphocyte count (ALC) ratio and IL-6 levels<br>• Change in urine neutrophil gelatinase-associated lipocalin (NGAL)
**Safety Endpoints**	• The incidence of TEAEs and SAEs<br>• The intensity and relationship of TEAEs and SAEs<br>• Clinically significant changes in vital signs and safety laboratory results
**Pharmacokinetic Assessments:**	In patients at selected sites, sparse sampling of plasma will be employed for PK assessment after finishing the first and third doses of study drug.
**Statistical Considerations**	**Sample Size Calculations**<br>A sample size of 216 patients will be randomized into four groups on a 1:1:1:1 basis, resulting in 54 patients randomized into each group. This sample size will provide 86% power for testing the difference in two populations having a median length of time of 72 and 144 hours, respectively, to tolerating solid food based on a log-rank test. This sample size will provide 80% power with a two-sided alpha of 0.05 (using the chi-squared test) to detect a 45% response rate for tolerating solid food in a Auxora

	dose group versus a 20% response rate in the Placebo group in the 72 hours after completion of the first study drug infusion.<br><br>**Treatment Populations**<br>• Modified Intent to Treat Population will consist of all randomized patients who receive any amount of study drug<br>• The Per-Protocol Population (PP) will consist of all randomized patients who complete the study and have no major protocol violations<br>• The PK population will consist of all randomized patients who receive any amount of active study drug and who have at least one post baseline PK sample analyzed<br><br>A full Statistical Analysis Plan (SAP) detailing the primary and secondary efficacy analyses and the safety analyses, will be developed. Data evaluation will include all primary and secondary endpoints, and will be based on descriptive, exploratory, and inferential statistical methods. The number of patients per group, means, standard deviations, medians, quartiles, minimum and maximum values will be determined for continuous variables. Absolute and percentage frequencies will be determined for categorical variables. Handling of missing data and additional sensitivity analyses will be described in the statistical plan.

Table of Contents

Sponsor Approval and Signature Page 2
Synopsis 3
List of Tables 15
List of Figures 15
List of Terms and Abbreviations 16
1 INTRODUCTION 19
1.1 Acute Pancreatitis 19
1.2 Pathophysiology of Acute Pancreatitis 20
1.3 Overview of CM4620 22
1.4 Pre-Clinical Development of Zegocractin 22
1.4.1 Pre-Clinical Safety and Toxicology Studies 22
1.4.2 Preclinical Efficacy Studies 23
1.5 Clinical Development of Auxora 25
1.5.1 Single Ascending Dose and Multiple Ascending Dose Studies 25
1.5.2 Open Label Study in Patients with AP and SIRS 27
1.5.3 Pharmacodynamic and Pharmacokinetic Study in Patients with AP 30
1.6 Rationale for the Study and Selected Doses 31
2 OBJECTIVES AND ENDPOINTS 34
2.1 Primary Objectives 34
2.2 Secondary Objective 34
2.3 Efficacy Endpoints 34
2.4 Safety Endpoints 34
3 INVESTIGATIONAL PLAN 36
3.1 Study Design 36
3.2 End of Study 38
3.3 Sponsor Termination of the Study 38
4 SELECTION OF PATIENTS 39
4.1 Inclusion Criteria 39
4.2 Exclusion Criteria 40
4.3 Re-Screening 40

5 TREATMENT OF PATIENTS ... 41
5.1 Overview ... 41
5.2 Enhanced Recovery Strategy for Tolerance of Solid Food ... 41
5.3 Discharge Criteria ... 41
5.4 Prohibited Medications ... 42
5.5 Compliance ... 42
6 PROCEDURES ... 43
6.1 Randomization Procedures ... 43
6.2 Discontinuation and Withdrawal ... 43
7 STUDY DRUG MATERIALS AND MANAGEMENT ... 44
7.1 Auxora Product Description ... 44
7.2 Placebo Product Description ... 44
7.3 Auxora and Placebo Storage ... 44
7.4 Auxora and Placebo Preparation ... 45
7.5 Auxora and Placebo Administration ... 45
7.6 Packaging and Labeling ... 45
7.7 Accountability, Handling and Disposal ... 46
8 VISITS AND ASSESSMENTS ... 47
8.1 Screening ... 47
8.2 Baseline Assessment ... 48
8.3 Randomization Using IXRS System ... 48
8.4 Start of First Infusion of Study Drug (SFISD) ... 48
8.5 24 hours ... 49
8.6 48 hours ... 49
8.7 72 hours ... 50
8.8 96, 120, 144, 168, 192, 216, 240 hours (±4 hours) ... 51
8.9 Days 12 to 28 (±4 hours) ... 51
8.10 Day 30 (±5 days) ... 52
8.11 Discharge Date ... 53
8.12 Study Assessments ... 53
8.12.1 Demographics, Medical History and Medications ... 53
8.12.2 Concomitant Medications ... 54

8.12.3 Abdominal Examination 54
8.12.4 Vital Signs 54
8.12.5 Arterial Blood Gas 54
8.12.6 SpO_2/FiO_2 55
8.12.7 Laboratory Analyses 55
8.12.8 PK analysis 56
8.12.9 Enhanced Recovery Strategy for Tolerance of Solid Food 56
8.12.10 Modified ANMS GCSI-Daily Diary (mGCSI-DD) 57
8.12.11 Pain Numeric Rating Scale (PNRS) 57
8.12.12 Contrast-Enhanced Computed Tomography (CECT) of the Abdomen/Pancreas 57
8.12.13 Systemic Inflammatory Response Syndrome (SIRS) 58
9 ADVERSE EVENTS 59
9.1 Definition of Adverse Event 59
9.2 Definition of Serious Adverse Event 59
9.3 Eliciting Adverse Event Information 60
9.4 Recording Adverse Events 60
9.5 Assessment of Relationship to Study Drug 61
9.6 Assessment of Severity 61
9.7 Reporting of Serious Adverse Events 62
9.8 Suspected Pregnancy in a Woman of Childbearing Potential 63
10 STATISTICAL METHODS 65
10.1 General Considerations 65
10.2 Sample Size 65
10.3 Study Assessments 65
10.4 Analysis Sets 66
10.4.1 Efficacy and Safety 66
10.4.2 Pharmacokinetics 66
10.5 Disposition 66
10.6 Analysis of Demographic and Baseline Data 66
10.7 Efficacy Analysis 67
10.8 Safety Analysis 68

10.9 Independent Data Monitoring Committee 69
11 ADMINISTRATIVE CONSIDERATIONS 70
11.1 Electronic Case Report Forms 70
11.2 Monitoring of the Study 70
11.3 Inspection of Records 70
11.4 Study Record Retention 70
11.5 Study Conduct: Good Clinical Practice and Declaration of Helsinki 70
11.6 Responsibilities of the Investigator and the IRB/EC 71
11.7 Confidentiality 71
11.8 Modification of the Protocol 71
11.9 Informed Consent 72
11.10 Protocol Deviations 72
11.11 Financial Disclosure 73
11.12 Sponsor Obligations 73
11.13 Investigator Documentation 73
11.14 Clinical Study Insurance 74
11.15 Use of Information 74
11.16 Publications 74
12 REFERENCES 75
Appendix 1. Definition of Organ Failure and Sepsis 78
Appendix 2. American Gastroenterological Association Institute Guideline on the Initial Management of Acute Pancreatitis 80
Appendix 3. CTSI Score 81
Appendix 4. Pain Numeric Rating Scale (PNRS) 82
Appendix 5. Imputed PaO_2/FiO_2 83
Appendix 6. Modified American Neurogastroenterology and Motility Society Gastrointestinal Cardinal Symptom Index Daily Dairy (mGCSI-DD) 84
Appendix 7. CT Findings in Chronic Pancreatitis 85
Appendix 8. Schedule of Events 86

List of Tables

Table 1. SAD (CM4620-101) 25
Table 2. MAD (CM4620-102) 26
Table 3. Demographics and Baseline Characteristics of Patients in CM4620-201 28
Table 4. Auxora Product Information 44
Table 5. Conversion of O_2 Flow to FiO_2 55

List of Figures

Figure 1. Schema Showing the Activation of CRAC Channels Found on Pancreatic Acinar Cells Triggering a Series of Events Which Lead to Acute Pancreatitis........ 21
Figure 2. Sites of action of Zegocractin in treating acute pancreatitis 24
Figure 3. Stimulated IL-2 production by WBCs in response to administration of Auxora 31
Figure 4. Higher versus Lower Risk of Organ Failure 36

List of Terms and Abbreviations

Abbreviation	Definition
ABG	arterial blood gas
AE	adverse event
AGA	American Gastroenterological Association
ALC	absolute lymphocyte count
ALT	alanine transaminase
ANC	absolute neutrophil count
ANMS	American Neurogastroenterology and Motility Society
AP	acute pancreatitis
AST	aspartate transaminase
ATP	adenosine triphosphate
AUC	Area under the concentration-time curve
Ca^{2+}	Calcium ion
CCK	cholecystokinin
CECT	Contrast-enhanced computed tomography
CFR	Code of Federal Regulations
C_{max}	Maximum plasma concentration
CMH	Cochran Mantel-Haenszel
CPK	Creatine kinase
CR	Complete response
CRAC	calcium release-activated calcium
CRF	case report form
CRP	C-Reactive Protein
CTSI	computed tomography severity index
CV	cardiovascular
EC	Ethics Committee
ECG	electrocardiogram
eCRF	Electronic case report form
EDC	Electronic Data Capture
EDTA	edetate disodium salt dehydrate
ER	endoplasmic reticulum
ERCP	endoscopic retrograde cholangiopancreatography
FAEE	fatty acid ethyl ester
FDA	U.S. Food and Drug Administration
FiO_2	fraction of inspired oxygen
GFR	glomerular filtration rate
GMP	Good Manufacturing Practice of Medicinal Products
HCT	Hematocrit

Abbreviation	Definition
Hr(s)	hour(s)
IC_{50}	Concentration producing a mean 50% inhibition
ICH	International Conference on Harmonization
I_{CRAC}	calcium release-activated calcium current
ICU	Intensive care unit
IDMC	Independent Data Monitoring Committee
IP	intraperitoneal
IP_3R	inositol 1,4,5-trisphosphate (IP3) receptor
IRB	Institutional Review Board
IV	intravenous/intravenously
IXRS	interactive voice/web response system
Kg	kilogram
L	liter
LAR	legal authorized representative
LDH	lactate dehydrogenase
LTF	Lost to follow-up
MAD	multiple ascending dose
MedDRA	Medical Dictionary for Regulatory Activities
mGCSI-DD	Modified American Neurogastroenterology and Motility Society Gastrointestinal Cardinal Symptom Index Daily Diary
MITT	Modified Intent-to-treat Population
MPO	myeloperoxidase
NF-κB	nuclear factor kappa B
NFAT	nuclear translocation of Nuclear Factor of Activated T cells
NG	nasogastric
NGAL	Neutrophil gelatinase-associated lipocalin
NJ	nasojejunal
NOAEL	no observable adverse effect level
NPO	Nil per Os (Nil per Mouth)
OR	odds ratio
Orai1	Calcium release-activated calcium channel protein 1
PaO_2/FiO_2	P/F Ratio
PI	Principal Investigator
PD	pharmacodynamic
PK	pharmacokinetic
PNRS	pain numeric rating scale
popPK	population PK
PP	Per-Protocol Population
PT	preferred Term

Abbreviation	Definition
QTcF	QT interval corrected for HR using Fridericia's method
SAD	single ascending dose
SAE	Serious adverse event
SaO_2	arterial blood oxygen saturation measured directly
SAP	Statistical analysis plan
SC	Supportive Care
SFISD	Start of First Infusion of Study Drug
SIRS	Systemic inflammatory response syndrome
SOC	Standard of care
SOC	System Organ Class
SOCE	Store-operated calcium entry
SpO_2	Oxyhemoglobin percent saturation measured by pulse oximetry
STIM1	Stromal interaction molecule 1
TEAE	Treatment-emergent adverse event
TLCS	taurolithocholate acid sulphate
T_{max}	time it takes for drug to reach maximum concentration
ULN	upper limit of normal
VTBI	Volume to be infused
WBC	White blood cell

1 INTRODUCTION

1.1 Acute Pancreatitis

Acute pancreatitis (AP) is an acute inflammatory process of the pancreas with varying involvement of local tissues and/or more remote organ systems. Due to the dynamic nature of the disease, it leads to wide-ranging outcomes that evolve rapidly in any given patient with little predictability. There is no prescribed order of events that the disease course follows, beyond the basic concept of an early (usually <1-2 weeks) and a late (>1-2 weeks) phase, with the former characterised by varying degrees of pancreatic and systemic inflammation, and the latter by an anergic phase that can make patients susceptible to infection.

Due to the evolving nature of AP, the severity may change during the course of the disease. The Atlanta classification of 2012 defines three degrees of severity: mild AP, moderately severe AP, and severe AP (Banks 2013). Terminology that is important in this classification includes transient organ failure, persistent organ failure, and local or systemic complications. Transient organ failure is organ failure that is present for < 48 hours. Persistent organ failure is defined as organ failure that persists for ≥ 48 hours. Local complications include peripancreatic fluid collections and acute necrotic collections, while systemic complications can be related to exacerbations of underlying co-morbidities. A new international classification, referred to as the 'determinant-based classification of severity', has been proposed as an alternative to the Atlanta classification (Dellinger 2012). This classification is based on the actual local and systemic determinants of severity, rather than a description of events that are correlated with severity. The derivation of a classification based on the above principles results in four categories of severity: mild, moderate, severe, and critical. The determinant-based classification approach to severity has elicited a great deal of interest; nevertheless, the Atlanta classification remains the more commonly used standard against which severity of AP is assessed.

AP can be subdivided into two histopathological subtypes: interstitial oedematous pancreatitis and necrotising pancreatitis. In the majority of cases, AP comprises clinically of a mild transitory form of oedematous-interstitial inflammation, which is self-limiting and resolves spontaneously. However, 15–20% of patients with AP will develop the more severe form of the disease (Whitcomb 2006). Necrotising pancreatitis most commonly manifests as necrosis involving both the pancreas and peripancreatic tissues and, less commonly, as necrosis of only the peripancreatic tissue or pancreatic parenchyma alone. Patients with peripancreatic necrosis alone have increased morbidity and intervention rates compared to patients with interstitial oedematous pancreatitis (Singh 2011). Repeated attacks of AP can result in chronic pancreatitis, which increases the risk of developing pancreatic cancer (Yadav 2013).

Gallstones and alcohol misuse are long-established risk factors for AP. The next most common cause is thought to be endoscopic procedures, e.g., endoscopic retrograde cholangiopancreatography (ERCP). Other causes include surgery or trauma, metabolic problems, infections, hereditary factors and drugs. A small residual group has no obvious cause and is labelled ''idiopathic.'' However, many of these may be due to unidentified microlithiasis (cholesterol crystals, biliary

sludge, or small stones). Smoking is an independent risk factor, and its effects could be synergistic with those of alcohol (Lindkvist 2008; Tolstrup 2009).

There are currently two primary aims in the initial treatment of patients with AP. The first aim is to provide supportive therapy, and to treat specific complications that may occur. The second aim is to limit the severity of pancreatic inflammation, necrosis, and systemic inflammatory response syndrome (SIRS) (Werner 2005).

There is currently no approved pharmaceutical treatment for AP, regardless of the severity of the disease (Lankisch 2015). Fluid resuscitation is at the cornerstone of early treatment of AP (Lankisch 2015). Analgesics, usually opioids, are administered initially to manage the abdominal pain. Enteral feeding can also be used and is preferred over exclusive parenteral nutrition. Thus, patients are generally managed with supportive care, including fluid replacement, painkillers, oxygen, feeding via a tube or into a vein, and antibiotics. Severely ill patients with organ failure are managed aggressively using a variety of different treatment strategies.

1.2 Pathophysiology of Acute Pancreatitis

The exocrine pancreas is a highly specialised secretory organ capable of synthesising, storing and releasing large quantities of digestive enzyme precursors into the small intestine, essential for the breakdown of food. Homeostasis of the functional cellular unit, the acinar cell, is therefore paramount for the smooth running of physiological processes; disruption can lead to severe damage of the pancreas, resulting in premature activation of zymogens, vacuolization and necrotic cell death, features typical of AP.

The pancreatic acinar cells are responsible for synthesising digestive proenzymes such as trypsinogen, which, when secreted with pancreatic fluids, become activated in the gut to help digest food. This secretory process is regulated through cytosolic calcium levels in the pancreatic acinar cells. At physiological concentrations, the neurotransmitter acetylcholine and the hormone cholecystokinin (CCK) evoke repetitive short-lasting increases in cytosolic calcium ions (Ca^{2+}) in pancreatic acinar cells, provided by Ca^{2+} release from internal stores such as the endoplasmic reticulum (ER) (Petersen 2006). Repetitive release of Ca^{2+} from internal stores triggers activation of store operated calcium entry (SOCE) through Calcium Release-Activated Calcium (CRAC) channels to replenish the stores from interstitial fluid and activates mitochondria to produce adenosine triphosphate (ATP) (Petersen 2011; Gerasimenko 2013). Both Ca^{2+} and ATP are required for the secretion of the digestive proenzymes and the accompanying fluid needed to wash the secreted proteases out of the duct system and into the gut (Petersen 2011).

The triggers of AP, such as gallstones and alcohol, lead to massive and sustained release of ER Ca^{2+} stores, resulting in maintenance of high cytosolic Ca^{2+} levels and a Ca^{2+} depleted state of the stores (Muallem 1988). The sustained cytosolic Ca^{2+} elevation is due to excessive Ca^{2+} entry into the cells from the interstitial fluid following depletion of ER Ca^{2+} stores. Mechanistically, when the ER stores are depleted of Ca^{2+} the acinar cell attempts to refill the stores with Ca^{2+} entering through a plasma membrane CRAC channel, with Orai1 being the key Ca^{2+}-conducting

component of the channel complex (Lur 2009). The opening of the Orai1 channel is activated by STIM1 in response to ER Ca^{2+} store depletion (Gerasimenko 2013).

The massive release of ER Ca^{2+} followed by the sustained elevation in cytosolic Ca^{2+} causes the digestive enzymes to be activated inappropriately within the acinar cells, resulting in autodigestion (i.e., digestion of the pancreas and surrounding tissue), necrosis (Phillip 2014), and the characteristic features of AP. Elevated cytosolic Ca^{2+} also results in lower production of ATP by mitochondria, which limits the cell's ability to handle stress and further promotes necrosis (Criddle 2016). Figure 1 shows the sequence of events leading to AP following activation of CRAC channels.

Figure 1. Schema Showing the Activation of CRAC Channels Found on Pancreatic Acinar Cells Triggering a Series of Events Which Lead to Acute Pancreatitis.

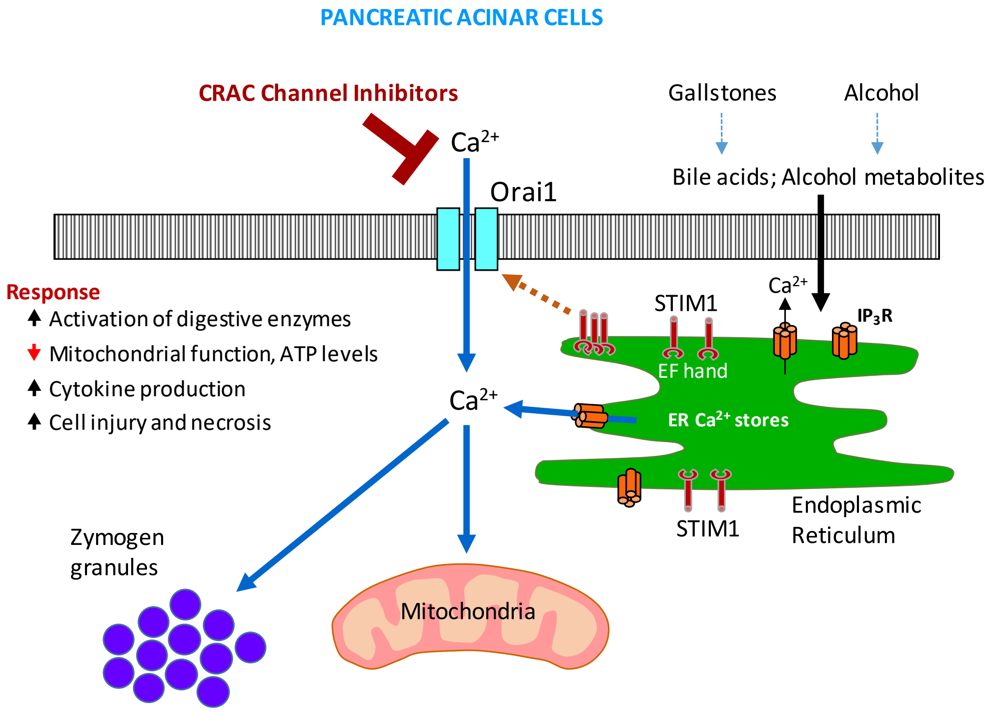

1.3 Overview of CM4620

CM4620 (also known by the international nonproprietary name, zegocractin) is a potent and selective inhibitor of CRAC channels. CRAC channels are composed of the pore-forming plasma membrane protein Orai1 and the calcium-sensing ER gating-protein STIM1. Low levels of calcium within the ER cause the STIM1 protein to oligomerize and move to locations closely apposed to Orai1. When STIM1 binds to Orai1, the Orai1 Ca^{2+} pore opens, permitting entry of extracellular calcium into the cell through the CRAC channel. This process is referred to as SOCE and evidence suggests that SOCE through CRAC channels plays a critical role in the degradation and necrosis of pancreatic acinar cells in patients with AP.

The potential for zegocractin to inhibit CRAC channels was investigated by measuring the electrophysiological current (calcium release-activated calcium current; I_{CRAC}) associated with calcium entry through CRAC channels in HEK293 cells stably expressing recombinant human Orai1/STIM1 (Wen 2015). Cellular recordings were performed using the whole-cell patch clamp method. Measurements of I_{CRAC} were made after the addition of extracellular 10 mM calcium chloride and subsequent administration of zegocractin at concentrations of 0.001, 0.01, 0.1, and 1 µM. Zegocractin was able to inhibit I_{CRAC} in a concentration-dependent manner, producing a mean 50% inhibition (IC_{50}) value of 119 nM. Rapid and complete inhibition was achieved at 1 µM of CM4620. Evaluations were performed to further elucidate the site of action of zegocractin. A mutation in Orai1 (Orai1-V102C) is known to produce constitutively active CRAC channels without the need for STIM1. In this evaluation, successive concentrations of zegocractin produced nearly complete inhibition of the STIM1-independent I_{CRAC}, indicating that Orai1 is a major site of action of the compound.

1.4 Pre-Clinical Development of Zegocractin

1.4.1 Pre-Clinical Safety and Toxicology Studies

Safety pharmacology studies conducted in rats indicated no zegocractin-induced adverse effects on central nervous or respiratory systems. Dose-limiting adverse clinical and cardiovascular effects were noted in a single telemetered cynomolgus monkey dosed at 25 mg/kg intravenously (IV) with Auxora (zegocractin intravenous emulsion). Cardiovascular data at lower doses (1, 3 and 10 mg/kg) showed transient, non-dose-related, slight-to-moderate increases in systolic/diastolic arterial blood pressures and negative chronotropic effects (mild and non-adverse) at all doses and in placebo treated animals.

Repeat-dose toxicity studies conducted in both rats and monkeys showed no observable adverse effect levels (NOAELs) of 25 mg/kg/day and 3 mg/kg/day, respectively. *In vitro* genetic toxicity studies were negative in the Ames bacterial reverse mutation assay and weakly positive/equivocal in a micronucleus assay conducted in human peripheral blood lymphocytes. A subsequent *in vivo* micronucleus study conducted in rats involving two different endpoints (bone marrow micronucleus and liver Comet assays) showed no evidence of DNA reactivity. Based on the results of the complete battery of genotoxicity testing, the weight of evidence indicates that zegocractin is neither mutagenic nor clastogenic. Hemolysis testing concluded that zegocractin placebo was

compatible with human plasma and non-hemolytic in human blood. Specific local tolerance studies to examine irritation/inflammation at the injection site were not performed, but no evidence of compound-related or vehicle-related local irritation was observed in the repeat-dose toxicity studies in rat and monkey. Finally, *in vitro* 3T3 results indicated that zegocractin is potentially phototoxic, so appropriate precautions are being taken in clinical trials.

1.4.2 Preclinical Efficacy Studies

Non-clinical studies conducted in murine and human pancreatic acinar cells to date have demonstrated that by acting on CRAC channels in pancreatic acinar cells, zegocractin can protect these cells from pancreatitis-induced cell death and can reduce biochemical, immunological and histopathologic consequences of experimental AP when administered early in the course of the disease (Wen 2015; Waldron 2019). Zegocractin caused marked reductions in serum amylase activity, pancreatic trypsin activity, myeloperoxidase (MPO) activity (a marker of neutrophil infiltration) in the pancreas and lung and led to a significant reduction in pancreatic histopathology scores. Importantly, the non-clinical findings provide proof of concept that zegocractin has the potential to be effective in the treatment of AP regardless of the cause and support the administration of zegocractin as an IV infusion to humans. Additional non-clinical evidence supports the position that the effects of zegocractin on the pancreas are complimented by its ability to reduce inflammatory responses that can manifest clinically as SIRS.

The protection offered by zegocractin *in vitro* has been confirmed in three diverse *in vivo* models representative of gallstone-, alcohol-, or hyper-stimulation–induced AP (taurolithocholate acid sulphate [TLCS]-induced, fatty acid ethyl ester [FAEE]-induced and cerulein-induced pancreatitis models, respectively). Trypsin activity within pancreatic tissue, MPO activity within pancreatic and lung tissue, and histopathological indices of pancreatic damage, such as edema, inflammatory cell infiltration, vacuolization, and necrosis, were all markedly reduced following single 5-20 mg/kg intraperitoneal (IP) doses of CM4620 in the mouse (cerulein-induced AP), two 5 or 20 mg/kg IP doses of CM4620 in the mouse (TLCS-induced and FAEE-induced APs models) or one 4-hour IV infusion of CM4620 Nano-emulsion (the intended clinical dosage form, route of administration and infusion duration) at doses of 5-20 mg/kg in the rat (cerulein-induced AP model) (Wen 2015; Waldron 2019). The timing of zegocractin administration relative to induction of pancreatitis was investigated in the TLCS-induced and FAEE-induced AP models and the results suggest that zegocractin may be more effective in minimizing pancreatic injury and subsequent downstream events if it is administered early in the course of disease, although later administration retains effectiveness in halting disease progression (Wen 2015).

The potential for zegocractin to reduce SIRS through an inhibitory effect on immune cells has been investigated *in vitro*, *ex vivo* and as part of *in vivo* pancreatitis models. *In vitro*, zegocractin inhibited human neutrophil function and inhibited release of key inflammatory cytokines (IL-2, IFNγ and IL-17) from human lymphocytes (IC_{50} 4-138 nM). Results from *ex vivo* pharmacodynamic (PD) analyses in blood demonstrated that zegocractin was able to significantly reduce stimulated IL-2 secretion from T cells, and presumably other lymphocytes, in a dose-dependent manner in Sprague Dawley rats and cynomolgus monkeys after 14 days of oral dosing. Further, the effects of zegocractin on MPO activity in the lung of mice in TLCS-induced

and FAEE-induced AP models and rats in the caerulein-induced AP model were assessed. MPO activity is a measure of neutrophil infiltration and when seen in the lung in these models it is indicative of a systemic inflammatory process analogous to SIRS in humans. Two IP doses of zegocractin at 20 mg/kg in mice (Wen 2015) or one 4-hour infusion of Auxora at 5 mg/kg in rats (Waldron 2019) were able to substantially reduce lung MPO activity (80% reduction in the mouse TLCS model, 73% reduction in the rat caerulein model). Lastly, in the IV infusion study in rats, Auxora significantly reduced IL-6 and TNFα mRNA levels in both pancreas and lung, as well as that of MPO.

In addition to the above, *in vitro* treatment with zegocractin protected mouse, rat and human pancreatic acinar cells from TLCS-induced necrosis or trypsinogen activation, blocked CCK-induced nuclear translocation of Nuclear Factor of Activated T cells (NFAT) translocation in isolated mouse acinar cells, CCK-induced nuclear factor kappa B (NF-κB) translocation in a rat acinar cell line, and lipopolysaccharide-induced expression of fibro-inflammatory mRNAs in mouse pancreatic stellate cells Furthermore, a single therapeutic IV infusion of Auxora in rat was shown to reduce markers of ER stress-associated cell death and apoptosis in rat pancreas (Waldron 2019).

Figure 2 highlights the potential role of zegocractin in treating AP through its effects on both the acinar cell and the immune system:

Figure 2. Sites of action of Zegocractin in treating acute pancreatitis

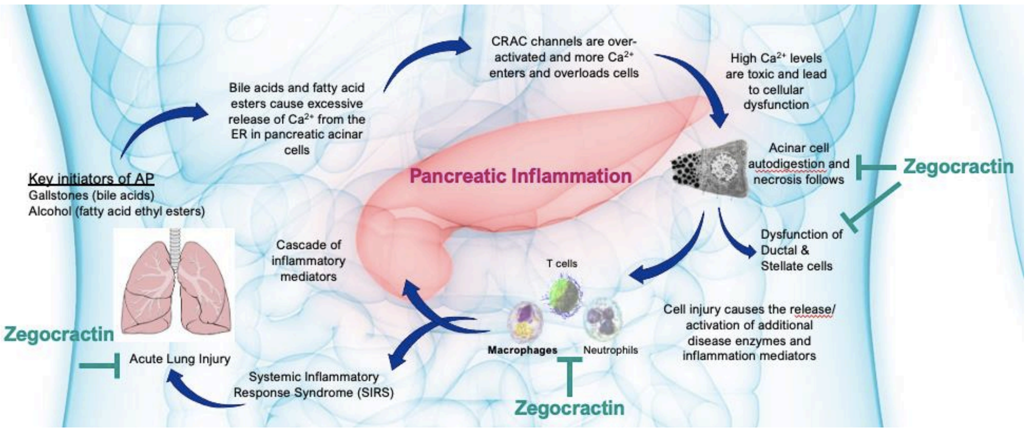

1.5 Clinical Development of Auxora

1.5.1 Single Ascending Dose and Multiple Ascending Dose Studies

CalciMedica has conducted two Phase 1 studies of Auxora in healthy subjects: a single ascending dose (SAD) study (CM4620-101) and a multiple ascending dose (MAD) study (CM4620-102). In CM4620-101 (Table 1), 32 healthy subjects were enrolled in five groups and randomized in a 3:1 ratio to receive a single dose of active versus placebo. The dose levels for each group are noted in Table 1. The dose volume of the emulsion was fixed at 1.3 mL/kg for all subjects in the SAD study groups, and Aurora or placebo was administered via a 4-hour IV infusion.

Table 1. SAD (CM4620-101)

Group	Active Treatment	Number of Active Treatment Subjects	Number of Placebo Treatment Subjects	IV Dose Volume (mL/kg)
1	0.1 mg/kg	6	2	1.3
2	0.24 mg/kg	3	1	1.3
3	0.48 mg/kg	3	1	1.3
4	1.0 mg/kg	6	2	1.3
5	2.1 mg/kg	6	2	1.3

IV = intravenous

Of the 32 enrolled subjects, there were no serious adverse events (SAE) or adverse events (AE) classified as moderate or severe in intensity. There were three clinical AEs that were all classified as mild in intensity. Two of the AEs were considered possibly related and one was considered unlikely or unrelated to study treatment. In each case, no action was taken with respect to study treatment because of the AEs. No laboratory abnormalities were observed that were considered clinically significant. There were vehicle-related increases in serum triglyceride and cholesterol levels noted in some subjects that returned to baseline within 24 hours. There was no evidence of any sustained treatment related increase in systolic or diastolic blood pressure. In addition, cardiac function, monitored by continuous electrocardiographic recording and serial biomarker testing, showed no evidence of any treatment related effect on heart rate, QTcF, cardiac troponin-T or B-type natriuretic peptide levels.

In the SAD study (CM4620-101), interim non-compartmental pharmacokinetic (PK) analysis indicates that Auxora likely distributes to three compartments. Plasma concentrations of compound rise steadily during the 4-hour infusion, with T_{max} achieved at the end of infusion (4 hours). After the end of infusion, there is a rapid and prominent distribution phase followed by a prolonged period of residual drug levels. The terminal elimination phase has not yet been fully characterized as it appears to be much longer than was anticipated based on pre-clinical PK data in mouse, rat, dog and monkey. Plasma concentrations during the terminal phase are approximately 5% of maximum serum concentration (C_{max}) values and, as indicated above, to date there have been no clinically significant AEs reported during this phase. Plasma exposures, defined by the area under the concentration-time curve $(AUC)_{0-24h}$, appear to be dose-proportional and reached a

maximum of 6710 ng*h/mL in Group 5, which is 4.3-fold below the mean AUC_{24h} in monkey at the NOAEL (29,000 ng*hr/mL).

In the MAD study (Table 2) of Auxora (CM4620-102), subjects in the first group were randomized to receive a single dose of active treatment, 0.50 mg/kg, versus placebo for seven consecutive days. Eight healthy subjects were enrolled in the first group, with five receiving active treatment and three receiving placebo. One of the subjects received placebo at the maximum dose volume of emulsion, 1.3 mL/kg, for 7 days, whereas all others were dosed on a weight-based adjustment of dose volume. There were no SAEs and no AEs classified as moderate or severe in intensity. There were 15 clinical AEs that were all classified as mild. In each case, no action was taken with respect to study treatment because of the AEs. No laboratory abnormalities were observed that were considered clinically significant.

Table 2. MAD (CM4620-102)

Group	Active Treatment Daily for 7 days	Number of Active Treatment Subjects	Number of Placebo Treatment Subjects	IV Dose Volume (mL/kg)
1	0.5 mg/kg	5	3	0.3125a
2	1.0 mg/kg	6	2	0.625

IV = intravenous
[a] one placebo patient received maximum dose volume of 1.3 mL/kg

Subjects in the second group of CM4620-102 were randomized to receive a single dose of active treatment, 1.0 mg/kg, versus placebo for seven consecutive days. Eight healthy subjects were enrolled in the second group, with six receiving active treatment and two receiving placebo for seven consecutive days. There were no SAEs and no AEs classified as moderate or severe in intensity. There were three AEs that were all classified as mild in intensity. In each case, no action was taken with respect to study treatment because of the AEs. No laboratory abnormalities were observed that were considered clinically significant.

There were vehicle-related increases in serum triglyceride noted in some subjects in both groups with levels returning to baseline within 24 hours. Cholesterol levels accumulated in some subjects in both groups with daily dosing, but the increases were not considered clinically significant and were related to the vehicle. Thus, the largest rise in cholesterol levels was in the subject who received placebo at the maximum dose volume of emulsion. The rise in cholesterol is believed to be due to the release of tissue cholesterol induced by the lecithin in the emulsion (Byers 1962), was noted in the pre-clinical studies in monkeys, and was reversible with cessation of dosing. There was no evidence of any sustained treatment related increase in systolic or diastolic blood pressure. In addition, cardiac function, monitored by continuous electrocardiographic recording and serial biomarker testing, showed no evidence of any sustained treatment related effect on heart rate, QTcF or B-type natriuretic peptide levels.

Non-compartmental PK analysis of Group 1 in CM4620-102 (0.5 mg/kg) indicates that zegocractin accumulated in plasma, with a 2.6-fold increase in systemic exposure (AUC_{24h}) on Day 7 compared to Day 1 of dosing, consistent with modeling simulations. C_{max} accumulated 1.6-fold (geometric mean of 363 ng/mL on Day 7). The geometric mean of the AUC_{24h} on Day 7

was 3190 ng*hr/mL, which is 9.1-fold below the NOAEL AUC_{24h} in monkey (29,000 ng*hr/mL). PK analysis of Group 2 in CM4620-102 (1.0 mg/kg) indicates that CM4620 accumulated in plasma, with a 2.6-fold increase in AUC_{24h} on Day 7 compared to Day 1 of dosing, consistent with modeling simulations. C_{max} accumulated 1.4-fold (geometric mean 637 ng/mL on Day 7). The geometric mean of the AUC_{24h} on Day 7 was 6830 ng*hr/mL, which is 4.2-fold below the NOAEL AUC_{24h} in monkey (29,000 ng*hr/mL). After the end of 7 days of infusion, there remained a prolonged period of residual drug levels in both MAD groups that remained significantly lower than the C_{max} on Day 7. A 3rd group of healthy subjects in the MAD was not dosed, despite the benign safety profile, because of the prolonged period of residual drug levels noted in the previous groups of healthy subjects.

Subjects in Groups 4 and 5 of the SAD study and Groups 1 and 2 of the MAD study who received zegocractin were followed for 1 year in a long-term extension study to assess for AEs and SAEs. In addition, PK levels were drawn in all 4 groups on Day 270 to further characterize the terminal phase and the prolonged period of minimal residual drug level. There were no SAEs and no adverse events rated moderate or severe in intensity in subjects followed for 365 days.

A population PK (popPK) model was built using the data from the SAD and MAD studies. The model suggested three compartments for distribution as well as gender and body weight-dependent differences in exposures. The model showed that females have a higher volume of distribution compared to males, resulting in lower plasma AUC_{24h} values versus males, and that patients with higher body weights will have a lower AUC. The model was then used to identify the dosing regimens for the first and second phases of the open-label study described below.

1.5.2 Open Label Study in Patients with AP and SIRS

CalciMedica has conducted a Phase 2a, open-label, dose-response, multi-center study of Auxora in patients with AP and accompanying SIRS and hypoxemia (CM4620-201). One patient was randomized having SIRS alone at Screening. The primary objective of the study was to evaluate safety and tolerability; the secondary objective was to evaluate efficacy and the PK profile of Auxora.

The study consisted of 2 phases; the Initial Phase consisted of 2 concurrently enrolled cohorts and the Second Phase consisted of 2 concurrently enrolled cohorts. In total, it was planned to have 4 Cohorts containing 24 adult male and female patients with AP and accompanying SIRS and hypoxemia. In the Initial Phase, 4 female patients were to be randomized in a 3:1 ratio to receive Auxora + Supportive Care (SC) or SC alone (Cohort 1). Concurrently, 4 male patients were to be randomized in a 3:1 ratio to receive Auxora + SC or SC alone (Cohort 2). Doses were to be 1.0 mg/kg on Day1 and 1.4 mg/kg daily on Days 2, 3 and 4 (low dose regimen). In the Second Phase, 8 female patients were to be randomized in a 3:1 ratio to receive Auxora + SC or SC alone (Cohort 3). Concurrently, 8 male patients were to be randomized in a 3:1 ratio to receive Auxora + SC or SC alone (Cohort 4). Planned doses for both Cohorts 3 and 4 were to be 2.08 mg/kg daily on Days 1 and 2 and 1.6 mg/kg daily on Days 3 and 4 (high dose regimen).

The decision to start Cohort 3 in the Second Phase was made after CalciMedica reviewed the available efficacy, safety and tolerability data from Cohort 1 and discussed this with the Principal

Investigator (PI). At this point, a decision was made to administer patients in Cohort 3 with the same dose level and schedule as in Cohort 1, as efficacy was observed in Cohort 1. Cohort 3, therefore, received the same dose level and schedule as Cohort 1, 1.0 mg.kg on Day1 and 1.4 mg/kg daily on Days 2, 3 and 4. The decision to start Cohort 4 in the Second Phase of the study was made after CalciMedica reviewed the available efficacy, safety and tolerability data from Cohort 2 and discussed this with the PI. Cohort 4 received 2.08 mg/kg daily on Days 1 and 2 and 1.6 mg/kg daily on Days 3 and 4 as planned.

The first infusion of Auxora was started within 6 (up to 8) hours of the patient or legal authorized representative (LAR) providing informed consent and was administered as a continuous IV infusion over 4 hours. Subsequent infusions were to be started every 24 hours (± 1 hour) from the start of the first infusion. In patients receiving Auxora+SC (all doses), there were 9 patients of 14 patients (64%) who did not receive all 4 scheduled doses, 7 of 9 patients because of rapid clinical improvement leading to early discharge and 2 of 9 patients because of study drug discontinuation. Five of 8 patients (63%) receiving the low dose regimen+SC and 4 of 6 patients (67%) receiving the high dose regimen+SC did not receive all 4 doses of CM4620-IE.

The demographic information and baseline characteristics for the patients enrolled in the study are noted in Table 3.

Table 3. Demographics and Baseline Characteristics of Patients in CM4620-201

Treatment	Auxora+SC<br>low dose regimen<br>(N = 8)	Auxora+SC<br>high dose regimen<br>(N = 6)	Auxora+SC<br>TOTAL<br>(N = 14)	SC Alone<br><br>(N = 7)
**Median Age (years)<br>Min, Max**	55<br>26, 66	43.5<br>37, 55	50.5<br>26, 66	54<br>40, 72
**Gender, n%**	Female 5 (63%)<br>Male 3 (38%)	Female 0<br>Male 6 (100%)	Female 5 (36%)<br>Male 9 (64%	Female 4 (57%)<br>Male 3 (43%)
**Race, n%**	Asian 1 (13%)<br>Black 1 (13%)<br>White 6 (75%)	Asian 0<br>Black 2 (33%)<br>White 4 (67%)	Asian 1 (7%)<br>Black 3 (21%)<br>White 10 (71%)	Asian 0<br>Black 3 (43%)<br>White 4 (57%)
**Median Weight (kg)<br>Min, Max**	86<br>56.2, 108.9	92.8<br>84.8, 113.8	87.5<br>56.2, 113.8	93.1<br>59, 108.9
**BMI (kg/m^2)<br>Min, Max**	31.6<br>22, 44.4	28.9<br>25, 38.2	30.3<br>22, 44.4	34<br>23.8, 41.6
**Hx Type 2<br>Diabetes Mellitus**	2 (25%)	1 (17%)	3 (21%)	1 (14%)
**Hx Hypertension**	4 (50%)	4 (67%)	8 (57%)	6 (86%)

n = number; SC = Supportive Care

The primary objective of this study was to assess the safety and tolerability of Auxora in patients with AP and accompanying SIRS and Hypoxemia. In this study, the low dose regimen+SC and

the high dose regimen+SC were well tolerated in patients with AP and SIRS, with no evidence of untoward safety or tolerability findings.

Treatment-emergent AEs (TEAEs) were reported in 7 of 8 patients (88%) receiving the low dose regimen+SC, 5 of 6 patients (83%) receiving the high dose regimen+SC, and 3 of 7 patients (43%) receiving SC alone. Severe TEAEs were reported in 0 of 8 (0%) patients receiving the low dose regimen+SC, 2 of 6 (33%) receiving the high dose regimen+SC, and 2 of 7 (29%) receiving SC alone. There were 3 TEAEs in 2 patients leading to discontinuation of the study drug. Both patients received the high dose regimen+SC.

Two different TEAE preferred terms (PTs) were reported in 2 or more patients receiving the low dose regimen+SC: Hypokalemia in 2 of 8 patients (25%) and Headache in 2 of 8 patients (25%). Three different TEAE PTs were reported in 2 or more patients receiving the high dose regimen+SC: Malnutrition, Confusional State and Acute Respiratory Distress Syndrome were each reported in 2 of 6 patients (33%). There were no TEAE PTs reported in 2 or more patients receiving SC alone.

There was 1 TEAE of Chromaturia in a patient receiving the high dose regimen+SC for which the causality was considered Possible. There were no other TEAEs, for which the causality was considered Possible, Probable or Definite.

SAEs were reported in 2 of 8 patients (25%) receiving the low dose regimen+SC, 1 of 6 patients (17%) receiving the high dose regimen+SC and 2 of 7 patients (29%) receiving SC alone. There was 1 death during the study. This patient, who received the high dose regimen+SC, experienced an SAE of Hypoxic-Ischemic Encephalopathy for which the outcome was fatal. The SAE was considered severe, and the outcome was designated recovered/resolved with sequelae. Causality was considered to be unrelated.

There were no untoward changes in vital signs, oxygenation, and laboratory values associated with treatment with either the low dose or high dose regimen of Auxora.

The secondary objective of the study was to evaluate efficacy. Treatment with Auxora (all doses) resulted in more rapid restoration of gut function, less persistent SIRS, a greater percentage of patients with a reduction in the severity of AP by CTSI scoring, and a decreased length of hospital stay compared to SC alone.

At 72 hours or Discharge (if earlier), 7 of 14 (50%) of patients receiving Auxora+SC (all doses) were able to tolerate solid food, defined as eating ≥ 50% of a solid meal without vomiting or an increase in pain, compared to 1 of 7 (14%) patients receiving SC alone. At Day 10 (216 hours or Discharge), 13 of 14 (92.9%) patients receiving Auxora+SC (all doses) were able to tolerate solid food compared to 3 of 7 (43%) patients receiving SC alone. The median (min, max) time to tolerating solid food for patients receiving the low dose regimen+SC was 57 hours (17, 121), for patients receiving the high dose regimen, 78.5 hours (39, 440) and for patients receiving only SC, 100 hours (22, 720).

Five of 14 patients (35.7%) randomized to Auxora+SC (all doses) had persistent SIRS, defined as SIRS lasting continuously for ≥ 48 hours starting on or after the Day 1 (0 hour) assessment, compared to 5 of 7 patients (71.4%) in the SC alone group.

A blinded central reader read all Screening, Day 5 or Discharge, and Unscheduled contrast-enhanced computed tomography (CECT) scans and gave a score for the severity of AP using the CTSI scoring system. One patient treated with the high dose regimen+SC did not receive a CTSI score at either the Screening or Day 5 or Discharge CECTs. One patient treated with SC alone did not receive a CTSI score at Screening because contrast was not given. On the Screening CECT, 8 patients treated with Auxora-IE+SC (all doses) had moderate or severe AP based on the CTSI score. Three of the 8 (37.5%) patients with moderate or severe AP improved with treatment to have mild AP based on the CTSI score on the Day 5 or Discharge CECT. On the Screening CECT, 4 patients treated with SC alone had moderate or severe AP. None of the 4 (0%) patients improved to have mild AP by the Day 5 or Discharge CECT.

The median length of stay (min, max) for patients randomized to Auxora-IE+SC (all doses) and SC alone were 3.70 (1.5, 18.3) and 6.02 (1.1, 30.0) days, respectively.

1.5.3 Pharmacodynamic and Pharmacokinetic Study in Patients with AP

In Study CM4620-202, A Pharmacodynamic and Pharmacokinetic Study of Auxora in Patients with Acute Pancreatitis, patients with AP (regardless of the presence of SIRS and/or hypoxemia) were administered a single IV infusion of 2.08 mg/kg Auxora and blood, plasma and serum were collected for analysis. It was planned to initially enroll 5 patients and then to enroll an additional 4 patients as needed. Ultimately, 7 patients were screened for the study, and all 7 enrolled in and completed the study. On Days 1 and 2, blood and plasma samples for PD and PK analyses, respectively, were obtained 30 minutes after completing the administration of Auxora and 24 hours from the start of the administration of Auxora. In patients hospitalized at Day 5 and 10, blood and plasma samples were obtained; if discharged earlier, samples were obtained at the time of discharge. After discharge, patients returned to the hospital on Day 30 to provide final blood and plasma samples.

Of the 7 patients, 5 (71%) were male and 2 (29%) were female. The median (min, max) age in all 7 patients was 42 (29, 54) years. The age range was 38 to 54 years in males and 29 to 35 years in females. The weight range was 49.4 to 102.1 kg and the BMI range was 19.3 to 32.2. Of the 7 patients, 4 (57%) were black or African and 3 (43%) were white. There were no (0%) Hispanic or Latino patients enrolled in the study. The cause of AP was alcohol in 5 of the 7 patients, hypertriglyceridemia in 1 of the 7, and unknown in the other.

A total of 3 patients experienced 7 TEAEs during the study. One (1) patient experienced a TEAE of Melena and a TEAE of bursitis, 1 patient had a TEAE of Pancreatitis Acute (which was also an SAE), and 1 patient experienced TEAEs of Pneumonia, Alcohol Withdrawal Syndrome, Pyrexia and Respiratory Distress (which was also an SAE). Of the 7 TEAEs, there were 3 mild, 2 moderate and 2 severe TEAEs. The 2 severe TEAEs (Pancreatitis Acute and Respiratory Distress) were also SAEs. The causality of the 7 TEAEs to Auxora was Unrelated for 5 TEAEs and Unlikely for 2 TEAEs.

The results of the study showed a rapid decrease in IL-2 production by stimulated white blood cells with infusion of Auxora with rapid return after discontinuation (Figure 3). The study suggested that there would be no long-term immunomodulatory effects from the administration of Auxora.

Figure 3. Stimulated IL-2 production by WBCs in response to administration of Auxora

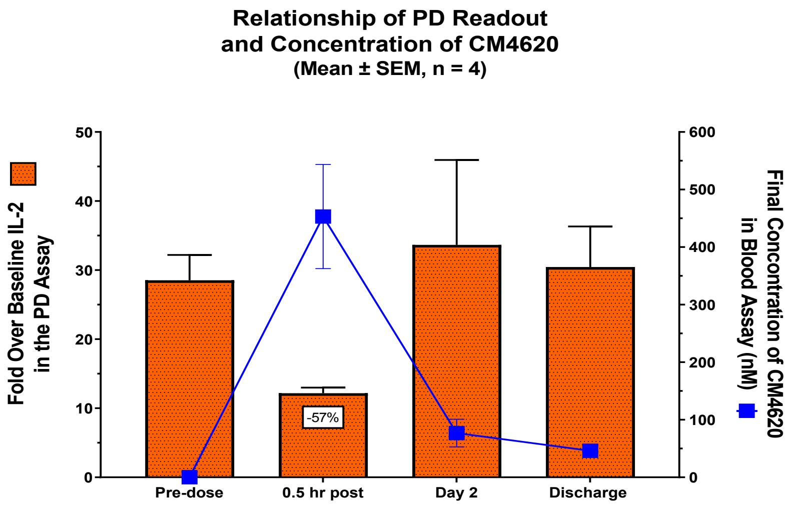

PD = pharmacodynamic; WBC = white blood cell

1.6 Rationale for the Study and Selected Doses

Auxora has been well tolerated in healthy volunteers and in patients with AP, SIRS, and hypoxemia with no untoward safety findings. In addition, an array of efficacy signals was noted in the open label study. The safety of the compound and the noted efficacy signals warrant continued development of Auxora for patients with AP given that there no approved products for the treatment of the disease. A randomized, double blind, placebo controlled, study would establish a clear proof of concept in patients with AP and SIRS, provide dose ranging data to support Phase 3 dosing, and evaluate a number of efficacy endpoints to inform Phase 3 endpoints.

The dosing regimen for the current study includes three consecutive days of dosing at 0.5, 1.0 or 2.0 mg/kg, each administered by 4-hour IV infusion The infusion duration and doses of 1.0 and 2.0 mg/kg were selected based on the results from previous Phase 1 and 2a studies that suggested these doses would have beneficial effects in patients with AP and SIRS. The 0.5 mg/kg dose was included to determine if this dose could define the minimally effective dose of Auxora.

From Phase 2a study CM4620-201, early tolerance of solid food was displayed in patients assigned to both the low and high dose regimens of Auxora (low dose regimen: 1.0 mg/kg on Day 1 and 1.4 mg/kg on Days 2-4; high dose regimen: 2.08 mg/kg on Days 1-2, 1.6 mg/kg on Days 3-4), although not all patients received the full 4 days of dosing because of early discharge. Additionally, no patients (0/6) in the high dose regimen group showed a worsening in their

Balthazar scores, indicative of increased pancreatic inflammation, whereas 2/8 patients (25%) in the low dose regimen group showed an increase in their scores. These data suggest that doses or systemic exposures associated with the high dose regimen provide the greatest opportunity for efficacy with acceptable tolerability. Since 75% of patients receiving the low dose regimen showed no worsening in their Balthazar scores, these doses may also be efficacious, albeit slightly less than those in the high dose regimen. The specific dose levels of 2.0 and 1.0 mg/kg were selected for the current study to closely match the Day 1 doses of the high and low dose regimens in the Phase 2a study, respectively. Doses notably higher than 2.0 mg/kg (1.25 mL/kg) were not selected as they carry increased risk of intolerance to either zegocractin or lipid load from the emulsion vehicle. The third dose level of 0.5 mg/kg was chosen to help define a minimum efficacious dose level. Adjustment of the zegocractin dose for body weight is based on covariate analysis from a popPK model that was developed from previous Phase 1 and Phase 2a studies. Dose adjustment is not being made for gender or race based on the absence of substantial covariate effects on the popPK model. The 3 dose levels selected are expected to achieve significant separation of plasma exposures on Day 1 (C_{max} and AUC_{0-24}) based on popPK modeling. Furthermore, 3 dose levels are considered adequate for dose-ranging studies, as described in International Conference on Harmonization (ICH) guidance E4.

The choice of the lower dose level (0.5 mg/kg) is also supported by non-clinical and clinical PK/PD data. Studies in mouse and rat models of AP indicated that achieving a C_{max} blood level of CM4620 *in vivo* close to or greater than the IC_{50} value in an *in vitro* whole blood assay of T lymphocyte function predicted efficacy in the animal model. Consistent with this idea, data from a small PK/PD study in AP patients (CM4620-202) showed that T lymphocyte function in an *ex vivo* whole blood assay was inhibited greater than 50% after a single dose of 2.08 mg/kg CM4620-IE, a dose level linked to signs of efficacy in the open-label study (CM4620-201). Analysis and modeling of clinical PK in patients receiving CM4620-IE indicates that C_{max} values of CM4620 after dosing at 0.5 mg/kg would fall close to the range of the IC_{50} value in an *in vitro* human whole blood assay of T lymphocyte function. Thus, if the animal models are predictive of human disease, the dose of 0.5 mg/kg CM4620-IE should mark the lower limit of the efficacious range.

The dosing regimen for the current study includes 3 consecutive days of dosing at the same dose level. The derivation of this regimen comes from both the Phase 1 MAD and Phase 2a studies, in that the dosing regimens for the Phase 2a open-label study were chosen based on maximum systemic exposures observed in the Phase 1 MAD study that were well tolerated. Thus, the low dose regimen in Phase 2a study CM4620-201 (1.0 mg/kg on Day 1, 1.4 mg/kg on Days 2-4) was designed to achieve plasma exposures on Day 4 (AUC_{0-24}) equivalent to those achieved on Day 7 from the high dose group in the MAD study that received 7 daily doses of 1.0 mg/kg. Over an abundance of caution, the high dose regimen in the Phase 2a study (2.08 mg/kg on Days 1-2, 1.6 mg/kg on Days 3-4) was designed to achieve plasma exposures on Day 4 that were only 30% higher than those achieved with the low dose regimen. Using this approach, no drug-related AEs were noted in the Phase 2a study. Although well-conceived for safety reasons, these forced titration regimens complicated the interpretation of PK exposures. Therefore, simplified repeat fixed-dose regimens are being utilized in the current study, whereby the same dose level is administered on three consecutive days. Three days of dosing was chosen because it matches the

median number of doses administered in the Phase 2a study, where evidence of efficacy signals was established.

2 OBJECTIVES AND ENDPOINTS

2.1 Primary Objectives

- To assess the dose response and efficacy of three different dose levels of Auxora in patients with AP and accompanying SIRS;
- To assess the time to medically indicated discharge in patients who are responders to early tolerance of solid food intake versus non-responders.

2.2 Secondary Objective

- To assess the safety and tolerability of varying doses of Auxora in patients with AP and accompanying SIRS.

2.3 Efficacy Endpoints

- Primary endpoint
  - Time to solid food tolerance
- Secondary endpoints:
  - Solid food tolerance at 48 hours, 72 hours, and 96 hours after the Start of First Infusion of Study Drug (SFISD) and at discharge
  - Time to medically indicated discharge
  - Length of stay in the hospital
  - Length of stay in the ICU for patients admitted to the ICU
  - Re-hospitalization for AP by Day 30
  - Change in severity of AP by CTSI score from screening to Day 30
  - Development of pancreatic necrosis ≥30% and >50%
  - The persistence of SIRS ≥48 hours after the SFISD
  - Incidence, severity, and duration of organ failure
  - Mortality by Day 30
  - Change in pain score and opioid use
- Exploratory endpoints:
  - Development of infected pancreatic necrosis
  - Development of sepsis
  - Hospital procedures for the management of pancreatic necrosis
  - Change in GCSI-DD score
  - Change in albumin
  - Change in absolute neutrophil count (ANC)/absolute lymphocyte count (ALC) ratio and IL-6 levels
  - Change in urine neutrophil gelatinase-associated lipocalin (NGAL) levels

2.4 Safety Endpoints

- The incidence of TEAEs and SAEs

- The intensity and relationship of TEAEs and SAEs
- Clinically significant changes in vital signs and safety laboratory results

3 INVESTIGATIONAL PLAN

3.1 Study Design

This double blind, randomized, placebo-controlled study will evaluate the efficacy, safety, and tolerability of three different dose levels of Auxora in patients with AP and accompanying SIRS.

Approximately 216 patients will be randomized 1:1:1:1 into one of 4 groups using a computer-generated randomization scheme accessed through an interactive voice/web response system (IXRS). Randomization will be first stratified by gender (male or female) and then by risk for organ failure in the gender subgroups (higher or lower). Higher risk for organ failure is defined by the presence of both an elevated hematocrit (HCT ≥44% for men or ≥40% for women) and hypoxemia (imputed $PaO_2/FiO_2 \leq 360$). Lower risk for organ failure is defined by the absence of either or both an elevated hematocrit and hypoxemia (Figure 4). The PaO_2/FiO_2 will be determined using an arterial blood gas or imputed using pulse oximetry (Appendix 5).

Figure 4. Higher versus Lower Risk of Organ Failure

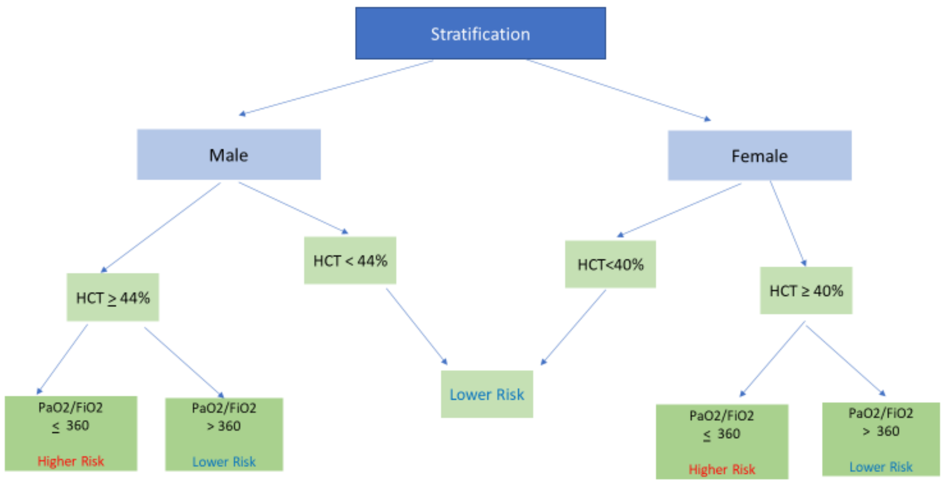

HCT = hematocrit

All patients will have received a Screening CECT of the abdomen/pancreas before being randomized into the study. CECTs performed as standard of care (SOC) may be used as the Screening CECT but must have been performed in the 24 hours before Consent or after Consent and before Randomization (Section 8.12.12).

The SFISD should occur within 8 hours of the patient or LAR providing informed consent. Patients randomized to Group 1 will receive 2.0 mg/kg of Auxora intravenously every 24 hours

(±1 hour) for a total of three doses. Patients randomized to Group 2 will receive 1.0 mg/kg of Auxora intravenously every 24 hours (±1 hour) for a total of three doses. Patients randomized to Group 3 will receive 0.5 mg/kg of Auxora intravenously every 24 hours (±1 hour) for a total of three doses. Patients randomized to Group 4 will receive emulsion without any active pharmaceutical ingredient. Patients in Group 4 will receive one of three randomly assigned dose volumes, 1.25 mL/kg, 0.625 mL/kg, or 0.3125 mL/kg, which will be administered intravenously every 24 hours (±1 hour) for a total of three doses. (Section 5.3) The dosing will be based on actual body weight obtained at the time of hospitalization or screening for the study. As described in the pharmacy manual, the upper limit of the volume of Auxora and volume of Placebo that will be administered will be 156.25 mL. The sponsor, investigators, pharmacists, and patients will be blinded to the assigned group. In the event of a medical emergency, investigators will be able to receive the treatment assignment if required to provide optimal care of the patient.

For all 4 groups, a study physician or appropriately trained delegate will perform study-specific assessments at screening, at the baseline assessment, immediately prior to the SFISD, and then every 24 hours until 240 hours after the SFISD, or until discharge if earlier. If patients remain hospitalized at Day 12, assessments will then be performed every 48 hours starting on Day 12 until Day 28, or until discharge if earlier. Patients discharged from the hospital before Day 25 will return at Day 30 (+5 days) to perform the Day 30 assessments. If patients are discharged on Days 25-29, the Day 30 assessments may be performed prior to discharge.

Patients will receive another CECT of the abdomen/pancreas at the Day 30 (±5 days) visit. All CECTs performed as SOC after randomization and before the Day 30 CECT will also be captured. A blinded central reader will read the Screening, Day 30, and any SOC CECTs obtained between randomization and the Day 30 visit.

Patients will complete the modified American Neurogastroenterology and Motility Society (ANMS) Gastrointestinal Cardinal Symptom Index Daily Diary (mGCSI-DD) worksheet at the baseline assessment, at 96 hours, 168 hours, Day 14 and Day 21 (for patients who remain hospitalized on these days), on the day of discharge, and daily at bedtime after discharge until the Day 30 visit. Patients who are discharged on Days 25-29 will not complete the mGCSI worksheet after discharge.

It is recommended that all patients randomized in the study should receive care consistent with the 2018 American Gastroenterological Association (AGA) Institute Technical Review of the Initial Medical Management of Acute Pancreatitis. Patients should receive local SOC for the management of other medical conditions.

In patients with AP, the AGA strongly recommends early oral feeding (within 24 hours) rather than keeping the patient nil per mouth (Nil per Os, NPO). Patients randomized into the study, therefore, will be offered a low fat, ≥500-calorie solid meal at each mealtime after the infusion of the first dose of study drug if alert and not on mechanical ventilation, or if not NPO for a planned surgery/medical procedure, or if not NPO because of an acute medical condition. If the patient does not wish to eat the solid meal when offered or is unable to tolerate the solid meal, they should then be offered a liquid meal. The same approach should occur at each subsequent mealtime until discharge. When patients eat a solid meal, it should be recorded if they ate ≥50% of the meal and

if they either vomited or experienced an increase in abdominal pain in the two hours after the meal.

It is also recommended that all patients randomized in the study should not be discharged from the hospital until solid food is tolerated, abdominal pain has resolved or been adequately controlled, and there is no clinical evidence of infection necessitating continued hospitalization. Tolerating solid food is defined as eating ≥50% of a low fat, ≥500-calorie solid meal without an increase in abdominal pain or vomiting. If the patient is not tolerating either solid or liquid meals, tube feedings should be considered.

All protocol required laboratory testing, except biomarker and PK samples, will be performed at the local laboratory. Results from the biomarkers and PK blood samples collected as part of the protocol and being tested at a central lab will not be available to assist the PI or treating physician in managing the patient.

CalciMedica may submit amendments to the protocol to modify the planned doses, the dosing schedule, the infusion time, the number of sites in the study, the total number of patients randomized in the study, the number of patients randomized in each group, and the number of groups in the study.

3.2 End of Study

The End of Study is considered the date on which the last patient randomized completes the Day 30 visit, unless CalciMedica terminates the study early.

3.3 Sponsor Termination of the Study

CalciMedica intends to complete the study as outlined. CalciMedica reserves the right, however, to terminate the study at any time because of:

- A recommendation from the Independent Data Monitoring Committee (IDMC)
- A directive from the U.S. Food and Drug Administration (FDA)
- A lack of enrollment

4 SELECTION OF PATIENTS

4.1 Inclusion Criteria

All of the following must be met for a patient to be randomized into the study:

1. The diagnosis of AP has been established by the presence of abdominal pain consistent with AP together with at least 1 of the following 2 criteria:
    a. Serum lipase > 3 times the upper limit of normal (ULN);
    b. Characteristic findings of AP on abdominal imaging;
2. The diagnosis of SIRS has been established by the presence of at least two of the following four criteria:
    a. Temperature < 36°C or > 38°C;
    b. Heart rate > 90 beats/minute;
    c. Respiratory rate >20 breaths/minute or arterial carbon dioxide tension ($PaCO_2$) <32 mmHg;
    d. White blood cell count (WBC) >12,000 mm^3, or <4,000 mm^3, or > 10% immature (band) forms;
3. At least one of the following criteria is present:
    a. A peripancreatic fluid collection or a pleural effusion on a CECT performed in the 24 hours before Consent or after Consent and before Randomization;
    b. Abdominal examination documenting either abdominal guarding or rebound tenderness;
    c. Hematocrit ≥44% for men or ≥40% for women;
4. The patient is ≥ 18 years of age;
5. Lack of pancreatic necrosis, pancreatic calcifications, pancreatic pseudocysts and no evidence for previous necrosectomy or pancreatic surgery identified by CECT performed in the 24 hours before Consent or after Consent and before Randomization.
6. A female patient of childbearing potential who is sexually active with a male partner is willing to practice acceptable methods of birth control for 180 days after the last dose of study drug. A female patient must not attempt to become pregnant for 180 days;
7. A male patient who is sexually active with a female partner of childbearing potential is willing to practice acceptable methods of birth control for 180 days after the last dose of study drug. A male patient must not donate sperm for 180 days;
8. The patient is willing and able to, or has a legal authorized representative (LAR) who is willing and able to, provide informed consent to participate, and to cooperate with all aspects of the protocol.

4.2 Exclusion Criteria

Patients with any of the following conditions or characteristics must be excluded from randomizing:

1. Expected survival <6 months;
2. Suspected presence of cholangitis in the judgment of the treating physician;
3. The patient has a known history of:
    a. Organ or hematologic transplant;
    b. HIV, hepatitis B, or hepatitis C infection;
    c. Chronic pancreatitis (Appendix 7);
4. Current treatment with:
    a. Chemotherapy;
    b. Immunosuppressive medications or immunotherapy (Section 5.4 for list of prohibited immunosuppressive medications and immunotherapy);
    c. Pancreatic enzyme replacement therapy;
    d. Hemodialysis or Peritoneal Dialysis;
5. The patient is known to be pregnant or is nursing;
6. The patient has participated in another study of an investigational drug or therapeutic medical device in the 30 days before randomization;
7. Allergy to eggs or known hypersensitivity to any components of study drug.

4.3 Re-Screening

A patient who fails the initial screening, may be rescreened once again (e.g. a reversible cause of screen failure has resolved and the subject meets all eligibility criteria).

5 TREATMENT OF PATIENTS

5.1 Overview

All patients randomized in the study should receive care consistent with the 2018 AGA Institute Technical Review of the Initial Medical Management of Acute Pancreatitis and local SOC for the management of other medical conditions. All protocol required laboratory testing, except biomarker and PK samples, will be performed at the local laboratory. Results from the biomarkers and PK blood samples collected as part of the protocol and being tested at a central lab will not be available to assist the PI or treating physician in managing the patient.

All CECTs performed as part of the study from Screening to the Day 30 visit will be read by a blinded central reader; these scans may also be read locally as necessary and used by the PI or treating physician for patient management.

5.2 Enhanced Recovery Strategy for Tolerance of Solid Food

Poor tolerance of solid food is proportional to severity in patients with AP, identified through an international Delphi process and validated in a study of 439 patients to be second to organ failure in the assessment of AP severity (Buxbaum 2018). Furthermore, as the Phase 2a study CM4620-201 showed a marked improvement in tolerance of solid food in association with CM4620 administration compared to placebo, this parameter will be measured as the primary efficacy endpoint. Patients randomized into the study will be offered a low fat, ≥500-calorie solid meal at each mealtime after the infusion of the first dose of study drug if alert and not on mechanical ventilation or if not NPO for a planned surgery/medical procedure, or if not NPO because of an acute medical condition. If the patient does not wish to eat the solid meal when offered or is unable to tolerate the solid meal, they should then be offered a liquid meal. The same approach should occur at each subsequent mealtime until discharge. When patients eat a solid meal, it should be recorded if they ate ≥50% of the meal and if they either vomited or experienced an increase in abdominal pain in the two hours after the meal. Tolerating solid food is defined by the consumption of ≥50% of a low fat, ≥500-calorie solid meal without an increase in abdominal pain or vomiting in the two hours after the meal. The time when the patient receives a solid meal that is then tolerated shall be recorded. If the patient is not tolerating either solid or liquid meals, tube feedings should be considered as per the AGA Institute Guideline on Initial Management of Acute Pancreatitis.

5.3 Discharge Criteria

The patient randomized to receive study drug should remain in the hospital until all 3 doses of study drug have been administered. The criteria for medically indicated discharge from the hospital are:

- Abdominal pain has resolved or is controlled with medications;
- Solid food is tolerated, which is defined as eating ≥50% of a low fat, ≥500 calorie solid meal without an increase in abdominal pain or vomiting;

- There is no clinical evidence of an infection necessitating continued hospitalization.

In the scenario where the PI or treating physician wants the infusion(s) to begin earlier than the 24 hour +/- 1 hour window (e.g. the patient is ready to be discharged before all 3 doses of study drug have been administered and / or to facilitate an infusion time where study personnel are available), the PI or treating physician should contact the medical monitor to discuss whether it is appropriate to begin infusion earlier (up to 6 hours earlier) than the 24 hour +/- 1 hour protocol window.

5.4 Prohibited Medications

Any medication, with the exception of those listed below, may be given at the discretion of the PI. Immunosuppressive medications/immunotherapy that should not be administered during the study include:

- Chemotherapy
- Cyclosporine, Tacrolimus
- Sirolimus, Everolimus
- Azathioprine
- Cyclosphosphamide
- Methotrexate
- Mycophenolate
- Leflunomide
- Biologics/Monoclonals: such as, but not limited to, abatacept, adalimumab, alemtuzumab, anakinra, basilizimab, belimumab, bevacizumab, brodalumab, canakinumab, certolizumab, cetuximab, clazakizumab, daclizumab, eculizumab, etanercept, golimumab, guselkumab, infliximab, interferon, ixekizumab, muromonab, natalizumab, omalizumab, rituximab, secukinumab, tocilizumab, trastuzumab, ustekinumab, vedolizumab,
- Baracitinib
- Tofacitinib

5.5 Compliance

Only the PI or his/her appropriately trained study staff will administer study drug to patients randomized in the trial in accordance with the protocol. Study drug must not be used for any reasons other than that described in the protocol.

6 PROCEDURES

6.1 Randomization Procedures

Approximately 216 patients will be randomized 1:1:1:1 into one of 4 groups using a computer-generated randomization scheme accessed through an IXRS. Randomization will be first stratified by gender (male or female) and then by risk for organ failure in the gender subgroups (higher or lower). High risker for organ failure is defined by the presence of both an elevated hematocrit (HCT ≥44% for men or ≥40% for women) and hypoxemia (imputed PaO_2/FiO_2 ≤360). Lower risk for organ failure is defined by the absence of either or both an elevated hematocrit and hypoxemia (Figure 4). The PaO_2/FiO_2 will be determined using an arterial blood gas or imputed using pulse oximetry (Appendix 5).

6.2 Discontinuation and Withdrawal

The term treatment discontinuation refers to a patient or PI discontinuing the administration of study drug before all 3 doses are administered despite the patient remaining in the hospital. Patients who do not receive all 3 doses because the treating physician discharged them from the hospital will not be considered as treatment discontinuation. Patients have the right to discontinue the administration of study drug at any time for any reason, without prejudice to their medical care. The PI may discontinue the administration of study drug because of an AE or change in medical status that raises a safety concern about the patient receiving additional doses of study drug. The PI **must** discontinue the administration of study drug if the patient is diagnosed with a new or recurrent malignancy, or if the patient was concomitantly administered a prohibited medication. If possible, the PI should contact the Medical Monitor to review the reasons for a patient's discontinuation from study drug. The PI should also record the reason for the treatment discontinuation in the electric case report form (eCRF) and appropriate source documents at the site. Even if the patient meets the definition of treatment discontinuation, patient should be encouraged to continue participation in the study for all regularly scheduled assessments, for safety and for efficacy.

Withdrawal refers only to the complete withdrawal of the patient from the study because of the withdrawal of consent. The PI should inform the Medical Monitor of the withdrawal of consent and record the withdrawal of consent in the eCRF and appropriate source documents at the site.

Patients will only be considered as lost to follow-up (LTF) after PI or designee documents they are unable to contact patient to complete final study assessment(s) or is unable to ascertain vital status (see Section 8.10).

7 STUDY DRUG MATERIALS AND MANAGEMENT

7.1 Auxora Product Description

Auxora is to be administered as an IV infusion and is supplied as a translucent, white to yellowish colored, sterile, non-pyrogenic emulsion containing 1.6 mg/mL of the active pharmaceutical ingredient zegocractin. Auxora is supplied as an 80 mL fill in a 100 mL, single-use glass vial. The drug product is formulated as an emulsion due to the low solubility of zegocractin in aqueous solution. Auxora contains egg phospholipids, medium chain triglycerides, glycerin, edetate disodium salt dehydrate (EDTA), sodium hydroxide (as needed to adjust pH), and sterile water for injection (Table 4).

Table 4. Auxora Product Information

**Product Name:**	Auxora
**Dosage Form:**	Injectable Emulsion (Liquid)
**Concentration**	1.6 mg/mL
**Route of Administration**	IV
**Physical Description**	Translucent, non-separated, white to yellowish emulsion
**Inactive Ingredients**	Sterile Water for Injection USP, Egg Phospholipid NF (80% Phosphatidylcholine), Medium Chain Triglycerides NF, Glycerin USP, and Edetate Disodium Salt Dihydrate (EDTA) USP. Sodium Hydroxide and Hydrochloric Acid may be added to adjust the pH.

IV = intravenous

7.2 Placebo Product Description

Matching Placebo is to be administered as an IV infusion and is supplied as a translucent, white to yellowish, sterile, non-pyrogenic emulsion carrier containing no active pharmaceutical ingredient. Placebo is supplied as an 80 mL fill in a 100 mL single-use vial. Placebo contains the same ingredients as Auxora except that it does not contain zegocractin.

7.3 Auxora and Placebo Storage

Auxora and Placebo must be maintained in a secure location with refrigerated temperature conditions of 2 to 8°C (36 to 46°F). Precaution should be taken to ensure that the Auxora and Placebo do not freeze. Temperature logs should be maintained and available at each monitoring or remote review visit. When a temperature is noted outside the range of 2°C to 8°C lasting for 24 hours or more, or if the temperature exceeds 20°C (68°F) or is below 0°C (32°F), CalciMedica or its designee must be notified. The stability of Auxora and Placebo has been demonstrated to 36 months when stored at 2 to 8°C.

7.4 Auxora and Placebo Preparation

The study pharmacist and/or designee will be responsible for the preparation and dispensation of Auxora and Placebo. Prior to administration, Auxora and Placebo must be transferred to a sterile container a using sterile technique. Specific details on how to calculate and prepare Auxora and Placebo, as well as the specific components that will be used to administer Auxora and Placebo, will be provided in a Pharmacy Manual. The Pharmacy Manual will also contain tables detailing the selected dose level, and the concentration of Auxora and Placebo.

7.5 Auxora and Placebo Administration

Both Auxora and Placebo will be administered intravenously over 4 hours at a constant rate of infusion. They will be administered every 24 hours (±1 hours) for three consecutive days for a total of 3 doses (Section 5.3). The dose and volume of Auxora, and the volume of Placebo, that will be administered will be calculated using the patient weight obtained at the time of hospitalization or during screening. A line into a peripheral or central vein may be used for the infusion. The peripheral IV should be 20 gauge in size or larger. If a smaller gauge IV is required, please contact the medical monitor. The peripheral IV or central line port should be dedicated when administering Auxora or Placebo other than 0.9% normal saline. Auxora and Placebo are compatible with 0.9% normal saline. The IV tubing used to administer Auxora and Placebo must contain a 1.2 micron filter. The Pharmacy Manual will contain a recommended procedure to prime the IV tubing and flush the tubing, but this may be adapted to local nursing standards. 0.9% normal saline may be used to clear the line to ensure that the volume to be infused (VTBI) is completely administered. If the administration of Auxora or Placebo is stopped because of a technical reason, such as failure of the IV site, or IV pump malfunction, the administration of Auxora or Placebo should be resumed when the technical reason is resolved, and continued at the same rate until the infusion is completed. The total amount of time for the start of infusion to end of infusion of Auxora or Placebo should be recorded.

CalciMedica may modify at any time the administered doses of Auxora or volumes of Placebo, the days of infusion, the timing of the infusion and the rate of infusion based on its review of the safety and tolerability data. CalciMedica will discuss with the PIs before implementation. For individual patients, if the administration of Auxora or Placebo is stopped because of a SAE that is considered to be probably or definitely related to Auxora or Placebo, the Medical Monitor must be immediately contacted.

7.6 Packaging and Labeling

Preparation, packaging and labeling of Auxora or Placebo will be in accordance with current Good Manufacturing Practice of Medicinal Products (GMP) guidelines. Medication labels will comply with legal requirements for labeling of investigational products in the United States as well as other country requirements, as applicable.

7.7 Accountability, Handling and Disposal

The PI or designee will ensure that deliveries of Auxora or Placebo from CalciMedica or its designee are received by a responsible person, and such deliveries are recorded; that Auxora or Placebo is handled and stored safely and properly; that Auxora or Placebo is only dispensed to study patients in accordance with the protocol; and that unused Auxora or Placebo is returned to CalciMedica or its designee or disposed of using standard procedures approved of in advance by CalciMedica or its designee. Appropriately trained study staff will administer all doses of Auxora or Placebo. The pharmacy will maintain a record of Auxora or Placebo accountability. The procedure for blinding the administration of study drug, and maintain the blind during the study, will be developed and adapted for all sites.

8 VISITS AND ASSESSMENTS

8.1 Screening

The PI or designee must provide informed consent to the patient, or LAR, allowing the patient or LAR adequate time to consider, ask questions and receive answers, prior to agreeing to participate.

- Record the time of the patient or LAR provides informed consent

After informed consent is obtained, the following procedures are to be performed unless already performed as part of SOC:

- Record the time of the onset of abdominal pain that sent patient to ER
- Record demographics and medical history
- Record list of medications being taken prior to hospitalization
- Record vital signs closest to the time of informed consent
- Perform an abdominal examination specifically assessing for guarding and rebound tenderness (see Section 8.12.3)
- Draw serum pregnancy test if the patient is a female of childbearing potential – analyze at local lab
    - If already performed as part of SOC, record results. Testing may be performed on blood drawn in the previous 12 hours prior to Consent or following consent but prior to randomization.
- Draw a blood sample for serum lipase–analyze at local lab
    - If already performed at local lab as part of SOC, record results. Testing may be performed on blood drawn in the previous 12 hours prior to Consent or following consent but prior to randomization.
- Draw a blood sample for CBC, differential, and platelets–analyze at local lab
    - If already performed at local lab as part of SOC, record results. Testing may be performed on blood drawn in the previous 12 hours prior to Consent or following consent but prior to randomization.
- Determine if SIRS is present (see Section 8.12.13)
- Obtain CECT of the abdomen/pancreas and determine if there is evidence of pleural effusion, peripancreatic fluid collection, pancreatic necrosis, pancreatic calcifications, pancreatic pseudocysts, and previous necrosectomy/previous pancreatic surgery are present on a CECT
    - If CECT already performed in the 24 hours prior to the patient or LAR providing informed consent, use results from that CECT (see Section 8.12.12)

If the patient satisfies all of the inclusion criteria and none of the exclusion criteria, immediately proceed to Baseline Assessment.

8.2 Baseline Assessment

Perform the following prior to randomization:

- Record height and weight
- Record SpO_2 and the FiO_2 (see Section 8.12.6) closest but prior to the time of randomization
- Record the patient's pain using the pain numeric rating scale (PNRS) scale closest but prior to the time of randomization
- Record the patient's symptoms using the mGCSI-DD worksheet closest but prior to the time of randomization
- Draw blood samples for Serum Procalcitonin and Serum Chemistries - analyze at local lab.
    - If already performed at local lab as SOC, record results. Testing may be performed on blood drawn in the previous 12 hours of randomization.
- Draw blood sample for Serum IL-6 – send to central lab. May be performed on blood drawn in the previous 12 hours of randomization
- Obtain urine sample for Urine NGAL - send to central lab. May be performed on urine collected in the previous 12 hours of randomization

8.3 Randomization Using IXRS System

8.4 Start of First Infusion of Study Drug (SFISD)

The SFISD should begin within 8 hours of the patient or LAR providing informed consent.

Perform the following procedures immediately prior to the SFISD:

- Record concomitant medications
- **Infuse the first dose of study drug**
    - Record the time of the SFISD
    - Record the time when the infusion is finished
    - Draw blood sample for PK as soon as possible after completion of the first infusion of study drug (within +2 hours)
- Begin Enhanced Recovery Strategy for Tolerance of Solid Food (see Section 5.2) and continue daily until patient discharge.

8.5 24 hours

24 hours from the SFISD:

- Record concomitant medications
- Record vital signs closest to the scheduled time of assessment
- Record the patient's pain using the PNRS closest to the scheduled time of assessment
- Record SpO_2 and the FiO_2 (see Section 8.12.6) closest to the scheduled time of assessment
- Determine if, and when the patient tolerated solid food in the previous 24 hours (see Section 5.2)
- Determine if the patient met the medically indicated discharge criteria in the previous 24 hours (see Section 5.3)
- Perform AE/SAE assessment
- **Prior to the infusion of the second dose of study drug:**
  - Draw blood samples for CBC, differential, platelets, Serum Procalcitonin and Serum Chemistries - analyze at local lab. Testing may be performed on blood drawn in the previous 12 hours.
  - Draw blood sample for Serum IL-6 – send to central lab. Testing may be performed on blood drawn in the previous 12 hours.
  - Draw blood sample for PK prior to the infusion of the second dose of study drug (within -30 min) - send to central lab.
  - Obtain urine sample for Urine NGAL-send to central lab. Testing may be performed on urine collected in the previous 12 hours.
- **Start the infusion of second dose of study drug 24 hours (±1 hour) from the SFISD (**see Section 5.3)
  - Record the time when the infusion starts and finishes

8.6 48 hours

48 hours from the SFISD:

- Record concomitant medications
- Record vital signs closest to the scheduled time of assessment
- Record the patient's pain using the PNRS closest to the scheduled time of assessment
- Record SpO_2 and the FiO_2 (see Section 8.12.6) closest to the scheduled time of assessment
- Determine if, and when the patient tolerated solid food in the previous 24 hours (see Section 5.2)

- Determine if the patient met the medically indicated discharge criteria in the previous 24 hours (see Section 5.3)
- Perform AE/SAE assessment
- **Prior to the infusion of the third dose of study drug:**
    - Draw blood samples for CBC, differential, platelets, Serum Procalcitonin and Serum Chemistries – analyze at local lab. Testing may be performed on blood drawn in the previous 12 hours.
    - Draw blood sample for Serum IL-6 – send to central lab. Testing may be performed on blood drawn in the previous 12 hours.
    - Obtain urine sample for Urine NGAL - send to central lab. Testing may be performed on urine collected in the previous 12 hours.
- **Start the infusion of third dose of study drug 48 hours (±1 hours) from the SFISD** (see Section 5.3)
    - Record the time that the infusion starts and finishes
- Assess whether patient discharge is upcoming and whether mGCSI-DD worksheets need to be dispensed per Section 8.12.10.

8.7 72 hours

For patients who remain hospitalized 72 hours from the SFISD:

- Record concomitant medications
- Record weight closest to the scheduled time of assessment
- Record vital signs closest to the scheduled time of assessment
- Record the patient's pain using the PNRS closest to the scheduled time of assessment
- Record SpO_2 and the FiO_2 (see Section 8.12.6) closest to the scheduled time of assessment
- Determine if, and when the patient tolerated solid food in the previous 24 hours (see Section 5.2)
- Determine if the patient met the medically indicated discharge criteria in the previous 24 hours (see Section 5.3)
- Perform AE/SAE assessment
- Draw blood samples for CBC, differential, platelets, Serum Procalcitonin and Serum Chemistries – analyze at local lab. Testing may be performed on blood drawn in the previous 12 hours or + 2 hours from assessment.
- Draw blood sample for Serum IL-6 – send to central lab. Testing may be performed on blood drawn in the previous 12 hours or + 2 hours from assessment.
- Draw blood sample for PK ± 2 hours – send to central lab

- Obtain urine sample for Urine NGAL - send to central lab. Testing may be performed on urine collected in the previous 12 hours or + 2 hours from assessment.
- Assess whether patient discharge is upcoming and whether mGCSI-DD worksheets need to be dispensed per Section 8.12.10.

8.8 96, 120, 144, 168, 192, 216, 240 hours (±4 hours)

For patients who remain hospitalized 96, 120, 144, 168, 192, 216, and 240 hours from the SFISD:

- Record concomitant medications
- Record vital signs closest to the scheduled time of assessment
- Record the patient's pain using the PNRS closest to the scheduled time of assessment
- Record SpO_2 and the FiO_2 (see Section 8.12.6) closest to the scheduled time of assessment
- Determine if, and when the patient tolerated solid food in the previous 24 hours (see Section 5.2)
- Determine if the patient met the medically indicated discharge criteria in the previous 24 hours (see Section 5.3)
- Perform AE/SAE assessment
- Assess whether patient discharge is upcoming and whether mGCSI-DD worksheets need to be dispensed per Section 8.12.10.
- If the patient remains hospitalized 96 hours from the SFISD, record the patient's symptoms using the mGCSI-DD worksheet
- If the patient remains hospitalized 168 hours (Day 7) from the SFISD, record the patient's symptoms using the mGCSI-DD worksheet

In addition, for patients who remain hospitalized 120, 168, and 216 hours from the SFISD:

- Draw blood samples for CBC, differential, platelets, Serum Procalcitonin, and Serum Chemistries – analyze at local lab. Testing may be performed on blood drawn in the previous 12 hours or + 4 hours from assessment.
- Draw blood sample for Serum IL-6 – send to central lab. Testing may be performed on blood drawn in the previous 12 hours or + 4 hours from assessment.
- Obtain sample for Urine NGAL - send to central lab. Testing may be performed on urine collected in the previous 12 hours or + 4 hours from assessment.

8.9 Days 12 to 28 (±4 hours)

For patients who remain hospitalized 12, 14, 16, 18, 20, 22, 24, 26, and 28 days from the SFISD:

- Determine if, and when the patient tolerated solid food in the previous 48 hours

- Determine if the patient met the medically indicated discharge criteria in the previous 48 hours (see Section 5.3)
- Perform AE/SAE assessment
- Assess whether patient discharge is upcoming and whether mGCSI-DD worksheets need to be dispensed per Section 8.12.10.
- If the patient remains hospitalized at Day 14 and Day 21, request the patient record their symptoms at bedtime using the mGCSI-DD worksheet (see Section 8.12.10).

8.10 Day 30 (±5 days)

Patients who had been discharged from the hospital before Day 25, should return on Day 30 for the following assessments:

- Record the investigator's opinion of etiology of the AP episode
- Record weight
- Perform CECT of the pancreas. May be performed in the 5 days before or after the visit.
- Collect mGCSI-DD worksheets from the patient
- Record concomitant medications
- Record vital signs
- Record the patient's pain using the PNRS
- Record SpO_2 and the FiO_2 (see Section 8.12.6)
- Perform AE/SAE assessment
- Draw blood samples for CBC, differential, platelets, Serum Procalcitonin, and Serum Chemistries – analyze at local lab
- Draw blood sample for Serum IL-6 – send to central lab
- Draw a blood sample for PK - send to central lab
- Obtain sample for Urine NGAL - send to central lab

For patients discharged from the hospital on Days 25-29, the Day 30 assessments may be performed on the Day of discharge.

- Patients who are discharged from the hospital on Days 25-29 will not complete the mGCSI-DD after discharge. They will only complete it on the day of discharge.

For patients who remain hospitalized on Day 30, the following additional assessment will be performed:

- Determine if, and when the patient tolerated solid food in the previous 48 hours
- Determine if the patient met the medically indicated discharge criteria in the previous 48 hours (see Section 5.3)

For patients that do not return for Day 30 visit and have not withdrawn consent, the Investigator or designee will continue to contact patient by phone to return for on-site visit up to eight weeks following visit date. Although an out of window visit assessment would be considered a protocol deviation, investigators should attempt to obtain as much as possible all of the Day 30 visit assessments, including the Day 30 CECT.

If patient is not reachable by phone after four weeks following the planned Day 30 visit, the Investigator or designee will send certified letter to last known address to request patient to contact Investigator to either schedule Day 30 visit or at least confirm patient is still alive by telephone. If patient does not respond to certified letter or if patient had withdrawn consent, the Investigator or designee will check public records (e.g., vital records search) to determine if the patient had died prior to the scheduled Day 30 visit or can be assumed alive for the purposes of study follow-up.

8.11 Discharge Date

The time and date of discharge will be recorded as well as if the patient meets criteria for medically indicated discharge. All patients will be trained to complete the mGCSI-DD and will complete the mGCSI-DD before leaving the hospital. If patients are discharged on Days 25-29, they may complete the Day 30 assessments on the day of discharge. Following discharge, the Investigator or designee should contact patient by telephone once per week to confirm diary completion and patient agrees to return for Day 30 visit.

If a patient is admitted to a hospital following discharge but prior to Day 30 visit, Investigator will report the reason for the hospitalization as per serious adverse event reporting guidelines (See Section 9.2 and Section 9.7). As the patient is still within the study period, investigators or designee should resume collection of study procedures as per Schedule of Events (See Appendix 8). If patient is not admitted to the hospital where the study is being conducted, investigators are asked to record any procedures (i.e., invasive mechanical ventilation) received while patient was admitted during the study period within an unscheduled procedures form in the eCRF.

8.12 Study Assessments

8.12.1 Demographics, Medical History and Medications

To the extent possible given the patient’s medical condition, a medical history including family history of pancreatitis and smoking history will be collected at Screening. Demographic data will be collected and will include self-reported race, ethnicity and gender. Patients must be specifically questioned if they have had previous episodes of AP and if they have chronic pancreatitis, diabetes mellitus, hypertension, coronary or cerebrovascular disease, and chronic obstructive pulmonary disease, as well as those conditions noted in the exclusion criteria. The extent of alcohol consumption will be collected. Selected medications that were being taken prior to coming to the emergency department will be collected as will, if available, a baseline serum creatinine.

8.12.2 Concomitant Medications

The name, dose and frequency of selected medications that were administered will be recorded. The time, dose, and route of administration of opioid pain medications that are actually administered will be recorded. Generic names should be used when possible.

8.12.3 Abdominal Examination

An abdominal examination will be performed and recorded at Screening to assess if the patient has abdominal guarding or rebound tenderness. For eligibility purposes, an abdominal examination completed as SOC within 12 hours of consent or following consent but prior to randomization may be used for Inclusion Criterion 3b. Guarding is the voluntary contraction or involuntary spasm of the abdominal wall musculature to avoid pain from palpation of the abdomen and/or protect underlying inflamed viscera. Rebound tenderness is the elicitation of tenderness by rapidly and smoothly removing the examining hand. In addition, the presence or absence of the following symptoms should also be recorded at screening: bruising in the subcutaneous fatty tissue around the umbilicus and bruising in the flanks.

8.12.4 Vital Signs

The patient's temperature, heart rate (beats per minute), systolic and diastolic blood pressures and respiratory rate (breaths per minute) closest to the time of assessment should be recorded. If intra-abdominal pressures are being monitored, the pressure closest to the time of assessment should be recorded.

8.12.5 Arterial Blood Gas

If an arterial blood gas (ABG) has been performed as supportive care, the time of the draw and following results will be recorded: the pH, PaO_2, $PaCO_2$, SaO_2 and FiO_2.

8.12.6 SpO_2/FiO_2

The SpO_2 will be determined using a hospital approved pulse oximeter. The PI or appropriately trained delegate should review the pulse oximetry waveform determining the SpO_2 to attest to its adequacy. The FiO_2 at the time of the SpO_2 measurement will be recorded. For patients with assisted breathing, the FiO_2 is read from the controlled oxygen source e.g., Venturi masks, ventilator and CPAP/BIPAP systems with calibrated oxygen blenders. For patients breathing unassisted i.e., room air, the FiO_2 is recorded as 0.21. If a patient is on an uncontrolled oxygen source, the following table provides the estimated FiO_2:

Table 5. Conversion of O_2 Flow to FiO_2

**Supplemental Oxygen L/min**	**Estimated FiO_2 (%)**		
	**Nasal Cannula**	**Face Mask**	**Face Mask with Reservoir**
Room Air	21		
1	24		
2	28		
3	32		
4	36		
5	40	40	
6	44	50	60
7		50	70
8		60	80
9		60	90
10		60	95

Adapted from Vincent 2009

8.12.7 Laboratory Analyses

At Screening, a lipase, CBC, differential, and platelets, will be performed at the local lab. For eligibility purposes, the results of the CBC collected within 24 hours of consent or following consent but prior to randomization will be used to determine the presence of SIRS (see Section 8.12.13) and a Hematocrit collected as SOC within 12 hours of consent or following consent but prior to randomization may be used for Inclusion Criterion 3c.

CBC with Differential and Platelets will be performed at 24, 48 and 72 hours, and if the patient remains hospitalized, at 120, 168, and 216 hours following SFISD as well as at Day 30 assessment. These samples will be sent to the local laboratory for analysis.

Serum Chemistries and Serum Procalcitonin will be performed at Baseline, 24, 48 and 72 hours, and if the patient remains hospitalized, at 120, 168, and 216 hours following SFISD as well as at Day 30 assessment. These samples will be sent to the local laboratory for analysis.

CBC results that should be reported in the eCRFs include absolute or percent neutrophil and lymphocyte counts. Serum chemistries reported in CRFs should include sodium, potassium, chloride, bicarbonate, blood urea nitrogen, creatinine, glucose, calcium, phosphorus, as well as magnesium, total protein, albumin, alkaline phosphatase, alanine transaminase (ALT), aspartate transaminase (AST), total bilirubin, lactate dehydrogenase (LDH), total cholesterol, triglycerides, and creatine kinase (CPK).

If a site is unable to perform any of the laboratory tests because it is not offered at the site, it will not be performed on patients randomized at the site.

Blood and urine samples for biomarker assessments (non-genetic testing) including, but not limited, to blood samples for IL-6 and urine samples for urine NGAL will be collected at baseline, 24, 48, and 72 hours, and if the patient remains hospitalized, at 120, 168, 216 hours following SFISD as well as at Day 30 assessment. A reference laboratory will analyze these blood and urine samples. The results of these assays will not be available to the PI or treating physician in managing the patient. It is the expectation that all sites participate in biomarker collection; however, if a site is unable to provide blood or urine samples for the biomarker analysis, this will be determined at study start and not considered a protocol deviation for that site. In addition, if a site is participating in biomarker collection but a patient does not want to provide biomarker samples this will be documented in the informed consent and not considered a protocol deviation.

8.12.8 PK analysis

Blood samples for PK analysis will be drawn as soon as possible after the completion of the first infusion of study drug (within +2 hours), prior to the start of second infusion of study drug (within -30 min), and at 72 hours (±2 hours) from the SFISD. If the patient is discharged after the third infusion of study drug and prior to 72 hours from the SFISD, the blood sample will be drawn prior to discharge. A PK sample will also be drawn at Day 30. The date and time that the sample for PK analysis is drawn shall be recorded. All samples for PK analysis should be stored at either -20°C or -80°C until shipment on dry ice to the central laboratory. As with the biomarkers, it is the expectation that all sites participate in the PK sample collection; however, if a site is unable to provide blood for the PK analysis, this will be determined at study start and not considered a protocol deviation for that site. In addition, if a site is participating in PK collection but a patient does not want to provide PK samples this will be documented in the informed consent and not considered a protocol deviation.

8.12.9 Enhanced Recovery Strategy for Tolerance of Solid Food

Patients randomized into the study will be offered a low fat, ≥500-calorie solid meal at each mealtime after the infusion of the first dose of study drug until discharge if alert and not on mechanical ventilation, or if not NPO for a planned surgery/medical procedure, or if not NPO because of an acute medical condition. See Section 5.2 for specific assessment procedures.

8.12.10 Modified ANMS GCSI-Daily Diary (mGCSI-DD)

The ANMS GCSI Daily Diary was designed to assess gastrointestinal symptoms associated with idiopathic and diabetic gastroparesis. It has been shown to be a reliable tool to evaluate dysmotility in patients with AP (Ma 2016). It will be used to assess gastrointestinal symptoms associated with AP in an exploratory manner. The mGCSI-DD has a 24-hour recall; thus, all items are designed to be self-administered on a daily basis.

The patient training for completing the daily diary is provided by the PI or appropriately trained delegate. Patients should understand their disorder: AP, where there is an abnormally delayed emptying of food from the stomach because of inflammation in the pancreas. The mGCSI-DD recording sheet and instructions are reviewed with the patient prior to the patient starting the recording of daily symptoms in the diary. The symptom severity response items are reviewed: "For each symptom listed below, please mark with an X the box that best describes the worst severity of each symptom during the past 24 hours. The next question asks you to record the number of times vomiting occurred in the last 24 hours. Please record the number of vomits (throwing up with food or liquid coming out) that occurred in the last 24 hours. Record zero, if you have not vomited during the past 24 hours. If you vomited, write down the number of all vomits. If you vomited once, record one. If you vomited three times during the day, record three. If you vomited three times, whether it was during the same trip to the bathroom or three separate trips, record three as the number of episodes of vomiting."

Patients discharged before Day 25 will complete the daily diary on the day of discharge, and then after discharge, daily at bedtime until the Day 30 visit.

8.12.11 Pain Numeric Rating Scale (PNRS)

In patients who are able to self-report their pain, the Pain Numeric Rating Scale (Appendix 4) will be used to grade the severity of the abdominal pain. It should be recorded if an opioid analgesic had been given in the 2 hours prior to the PNRS determination.

8.12.12 Contrast-Enhanced Computed Tomography (CECT) of the Abdomen/Pancreas

The recommended CECT protocol will consist of:

- A non-ionic contrast bolus of 100-150cc will be administered at 3-5cc/second
- Slice thicknesses will be 5 mm or less and will be obtained through the pancreas during the pancreatic phase (35 seconds after the start of the non-ionic contrast bolus) and through the entire abdomen during the portal venous phase (50 to 70 seconds after the start of the non-ionic contrast bolus) to visualize the pancreas and surrounding tissue

If CECT is not available (e.g. medical contraindication to contrast medium used in CECT) and another appropriate imaging is available (e.g. MRI, MRCP), please contact medical monitor to discuss whether local SOC imaging can be used for eligibility and Day 30 purposes.

CECT contrast protocol may be altered to local SOC. The lack of perfusion of the pancreas on the CECT will be exclusionary. If a CECT had already been performed more than 24 hours but less than 48 hours before Consent, and there is a contraindication to repeating it, the PI should discuss with the medical monitor if the CECT may be used for evaluating the inclusion and exclusion criteria for the study.

The PI or treating physician may also elect to delay the Day 30 CECT (±5 days) due to patient-specific safety considerations but must inform the Medical Monitor of the decision. The PI or treating physician may also elect not to perform the CECT because of futility considerations, e.g., the patient is unlikely to survive in the next 48 hours but must discuss the decision with the Medical Monitor.

The CECT may also be used to make the diagnosis of chronic pancreatitis. Patients with a diagnosis of chronic pancreatitis in the chart or who self-report chronic pancreatitis but are neither on enzyme replacement nor following up with a gastroenterologist as an outpatient, should be considered for the study after a careful review of a CECT (Appendix 7). If the findings in the mild-moderate or severe categories are not present on the CECT, please contact the medical monitor to discuss whether the patient would be appropriate for randomization if they meet the other inclusion and none of the exclusion criteria. The 'equivocal' criteria do not exclude patients from randomization.

The Screening and Day 30 CECTs will be read by a blinded central reader. They will also be read locally and may be used by the PI or treating physician, as needed, for patient management.

If the patient undergoes additional CECTs at the discretion of the PI or treating physician from randomization to the Day 30 visit, the date of the CECT will be recorded and a blinded central reader will read the images.

8.12.13 Systemic Inflammatory Response Syndrome (SIRS)

The physician, or appropriately trained designee will determine the presence of SIRS at Screening. The most extreme value for each criterion in the 24 hours before consent may be used. SIRS is defined as the presence of at least 2 of the 4 criteria:

- Temperature < 36°C or > 38°C
- Heart rate > 90 beats/minute
- Respiratory rate > 20 breaths/minute or arterial carbon dioxide tension ($PaCO_2$) < 32 mmHg
- White blood cell count (WBC) >12,000 mm3, or < 4,000 mm3, or > 10% immature (band) forms

9 ADVERSE EVENTS

9.1 Definition of Adverse Event

An adverse event (AE) is defined as any untoward medical event in a patient regardless of its causal relationship to study treatment. An AE can be any unfavorable and unintended sign (including any clinically significant medical test abnormality), symptom, or disease temporally associated with the use of study drug, whether or not it is considered related to study drug administration. Included in this definition is any newly occurring event or previous condition that has increased in severity or frequency since the administration of study drug.

A medical test abnormality (e.g., laboratory test value, vital sign recording, ECG finding, physical examination finding) will be considered clinically significant and consequently recorded as an AE only if it meets one of the following criteria:

- Induces clinical signs or symptoms
- Requires active intervention
- Requires interruption or discontinuation of study medication

9.2 Definition of Serious Adverse Event

A serious adverse event (SAE) is any AE occurring at any dose and regardless of causality that:

- Results in death
- Is life-threatening
- Requires hospitalization or prolongation of existing hospitalization
- Results in persistent or significant incapacity or substantial disruption of the ability to conduct normal life function
- Is a congenital anomaly or birth defect in an offspring of a patient receiving study drug
- Is an important medical event

The term “life-threatening” refers to an event in which the patient was at immediate risk of death at the time of the event. It does not refer to an event that hypothetically might have caused death if it were more severe.

Important medical events are those that may not need any of the criteria defined above; however, they may be considered serious when, based upon appropriate medical judgment, they may jeopardize the patient and may require medical or surgical intervention to prevent one of the other outcomes listed in the SAE definition.

Pregnancy is not considered an AE; however, information will be collected for any pregnancies that occur during study drug administration or the thirty days thereafter. Certain pregnancy outcomes will require submission as an SAE.

9.3 Eliciting Adverse Event Information

At every AE/SAE assessment, the patient must be asked a standard, non-directed question, such as, “how have you been feeling since your last visit?” to elicit any medically related changes in their well-being. In addition, the hospital chart and other documents relevant to patient safety must be reviewed when the patient is in the hospital.

9.4 Recording Adverse Events

Recording of AEs must begin after randomization. All conditions present before randomization, including untoward medical events during Screening, should be documented as medical history. Documentation shall continue until the patient dies, the patient withdraws consent, or the patient’s participation in the study ends. Information to be collected includes:

- Type of event
- Date of onset
- Date of resolution
- Investigator-specified relationship to study drug and assessment of severity
- Seriousness
- Any action taken

While an AE is ongoing, changes in the severity (e.g., worsening and improving) should be noted in the source documents, but when documenting the AE, only the total duration and the greatest severity should be recorded in the case report form. AEs characterized as intermittent require documentation of onset and duration.

All AEs reported or observed during the study must be followed to resolution. Or, if not fully resolved, until the condition has stabilized, the patient dies, withdraws consent, the site is closed, or CalciMedica ends the trial, whichever is first.

AEs resulting from concurrent illnesses, reactions to concurrent medications, or progression of disease states must also be reported. Pre-existing conditions (present before the start of the AE collection period) are considered concurrent medical conditions and should NOT be recorded as AEs. However, if the patient experiences a worsening or complication of such a concurrent condition, the worsening or complication should be recorded as an AE. Investigators should ensure that the AE term recorded captures the change in condition (e.g., “worsening of…”).

Each AE should be recorded to represent a single diagnosis. Accompanying signs (including abnormal laboratory test values or ECG findings) or symptoms should NOT be recorded as additional AEs. If a diagnosis is unknown, sign(s) or symptom(s) should be recorded as an AE(s).

Elective procedures (surgeries or therapies) performed to manage/treat conditions that existed prior to the patient enrolling in the trial should not be recorded as AEs but should be documented in the patient’s source documents. If a planned procedure is performed early (e.g., as an emergency) because the pre-existing condition worsens, the worsening condition should be captured as an AE.

9.5 Assessment of Relationship to Study Drug

The Investigator must use the following classification and criteria to characterize the relationship or association of study drug in causing or contributing to the AE:

- **Unrelated**: This relationship suggests that there is no association between study drug and the reported event
- **Unlikely**: This relationship suggests that there is an unlikely association between study drug and the reported event
- **Possible**: This relationship suggests that treatment with study drug caused or contributed to the AE. That is, the event follows a reasonable temporal sequence from the time of study drug administration, and/or follows a known response pattern to the study drug, but could have been produced by other factors
- **Probable**: This relationship suggests that a reasonable temporal sequence of the event with study drug administration exists and, based upon the known pharmacological action of the drug, known or previously reported adverse reactions to the drug or class of drugs, or judgment based on the Investigator's clinical experience, the association of the event with study drug administration seems likely
- **Definite**: This relationship suggests a definite causal relationship exists between study drug administration and the AE, and other conditions (concurrent illness, progression/expression of disease state, or concurrent medication reaction) do not appear to explain the event

9.6 Assessment of Severity

The Investigator must use the following criteria to rate the intensity of the AE:

- **Mild**: Symptoms causing no or minimal interference with usual social and functional activities
- **Moderate**: Symptoms causing greater than minimal interference with usual social and functional activities
- **Severe**: Symptoms causing inability to perform usual social and functional activities

9.7 Reporting of Serious Adverse Events

The Investigator is responsible for reporting to CalciMedica or designee within 24 hours from the time when site personnel learn about the event, all SAEs that are observed or reported by the patient during the study (from randomization until the patient dies, the patient withdraws consent, or the patient's participation in the study ends) regardless of the relationship to study drug or clinical significance. Any additional information that becomes available later should be submitted within 1 working day of receipt. All SAEs reported or observed during the study must be followed to resolution or until the Investigator deems the event to be chronic or the patient to be stable. CalciMedica or its designee may contact the Investigator to obtain additional information on any SAE that has not resolved at the time the patient completes the study. SAEs ongoing at database lock will be noted as such. The PIs are also responsible for informing their IRB/EC of any SAEs at their site. SAE correspondence with IRBs/ECs must be submitted to CalciMedica or its designee for filing.

A study manual will contain the details needed to report SAEs. If any questions on SAEs, contact information is as follows:

- **SAE reporting email address**: CalciMedicaSafety@safety-sphere.com
- **SAE phone number**: 844-965-1070 (toll free)
- **SAE Fax number**: 1 (833) 292-6393

CalciMedica will notify the FDA in a written safety report of any suspected adverse reaction or adverse reaction associated with the use of Auxora that is serious and unexpected.

- Suspected adverse reaction means any AE for which there is a reasonable possibility that the drug caused the AE. For the purposes of IND safety reporting, 'reasonable possibility' means CalciMedica determines that there is evidence to suggest a causal relationship between the drug and the AE (definite, probable, possible) regardless of the investigator's causality assessment
- Adverse reaction means any AE caused by a drug. Adverse reactions are a subset of all suspected adverse reactions where there is reason to conclude that the drug caused the event
- Serious, as defined in Section 9.2
- Unexpected AE or suspected adverse reaction refers to an event or reaction that is not listed in the investigator's brochure or is not listed at the specificity or severity that has been observed

CalciMedica or its designee will notify the FDA of any unexpected serious suspected adverse reactions associated with the use of Auxora that are fatal or life threatening as soon as possible but no later than seven calendar days after the initial receipt of the information. Initial notification will be followed by a written report within fifteen calendar days.

CalciMedica or its designee will notify the FDA of any unexpected serious suspected adverse reactions associated with the use of Auxora that are not fatal or life threatening fifteen calendar days.

CalciMedica or its designee will provide copies of any reports to regulatory agencies regarding unexpected serious suspected adverse reactions associated with the use of Auxora to the Investigators for review and submission to the IRB/EC.

In addition, if any of the events below, which are commonly observed and thus 'anticipated' in this patient population, are reported as a SAE, they will not be reported to the regulatory authority as individual expedited reports except in unusual circumstances or if required by local regulations:

- Hypoxemia and acute respiratory distress syndrome
- Oliguria and acute kidney injury
- Hypotension and shock
- Bacteremia
- Pneumonia
- Obtundation
- Splanchnic venous thrombosis
- Abdominal compartment syndrome
- Gastroparesis and ileus
- Local complications of the pancreas and peripancreatic tissue
- Percutaneous insertion of abdominal drains
- Endoscopic necrosectomy
- ERCP
- Abdominal surgery including but not limited to cholecystectomy, pancreatic necrosectomy, and placement of pancreatic drains

9.8 Suspected Pregnancy in a Woman of Childbearing Potential

A female patient of childbearing potential is a female who is not surgically sterile (no history of a bilateral salpingo-oophorectomy) and is not postmenopausal for at least 1 year.

A female patient of childbearing potential who receives study drug and is sexually active with a male partner, and a male patient who receives study drug and is sexually active with a female of childbearing potential, must be willing to use two highly effective methods of contraception (e.g., barrier methods, spermicidals, intrauterine devices, and/or hormonal contraception) for

180 days after last dose of study drug. No contraception is required if a female patient or partner has undergone a bilateral salpingo-oophorectomy.

Two of the following methods of birth control must be practiced unless a sexually active female patient or partner of childbearing potential has undergone a bilateral salpingo-oophorectomy:

- Male partner has a vasectomy for at least six months duration
- Use of an intrauterine device
- Use of hormonal contraceptives (oral, parenteral, vaginal or transdermal)
- Double barrier contraception with the male partner using a condom and the female using a contraceptive sponge, spermicidal jelly or cream or diaphragm plus spermicidal jelly or cream

The Investigator should be immediately informed if a female patient or partner of childbearing potential suspects she is pregnant up to 180 days after last dose of study drug. If the female patient is receiving study drug when discovered to be pregnant, the study drug should be immediately discontinued. If a pregnancy is confirmed, the Investigator must immediately report a pregnancy and record the event using a Pregnancy Report Form. Pregnancy is not considered an AE but the Investigator must follow a pregnant patient or partner. The Investigator must report follow-up information regarding the course of the pregnancy, including perinatal or neonatal outcome. Infants resulting from such pregnancies should be assessed for normality at birth and should be followed for 6 months to assess for development milestones. CalciMedica or its designee may contact the Investigator to request additional information throughout the course of the pregnancy.

The following pregnancy outcomes must be considered SAEs and will require additional reporting in the eCRF and on an SAE form:

- Congenital anomaly/birth defect
- Stillbirth
- Spontaneous miscarriage

10 STATISTICAL METHODS

10.1 General Considerations

Data summaries and listings will be generated using SAS version 9.3 or a more recent version (SAS Institute Inc., Cary, NC, USA).

The statistical analysis plan (SAP) and/or the clinical study report will provide additional details of the analysis, which may include details of missing and, if applicable, unused data, as well as additional sensitivity analyses of the primary and secondary variables. The clinical study report will describe deviations from the SAP, if any.

The statistical analysis approach will be descriptive, exploratory and inferential to determine the relationship between dose response and endpoints in identifying the optimal dose and primary endpoint(s); also, in identifying the priority order of sequential testing for secondary endpoints.

10.2 Sample Size

A sample size of 216 patients will be randomized into four groups on a 1:1:1:1 basis, resulting in 54 patients randomized into each group. This sample size will provide 86% power for testing the difference in two populations having a median length of time of 72 and 144 hours, respectively, to tolerating solid food based on a log-rank test. This sample size will provide 80% power with a two-sided alpha of 0.05 (using the chi-squared test) to detect a 45% response rate for tolerating solid food in a Auxora dose group versus a 20% response rate in the Placebo group in the 72 hours after completion of the first study drug infusion.

10.3 Study Assessments

Efficacy assessments will include:

- Enhanced Recovery Strategy for Tolerance of Solid Food
- Modified Gastrointestinal Cardinal Symptom Index Score
- CECT of the Pancreas
- PNRS score
- Organ Failure scores
- Vital sign measurements
- Laboratory measurements
- Concomitant medications

Safety assessments will include:

- Vital signs measurements
- Laboratory measurements
- Concomitant medications
- TEAEs and SAEs

PK assessments and analyses will include:

- Adding the plasma concentration data to the current population PK model to refine the model and covariate analysis
- Using the revised population PK model to determine if AP disease severity alters PK properties of CM4620
- Determining whether a relationship exists between plasma trough levels of CM4620 and patient response to Auxora (i.e., an exposure-response relationship)

10.4 Analysis Sets

10.4.1 Efficacy and Safety

The efficacy analyses will be performed on the Modified Intent-to-treat Population (MITT) consisting of all randomized patients who receive any amount of study drug.

As the sensitive analysis, the primary efficacy analyses will also be performed on the Per-Protocol Population (PP) consisting of all randomized patients who complete the study and have no major protocol violations.

The safety analyses will be performed on MITT data set.

10.4.2 Pharmacokinetics

The PK population will consist of all randomized patients who receive any amount of active study drug and who have at least one post baseline PK sample analyzed.

10.5 Disposition

The number and percentage (n, %) of patients enrolled, screen failed, treated, completed study, and discontinued (with reason) will be summarized. Sample size for each analysis population will be identified for each treatment group. All screened patients will be included in the disposition analysis.

10.6 Analysis of Demographic and Baseline Data

Patient demographic and baseline characteristics will be summarized by mean, standard deviation, median, minimum, and maximum for continuous variables; and by counts and percentages for categorical variables. Summaries will be provided separately for each treatment group.

10.7 Efficacy Analysis

The efficacy endpoints are the following:

Primary endpoint

- Time to solid food tolerance

Secondary endpoints

- Solid food tolerance at 48 hours, 72 hours, and 96 hours after the SFISD and at discharge
- Time to medically indicated discharge
- Length of stay in the hospital
- Length of stay in the ICU for patients admitted to the ICU
- Re-hospitalization for AP by Day 30
- Change in severity of AP by CTSI score from screening to Day 30
- Development of pancreatic necrosis ≥30% and >50%
- The persistence of SIRS ≥48 hours after the SFISD
- Incidence, severity, and duration of organ failure
- Mortality by Day 30
- Change in pain score and opioid use

Exploratory endpoints

- Development of infected pancreatic necrosis
- Development of sepsis
- Hospital procedures for the management of pancreatic necrosis
- Change in GCSI-DD score
- Change in albumin
- Change in ANC/ALC ratio and IL-6 levels
- Change in urine NGAL

Time to solid food tolerance is the number of hours from the date and time of the SFISD for the patient to the first date and time the patient receives a solid meal that is tolerated, which is defined as eating ≥50% of a low fat, ≥500-calorie solid meal without an increase in abdominal pain or vomiting in the two hours after completing the meal.

Time to solid food tolerance for patients who could not tolerate solid food during the study will be censored on the latest date and time of food tolerance assessment.

Time-to-event endpoints will be summarized using Kaplan-Meier analyses. The medians and quartiles of time-to-events will be summarized. Time to solid food tolerance will be compared between each treatment groups and the placebo group using the stratified log-rank test as the primary analysis. The tests will be at an alpha level of 0.05 without multiplicity adjustment. The stratification variables will include gender (male vs. female) and disease severity status (higher

risk for organ failure defined by the presence of both a HCT ≥44% for men, or ≥40% for women, and a PaO_2/FiO_2 ≤360 before randomization (Figure 4).

In addition, the hazard ratio (HR) and the 95% CI will be estimated using a Cox proportional-hazard model with treatment group as the independent variable and stratified by the same randomization stratification factors as used for the log-rank test.

Percentage of patients who tolerated solid food at 48, 72 and 96 hours from the SFISD will be summarized and plotted by treatment groups across time points. The solid food tolerance rate at 72 hours will be compared between the treatment groups and the placebo group using a Cochran-Mantel-Haenszel (CMH) test at an alpha level of 0.05 stratified by gender (male vs. female) and disease severity status (higher risk for organ failure defined by the presence of both a HCT ≥44% for men, or ≥40% for women, and a PaO_2/FiO_2 ≤360 before randomization (see Figure 4). The Mantel-Haenszel weighted difference in complete response (CR) rate between the treatment groups and the placebo group and its 2-sided 95% CI will be provided.

In addition, the odds ratio (OR) and the 95% CI will be estimated using a logistic regression model with treatment group as the independent variable and stratified by the same randomization stratification factors as used for the CMH test.

Time to medically indicated discharge is the number of days from the SFISD to the date of meeting discharge criteria in Section 5.3 and will be compared between each treatment group and the placebo group using the stratified log-rank test. patients who could tolerate solid food and who could not tolerate solid food at 48, 72, and 96 hours using Kaplan-Meier curve and log-rank test.

Other secondary and exploratory efficacy endpoints will be summarized with details described in the separate SAP.

10.8 Safety Analysis

Safety will be assessed by subject reported and Investigator observed AEs along with clinical laboratory tests (hematology and chemistries), and vital signs. Safety variables will be tabulated by treatment groups and presented for all treatment patients. Exposure to study treatments, reasons for discontinuation, deaths and causes of deaths will be tabulated. TEAEs are defined as events that first occurred or worsened after the first dose of study drug. TEAEs will be mapped to the appropriate SOC and PT according to the Medical Dictionary for Regulatory Activities (MedDRA).

Summaries will be provided for all AEs, AEs considered related to study treatment, SAEs, and related SAEs as follows:

- By maximum severity
- Incidence by SOC (by severity grade and overall)
- Incidence by PT (by severity grade and overall)

The percentage of 30 day all-cause mortality will be calculated based on each subject's date of death relative to first dose of study treatment and tabulated by treatment groups.

Laboratory test results will be used in the summary. The lower and upper reference range values for blood tests from the laboratories will be used to grade the lab results to low, normal and high. Shift tables will display (1) shift from baseline grade to the worst grade, and (2) shift from baseline grade to the last grade. Vital signs will be summarized by visit using proportion of patients with each vital sign being too high or too low according to conventionally accepted vital sign normal ranges.

Concomitant medication will be coded by the WHO Drug Dictionary and summarized by Therapeutic subgroup and PTs, using counts and percentages. Concomitant medications are the medications taken with a start date on or after the start of the administration of study treatment, or those with a start date before the start of the administration of study treatment and a stop date on or after the start of the administration of study treatment.

10.9 Independent Data Monitoring Committee

An IDMC will be convened and will monitor safety for this study on an ongoing basis. An IDMC charter will govern the IDMC and will describe the scope of responsibilities of the IDMC. If the IDMC recommends alteration of the dosing regimen because of safety issues, the FDA and other regulatory agencies will be notified as appropriate. The IDMC responsibilities include protecting the safety of the study patients and making recommendations to CalciMedica concerning the conduct of the study.

11 ADMINISTRATIVE CONSIDERATIONS

11.1 Electronic Case Report Forms

The study will use an Electronic Data Capture (EDC) system that is 21 CFR Part 11 compliant to electronically capture data for all screened and randomized patients.

11.2 Monitoring of the Study

The site monitor, as a representative of CalciMedica, will closely follow the conduct of the study. The site monitor will visit or remotely review the site's study activities periodically and maintain necessary telephone and letter contact with the PI and his/her study staff. The site monitor will maintain current knowledge of each site's study activity by observing the conduct of the study at the site, reviewing study records and source documentation, and discussing the conduct of the study with the PI and his/her study staff.

11.3 Inspection of Records

The PI, his/her study staff and the study site will provide direct access to all study records to assist study-related monitoring and audits, Institutional Review Board/Ethics Committee/Research Ethics Board (IRB/EC) reviews, and regulatory inspections. In the event of an audit, the PI agrees to allow CalciMedica or its representatives and relevant regulatory authorities access to all study records.

If any regulatory agency schedules an audit, the PI should promptly notify CalciMedica or its representatives and promptly forward to CalciMedica copies of any audit reports he/she receives.

11.4 Study Record Retention

The PI or his/her study staff must retain essential documents for at least 2 years after the last approval of a marketing application in an ICH region. They should retain these documents longer if required because of regulatory requirements or because of an agreement with CalciMedica.

11.5 Study Conduct: Good Clinical Practice and Declaration of Helsinki

CalciMedica will design the clinical study, shall implement it, and report it in accordance with the ICH Harmonized Guideline for Good Clinical Practice, with applicable local regulations (e.g., European Directive 2001/83/EC and US Code of Federal Regulations Title 21), and with the ethical principles laid down in the Declaration of Helsinki.

The PI agrees to conduct the study in accordance with the ICH Guideline for Good Clinical Practice, with applicable local regulations (e.g., European Directive 2001/83/EC and US Code of Federal Regulations Title 21) and with the principles of the Declaration of Helsinki. The PI must conduct all aspects of this study in accordance with all national, state and local laws or regulations.

11.6 Responsibilities of the Investigator and the IRB/EC

A properly constituted IRB/EC must review and approve the protocol and the proposed informed consent form before the start of the study at the site. The PI or his/her study staff must provide CalciMedica or its designee a signed and dated statement that the IRB/EC has approved the protocol and the informed consent form before consenting patients for the study. Prior to starting the study, the PI will sign a protocol signature page confirming that he/she will conduct the study in accordance with this protocol and he/she will give CalciMedica or its designee and regulatory authorities access to all relevant data and records.

The IRB/EC chairperson or designee must sign all IRB/EC approvals and must identify the IRB/EC by name and address, the clinical protocol, and the date of approval.

The PI is responsible for obtaining reviews of the clinical research at intervals specified by the IRB/EC. The specified intervals should not exceed 1 year. The PI must supply CalciMedica or its designee written documentation of the reviews of the clinical research.

11.7 Confidentiality

All laboratory specimens, evaluation forms, reports, and other records will be identified in a manner designed to maintain patient confidentiality. All records will be kept in a secure storage area with limited access. Clinical information will not be released without the written permission of the patient (or the patient's legal representative) except as necessary for monitoring and auditing by CalciMedica or its designee, inspections by relevant regulatory authorities, or reviews by the IRB/EC.

The PI, all study staff and all co-workers involved in the study may not disclose or use for any purpose other than the conduct of the study any data, record or other unpublished confidential information disclosed to them for the purpose of the study. They must obtain prior written agreements from CalciMedica or its designee for the disclosure of any said confidential information to other parties.

11.8 Modification of the Protocol

Any changes that arise after the approval of the protocol must be documented as protocol amendments. Amendments (substantial/non-substantial) require regulatory approval and IRB/EC approval or notification. Only after approval by CalciMedica, the PI, the IRB/EC, and if applicable the regulatory authorities, will the protocol amendments become effective. In cases when the protocol is amended to enhance patient safety, the amendment may be implemented but must be immediately submitted to the IRB/EC and regulatory authorities.

The revision number and date of the amendment will be recorded on the title page of the protocol.

The PI is responsible for informing the IRB/EC of all problems involving risks to patients. In case of urgent safety measures, CalciMedica or its designee will immediately notify the PIs and relevant regulatory authorities.

11.9 Informed Consent

Because the study will be conducted under a United States Investigational New Drug Application, informed consent that is in compliance with Title 21 of the United States Code of Federal Regulations (CFR) Part 50 will be obtained from each patient or LAR before the patient enters the study or before any unusual or non-routine procedure is performed. For sites outside the United States, the signed informed consent form will be obtained in compliance with local regulations, ICH E6 (R2) and the principles of the Declaration of Helsinki.

CalciMedica or its designee may provide to the PI or his/her study staff an informed consent form template. The informed consent form must be reviewed by CalciMedica or its designee before the PI or his/her study staff submits it to the IRB/EC. After CalciMedica or its designee review the informed consent form, the PI or his/her study staff will submit it to the IRB/EC for review and approval. If the informed consent form is revised during the course of the study, CalciMedica or its designee must agree with revisions before the PI or his/her study staff submits it to the IRB/EC. The study staff must provide CalciMedica or its designee a copy of the revised informed consent form after IRB/EC approves it. All patients or LARs affected by the revision must sign the revised informed consent form after the IRB/EC approves it.

Before enrolling in the study, each prospective patient or LAR will receive a full explanation of the study and review the approved informed consent form. Once the PI or designee is assured that the patient or LAR understands the implications of participating in the study, he/she will ask the patient or LAR to give consent for the patient to participate in the study by signing the informed consent form.

A patient may participate in the study only after providing consent using an IRB/EC approved informed consent form. A LAR of the patient may provide informed consent on behalf of the patient under conditions authorized by local laws and regulations. The patient or LAR must provide informed consent before the patient undergoes any study-specific procedures described in the protocol. The PI or designee will provide a copy of the informed consent form to the patient and/or LAR. The process of obtaining informed consent must also be documented in the patient source documents.

11.10 Protocol Deviations

The PI or designee must document any protocol deviation. Reporting of protocol deviations to the appropriate IRB/EC is the responsibility of the PI and must follow the applicable IRB/EC guidelines. A protocol deviation is any change, divergence, or departure from the study design or procedures defined in the protocol.

As referenced in ICH E3, the definition of important protocol deviations for a particular trial is determined in part by study design, the critical procedures, study data, subject protections described in the protocol, and the planned analyses of study data. In keeping with the flexibility of the guidance, sponsors can amend or add to the examples of important deviations provided in ICH E3 in consideration of a trial’s requirements.

For the purposes of this study, an important protocol deviation is one that has an impact on subject safety, may substantially alter risks to subjects, may have an effect on the integrity of the study data, or may affect the subject's willingness to participate in the study.

If there is an immediate hazard to the patient, the PI may deviate from the protocol without prior approval from CalciMedica or its designee and the IRB/EC but must notify CalciMedica or its designee and IRB/EC of the deviation as soon as he/she is able to do so.

11.11 Financial Disclosure

PIs and sub-investigators are required to provide financial disclosure information prior to starting the study. In addition, the PIs and sub-investigators must provide CalciMedica or its designee with updated information if any relevant changes occur during the course of the study and for one year following the completion of the study.

Any PIs, sub-Investigators or study staff with a vested financial interest in the success of the study may not participate in the study.

11.12 Sponsor Obligations

CalciMedica or designee is not financially responsible for further testing/treatment of any medical condition that may be detected during Screening. In addition, CalciMedica is not financially responsible for the treatment of the patient's underlying disease.

11.13 Investigator Documentation

Before beginning the study, the PI will be asked to comply with ICH E6 (R2) 8.2 and title 21 CFR by providing to CalciMedica or designee the following documents:

- The IRB/EC approval of the protocol
- The IRB/EC approved informed consent form
- Any written information regarding the study that will be provided to the patient or LAR
- A Form FDA 1572, fully executed, and all updates on new fully executed Form FDA 1572 (Unless granted an exemption by the FDA and in compliance with local regulations)
- A current Curriculum Vitae for the PI and each sub-Investigator listed on Form FDA 1572. Evidence of licensure must be noted on the Curriculum Vitae or a copy of the license must be provided.
- Completed financial disclosure forms to allow CalciMedica or designee to submit complete and accurate certification or disclosure statements required under US Title 21 CFR 54. In addition, the PI and sub-Investigators must provide to CalciMedica or designee a commitment to update this information promptly if any relevant changes occur during the course of the study and for 1 year following completion of the study.

- Laboratory certifications and normal ranges for any laboratories used by the site for the conduct of the study.

11.14 Clinical Study Insurance

CalciMedica has subscribed to an insurance policy, covering in its terms and provisions its legal liability for injuries caused to participating persons and arising out of this research that is performed strictly in accordance with the scientific protocol as well as with applicable law and professional standards.

11.15 Use of Information

All information supplied by CalciMedica to the PI and his/her study staff is privileged and confidential. The PI and his/her study staff agree to use this information to accomplish the study and not to use it for other purposes without consent from CalciMedica. Furthermore, the PI and his/her study staff are obligated to provide CalciMedica with complete data obtained during the study. The information obtained from the clinical study will be used towards the development of Auxora, and may be disclosed to regulatory authorities, other Investigators, corporate partners or consultants as required.

11.16 Publications

CalciMedica Inc. reserves the right to review all planned communications and manuscripts based on the results of this study. This reservation of the right is not intended to restrict or hinder publication or any other dissemination of study results, but rather to allow CalciMedica to confirm the accuracy of the data, to protect proprietary information, and to provide comments based on information that may not yet be available to the study investigators. CalciMedica Inc. supports communication and publication of study results whatever the findings of the study. CalciMedica Inc. also encourages disclosure of any conflict of interest from all authors or investigators when manuscripts are submitted for publication.

12 REFERENCES

Balthazar EJ. Acute pancreatitis: assessment of severity with clinical and CT evaluation. Radiology. 2002;223(3):603-13. doi: 10.1148/radiol.2233010680.

Banks PA, Bollen TL, Dervenis C, Gooszen HG, Johnson CD, Sarr MG, Tsiotos GG, Vege SS, Acute Pancreatitis Classification Working G. Classification of acute pancreatitis--2012: revision of the Atlanta classification and definitions by international consensus. Gut. 2013;62(1):102-11. doi: 10.1136/gutjnl-2012-302779.

Buxbaum J, Quezada M, Chong B, Gupta N, Yu CY, Lane C, Da B, Leung K, Shulman I, Pandol S, Wu B. The Pancreatitis Activity Scoring System predicts clinical outcomes in acute pancreatitis: findings from a prospective cohort study. Am J Gastroenterol. 2018;113(5):755-64. doi: 10.1038/s41395-018-0048-1.

Byers SO, Friedman M, Sugiyama T. Mechanism underlying phosphatide-induced hypercholesterolemia. J Biol Chem. 1962;237:3375-80.

Criddle DN. Reactive oxygen species, Ca(2+) stores and acute pancreatitis; a step closer to therapy? Cell Calcium. 2016;60(3):180-9. doi: 10.1016/j.ceca.2016.04.007.

Crockett SD, Wani S, Gardner TB, Falck-Ytter Y, Barkun AN, American Gastroenterological Association Institute Clinical Guidelines C. American Gastroenterological Association Institute Guideline on Initial Management of Acute Pancreatitis. Gastroenterology. 2018;154(4):1096-101. doi: 10.1053/j.gastro.2018.01.032.

Dellinger EP, Forsmark CE, Layer P, Levy P, Maravi-Poma E, Petrov MS, Shimosegawa T, Siriwardena AK, Uomo G, Whitcomb DC, Windsor JA, Pancreatitis Across Nations Clinical R, Education A. Determinant-based classification of acute pancreatitis severity: an international multidisciplinary consultation. Ann Surg. 2012;256(6):875-80. doi: 10.1097/SLA.0b013e318256f778.

Fowler AA, III, Truwit JD, Hite RD, Morris PE, DeWilde C, Priday A, Fisher B, Thacker LR, 2nd, Natarajan R, Brophy DF, Sculthorpe R, Nanchal R, Syed A, Sturgill J, Martin GS, Sevransky J, Kashiouris M, Hamman S, Egan KF, Hastings A, Spencer W, Tench S, Mehkri O, Bindas J, Duggal A, Graf J, Zellner S, Yanny L, McPolin C, Hollrith T, Kramer D, Ojielo C, Damm T, Cassity E, Wieliczko A, Halquist M. Effect of Vitamin C Infusion on Organ Failure and Biomarkers of Inflammation and Vascular Injury in Patients With Sepsis and Severe Acute Respiratory Failure: The CITRIS-ALI Randomized Clinical Trial. JAMA. 2019;322(13):1261-70. doi: 10.1001/jama.2019.11825.

Freedman, S., & Forsmark, C. Chronic Pancreatitis: Clinical Manifestations and Diagnosis in Adults. 2020. UpToDate. Retrieved March 22, 2022 from https://www.uptodate.com/contents/chronic-pancreatitis-clinical-manifestations-and-diagnosis-in-adults

Gerasimenko JV, Gryshchenko O, Ferdek PE, Stapleton E, Hebert TO, Bychkova S, Peng S, Begg M, Gerasimenko OV, Petersen OH. Ca2+ release-activated Ca2+ channel blockade as a potential tool in antipancreatitis therapy. Proc Natl Acad Sci U S A. 2013;110(32):13186-91. doi: 10.1073/pnas.1300910110.

Lankisch PG, Apte M, Banks PA. Acute pancreatitis. Lancet. 2015;386(9988):85-96. doi: 10.1016/S0140-6736(14)60649-8.

Lindkvist B, Appelros S, Manjer J, Berglund G, Borgstrom A. A prospective cohort study of smoking in acute pancreatitis. Pancreatology. 2008;8(1):63-70. doi: 10.1159/000114868.

Lur G, Haynes LP, Prior IA, Gerasimenko OV, Feske S, Petersen OH, Burgoyne RD, Tepikin AV. Ribosome-free terminals of rough ER allow formation of STIM1 puncta and segregation of STIM1 from IP(3) receptors. Curr Biol. 2009;19(19):1648-53. doi: 10.1016/j.cub.2009.07.072.

Ma J, Pendharkar SA, O'Grady G, Windsor JA, Petrov MS. Effect of Nasogastric Tube Feeding vs Nil per Os on Dysmotility in Acute Pancreatitis: Results of a Randomized Controlled Trial. Nutr Clin Pract. 2016;31(1):99-104. doi: 10.1177/0884533615603967.

Muallem S, Schoeffield MS, Fimmel CJ, Pandol SJ. Agonist-sensitive calcium pool in the pancreatic acinar cell. I. Permeability properties. Am J Physiol. 1988;255(2 Pt 1):G221-8. doi: 10.1152/ajpgi.1988.255.2.G221.

Petersen OH, Gerasimenko OV, Gerasimenko JV. Pathobiology of acute pancreatitis: focus on intracellular calcium and calmodulin. F1000 Med Rep. 2011;3:15. doi: 10.3410/M3-15.

Petersen OH, Sutton R. Ca2+ signalling and pancreatitis: effects of alcohol, bile and coffee. Trends Pharmacol Sci. 2006;27(2):113-20. doi: 10.1016/j.tips.2005.12.006.

Phillip V, Steiner JM, Algul H. Early phase of acute pancreatitis: Assessment and management. World J Gastrointest Pathophysiol. 2014;5(3):158-68. doi: 10.4291/wjgp.v5.i3.158.

Singh VK, Bollen TL, Wu BU, Repas K, Maurer R, Yu S, Mortele KJ, Conwell DL, Banks PA. An assessment of the severity of interstitial pancreatitis. Clin Gastroenterol Hepatol. 2011;9(12):1098-103. doi: 10.1016/j.cgh.2011.08.026.

Tolstrup JS, Kristiansen L, Becker U, Gronbaek M. Smoking and risk of acute and chronic pancreatitis among women and men: a population-based cohort study. Arch Intern Med. 2009;169(6):603-9. doi: 10.1001/archinternmed.2008.601.

Vincent JL, Rello J, Marshall J, Silva E, Anzueto A, Martin CD, Moreno R, Lipman J, Gomersall C, Sakr Y, Reinhart K, Investigators EIGo. International study of the prevalence and outcomes of infection in intensive care units. JAMA. 2009;302(21):2323-9.

Waldron RT, Chen Y, Pham H, Go A, Su HY, Hu C, Wen L, Husain SZ, Sugar CA, Roos J, Ramos S, Lugea A, Dunn M, Stauderman K, Pandol SJ. The Orai Ca(2+) channel inhibitor CM4620 targets both parenchymal and immune cells to reduce inflammation in experimental acute pancreatitis. J Physiol. 2019;597(12):3085-105. doi: 10.1113/jp277856.

Wen L, Voronina S, Javed MA, Awais M, Szatmary P, Latawiec D, Chvanov M, Collier D, Huang W, Barrett J, Begg M, Stauderman K, Roos J, Grigoryev S, Ramos S, Rogers E, Whitten J, Velicelebi G, Dunn M, Tepikin AV, Criddle DN, Sutton R. Inhibitors of ORAI1 Prevent Cytosolic Calcium-Associated Injury of Human Pancreatic Acinar Cells and Acute Pancreatitis in 3 Mouse Models. Gastroenterology. 2015;149(2):481-92 e7.

Werner J, Feuerbach S, Uhl W, Buchler MW. Management of acute pancreatitis: from surgery to interventional intensive care. Gut. 2005;54(3):426-36. doi: 10.1136/gut.2003.035907.

Whitcomb DC. Clinical practice. Acute pancreatitis. N Engl J Med. 2006;354(20):2142-50. doi: 10.1056/NEJMcp054958.

Yadav D, Lowenfels AB. The epidemiology of pancreatitis and pancreatic cancer. Gastroenterology. 2013;144(6):1252-61. doi: 10.1053/j.gastro.2013.01.068.

Appendix 1. Definition of Organ Failure and Sepsis

The definition of organ failure encompasses three organ systems, respiratory system, renal system and cardiovascular system.

For the respiratory system, organ failure will be defined as:

- $PaO_2/FiO_2 \leq 300$ determined by arterial blood gas or imputed using pulse oximetry, or
- The use of high flow nasal cannula, non-invasive or invasive mechanical ventilation

For the renal system, in patients without known chronic kidney disease (baseline eGFR ≥60 mL/min/1.73 m^2), organ failure will be defined as:

- An increase in serum creatinine ≥300% if the baseline creatinine is known from prior to the hospitalization for AP, or
- A serum creatinine ≥1.9 mg/dL, or
- The initiation of renal replacement therapy

For patients with pre-existing chronic kidney disease (baseline GFR <60 mL/min/1.73 m^2), organ failure will be defined as:

- An increase in serum creatinine ≥300% if the baseline creatinine is known from prior to the hospitalization for AP, or
- An increase of serum creatinine to a level >4.0 mg/dl, or
- The initiation of renal replacement therapy

For the cardiovascular system, organ failure will be defined as:

- A systolic blood pressure <90 mmHg that is not fluid responsive, or
- The use of either vasopressor or inotropic support

Sepsis is the life-threatening organ dysfunction caused by a dysregulated host response to infection. For this study, organ dysfunction in the setting of infection will be defined by the presence of one of the following criteria:

- $PaO_2/FiO_2 \leq 300$ determined by arterial blood gas or imputed using pulse oximetry
- The use of noninvasive or invasive mechanical ventilation
- Platelet count <100 $x10^3$/uL
- Total bilirubin ≥2.0 mg/dL
- The use of vasopressor or inotropic support
- Serum creatinine ≥1.9 mg/dL in patients without pre-existing chronic kidney disease

- Serum creatinine >4.0 mg/dL in patients with pre-existing chronic kidney disease
- An increase in serum creatinine ≥300% if the baseline creatinine is known from prior to the hospitalization for acute pancreatitis
- The initiation of renal replacement therapy

Modified Marshall Scoring System: Organ Dysfunction in Acute Pancreatitis

		**0 (None)**	**1 (None)**	**2 (Mild)**	**3 (Moderate)**	**4 (Severe)**
Respiratory	PaO_2/FiO_2	>400	301-400	201-300	101-200	≤100
Renal	Serum Cr mg/dL	<1.4	1.4-1.8	1.9-3.6	3.7-4.9	>4.9
CV no inotrope	SBP mmHg	≥90	<90 fluid responsive	<90 not fluid responsive	<90, pH <7.3	<90, pH <7.2

CV = cardiovascular

Adapted from Banks 2013

Appendix 2. American Gastroenterological Association Institute Guideline on the Initial Management of Acute Pancreatitis

	**Recommendation**	**Strength**	**Quality**
1a.	In patients with AP, the AGA suggests using goal-directed therapy for fluid management.<br>*Comment: The AGA makes no recommendation whether normal saline or Ringer's lactate is used.*	Conditional	Very Low
1b.	In patients with AP, the AGA suggests against the use of HES fluids	Conditional	Very Low
2	In patients with predicted severe AP and necrotizing AP, the AGA suggests against the use of prophylactic antibiotics	Conditional	Low
3	In patients with acute biliary pancreatitis and no cholangitis, the AGA suggests against the routine use of urgent ERCP	Conditional	Low
4	In patients with AP, the AGA recommends early (within 24 h) oral feeding as tolerated, rather than keeping the patient nil per os	Strong	Moderate
5	In patients with AP and inability to feed orally, the AGA recommends enteral rather than parenteral nutrition	Strong	Moderate
6	In patients with predicted severe or necrotizing pancreatitis requiring enteral tube feeding, the AGA suggests either NG or NJ route	Conditional	Low
7	In patients with acute biliary pancreatitis, the AGA recommends cholecystectomy during the initial admission rather than after discharge	Strong	Moderate
8	In patients with acute alcoholic pancreatitis, the AGA recommends brief alcohol intervention during admission	Strong	Moderate

AGA = American Gastroenterological Association; AP = acute pancreatitis; ERCP = endoscopic retrograde cholangiopancreatography; HES = hydroxyethyl starches; NG = nasogastric; NJ = nasojejunal

Adapted from Crockett 2018

Appendix 3. CTSI Score

Grade	CT Finding	Points
A	Normal pancreas	0
B	Enlargement of pancreas	1
C	Inflammatory changes in pancreas and peripancreatic fat	2
D	Ill-defined single peripancreatic fluid collection	3
E	Two or more poorly defined peripancreatic fluid collections	4

Pancreatic Necrosis	Points
None	0
<30%	2
30-50%	4
>50%	6

Adapted from Balthazar 2002

The CTSI score is the sum of the Balthazar and Pancreatic Necrosis scores:

- 0-3: mild acute pancreatitis
- 4-6: moderate acute pancreatitis
- 7-10: severe acute pancreatitis

Appendix 4. Pain Numeric Rating Scale (PNRS)

Pain Numeric Rating Scale

1. On a scale of 0 to 10, with 0 being no pain at all and 10 being the worst pain imaginable, how would you rate your pain RIGHT NOW.

0	1	2	3	4	5	6	7	8	9	10
**No Pain**										**Worst Pain Imaginable**

Appendix 5. Imputed PaO_2/FiO_2

FiO_2 \ SpO_2	0.7	0.75	0.8	0.81	0.82	0.83	0.84	0.85	0.86	0.87	0.88	0.89	0.9	0.91	0.92	0.93	0.94	0.95	0.96
**0.21**	174	191	211	216	221	226	232	238	245	252	260	269	279	291	304	319	337	360	~~390~~
**0.24**	153	167	185	189	193	198	203	208	214	221	228	236	244	254	266	279	295	315	341
**0.27**	136	148	164	168	172	176	180	185	190	196	202	209	217	226	236	248	262	280	303
**0.3**	122	133	148	151	155	158	162	167	171	177	182	189	196	203	213	223	236	252	273
**0.35**	105	114	127	129	132	136	139	143	147	151	156	162	168	174	182	191	202	216	234
**0.4**	92	100	111	113	116	119	122	125	129	132	137	141	147	153	159	168	177	189	205
**0.45**	81	89	98	101	103	106	108	111	114	118	121	126	130	136	142	149	157	168	182
**0.5**	73	80	89	91	93	95	97	100	103	106	109	113	117	122	128	134	142	151	164
**0.55**	67	73	81	82	84	86	89	91	94	96	99	103	107	111	116	122	129	138	149
**0.6**	61	67	74	76	77	79	81	83	86	88	91	94	98	102	106	112	118	126	136
**0.65**	56	62	68	70	71	73	75	77	79	81	84	87	90	94	98	103	109	116	126
**0.7**	52	57	63	65	66	68	70	71	73	76	78	81	84	87	91	96	101	108	117
**0.75**	49	53	59	60	62	63	65	67	69	71	73	75	78	81	85	89	94	101	109
**0.8**	46	50	55	57	58	59	61	63	64	66	68	71	73	76	80	84	89	95	102
**0.85**	43	47	52	53	55	56	57	59	61	62	64	67	69	72	75	79	83	89	96
**0.9**	41	44	49	50	52	53	54	56	57	59	61	63	65	68	71	74	79	84	91
**0.95**	39	42	47	48	49	50	51	53	54	56	58	60	62	64	67	71	75	80	86
**1**	37	40	44	45	46	47	49	50	51	53	55	57	59	61	64	67	71	76	82

Adapted from Fowler 2019

Appendix 6. Modified American Neurogastroenterology and Motility Society Gastrointestinal Cardinal Symptom Index Daily Dairy (mGCSI-DD)

	None	Mild	Moderate	Severe	Very Severe
Nausea (feeling sick to your stomach as if you are going to vomit or throw up)	☐	☐	☐	☐	☐
Not able to finish a normal sized meal (for a healthy person)	☐	☐	☐	☐	☐
Feeling excessively full after meals	☐	☐	☐	☐	☐
Abdominal Pain (around or above the navel)	☐	☐	☐	☐	☐
The next question asks you to record the number of times vomiting occurred in the last 24 hours. Please record the number of vomits (throwing up with food or liquid coming out) that occurred in the last 24 hours. Record zero, if you have not vomited during the past 24 hours. If you vomited, write down the number of all vomits. If you vomited once, record one. If you vomited three times during the day, record three. If you vomited three times, whether it was during the same trip to the bathroom or three separate trips, record three as the number of episodes of vomiting.<br><br>During the past 24 hours, how many episodes of vomiting did you have: ______________________					
In thinking about your pancreatitis, what was the overall severity of your symptoms over the past 24 hours	☐	☐	☐	☐	☐

Appendix 7. CT Findings in Chronic Pancreatitis

CT findings in chronic pancreatitis

Grade	CT findings
Normal	No abnormal findings on a good-quality study visualizing the entire gland
Equivocal	One of the following:<br>▪ Mild dilatation of the pancreatic duct (2 to 4 mm) in the body of the gland<br>▪ Gland enlargement ≤2-fold normal
Mild-moderate	One of the preceding findings plus at least one of the following:<br>▪ Pancreatic duct dilatation (>4 mm)<br>▪ Pancreatic duct irregularity<br>▪ Cavity (ies) <10 mm<br>▪ Parenchymal heterogeneity<br>▪ Increased echogenicity of duct wall<br>▪ Irregular contour of the head or body<br>▪ Focal necrosis or loss of parenchyma
Severe	Mild/moderate features plus one or more of the following:<br>▪ Cavity (ies) >10 mm<br>▪ Intraductal filling defects<br>▪ Calculi/pancreatic calcification<br>▪ Ductal obstruction (stricture)<br>▪ Severe duct dilatation or irregularity<br>▪ Contiguous organ invasion

CT: computed tomography.

UpToDate®

Adapted from Freedman 2020

Appendix 8. Schedule of Events

	Screening	If Eligible, Baseline Assessment	R	SFISD	24 (±1) Hours[i]	48 (±1) Hours[i]	72 (±2) Hours	96, 144, 192, 240 (±4) Hours[h,k]	120, 168, 216 (±4) Hours[h,k]	Days 12-28 Q48 (±4) Hours[h,k]	Day 30 (±5) Days
Informed Consent	X										
Demographics	X										X[l]
Medical History	X										
List of Medications	X										
Concomitant Meds				X	X	X	X	X	X		X
Height		X									
Weight		X					X				X
Vital Signs	X				X	X	X	X	X		X
Abdominal Exam	X										
PNRS		X			X	X	X	X	X		X
Serum Lipase	X[a]										
CBC+diff+Platelets	X[a]				X[a]	X[a]	X[a]		X[a]		X[a]
Presence of SIRS[b]	X										
SpO_2+FiO_2		X			X	X	X	X	X		X
CECT of Pancreas[c]	X										X
Serum Pregnancy Test	X[a]										
Randomize Patient[d]			X								
Serum Chemistries		X[a]			X[a]	X[a]	X[a]		X[a]		X[a]
Serum Procalcitonin		X[a]			X[a]	X[a]	X[a]		X[a]		X[a]
Serum Biomarker[e]		X			X	X	X		X		X
Urine Biomarker[e]		X			X	X	X		X		X
Administer Study Drug				X	X[i]	X[i]					
PK sample[e]				X[j]	X[j]		X[j]				X[j]
Medically Indicated Discharge Criteria[f]					X	X	X	X	X	X	X
Enhanced Recovery Strategy[g]				X	X	X	X	X	X	X	X[m]
mGCSI-DD[h]		X[h]			X[h]					X[h]	X[h]
AE/SAE Evaluation			X	X	X	X	X	X	X	X	X
Telephone Contacts, as needed		Following patient discharge, Investigator or designee should contact patient at least once weekly to confirm diary completion, Day 30 visit schedule and assess for AE/SAEs.									

AE = adverse event; CECT = contrast-enhanced computed tomography; PK = pharmacokinetics; mGCSI-DD = Modified American Neurogastroenterology and Motility Society Gastrointestinal Cardinal Symptom Index Daily Diary; PI = Principal Investigator; PNRS = Pain Numeric Rating Scale; SAE = serious adverse event; SFISD = Start of First Infusion of Study Drug

a. Blood sample for serum pregnancy test, serum lipase and CBC+diff+Platelets at Screening will be analyzed at local laboratory. May use results obtained as standard of care if blood drawn in the previous 12 hours prior to consent or following consent but prior to randomization. Following screening, all protocol-required blood samples, except as noted in Footnote "e", will be analyzed at local laboratory. Baseline blood samples including biomarkers may be performed on blood drawn in the previous 12 hours of randomization. For all later assessment times, blood drawn in the previous 12 hours maybe used (except PK).

b. The most extreme value for each criterion in the 24 hours before consent or following consent but prior to randomization may be used.

c. A CECT already performed in the 24 hours prior to the patient or LAR providing informed consent may be used as the Screening CECT. In addition to the Screening and the Day 30 visit CECTs, CECTs performed as standard of care after randomization and to 25 days after SFISD will be captured and submitted for central reading. If the CECT is performed 25 to 29 days after SFISD, it will substitute for the Day 30 visit CECT scan and will be captured.

d. Hematocrit collected as standard of care within 12 hours of consent or following consent but prior to randomization may be used for Inclusion Criterion 3c.

e. Biomarker blood and urine samples and blood samples for PK will be analyzed at central laboratory. If site or patient does not participate in these assessments this will be documented at start of study and will not be considered a protocol deviation.

f. Determine if patient had met the medically indicated discharge criteria within the prior 24- or 48-hour visit assessment.

g. Patients will be offered a low fat, ≥500-calorie solid meal at each mealtime after the infusion of the first dose of study drug until discharge if alert and not on mechanical ventilation, or if not NPO for a planned surgery/medical procedure, or if not NPO because of an acute medical condition. If the patient does not wish to eat the solid meal when offered or is unable to tolerate the solid meal, they should then be offered a liquid meal. Determine for each meal if the patient ate ≥50% of the meal without vomiting or having abdominal pain in the two hours after the meal.

h. The mGCSI-DD score will be completed by all eligible patients at the baseline assessment and on the day of discharge, which will vary by patient as shown in schedule above. In addition, if the patient remains hospitalized at 96 and/or 168 hours and/or at Day 14 and/or Day 21 patient should be requested to record their symptoms at bedtime using the mGCSI-DD worksheet. Patients will be trained on completing the mGCSI-DD worksheet. Upon discharge, patient will record the mGCSI-DD score daily before bedtime until the Day 30 visit.

i. In the scenario where the PI or treating physician wants the infusion(s) to begin earlier than the 24 +/- 1 hour window, the PI or treating physician should contact the medical monitor to discuss whether it is appropriate to begin infusion earlier (up to 6 hours earlier) than the 24 +/- 1 hour protocol window.

j. Blood samples for PK analysis will be drawn as soon as possible after the completion of the first infusion of study drug (within +2 hours), prior to the start of second infusion of study drug (within -30 min), at 72 hours (±2 hours) from the SFISD, and at the Day 30 visit. If the patient is discharged after the third infusion of study drug and prior to 72 hours from the SFISD, the blood sample will be drawn prior to discharge.

k. Study assessments continue daily, or every other day, as noted in schedule of events until discharge or the Day 30 visit. All patients return for Day 30 assessments if discharged earlier than Day 25.

l. At the Day 30 visit (or at the time of death if before Day 30), record the Investigator's opinion on the etiology of the episode of AP.

m. Only patients who remain hospitalized at Day 30.

CM4620-203: A Randomized, Double-Blind, Placebo Controlled Dose-Ranging Study of Auxora in Patients with Acute Pancreatitis and Accompanying Systemic Inflammatory Response Syndrome (CARPO)

Version 3.0 March, 2022

Summary of Changes:
Version 3.0 of the protocol is amended to expand the time frame for the screening CECT from 12 hours to 24 hours prior to Informed Consent to minimize the need for potentially eligible patients to need a repeat. The time frame for the SIRS symptoms used for patient eligibility was expanded from 6 hours to 12 hours. Study procedures were amended to have sites remain in contact with patients following hospital discharge in advance of the Day 30. Visit were clarified to help ensure compliance with diary completion and minimize missing data. Finally, some administrative changes were made to clarify wording that was unclear within the visit assessments and schedule of events.

There is no change to exposure or risk to patients.

New wording will be displayed with an underline and replaced wording ~~crossed through.~~
"…" : denotes a purposeful gap in text from the protocol to summary of change document as there were no changes made to the text with the amendment

A summary of changes with rationale follows:

#	Nature of change from protocol V 2.0/ Dec. 14, 2020	Rationale
1	Cover Page<br>Added IND #123533<br>Updated CalciMedica company logo	Good documentation / good research practices
2	Study Synopsis<br>Updated throughout to match the changes noted below.	Good documentation / good research practices
3	Section 3.1 Study Design – various updates as noted below<br>All patients will have received a Screening CECT of the abdomen/pancreas before being randomized into the study. CECTs performed as standard of care may be used as the Screening CECT but must have been	Minimize patients having to repeat CECT to determine eligibility for the study.

#	**Nature of change from protocol V 2.0/ Dec. 14, 2020**	**Rationale**
	performed in the ~~12~~24 hours before Consent or after Consent and before Randomization.	
	For all 4 groups, a study physician or appropriately trained delegate will perform study-specific assessments at screening, at the baseline assessment, immediately prior to the SFISD, and then every 24 hours until 240 hours after the SFISD, or until discharge if earlier. If patients remain hospitalized at Day 12, assessments will then be performed every 48 hours starting on Day 12 until Day 28, or until discharge if earlier.<br>…	Prior language did not provide sufficient specificity
	Patients will receive another CECT of the abdomen/pancreas at the Day 30 (±5 days) visit. All CECTs performed as standard of care after randomization and before the Day 30 CECT will also be captured. A blinded central reader will read the Screening, Day 30, and any standard of care CECTs obtained between randomization and the Day 30 visit~~30 days~~..<br>…	Standardize language in the protocol
	In patients with acute pancreatitis, the AGA strongly recommends early oral feeding (within 24 hours) rather than keeping the patient nil per mouth (Nil per Os, NPO). Patients randomized into the study, therefore, will be offered a low fat, ≥500-calorie solid meal at each mealtime after the infusion of the first dose of study drug if alert and not on mechanical ventilation, or if not NPO for a planned surgery/medical procedure, or if not NPO because of an acute medical condition. If the patient does not wish to eat the solid meal when offered or is unable to tolerate the solid meal, they should then be offered a liquid meal. The same approach should occur at each subsequent mealtime until discharge.<br>…	Standardize language to identify patients who are NPO for conditions other than acute pancreatitis<br><br>Prior language did not provide sufficient specificity
	It is also recommended that all patients randomized in the study should not be discharged from the hospital	Language to standardize patient status at the time of discharge

#	Nature of change from<br>protocol V 2.0/ Dec. 14, 2020	Rationale
	until solid food is tolerated, abdominal pain has resolved or been adequately controlled, and there is no clinical evidence of infection necessitating continued hospitalization.	
4	Section 3.2 End of Study<br><br>The End of Study is considered the date on which the last patient randomized completes the ~~visit on~~ Day 30 visit, unless CalciMedica terminates the study early.	Prior language did not provide sufficient specificity
5	Section 4.1 Inclusion Criteria<br>3. At least one of the following criteria is present:<br>a. A peripancreatic fluid collection or a pleural effusion on a contrast-enhanced computed tomography (CECT) performed in the ~~12~~ 24 hours before Consent or after Consent and before Randomization;<br>5. Lack of pancreatic necrosis, pancreatic calcifications, pancreatic pseudocysts and no evidence for previous necrosectomy or pancreatic surgery identified by CECT performed in the ~~12~~ 24 hours before Consent or after Consent and before Randomization;	Minimize patients having to repeat CECT to determine potential eligibility for enrolment
6	Section 4.3 Rescreening<br><br>A patient who fails the initial screening because SIRS was not present, or either guarding or rebound were not present, or HCT was not ≥ 44% for a man or ≥ 40% for a woman may be re-screened one time ~~once again within 6 hours of the original consent~~. The CECT used to evaluate for inclusion and exclusion criteria must be within ~~12~~ 24 hours of the rescreening or must be performed after rescreening and before randomization.	Prior language did not provide specificity
7	Section 5.2 Enhanced Recovery Strategy<br><br>Patients randomized into the study will be offered a low fat, ≥500-calorie solid meal at each mealtime after the infusion of the first dose of study drug if alert and not	Standardize language to identify patients who are NPO for conditions other than acute pancreatitis

#	**Nature of change from protocol V 2.0/ Dec. 14, 2020**	**Rationale**
	on mechanical ventilation or if not NPO for a planned surgery/medical procedure, or if not NPO because of an acute medical condition. If the patient does not wish to eat the solid meal when offered or is unable to tolerate the solid meal, they should then be offered a liquid meal. The same approach should occur at each subsequent mealtime until discharge.	Prior language did not provide sufficient specificity
8	Section 5.3 Discharge Criteria<br><br>• There is no clinical evidence of an infection necessitating continued hospitalization.	Language to standardize patient status at the time of discharge
9	Section 5.4 Prohibited Medications<br><br>Added three (3) additional medications:<br>• clazakizumab<br>• Baracitinib<br>• Tofacitinib	Ensure appropriate patients are enrolled to study
10	Section 6.2 Discontinuation and Withdrawal<br><br>The term treatment discontinuation refers to a patient or PI discontinuing the administration of study drug before all 3 doses are administered despite the patient remaining in the hospital. Patients who do not receive all 3 doses because the treating physician discharged them from the hospital will not be considered as treatment discontinuation ~~to have discontinued study medication~~.<br>…<br><br>The PI should also record the reason for the treatment discontinuation in the eCRF and appropriate source documents at the site. Even if the patient ~~discontinues receiving study drug~~ meets the definition of treatment discontinuation, patient should be encouraged to continue participation in the study for all regularly scheduled assessments, for safety and for efficacy	Clarify wording to be consistent with FDA guidance<br><br>Clarify wording to be consistent with FDA guidance

#	**Nature of change from protocol V 2.0/ Dec. 14, 2020**	**Rationale**
	~~diligence should occur that all study visits and assessments are completed~~.<br><br>…<br><br>Patients will only be considered as lost to follow-up (LTF) after PI or designee documents they are unable to contact patient to complete final study assessment(s) or is unable to ascertain vital status (see Section 8.10).	
11	Section 7.1, Table 4<br><br>Removed reference to Manufacturer in table	This information is not required in the protocol.
12	Section 7.6 Packaging and Labeling<br><br>Preparation, packaging and labeling of Auxora or Placebo will be in accordance with current Good Manufacturing Practice of Medicinal Products (GMP) guidelines. Medication labels will comply with legal requirements for labeling of investigational products in the United States and/or other country requirements, as applicable.	Prior language did not provide sufficient specificity if other countries participate in the study.
13	Section 8.1 Screening<br><br>After informed consent is obtained, the following procedures are to be performed unless already performed as part of standard of care:<br><br>Record the time of the onset of abdominal pain that sent patient to ER<br><br>…<br><br>Draw serum pregnancy test if the patient is a female of childbearing potential – analyze at local lab<br><br>o If already performed as part of standard of care, record results. Testing may be performed on blood drawn in the previous ~~6~~ 12 hours prior to Consent or following consent but prior to randomization.<br><br>Draw a blood sample for serum lipase–analyze at local lab	To standardize timing of study specific assessments to match SIRS screening criteria.

#	**Nature of change from protocol V 2.0/ Dec. 14, 2020**	**Rationale**
	o If already performed at local lab as part of standard of care, record results. Testing may be performed on blood drawn in the previous ~~6~~ <u>12</u> hours prior to Consent <u>or following consent but prior to randomization</u>.<br><br>Draw a blood sample for CBC, differential, and platelets–analyze at local lab<br><br>o If already performed at local lab as part of standard of care, record results. Testing may be performed on blood drawn in the previous ~~6~~ <u>12</u> hours prior to Consent <u>or following consent but prior to randomization</u>.<br><br>…<br><br>o If CECT already performed in the ~~12~~<u>24</u> hours prior to the patient or LAR providing informed consent, use results from that CECT	Minimize patients having to repeat CECT to determine eligibility for the study.
14	Section 8.2 Baseline<br><br>Perform the following prior to randomization ~~(2 hours)~~:<br><br>…<br><br>• Draw blood samples for Serum Procalcitonin and Serum Chemistries- analyze at local lab.<br><br>o If already performed at local lab as standard of care, record results. Testing may be performed on blood drawn in the previous ~~6~~ <u>12</u> hours of randomization.<br><br>• Draw blood sample for Serum IL-6 –send to central lab. May be performed on blood drawn in the previous ~~6~~<u>12</u> hours <u>of randomization.</u><br><br>Obtain urine sample for Urine NGAL-send to central lab. May be performed on urine collected in the previous ~~6~~<u>12</u> hours <u>of randomization.</u>	To standardize timing of study specific assessments to match SIRS screening criteria.

#	Nature of change from protocol V 2.0/ Dec. 14, 2020	Rationale
15	Section 8.4 Start of the First Infusion (SFISD)<br>• Begin Enhanced Recovery Strategy for Tolerance of Solid Food (see Section **Error! Reference source not found.**) and continue daily until ~~the~~ patient ~~is tolerating solid food~~ discharge.	Clarify visit assessments
16	Sections 8.5 and 8.6<br>24 hours<br>48 hours<br>• Determine if the patient met the medically indicated discharge criteria in the previous 24 hours (see Section 5.3)	Clarify visit assessments
17	Section 8.7 72 Hours<br>• Determine if the patient met the medically indicated discharge criteria in the previous 24 hours (see Section 5.3)<br>• Draw blood samples for CBC, differential, platelets, Serum Procalcitonin and Serum Chemistries – analyze at local lab. Testing may be performed on blood drawn in the previous 12 hours or + 2 hours from assessment.<br>• Draw blood sample for Serum IL-6 –send to central lab. Testing may be performed on blood drawn in the previous 12 hours or + 2 hours from assessment.<br>• Draw blood sample for PK ± 2 hours– send to central lab<br>• Obtain urine sample for Urine NGAL-send to central lab. Testing may be performed on urine collected in the previous 12 hours or + 2 hours from assessment.	Clarify visit assessments<br><br>Standardization to study specific blood draws for this visit assessment

#	**Nature of change from protocol V 2.0/ Dec. 14, 2020**	**Rationale**
18	Section 8.8 96 - 240 hours (± 4 hours)<br>• Determine if the patient met the medically indicated discharge criteria in the previous 24 hours (see Section 5.3)<br><br>In addition, for patients who remain hospitalized 120, 168, and 216 hours from the SFISD:<br>• Draw blood samples for CBC, differential, platelets, Serum Procalcitonin, and Serum Chemistries – analyze at local lab. Testing may be performed on blood drawn in the previous 12 hours or + 4 hours from assessment.<br>• Draw blood sample for Serum IL-6 –send to central lab. Testing may be performed on blood drawn in the previous 12 hours or + 4 hours from assessment.<br>• Obtain sample for Urine NGAL-send to central lab. Testing may be performed on urine collected in the previous 12 hours or + 4 hours from assessment.	Clarify visit assessments<br><br>Standardization to study specific blood draws for this visit assessment
19	Section 8.9 Days 12 to 28 (± 4 hours)<br>• Determine if the patient met the medically indicated discharge criteria in the previous 48 hours (see Section 5.3)	Clarify visit assessments
20	Section 8.10 Day 30 (± 5 days)<br>• Determine if the patient met the medically indicated discharge criteria in the previous 48 hours (see Section 5.3)<br><br>For patients that do not return for Day 30 visit and have not withdrawn consent, the Investigator or designee will continue to contact patient by phone to return for on-site visit up to three weeks following visit date. If patient is not reachable by phone after four weeks following the planned Day 30 visit, the Investigator or designee will send certified letter to last known address to request patient to contact Investigator to either schedule Day 30 visit or at least confirm patient is still alive by telephone. If patient does not respond to certified letter or if patient had withdrawn consent, the Investigator or designee will check public records (e.g.,	Clarify visit assessments<br><br>To be consistent with FDA guidance

#	**Nature of change from protocol V 2.0/ Dec. 14, 2020**	**Rationale**
	vital records search) to determine if the patient had died prior to the scheduled Day 30 visit or can be assumed alive for the purposes of study follow-up.	
21	Section 8.11 Discharge Date<br><br>The time and date of discharge will be recorded as well as if the patient meets criteria for medically indicated discharge. All patients will be trained to complete the mGCSI-DD and will complete the mGCSI-DD before leaving the hospital. If patients are discharged on Days 25-29, they may complete the Day 30 assessments on the day of discharge. Following discharge, Investigator or designee should contact patient by telephone once per week to confirm diary completion and return for Day 30 visit.	To clarify scope of responsibilities
22	Section 8.12.7 Laboratory Assessments<br><br>If a site is unable to perform any of the laboratory tests because it is not offered at the site ~~or is not part of standard of care testing~~, it will not be performed on patients randomized at the site. The only exception to this would be absolute and/or percent neutrophil and lymphocyte counts.<br><br>Blood and urine samples for biomarker assessments (non-genetic testing) including, but not limited, to IL-6 and urine samples for urine NGAL will be collected at baseline, 24, 48, and 72 hours, and if the patient remains hospitalized, at 120, 168, 216 hours following SFISD as well as at Day 30 assessment. A reference laboratory will analyze these blood and urine samples.~~IL-6 and urine NGAL~~. The results of these assays will not be available to the PI or treating physician in managing the patient. It is the expectation that all sites participate in biomarker collection; however, if a site is unable to provide blood or urine samples for the biomarker analysis, this will be determined at study start and not considered a protocol deviation for that site. In addition, if a site is participating in biomarker collection but a patient does not want to provide biomarker samples this will be	Clarify visit assessments<br><br>To clarify that genetic testing will not be performed

#	Nature of change from protocol V 2.0/ Dec. 14, 2020	Rationale
	documented in the informed consent and not considered a protocol deviation.	
23	Section 8.12.9 Enhanced Recovery Strategy<br>Patients randomized into the study will be offered a low fat, ≥500-calorie solid meal at each mealtime after the infusion of the first dose of study drug until discharge if alert and not on mechanical ventilation, presurgical NPO, or has an acute medical condition. See Section **Error! Reference source not found.** for specific assessment procedures	Standardize language to identify patients who are NPO for conditions other than acute pancreatitis
24	Section 8.12.12 Contrast-Enhanced CT<br>…<br>The lack of perfusion of the pancreas on the CECT will be exclusionary. The CECT protocol may be altered to local standard of care. If a CECT had already been performed more than 24 hours but less than 48 hours before Consent, and there is a contraindication to repeating it, the PI should discuss with the medical monitor if the CECT may be used for evaluating the inclusion and exclusion criteria for the study. The PI or treating physician may also elect to delay the Day 30 CECT (±5 days) CECT due to patient-specific safety considerations but must inform the Medical Monitor of the decision. The PI or treating physician may also elect not to perform the CECT because of futility considerations, e.g., the patient is unlikely to survive in the next 48 hours, but must discuss the decision with the Medical Monitor.<br>The CECT may also be used to make the diagnosis of chronic pancreatitis. Patients with a diagnosis of chronic pancreatitis in the chart or who self-report chronic pancreatitis, but are neither on enzyme replacement nor following up with a gastroenterologist as an outpatient, should be considered for the study after a careful review of a CECT. (Appendix 7). If the findings in the mild-moderate or severe categories are not present on the CECT, please contact the medical monitor to discuss whether the patient would be appropriate for randomization if they meet the other inclusion and none of the exclusion criteria. The	Prior language did not provide sufficient specificity<br>Prior language did not provide sufficient specificity

#	Nature of change from protocol V 2.0/ Dec. 14, 2020	Rationale
	'equivocal' criteria do not exclude patients from randomization.<br><br>…<br><br>If the patient undergoes additional CECTs at the discretion of the PI or treating physician from randomization to the Day 30 visit, the date of the CECT will be recorded and a blinded central reader will read the images.	
25	Section 8.12.13 Systemic Inflammatory Response Syndrome (SIRS)<br><br>The physician, or appropriately trained designee will determine the presence of SIRS at Screening. The most extreme value for each criterion in the ~~6~~ 12 hours before Screening may be used.	Allow sites to obtain vitals prior to administration of opioids that would mask the presence of SIRS
26	Section 10.7 Efficacy Analysis<br><br>Efficacy endpoints detailed in synopsis were added to this section without modification.<br><br>Time to solid food tolerance is the number of hours from the date and time of the SFISD for the patient to the first date and time the patient receives a solid meal that is tolerated, which is defined as eating ≥50% of a low fat, ≥500-calorie solid meal without an increase in abdominal pain or vomiting in the two hours after completing the meal. ~~Time to medically indicated discharge is the number of days from the SFISD to the date of meeting discharge criteria in Section **Error! Reference source not found.**~~.<br><br>Time to solid food tolerance for patients who could not tolerate solid food during the study will be censored on the latest date and time of food tolerance assessment ~~measurement~~. ~~Time to medically indicated discharge for patients who do not have documented medically indicated discharge per discharge criteria in Section **Error! Reference source not found.** during the study~~	To make this section consistent with synopsis and to clarify the analysis that will be performed

#	Nature of change from protocol V 2.0/ Dec. 14, 2020	Rationale
	will be censored on the earliest date of hospital discharge or study withdrawal or study completion.<br><br>Time-to-event endpoints will be summarized using Kaplan-Meier analyses. The medians and quartiles of time-to-events will be summarized. Time to solid food tolerance will be compared between the each treatment groups and the placebo group using the stratified log-rank test as the primary analysis. The tests will be at an alpha level of 0.05 without multiplicity adjustment. The stratification variables will include gender (male vs. female) and disease severity status (higher risk for organ failure defined by the presence of both a HCT ≥44% for men, or ≥40% for women, and a PaO_2/FiO_2 ≤360 before randomization (**Error! Reference source not found.**).<br><br>…<br><br>Time to medically indicated discharge is the number of days from the SFISD to the date of meeting discharge criteria in Section **Error! Reference source not found.** and will be compared between each treatment group and the placebo group using the stratified log-rank test patients who could tolerate solid food and who could not tolerate solid food at 48, 72, and 96 hours using Kaplan-Meier curve and log-rank test.<br><br>Other secondary and exploratory efficacy endpoints will be summarized with details described in the separate statistical analysis plan (SA)out profile.	
27	Section 12 REFERENCES	Added new reference as noted in Section 8.12.12
28	Appendix 7: CT Findings in Chronic Pancreatitis	Added new reference as noted in Section 8.12.12

#	**Nature of change from protocol V 2.0/ Dec. 14, 2020**	**Rationale**
29	Appendix ~~7~~ 8: Schedule of Events<br><br>➢ Corrected typographical error to change ‘serum’ to ‘plasma’ PK<br>➢ Clarified reference to biomarkers for consistency with section 8.12.7<br>➢ Added assessment to determine if patient had met the medically indicated discharge criteria within the prior 24- or 48-hour visit assessment period.<br>➢ Added telephone contacts to patient following discharge to help ensure compliance with diary completion, assess for AEs/SAEs and return for Day 30 visit.<br>➢ Clarified footnotes to improve protocol adherence	Updated to reflect clarifications made in Section 8 – Visit and Assessments; renumbered Appendix sequence to 8

Version 4.0 October 05, 2023

There is no change to exposure or risk to patients with this Protocol Amendment. Minor typographical, grammatical, and administrative additions to provide cross section references were made to the document.

New wording will be displayed with an underline and replaced wording ~~crossed through.~~
“…” : denotes a purposeful gap in text from the protocol to summary of change document as there were no changes made to the text with the amendment.

All other changes are listed with rationale below:

#	**Nature of change from protocol V 3.0/ March 31, 2022**	**Rationale**
1	Cover Page<br>Updated CalciMedica company logo	Good documentation / good research practices
2	Study Synopsis<br>Updated throughout to match the changes noted below	Good documentation / good research practices

#	Nature of change from protocol V 3.0/ March 31, 2022	Rationale
3	Section 1.3 Overview of CM4620<br><br>CM4620 (also known by the international nonproprietary name, zegocractin) is a potent and selective inhibitor of CRAC channels. CRAC channels …	Zegocractin is the International Nonproprietary Name (INN) for CM4620
4	Study Synopsis & Protocol<br><br>Throughout document replaced CM4620 with zegocractin and CM4620-IE with Auxora (zegocractin intravenous emulsion)	Zegocractin is the International Nonproprietary Name (INN) for CM4620 and Auxora is the trademarked name for the zegocractin intravenous emulsion.
5	Figure 2 replaced with updated graphic	To highlight the potential role of CM4620 in treating Acute Pancreatitis at a macroscopic (organ) level
6	Section 3.1 Study Design<br><br>…<br><br>The sponsor, investigators, pharmacists, and patients will be blinded to the assigned group. In the event of a medical emergency, investigators will be able to receive the treatment assignment if required to provide optimal care of the patient.	Prior language did not provide specificity to mention pharmacists, as pharmacists were included as investigators.
7	Section 4.3 Rescreening<br><br>A patient who fails the initial screening ~~because SIRS was not present, or either guarding or rebound were not present, or HCT was not ≥44% for a man or ≥40% for a woman,~~ may be rescreened once again (e.g. a reversible cause of screen failure has resolved and the subject meets all eligibility criteria). ~~The CECT used to evaluate for inclusion and exclusion criteria must be within 24 hours of the rescreening or must be performed after rescreening and before randomization.~~	Language updated for clarity and succinctly indicate that when screen failure issue resolves, rescreens are allowed if all eligibility criteria requirements are met.

#	**Nature of change from protocol V 3.0/ March 31, 2022**	**Rationale**
8	Section 5.3 Discharge Criteria<br><br>~~If the patient is ready to be discharged before all 3 doses of study drug have been administered, the PI or treating physician should contact the Medical Monitor prior to discharging the patient.~~ In the scenario where the PI or treating physician wants the infusion(s) to begin earlier than the 24 hour +/- 1 hour window (e.g. the patient is ready to be discharged before all 3 doses of study drug have been administered and / or to facilitate an infusion time where study personnel are available), the PI or treating physician should contact the medical monitor to discuss whether it is appropriate to begin infusion earlier (up to 6 hours earlier) than the 24 hour +/- 1 hour protocol window.	Given no safety issues, flexibility is justified in relation to protocol being implemented within standard of care ER/inpatient hospital stays and to minimize the risk of reoccurrence due to lack of full treatment course. No meaningful difference in exposure is expected when modifying the infusion start window from -1 hour to within -6 hours, and discussion with the Medical Monitor will allow for clinical practice flexibility while maintaining drug exposure limits.
9	Section 5.4 Prohibited Medications<br><br>Added clarifying language and<br>one (1) additional prohibited medication:<br><br>Biologics/Monoclonals: such as, but not limited to,<br>…<br><br>guselkumab	Ensure appropriate patients are enrolled to study
10	Section 7.3 Auxora and Placebo Storage<br>The stability of Auxora and Placebo has been demonstrated to ~~24~~ 36 months when stored at 2 – 8 °C ~~and is being evaluated for longer periods in ongoing studies~~.	Extended stability data has been established
11	Section 8.4 Start of the First Infusion (SFISD)<br>…<br><br>Draw blood sample for PK as soon as possible after completion of the first infusion of study drug (within +2 hours)	Prior language did not provide specificity
12	Section 8.5 24 hours<br>…	Prior language did not provide specificity

#	Nature of change from protocol V 3.0/ March 31, 2022	Rationale
	o Draw blood sample for PK prior to the infusion of the second dose of study drug (within -30 min) - send to central lab.	
13	Section 8.10 Day 30 (±5 days)<br><br>…<br>• For patients that do not return for Day 30 visit and have not withdrawn consent, the Investigator or designee will continue to contact patient by phone to return for on-site visit up to ~~three~~ eight weeks following visit date. Although an out of window visit assessment would be considered a protocol deviation, investigators should attempt to obtain as much as possible all of the Day 30 visit assessments, including the Day 30 CECT.	To be consistent with FDA guidance regarding collecting follow up safety data in all enrolled patients.
14	Section 8.11 Discharge Date<br>…<br>If a patient is admitted to a hospital following discharge but prior to Day 30 visit, Investigator will report the reason for the hospitalization as per serious adverse event reporting guidelines (See Sections 9.2 and 9.7). As the patient is still within the study period, investigators or designee should resume collection of study procedures as per Schedule of Events (See Appendix 8). If patient is not admitted to the hospital where the study is being conducted, investigators are asked to record any procedures (i.e., invasive mechanical ventilation) received while patient was admitted during the study period within an unscheduled procedures form in the eCRF.	Clarify visit assessments to be consistent with FDA guidance regarding collecting follow up safety data in all enrolled patients.
15	Sections 8.12.3 Abdominal Examination<br><br>An abdominal examination will be performed and recorded at Screening to assess if the patient has abdominal guarding or rebound tenderness. For eligibility purposes, an abdominal examination completed as SOC within 12 hours of ~~Screening~~ consent or following consent but prior to randomization may be used for Inclusion Criterion 3b.	Prior language did not provide specificity / ensure appropriate patients are enrolled

#	**Nature of change from<br>protocol V 3.0/ March 31, 2022**	**Rationale**
16	Section 8.12.7 Laboratory Analyses<br><br>At Screening, a lipase, CBC, differential, and platelets, will be performed at the local lab. For eligibility purposes, the results of the CBC collected within 24 hours of consent or following consent but prior to randomization will be used to determine the presence of SIRS (see Section 8.12.13) and a Hematocrit collected as SOC within 12 hours of consent or following consent but prior to randomization may be used for Inclusion Criterion 3c.	Prior language did not provide specificity / ensure appropriate patients are enrolled.
17	Section 8.12.7 Laboratory Analyses<br>…<br>If a site is unable to perform any of the laboratory tests because it is not offered at the site, it will not be performed on patients randomized at the site. ~~The only exception to this would be absolute and/or percent neutrophil and lymphocyte counts.~~	Prior Protocol Amendment missed removing this text. As noted in sentence prior, all protocol-specified laboratory analytes are required unless not offered at the site/institution.
18	Section 8.12.8 PK Analysis<br>Blood samples for PK analysis will be drawn as soon as possible after the completion of the first infusion of study drug (within +2 hours), prior to the start of second infusion of study drug (within -30 min), and at 72 hours (±2 hours) from the SFISD.	Clarify visit assessments / prior language did not provide specificity
19	Section 8.12.12 Contrast-Enhanced CT<br>…<br>If CECT is not available (e.g. medical contraindication to contrast medium used in CECT) and another appropriate imaging is available (e.g. MRI, MRCP), please contact medical monitor to discuss whether local SOC imaging can be used for eligibility and Day 30 purposes.<br><br>CECT contrast protocol may be altered to local SOC. The lack of perfusion of the pancreas on the CECT will be exclusionary. ~~The CECT protocol may be~~	Given no safety issues, flexibility is justified in relation to protocol being implemented within standard of care ER/inpatient hospital stays.<br><br>Prior language edited for clarity

#	Nature of change from protocol V 3.0/ March 31, 2022	Rationale
	~~altered to local SOC.~~ If a CECT had already been performed more than 24 hours but less than 48 hours before Consent, and there is a contraindication to repeating it, the PI should discuss with the medical monitor if the CECT may be used for evaluating the inclusion and exclusion criteria for the study.	
20	Section 8.12.13 Systemic Inflammatory Response Syndrome (SIRS)<br><br>The physician, or appropriately trained designee will determine the presence of SIRS at Screening. The most extreme value for each criterion in the ~~12~~ 24 hours before ~~Screening~~ consent may be used. ~~The respiratory rate prior to the administration of opioids may be used if more than 12 hours before Screening consent after discussion with the medical monitor.~~ SIRS is defined as the presence of at least 2 of the 4 criteria:	Given no safety issues, flexibility is justified in relation to protocol being implemented within standard of care ER/inpatient hospital stays.
21	Section 9.4 Recording Adverse Events<br>…<br>All AEs reported or observed during the study must be followed to resolution. Or, if not fully resolved, until the condition has stabilized, the patient dies, withdraws consent, the site is closed or CalciMedica ends the trial whichever is first.	Prior language did not provide specificity
22	Section 9.7 Reporting of Serious Adverse Events<br><br>…<br><br>In addition, if any of the events below, ~~the following SAEs~~ which ~~that~~ are commonly observed and thus 'anticipated' in this patient population, are reported as a SAE, they will not be reported to the regulatory authority as individual expedited reports except in unusual circumstances or if required by local regulations:<br><br>• Hypoxemia and acute respiratory distress syndrome<br>• Oliguria and acute kidney injury	As study is global, protocol needs to be consistent for all regulatory authority requirements.

#	**Nature of change from protocol V 3.0/ March 31, 2022**	**Rationale**
	• Hypotension and shock<br>• Bacteremia<br>• Pneumonia<br>• Obtundation<br>• Splanchnic venous thrombosis<br>• Abdominal compartment syndrome<br>• Gastroparesis and ileus<br>• Local complications of the pancreas and peripancreatic tissue<br>• Percutaneous insertion of abdominal drains<br>• Endoscopic necrosectomy<br>• ERCP<br>• Abdominal surgery including but not limited to cholecystectomy, pancreatic necrosectomy, and placement of pancreatic drains	
23	Section 11.10 Protocol ~~Violations and~~ Deviations<br><br>The PI or designee must document any protocol deviation ~~or violation~~. Reporting of protocol deviations ~~and violations~~ to the appropriate IRB/EC is the responsibility of the PI and must follow the applicable IRB/EC guidelines. A protocol deviation is any change, divergence, or departure from the study design or procedures defined in the protocol.<br><br>As referenced in ICH E3, the definition of important protocol deviations for a particular trial is determined in part by study design, the critical procedures, study data, subject protections described in the protocol, and the planned analyses of study data. In keeping with the flexibility of the guidance, sponsors can amend or add to the examples of important deviations provided in ICH E3 in consideration of a trial's requirements.<br><br>~~Major protocol deviations are a subset of protocol deviations that might significantly affect the completeness, accuracy, and/or reliability of the study data or that might significantly affect a subject's rights,~~	Support ICH E3 consistency

#	Nature of change from protocol V 3.0/ March 31, 2022	Rationale
	~~safety, or well-being~~. For the purposes of this study, an important protocol deviation is one that has an impact on subject safety, may substantially alter risks to subjects, may have an effect on the integrity of the study data, or may affect the subject's willingness to participate in the study.	
24	Appendix 1: Definition of Organ Failure and Sepsis<br><br>The definition of organ failure encompasses three organ systems, respiratory system, renal system, and cardiovascular system.<br><br>For the respiratory system, organ failure will be defined as:<br><br>• $PaO_2/FiO_2 \leq 300$ determined by arterial blood gas or imputed using pulse oximetry, or<br><br>• The use of high flow nasal cannula, non-invasive or invasive mechanical ventilation<br><br>…	To reflect changing standard of care in ventilating patients with respiratory failure
25	Appendix 8: Schedule of Events<br><br>Added footnote to Presence of SIRS<br>Added footnote to Randomize Patient<br>Added footnote to Administer Study Drug<br><br>Footnote j (previously footnote g): Blood samples for PK analysis will be drawn as soon as possible after the completion of the first infusion of study drug (within +2 hours), prior to the start of second infusion of study drug (within -30 min), at 72 hours (±2 hours) from the SFISD, and at the Day 30 visit.<br><br>Renumbered footnote(s) references to align with changes noted above.	Updated to reflect clarifications made in Sections 5.3, 8.4, 8.5, 8.12.7, 8.12.8 and 8.12.13.

Statistical Analysis Plan

Protocol CM4620-203

A Randomized Double-Blind, Placebo Controlled Dose-Ranging Study of Auxora in Patients with Acute Pancreatitis and Accompanying Systemic Inflammatory Response Syndrome (CARPO)

PREPARED BY:

NAME: Jeffrey Zhang, PhD.

POSITION: Principal Statistician, Princeton Pharmatech

VERSION: Final 1.0

DATE: 10 June 2024

APPROVAL FORM

STATISTICAL ANALYSIS PLAN

**Protocol No.**	CM4620-203
**Protocol Title**	**A Randomized Double-Blind, Placebo Controlled Dose-Ranging Study of Auxora in Patients with Acute Pancreatitis and Accompanying Systemic Inflammatory Response Syndrome (CARPO)**
**Version**	Final 1.0
**Date**	10 June 2024
**Author**	Jeffrey Zhang, PhD, Principal Statistician, Princeton Pharmatech

DocuSigned by:
Jeffrey Zhang, PhD
Signer Name: Jeffrey Zhang, PhD
Signing Reason: I approve this document
Signing Time: 6/10/2024 | 8:14:49 AM PDT
17A8AB3000DE4D7C97C78888DF573F10

6/10/2024

Signature Date

APPROVED BY:

DocuSigned by:
Sudarshan Hebbar
Signer Name: Sudarshan Hebbar
Signing Reason: I approve this document
Signing Time: 6/10/2024 | 8:10:56 AM PDT
732B16E369D74416B6B6AE164D8A9C32

6/10/2024

Sudarshan Hebbar, MD
Chief Medical Officer, CalciMedica, Inc.

Date

TABLE OF CONTENTS

1. INTRODUCTION....6

2. STUDY OBJECTIVES....6
   2.1 Primary Objectives:....6
   2.2 Secondary Objective:....6

3. STUDY DESIGN....6
   3.1 Overall Study Design....6
   3.2 Schedule of Study Procedures....8

4. SAMPLE SIZE....9

5. STUDY ENDPOINTS....10
   5.1 Efficacy Endpoints:....10
   5.2 Safety Endpoints:....10
   5.3 Proof of Concept Composite Efficacy Endpoint:....10

6. ANALYSIS POPULATION(S)....11
   6.1 Efficacy and Safety....11

7. TIMINGS OF ANALYSES....11
   7.1 Final Analysis....11

8. STATISTICAL ANALYSIS....11
   8.1 General Considerations....11
      8.1.1 Number of Digits to Report....11
      8.1.2 Significance Level and Confidence Interval....11
      8.1.3 Descriptive Statistics Values to Calculate....12
      8.1.4 Derived Variables....12
      8.1.5 Missing Data for Safety Endpoints and Concomitant Medications....13
      8.1.6 Study Visits....13
      8.1.7 Pooling of Sites....13
   8.2 Subject Disposition and Withdraws....13
   8.3 Demographic and Baseline Characteristics....13
   8.4 Concomitant Medications....14
   8.5 Study Medication Exposure....14
   8.6 Primary Endpoint Analysis....14
      8.6.1 Generalized Multiple Comparisons and Modelling (gMCP-Mod)....14
      8.6.2 Definition of Estimand....16
      8.6.3 Main Analytical Approach....17
      8.6.4 Sensitivity Analysis....18
      8.6.5 Supplementary Analyses....18

8.7 Secondary Endpoint Analyses 18
8.7.1 Percentage of Patients who Tolerated Solid Food at 48, 72, and 96 Hours from the SFISD and at Discharge 18
8.7.2 Time to Medically Indicated Discharge 19
8.7.3 Length of Stay in the Hospital 20
8.7.4 Re-hospitalization for acute pancreatitis (AP) Through Day 30 20
8.7.5 Change in Severity of AP by CTSI Score from Screening to Day 30 21
8.7.6 Development of pancreatic necrosis ≥30% and >50% 21
8.7.7 Persistence of SIRS ≥48 Hours After the SFISD 21
8.7.8 Incidence, Severity, and Duration of Organ Failure 22
8.7.9 Mortality by Day 30 23
8.7.10 Change in Pain Score 23
8.8 Exploratory Endpoints 23
8.9 Proof-of-Concept Composite Endpoint Analysis 24
8.10 Safety Endpoint Analyses 27
8.10.1 Adverse Events 27
8.10.2 Vital Signs 28
8.10.3 Laboratory Analyses 28
8.10.4 Arterial Blood Gas 29
8.10.5 SpO_2/FiO_2 29
SpO_2 and FiO_2 parameters, and imputed P/F ratios will be listed 29
8.11 PK Assessments 29
8.12 Subgroup Analyses 29

9. INTERIM ANALYSIS 29
9.1 Interim Safety Analyses 29

10. VALIDATION 30

11. PROGRAMMING AND DATA PRESENTATION CONVENTIONS 30

12. SUMMARY OF CHANGES TO STATISTICAL ANALYSIS PLAN VERSION 1.0 30

13. SUMMARY OF CHANGES TO PROTOCOL PLANNED ANALYSIS 30

14. BIBLIOGRAPHY 30

TABLE OF TABLES

Table 1. Schedule of Assessments 8
Table 2. Number of Decimal Places (DP) 11
Table 3. Summary of changes from statistical analysis plan version 1.0 30

LIST OF ABBREVIATIONS

Abbreviation	Definition
AE	Adverse event
AP	Acute pancreatitis
ARDS	Acute respiratory distress syndrome
BLQ	Below the limit of quantification
BMI	Body mass index
CI	Confidence interval
COVID-19	Disease from infection with coronavirus 2019 or SARS-CoV-2
CM	Concomitant medication
CRO	Contract Research Organization
CS	Clinically significant
CSR	Clinical study report
DP	Decimal places
eCRF	Electronic case report form
ECMO	Extracorporeal membrane oxygenation
IDMC	Independent data monitoring committee
IWRS	Interactive web response system
LAR	Legally authorized representatives
LOCF	Last observation carried forward
MedDRA	Medical Dictionary for Regulatory Activities
PCS	Potentially clinically significant
PK	Pharmacokinetic(s)
PT	Preferred term
QC	Quality control
SAE	Serious adverse event
SAP	Statistical analysis plan
SD	Standard deviation
SFISD	Start of first infusion of study drug
SOC	System organ class
TEAE	Treatment-emergent adverse event
TFLs	Tables, figures and listings
ULN	Upper limit of normal
WHO	World health organization

1. INTRODUCTION

This statistical analysis plan (SAP) is based on the CM4620-203 Protocol version 4 dated 05 October 2023. The plan covers statistical analysis, tabulations and listings of safety and efficacy data to assess the safety and efficacy of Auxora compared to placebo in patients with acute pancreatitis and accompanying systemic inflammatory response syndrome (CARPO).

The statistical analyses and production of the outputs described in the SAP will be conducted and quality control (QC) reviewed, using SAS version 9.4 or higher.

2. STUDY OBJECTIVES

2.1 Primary Objectives:

- To assess the dose response and efficacy of three different dose levels of Auxora in patients with acute pancreatitis and accompanying SIRS;
- To assess the time to medically indicated discharge in patients who are responders to early tolerance of solid food intake versus non-responders.

2.2 Secondary Objective:

- To assess the safety and tolerability of varying doses of Auxora in patients with acute pancreatitis and accompanying SIRS

3. STUDY DESIGN

3.1 Overall Study Design

This double blind, randomized, placebo-controlled study will evaluate the efficacy, safety, and tolerability of three different dose levels of Auxora in patients with acute pancreatitis and accompanying SIRS.

Approximately 216 patients will be randomized 1:1:1:1 into one of 4 groups using a computer generated randomization scheme accessed through an interactive voice/web response system (IXRS). Randomization will be first stratified by sex (male or female) and then by risk for organ failure in the sex subgroups (higher or lower). Higher risk for organ failure is defined by the presence of both an elevated hematocrit (HCT ≥44% for men or ≥40% for women) and hypoxemia (imputed PaO_2/FiO_2 ≤360). Lower risk for organ failure is defined by the absence of either or both an elevated hematocrit and hypoxemia. The PaO_2/FiO_2 will be determined using an arterial blood gas or imputed using pulse oximetry.

All patients will have received a Screening CECT/MRI of the abdomen/pancreas before being randomized into the study. CECTs/MRIs performed as standard of care may be used as the Screening CECT/MRI but must have been performed in the 24 hours before Consent or after Consent and before Randomization.

The Start of First Infusion of Study Drug (SFISD) should occur within 8 hours of the patient or LAR providing informed consent. Patients randomized to Group 1 will receive 2.0 mg/kg (1.25 mL/kg) of Auxora intravenously every 24 hours (±1 hour) for a total of three doses. Patients

randomized to Group 2 will receive 1.0 mg/kg (0.625 mL/kg) of Auxora intravenously every 24 hours (±1 hour) for a total of three doses. Patients randomized to Group 3 will receive 0.5 mg/kg (o.3125 mL/kg) of Auxora intravenously every 24 hours (±1 hour) for a total of three doses. Patients randomized to Group 4 will receive emulsion without any active pharmaceutical ingredient (placebo). Patients in Group 4 will receive one of three randomly assigned dose volumes, 1.25 mL/kg, 0.625 mL/kg, or 0.3125 mL/kg, which will be administered intravenously every 24 hours (±1 hour) for a total of three doses. The dosing will be based on actual body weight obtained at the time of hospitalization or screening for the study. As described in the pharmacy manual, the upper limit of the volume of Auxora and volume of placebo that will be administered will be 156.25 mL. The sponsor, investigators and patients will be blinded to the assigned group. In the event of a medical emergency, investigators will be able to receive the treatment assignment if required to provide optimal care of the patient.

For all 4 groups, a study physician or appropriately trained delegate will perform assessments at screening, at the baseline assessment, immediately prior to the SFISD, and then every 24 hours until 240 hours after the SFISD, or until discharge if earlier. If patients remain hospitalized at Day 12, assessments will then be performed every 48 hours starting on Day 12 until Day 28, or until discharge if earlier. Patients discharged from the hospital before Day 25 will return at Day 30 (+5 days) to perform the Day 30 assessments. If patients are discharged on Days 25-29, the Day 30 assessments may be performed prior to discharge.

Patients will receive another CECT of the abdomen/pancreas at the Day 30 (±5 days) visit. All CECTs performed as standard of care after randomization and before the Day 30 CECT will also be captured. A blinded central reader will read the Screening, Day 30, and any standard of care CECTs obtained between randomization and 30 days.

Patients will complete the modified American Neurogastroenterology and Motility Society Gastrointestinal Cardinal Symptom Index Daily Diary (mGCSI-DD) worksheet at the baseline assessment, at 96 hours, 168 hours, Day 14 and Day 21 (for patients who remain hospitalized on these days), on the day of discharge, and daily at bedtime after discharge until the Day 30 visit. Patients who are discharged on Days 25-29 will not complete the mGCSI worksheet after discharge.

It is recommended that all patients randomized in the study should receive care consistent with the 2018 American Gastroenterological Association (AGA) Institute Technical Review of the Initial Medical Management of Acute Pancreatitis. Patients should receive local standard of care (SOC) for the management of other medical conditions.

In patients with acute pancreatitis, the AGA strongly recommends early oral feeding (within 24 hours) rather than keeping the patient nil per mouth (Nil per Os, NPO). Patients randomized into the study, therefore, will be offered a low fat, ≥500-calorie solid meal at each mealtime after the infusion of the first dose of study drug if alert and not on mechanical ventilation. If the patient does not wish to eat the solid meal when offered or is unable to tolerate the solid meal, they should then be offered a liquid meal. The same approach should occur at each subsequent mealtime. When patients eat a solid meal, it should be recorded if they ate ≥50% of the meal and

if they either vomited or experienced an increase in abdominal pain in the two hours after eating a meal.

It is also recommended that all patients randomized in the study should not be discharged from the hospital until solid food is tolerated, abdominal pain has resolved or been adequately controlled, and there is no clinical evidence of infection. Tolerating solid food is defined as eating ≥50% of a low fat, ≥500-calorie solid meal without an increase in abdominal pain or vomiting. If the patient is not tolerating either solid or liquid meals, tube feedings should be considered.

All protocol required laboratory testing, except biomarker and PK samples, will be performed at the local laboratory. Results from the biomarkers and PK blood samples collected as part of the protocol and being tested at a central lab will not be available to assist the PI or treating physician in managing the patient.

3.2 Schedule of Study Procedures

Table 1. Schedule of Assessments

	Screening	If Eligible, Baseline Assessment	R	SFISD	24 (±1) Hours[i]	48 (±1) Hours[i]	72 (±2) Hours	96, 144, 192, 240 (±4) Hours[h,k]	120, 168, 216 (±4) Hours[h,k]	Days 12-28 Q48 (±4) Hours[h,k]	Day 30 (±5) Days
Informed Consent	X										
Demographics	X										X[l]
Medical History	X										
List of Medications	X										
Concomitant Meds				X	X	X	X	X	X		X
Height		X									
Weight		X					X				X
Vital Signs	X				X	X	X	X	X		X
Abdominal Exam	X										
PNRS		X			X	X	X	X	X		X
Serum Lipase	X[a]										
CBC+diff+Platelets	X[a]				X[a]	X[a]	X[a]		X[a]		X[a]
Presence of SIRS[b]	X										
SpO_2+FiO_2		X			X	X	X	X	X		X
CECT of Pancreas[c]	X										X
Serum Pregnancy Test	X[a]										
Randomize Patient[d]			X								
Serum Chemistries		X[a]			X[a]	X[a]	X[a]		X[a]		X[a]
Serum Procalcitonin		X[a]			X[a]	X[a]	X[a]		X[a]		X[a]
Serum Biomarker[e]		X			X	X	X		X		X
Urine Biomarker[e]		X			X	X	X		X		X
Administer Study Drug				X	X[i]	X[i]					

PK sample[e]				X[j]	X[j]		X[j]				X[j]
Medically Indicated Discharge Criteria[f]					X	X	X	X	X	X	X
Enhanced Recovery Strategy[g]				X	X	X	X	X	X	X	X[m]
mGCSI-DD[h]		X[h]			X[h]					X[h]	X[h]
AE/SAE Evaluation			X	X	X	X	X	X	X	X	X
Telephone Contacts, as needed		Following patient discharge, Investigator or designee should contact patient at least once weekly to confirm diary completion, Day 30 visit schedule and assess for AE/SAEs.									

AE = adverse event; CECT = contrast-enhanced computed tomography; PK = pharmacokinetics; mGCSI-DD = Modified American Neurogastroenterology and Motility Society Gastrointestinal Cardinal Symptom Index Daily Diary; PI = Principal Investigator; PNRS = Pain Numeric Rating Scale; SAE = serious adverse event; SFISD = Start of First Infusion of Study Drug

a. Blood sample for serum pregnancy test, serum lipase and CBC+diff+Platelets at Screening will be analyzed at local laboratory. May use results obtained as standard of care if blood drawn in the previous 12 hours prior to consent or following consent but prior to randomization. Following screening, all protocol-required blood samples, except as noted in Footnote “e”, will be analyzed at local laboratory. Baseline blood samples including biomarkers may be performed on blood drawn in the previous 12 hours of randomization. For all later assessment times, blood drawn in the previous 12 hours maybe used (except PK).
b. The most extreme value for each criterion in the 24 hours before consent or following consent but prior to randomization may be used.
c. A CECT already performed in the 24 hours prior to the patient or LAR providing informed consent may be used as the Screening CECT. In addition to the Screening and the Day 30 visit CECTs, CECTs performed as standard of care after randomization and to 25 days after SFISD will be captured and submitted for central reading. If the CECT is performed 25 to 29 days after SFISD, it will substitute for the Day 30 visit CECT scan and will be captured.
d. Hematocrit collected as standard of care within 12 hours of consent or following consent but prior to randomization may be used for Inclusion Criterion 3c.
e. Biomarker blood and urine samples and blood samples for PK will be analyzed at central laboratory. If site or patient does not participate in these assessments this will be documented at start of study and will not be considered a protocol deviation.
f. Determine if patient had met the medically indicated discharge criteria within the prior 24- or 48-hour visit assessment.
g. Patients will be offered a low fat, ≥500-calorie solid meal at each mealtime after the infusion of the first dose of study drug until discharge if alert and not on mechanical ventilation, or if not NPO for a planned surgery/medical procedure, or if not NPO because of an acute medical condition. If the patient does not wish to eat the solid meal when offered or is unable to tolerate the solid meal, they should then be offered a liquid meal. Determine for each meal if the patient ate ≥50% of the meal without vomiting or having abdominal pain in the two hours after the meal.
h. The mGCSI-DD score will be completed by all eligible patients at the baseline assessment and on the day of discharge, which will vary by patient as shown in schedule above. In addition, if the patient remains hospitalized at 96 and/or 168 hours and/or at Day 14 and/or Day 21 patient should be requested to record their symptoms at bedtime using the mGCSI-DD worksheet. Patients will be trained on completing the mGCSI-DD worksheet. Upon discharge, patient will record the mGCSI-DD score daily before bedtime until the Day 30 visit.
i. In the scenario where the PI or treating physician wants the infusion(s) to begin earlier than the 24 +/- 1 hour window, the PI or treating physician should contact the medical monitor to discuss whether it is appropriate to begin infusion earlier (up to 6 hours earlier) than the 24 +/- 1 hour protocol window.
j. Blood samples for PK analysis will be drawn as soon as possible after the completion of the first infusion of study drug (within +2 hours), prior to the start of second infusion of study drug (within -30 min), at 72 hours (±2 hours) from the SFISD, and at the Day 30 visit. If the patient is discharged after the third infusion of study drug and prior to 72 hours from the SFISD, the blood sample will be drawn prior to discharge.
k. Study assessments continue daily, or every other day, as noted in schedule of events until discharge or the Day 30 visit. All patients return for Day 30 assessments if discharged earlier than Day 25.
l. At the Day 30 visit (or at the time of death if before Day 30), record the Investigator’s opinion on the etiology of the episode of AP.
m. Only patients who remain hospitalized at Day 30.

4. SAMPLE SIZE

A sample size of 216 patients will be randomized into four groups on a 1:1:1:1 basis, resulting in 54 patients randomized into each group. This sample size will provide 86% power for testing the difference in two populations having a median length of time of 72 and 144 hours, respectively, to tolerating solid food. This sample size will provide 80% power with a two-sided alpha of 0.05 to detect a 45% response rate for tolerating solid food in an Auxora dose group versus a 20% response rate in the placebo group in the 72 hours after completion of the first study drug infusion.

5. STUDY ENDPOINTS

5.1 Efficacy Endpoints:

- Primary endpoint
  - Time to solid food tolerance
- Secondary endpoints:
  - Solid food tolerance at 48 hours, 72 hours, and 96 hours after the Start of First Infusion of Study Drug (SFISD) and at discharge
  - Time to medically indicated discharge
  - Length of stay in the hospital
  - Length of stay in the intensive care unit (ICU) for patients admitted to the ICU
  - Re-hospitalization for acute pancreatitis (AP) by Day 30
  - Change in severity of AP by CTSI score from screening to Day 30
  - Development of pancreatic necrosis ≥30% and >50%
  - The persistence of SIRS ≥48 hours after the SFISD
  - Incidence, severity, and duration of organ failure
  - Mortality by Day 30
  - Change in pain score and opioid use
- Exploratory endpoints:
  - Development of infected pancreatic necrosis
  - Development of sepsis
  - Hospital procedures for the management of pancreatic necrosis
  - Change in GCSI-DD score
  - Change in albumin
  - Change in absolute neutrophil count (ALC)/absolute lymphocyte count (ALC) ratio
  - Change in IL-6 levels
  - Change in urine neutrophil gelatinase-associated lipocalin (NGAL) levels

5.2 Safety Endpoints:

- The incidence of TEAEs and SAEs
- The intensity and relationship of TEAEs and SAEs
- Clinically significant changes in vital signs and safety laboratory results

5.3 Proof of Concept Composite Efficacy Endpoint:

The proof-of-concept efficacy analysis uses a hierarchical combination applying the method of Finkelstein-Schoenfeld (Finkelstein 1999) to:

- All-cause mortality at Day 30 Visit, and
- Persistent severe respiratory failure through Day 30 Visit, and
- Pancreatic necrosis ≥30% through Day 30 Visit, and
- Rehospitalization for AP through Day 30 Visit, and
- Time to solid food tolerance within 30 Days of the study

6. ANALYSIS POPULATION(S)

6.1 Efficacy and Safety

The efficacy analyses will be performed on the Modified Intent-to-treat Population (MITT) consisting of all randomized patients who receive any amount of study drug. The safety analyses will be performed on the MITT data set.

7. TIMINGS OF ANALYSES

7.1 Final Analysis

The final analysis will be conducted when all randomized patients complete the study.

8. STATISTICAL ANALYSIS

8.1 General Considerations

The statistical evaluation will be performed using SAS® Version 9.4 or later.

In general, descriptive statistical methods will be used to summarize the data from this study. Unless stated otherwise, the term "descriptive statistics" refers to number of patients (n), mean, median, standard deviation (SD), standard error (SE), minimum and maximum for continuous data, interquartile ranges for nonparametric data, and frequencies and percentages for categorical data. Safety and efficacy summaries will be performed on the efficacy and safety analysis sets, respectively.

8.1.1 Number of Digits to Report

Table 2. Number of Decimal Places (DP)

Statistic	Specification	Apply to
Minimum, maximum	Same number of DPs as the data provided in the datasets	All original, i.e., non-derived, data provided in the datasets
mean, median, confidence intervals	One more DP than the raw data	All
SD, SE	Two more DP than the raw data	All
Percentages	1 DP	All
p-values	3 DP	All
Odds Ratio	2 DP	All
Hazard Ratio	3 DP	All

8.1.2 Significance Level and Confidence Interval

The statistical tests will be performed as two-sided with significance level of 5% unless specified otherwise. The confidence intervals will be determined with a confidence level of 95%.

8.1.3 Descriptive Statistics Values to Calculate

Where appropriate, variables will be summarized descriptively (frequency and percent will be summarized for categorical variables; mean, SD, median, minimum, and maximum will be presented for continuous variables) by study visit, and by treatment group.

The denominator for the percentages will be the total number of subjects in the treatment group and Analysis Set being presented, unless otherwise specified (e.g., on some occasions, percentages may be calculated based on the total number of subjects with available data at a particular visit and/or time point).

8.1.4 Derived Variables

8.1.4.1 Definition(s) of Baseline(s)

Unless otherwise specified, the baseline values are the last available assessment before or at the time of the SFISD. If multiple measurements are recorded on the same day, the last measurement recorded prior to or at the time of the SFISD will be used as the baseline value. If these multiple measurements occur at the same time or time is not available, then the average of these measurements (for continuous data) or the worst among these measurements (for categorical data) will be considered as the baseline value.

8.1.4.2 Study Time

For patients enrolled in the study, the first dose of study medication will be administered at study hour 0. The study time in hours for an event is defined as event date and time – date and time of SFISD. The study day is defined as event date – date of SFISD + 1 day.

8.1.4.3 Definition(s) of (Percent) Change from Baseline(s)

For numerical variables, change from baseline will be calculated as the post-baseline value minus the baseline value. If percent change from baseline is required, then percent change from baseline will be calculated as the change from baseline divided by the baseline value, multiplied by 100. If baseline value cannot be determined for a particular variable, the change from baseline and percent change from baseline will not be calculated.

8.1.4.4 Adverse Events

Treatment-emergent AEs (TEAEs) are defined as AEs that started or worsened in severity on or after the SFISD. All adverse events will be coded from the reported term using the Medical Dictionary for Regulatory Activities (MedDRA) version 23.0 or the later version.

8.1.4.5 Concomitant Medications

All concomitant medications will be coded using the WHO drug dictionary (B3 WHO Drug Dictionary Enhanced – Sep 2019 or the later version).

8.1.4.6 Lab Values Below/Above the Limit of Quantitation Deriving

The lab values below the limit of quantification (BLQ) are collected in the form like "< 2" in the clinical database. In this case the numeric value of the lab is a missing value. The cut-off value '2' will be used to impute the missing value for this case. Similarly, for the lab values above the limit of quantification, the cut-off value will be used for the numeric value of the lab. Local labs are used in this study and all lab parameters will be standardized to common SI units.

8.1.5 Missing Data for Safety Endpoints and Concomitant Medications

Missing data will not be imputed in the summary of safety endpoints at each time point. Imputation of missing or incomplete dates will be performed on AEs and concomitant medications (procedures) conservatively for determining the timing relative to the dose of study product unless otherwise specified. Partial or missing dates will be listed as recorded in the electronic case report form (eCRF).

8.1.6 Study Visits

The study summary and analysis will be based on the eCRF visits.

8.1.7 Pooling of Sites

Data collected from all sites will be pooled together for the analysis.

8.2 Subject Disposition and Withdraws

The number of patients who enter screening will be summarized, and the percentage of these patients who fail to meet entry criteria will be reported for total subjects. Screen failures will be summarized in total and by each reason for screen failure.

Subject disposition will be summarized for patients' completion status of study treatment, status of outcome/discharge, and corresponding discontinuation reasons in tables for each treatment group and then total subjects. The data listing for subject discontinuation will be generated.

8.3 Demographic and Baseline Characteristics

Patient demographic and baseline characteristics will be summarized by mean, SD, median, minimum, and maximum for continuous variables; interquartile ranges for nonparametric data, and by counts and percentages for categorical variables. Summaries will be provided separately for each treatment group and then total subjects. The following subject demographic and baseline characteristic are summarized. The demographic variables consist of age, age category (18-39, 40-64, 65+), sex, ethnicity, and race. Baseline characteristics include height, weight, body mass index (BMI), time from symptom onset to randomization in days, the number of SIRS criteria met at Screening: two versus greater than two, number of patients at high risk for organ failure [defined by the presence of both an elevated hematocrit (HCT ≥44% for men or ≥40% for women) and hypoxemia (imputed PaO_2/FiO_2 ≤360)], number of patients with baseline hematocrit value and category (<44%, >=44% for males; <40%, >=40% for females), the number of patients with abdominal guarding or rebound tenderness, and the number of patients

with CECT at Screening with peripancreatic fluid or pleural effusion by central reading. The Modified Intent to Treat (MITT) Analysis Set will be used for the summaries.

Other baseline variables such as relevant medical history or other medically relevant criteria, such as the cause of acute pancreatitis, will also be included in the baseline tables.

8.4 Concomitant Medications

Concomitant medication is defined as any medication, other than study medication, which is taken on or after the start day of treatment. The concomitant medication data will be coded using World Health Organization Drug Dictionary (B3 WHO Drug DDE – Sep 2019 or the later version). The number and percentage of subjects taking selected concomitant medications will be summarized overall and by Anatomic Therapeutic Chemical (ATC) Classification level 2 terms, preferred drug names and treatment groups. A separate listing for selected concomitant medications will also be provided.

8.5 Study Medication Exposure

The number of treatment doses, delayed doses, reduced doses, and interrupted doses per patient of double-blind study medication will be summarized in the table for the MITT population. The percentage of delayed doses, reduced doses, and interrupted doses per patient and per total number doses will be summarized.

8.6 Primary Endpoint Analysis

8.6.1 Generalized Multiple Comparisons and Modelling (gMCP-Mod)

The primary objective of this study is to demonstrate a dose-response effect of Auxora compared to placebo of the primary endpoint time to solid food tolerance through Day 30. To demonstrate the dose-response relationship between treatment with Auxora compared to placebo for the primary endpoint, the primary analysis will be based on a generalized Multiple Comparisons and Modeling (gMCP-Mod) analysis [Bretz et al., 2005; Pinheiro et al., 2014]. This analysis tests for a dose-response relationship, allowing for uncertainty in the dose-response relationship through inclusion of contrasts from multiple pre-specified candidate models assessing dose-response.

The gMCP-Mod procedure extends the MCP-Mod methodology to non-continuous endpoints. This is accomplished by decoupling the dose-response model from the expected response through the use of an ANOVA-style parameterization of the dose-response parameter. For the time-to-event endpoint used in this study, a stratified Cox regression of the time to solid food tolerance at each dose level will be used, stratified by the CRF recorded sex (male or female) and then by risk for organ failure based on CRF data in the sex subgroups (higher or lower). After this first step, the mean estimates of log hazard ratio from the Cox regression model and the corresponding covariance matrix, both of which are on the log scale, will then be used during the rest of the gMCP-Mod procedure. Point estimates from the selected models will be displayed on the ratio scale in all statistical outputs.

The gMCP-Mod (Pinheiro et al., 2014) will be employed in a three-step approach in the manner described below:

- Step 1: Conventional Step – Hazard Ratio Between Each Dose Versus Placebo

  The data are analyzed using stratified Cox regression to obtain treatment effects and corresponding variance covariance matrix. The Cox regression will be stratified by sex (male or female) and the risk for organ failure (higher or lower).

  The estimated treatment hazard ratio and the associated 95% confidence intervals will be presented for the treatment contrast of each dose of Auxora versus placebo

- Step 2: Proof-of-concept Step – Candidate Models and Multiple Testing Procedure

  The null hypothesis of flat dose-response relationship as compared to placebo for the primary efficacy endpoint will be tested at a significance level of one-sided 15% against the alternative hypothesis of a non-constant dose-response curve using a multiple contrast test as described in the gMCP-Mod methodology (Pinheiro et al., 2014). The contrast test statistic is a linear combination of the optimal contrast coefficients corresponding to the candidate dose-response curve with the hazard ratio at each individual dose obtained from Step 1 (conventional step).

  The contrast coefficients are chosen to maximize the power to detect pre-specified candidate models. There are two candidate models that will be considered for the primary analysis with the optimal contrasts shown in following table. These optimal contrasts will be updated using the expected model means from the candidate set of the two models and the estimated variance-covariance matrix from the collected data. The updated contrast coefficients will be described in the final CSR.

  The optimal contrast based on Candidate models for Auxora dose for log(Hazard Ratio):

Model	Contrast Coefficients for Dose		
Dose	0.5 mg/kg	1.0 mg/kg	2.0 mg/kg
Emax	0.508	0.588	0.629
sigEmax	-0.611	0.488	0.623

  Note: Candidate model of Emax model is based on the assumption of parameter of ED50=0.026 mg/kg. Candidate model of sigEmax mode is based on the assumption of parameters of ED50=0.707 mg/kg and h=6.34.

  The proof of concept (PoC) is established if at least one dose-response model is statistically significant, thereby rejecting the null hypothesis of a flat dose-response curve and indicating a benefit of Auxora over placebo.

  The global test decision is based on the maximum of the contrast test statistics. A critical value q controlling the type I error rate can be derived from the fact that the contrast test statistics approximately follow a multivariate normal distribution and that their maximum

follows the distribution of the maximum of a multivariate normal distribution. If the maximum contrast test statistic exceeds the critical value q, the overall null hypothesis of a flat dose-response curve is rejected, and one proceeds to characterize the dose-response efficacy relationship.

For each candidate dose-response curve, the test statistics and corresponding adjusted p-values will be presented.

Once proof of concept has been established, then subsequently, it will be tested whether at least one dose of Auxora achieves time to solid food tolerance superior to placebo (i.e., the maximum anticipated effect of Auxora for achieving the primary endpoint, based on the best fitting monotonic dose-response model).

- Step 3: Dose-finding Step – Dose-Response Model Fitting

  Once the significance of a dose-response signal is established, the dose-response profile and the target dose can be estimated using the best-fitting dose-response model. All significant dose-response models will be re-fitted to the data without any parameter assumptions to generate a set of new estimates of the model parameters from the data. The final dose-response model will be obtained via the model with the smallest Akaike Information Criterion (AIC) (the smaller the AIC value the better the model fit). Estimates for the dose groups will be calculated and will be based on the final dose-response model.

  The dose-response curve estimate with the model-based two-sided 95% confidence interval will be presented graphically. In addition, the plot will include the mean responses from the Cox Regression and the associated 95% confidence intervals for each of the studied dose groups.

The target dose(s) suggested by gMCP-Mod based on time to solid food tolerance serves as an important consideration in dose selection. However, the final dose selection will be determined based on the following:

- Efficacy of doses based on the totality of efficacy data
- Safety endpoints including AEs, and SAEs

8.6.2 Definition of Estimand

Time to solid food tolerance through the Day 30 is the primary endpoint.

The primary estimand corresponding to the primary endpoint is defined as:

1. Treatment: 2.0 mg/kg (1.25 mL/kg), 1.0 mg/kg (0.625 mL/kg), or 0.5 mg/kg (0.3125 mL/kg), every 24 hours (±1 hour) for three consecutive days for a total of three infusions.
2. Population: Patients with Acute Pancreatitis and Accompanying Systemic Inflammatory Response Syndrome
3. Variable: Time to solid food tolerance as the number of hours from the date and time of the SFISD for the patient to the first date and time the patient receives a solid meal that is

tolerated, which is defined as eating ≥50% of a low fat, ≥500-calorie solid meal without an increase in abdominal pain or vomiting within the two hours of mealtime.

4. Population level summary: hazard ratio using stratified Cox Regression

Intercurrent events under consideration: 1) prohibited immunosuppressive medications or immunotherapies, 2) treatment discontinuation, and 3) death.

The null and alternative hypotheses are:

(1) Null hypothesis H0: Time to solid food tolerance curves are the same between Auxora and placebo

(2) Alternative hypothesis H1: Time to solid food tolerance curves are different between Auxora and placebo

8.6.3 Main Analytical Approach

Time to solid food tolerance through Day 30 visit will be analyzed based on the Generalized Multiple Comparison Modelling (gMCP-Mod) approach described in Session 8.6.1.

Time to solid food tolerance is defined as the number of hours from the date and time of the SFISD for the patient to the first date and time the patient receives a solid meal that is tolerated. If the mealtime was not recorded, the mealtime will be estimated using the middle time of the meal serving interval.

Tolerating a solid meal is defined as eating ≥50% of a low fat, ≥500-calorie solid meal without an increase in abdominal pain or vomiting within the two hours of mealtime. In addition, to meet the definition, all subsequent meals during the hospitalization should not show an increase in abdominal pain or vomiting within the two hours of mealtime. If a procedure that could impact food tolerance is performed after a solid meal is initially tolerated that results in subsequent meals not being tolerated, the time of the initial tolerated meal will be considered in the calculation of the time to solid food tolerance. The procedures that impact food tolerance and/or its assessment are defined as (1) a procedure that requires a period of nil by mouth (2) a procedure and/or treatment that causes gastrointestinal dysfunction e.g., cholecystectomy, necrosectomy. The procedures performed after the initial food tolerance will be reviewed before the database lock to identify the procedures that could impact the time to the tolerated solid food. If the subject was not discharged from the hospital during the study and did not tolerate solid food, the time to solid food tolerance will be censored at the latest date and time of the hospital stay.

If a patient is discharged home without tolerating solid food, the daily record of the modified American Neurogastroenterology and Motility Society Gastrointestinal Cardinal Symptom Index Daily Diary (mGCSI-DD) at or after hospital discharge will be used in the calculation of the time to solid food tolerance. The patients who did not tolerate solid food while hospitalized will be considered as tolerating solid food at or after hospital discharge at 8am on the first of three consecutive days where the following 4 criteria are met for the mGCSI-DD:

- none for vomiting
- none or mild for nausea
- none or mild for not being able to finish a normal sized meal for a healthy person
- none or mild for abdominal pain

If the mGCSI-DD is not available at or after discharge in a patient discharged home without tolerating a solid meal, the time to solid food will be censored at 168 hours.

8.6.4 Sensitivity Analysis

The sensitivity analysis is to evaluate the assumptions of using the mGCSI-DD in the primary analysis of patients discharged from the hospital without solid food tolerance. Time to solid food tolerance for patients who could not tolerate solid food during the hospital will be censored to 168 hours if the patient is discharged from the hospital during the study and not tolerating solid food. If the subject was not discharged from the hospital during the study and did not tolerate solid food, the time to solid food tolerance will be censored at the latest date and time of the hospital stay.

8.6.5 Supplementary Analyses

Time to solid food tolerance through the Day 30 visit will be displayed using a Kaplan-Meier estimate and will be compared between the 3 treatment groups and the placebo group using a stratified log-rank test stratified by eCRF recorded sex (male or female) and the risk for organ failure (higher or lower) based on eCRF data.

Supplementary analysis 1: The definition of time to solid food tolerance and the censoring method follow the primary analysis method.

Supplementary analysis 2: The definition of time to solid food tolerance and the censoring method follow the sensitivity analysis method.

8.7 Secondary Endpoint Analyses

8.7.1 Percentage of Patients who Tolerated Solid Food at 48, 72, and 96 Hours from the SFISD and at Discharge

Solid food tolerance will be calculated using the main analytical approach as described in Section 8.6.3.

If the time to first solid food tolerance ≤ 48 hours then solid food tolerance at 48, 72 and 96 hours are all yes.

If the time to first solid food tolerance >48 and ≤72 hours, then solid food tolerance at 72 and 96 hours are all yes and solid food tolerance at 48 hours is no.

If the time to first solid food tolerance >72 and ≤96 hours, then solid food tolerance at 96 hours is yes and solid food tolerance at 48 and 72 hours are all no.

If the time to first solid food tolerance > 96 hours or the patient never tolerates the solid food during the study then solid food tolerance at 48, 72, and 96 hours are all no.

If the time to first solid food tolerance ≤time to hospital discharge, solid food tolerance at discharge is yes, otherwise, solid food tolerance at discharge is no. If a patient was not discharged from the hospital before end of study, solid food tolerance at the end of study is no.

If it is unknown if solid food has been tolerated at a specific time point (time to first solid food tolerance is censored before the specific time point), the solid food tolerance will be imputed as no.

The proportion and 95% CI of patients who tolerated solid food at 48, 72, and 96 hours from the SFISD, at discharge, and at the end of the study will be displayed using a Clopper-Pearson interval and will be compared between each Auxora treatment group and placebo group using a Cochran-Mantel-Haenszel test stratified by the eCRF recorded sex (male or female) and the risk for organ failure (higher or lower) based on eCRF data. Prohibited immunosuppressive medications or immunotherapies and treatment discontinuation will be ignored (treatment policy strategy).

8.7.2 Time to Medically Indicated Discharge

The time to medically indicated discharge is the number of days from the SFISD to the first date of meeting the criteria defining medically indicated discharge:

- the patient tolerates solid food, as defined by the main analytical approach; and
- abdominal pain is controlled or resolved, which will be defined by a reduction in pain, as assessed by the PNRS scale, of ≥50% from the peak level in the first 24 hours and no use of opioids; and
- there is no clinical evidence of an infection requiring continued hospitalization.

In patients who do not meet the criteria for medically indicated discharge when discharged from the hospital, the time to medically indicated discharge will be defined as 8am on the first of three consecutive days where the 4 criteria are met for the mGCSI-DD as defined in 8.6.3. If a patient is discharged not meeting the criteria for medically indicated discharged and does not meet criteria for the mGCSI-DD or has not completed the mGCSI-DD, the time to medically indicated discharge will be censored at 168 hours. If the patient dies while hospitalized, the time to medically indicated discharge will be censored at Day 30.

The time to medically indicated discharge will be summarized using Kaplan-Meier analyses. The medians and quartiles of time-to-events will be summarized for each study group. The time to medically indicated discharge will be compared between each Auxora treatment group and placebo group using stratified log-rank test stratified by sex (male or female) and the risk for organ failure (higher or lower). Prohibited immunosuppressive medications or immunotherapies and treatment discontinuation will be ignored (treatment policy strategy).

The proportion and 95% CI of patients who meet the medical discharge criteria by the 96-hour visit and the date of discharge will be displayed using a Clopper-Pearson interval and will be compared between each Auxora treatment group and placebo group using a Cochran-Mantel-Haenszel test stratified by the eCRF recorded sex (male or female) and the risk for organ failure (higher or lower) based on eCRF data. Prohibited immunosuppressive medications or immunotherapies and treatment discontinuation will be ignored (treatment policy strategy).

The correlation between meeting the medically indicated discharge criteria on the day of discharge with each of the following events, hospital readmission for acute pancreatitis, the development of pancreatic necrosis/necrotizing pancreatitis, and worsening CTSI score at Day 30 from Screening, will be summarized by treatment groups by Matthews correlation coefficient (MCC).

8.7.3 Length of Stay in the Hospital

The number of days patients are in the hospital from the SFISD, including ICU stay and readmissions for any etiology, during the 30 Days of the study will be summarized by treatment groups and compared between each Auxora treatment group and placebo group using an analysis of variance model, which includes treatment group, the stratification factor of the eCRF recorded sex (male or female), and then the stratification factor of the risk for organ failure (higher or lower) based on eCRF data as the fixed effects in the model. The proportion of patients in each quartile of hospital stay (1-7 days, 8-14 days, 15-21 days, and 22-30 days) and the patients who remain hospitalized at Day 30 will also be summarized by treatment groups.

Two sets of analyses will be conducted. The first analysis is to assess patient benefit; the number of days in the hospital will be defined as 30 if the patient dies due to acute pancreatitis (composite strategy). The second analysis is to assess healthcare systems benefit; the number of days in the hospital before the patient's death will be used in the analysis (while alive strategy).

8.7.4 Re-hospitalization for acute pancreatitis (AP) Through Day 30

The date of each re-hospitalization and the reasons for re-hospitalizations will be collected for each patient. The proportion and 95% CI of patients who are re-hospitalized for either acute pancreatitis or the development of pancreatic necrosis/necrotizing pancreatitis through the Day 30 visit will be displayed using a Clopper-Pearson interval and will be compared between each Auxora treatment group and placebo group using a Cochran-Mantel-Haenszel test stratified by the sex (male or female) and the risk for organ failure (higher or lower) based on eCRF data. Prohibited immunosuppressive medications or immunotherapies and treatment discontinuation will be ignored (treatment policy strategy). If the re-hospitalization for acute pancreatitis status is unknown, the re-hospitalization for acute pancreatitis will be imputed as No.

The proportion and 95% CI of patients who are re-hospitalized for other causes through Day 30 will also be analyzed in a similar manner.

8.7.5 Change in Severity of AP by CTSI Score from Screening to Day 30

Independent reviewers will perform a blinded central review of Contrast Enhanced Computed Tomography (CECT) imaging data, or MRI imaging data, obtained at the Screening and Day 30 visits to assess the computed tomography severity index (CTSI). Independent review will be performed by two independent radiologists (primary review) using a consensus methodology.

If the patient undergoes additional CECTs or MRIs at the discretion of the PI or treating physician from randomization to the Day 30 visit, the CTSI data will be listed but not used in the analysis unless the Day 30 visit data are missing. If the Day 30 visit data is missing, the last available unscheduled post-treatment data will be used for the change from baseline at Day 30 analysis based on the LOCF method.

Shift tables will display changes in severity by CTSI score (mild, moderate, severe) from baseline compared to Day 30 by treatment groups.

Proportion of patients with moderate or severe AP by CTSI score at baseline who became mild AP at Day 30, and the proportion of patients with mild AP at baseline who become moderate or severe AP will be displayed and analyzed in the same way as the re-hospitalization for acute pancreatitis through Day 30.

8.7.6 Development of pancreatic necrosis ≥30% and >50%

The development of pancreatic necrosis ≥30% and >50% through Day 30 will be assessed by the independent reviewers in the same way as the CTSI score.

The proportion and 95% CI of patients who developed pancreatic necrosis ≥30% and >50% during the study will be displayed and analyzed in the same way as the re-hospitalization for acute pancreatitis through Day 30. If the Day 30 data is missing, the last available unscheduled post-treatment data will be used as pancreatic necrosis status at Day 30 based on the LOCF method.

The proportion and 95% CI of patients who developed necrotizing pancreatitis, defined as either pancreatic or peripancreatic necrosis, during the study will be displayed and analyzed in the same way as the re-hospitalization for acute pancreatitis through Day 30. If the Day 30 data is missing, the last available unscheduled post-treatment data will be used as pancreatic necrosis status at Day 30 based on the LOCF method.

8.7.7 Persistence of SIRS ≥48 Hours After the SFISD

The persistence of SIRS ≥48 Hours after the SFISD is defined by the presence of at least two of the following four criteria at the first visit 48 hours after the SFISD:

- Temperature < 36°C or > 38°C;
- Heart rate > 90 beats/minute;
- Respiratory rate >20 breaths/minute or arterial carbon dioxide tension ($PaCO_2$) <32 mmHg;

- White blood cell count (WBC) >12,000 mm3, or <4,000 mm3;

The proportion and 95% CI of patients who developed persistence of SIRS ≥48 Hours After the SFISD will be displayed and analyzed in the same way as the re-hospitalization for acute pancreatitis through Day 30. If the patient is discharged before 48 Hours after the SFISD, the last available post-treatment data will be used to define the SIRS status at 48 hours after the SFISD based on the LOCF method.

8.7.8 Incidence, Severity, and Duration of Organ Failure

The incidence, severity and duration of organ failure will be defined by the development of severe respiratory failure or severe renal failure or severe cardiac failure. Severe respiratory failure will be defined as those patients receiving invasive mechanical ventilation (IMV) or those receiving for ≥ 48 hours use of either high flow nasal cannula (HFNC) or non-invasive mechanical ventilation (NIMV). Use of IMV, HFNC, or NIMV for ventilatory support during a procedure, or use of NIMV for the treatment of obstructive sleep apnea, will not be considered as meeting the definition of severe respiratory failure. Severe renal failure will be defined as the initiation of renal replacement therapy. Severe cardiovascular failure will be defined as the use of vasopressor or inotropic support for ≥48 hours. Use of vasopressor or inotropic support during a procedure will not be considered as meeting the definition of severe cardiovascular failure.

The proportion and 95% CI of patients who developed severe respiratory failure or severe renal failure or severe cardiovascular failure will be displayed and analyzed in the same way as the re-hospitalization for acute pancreatitis through Day 30. The proportion of and 95% CI of patients who developed more than one organ failure (multi-organ failure) will be also displayed and analyzed. The mean duration of severe respiratory failure, severe renal failure, and severe cardiac failure will be summarized by treatment groups.

Given the previous results from study CM4620-204, a sensitivity analysis that will combine the subjects from the 1.25 mL/kg and 0.625 mL/kg groups into one combined group and will combine the subjects from the 0.3125 mL/kg and placebo groups into another combined group. The proportion and 95% CI of patients who developed severe respiratory failure, as well as those who developed multiorgan failure, in the two combined groups will be displayed and analyzed in the same way as the re-hospitalization for acute pancreatitis through Day 30.

A gMCP-Mod analysis of severe respiratory failure will be conducted in the same three steps method as Session 8.6.1.

- Step 1: Conventional Step – Odds Ratio Between Each Dose Versus Placebo

The rate of severe respiratory failure will be analyzed using the logistic regression with treatment, sex (male or female) and the risk for organ failure (higher or lower) as covariates to obtain treatment effects and corresponding variance covariance matrix.

- Step 2: Candidate Models and Multiple Testing Procedure

The optimal contrast based on Candidate models for Auxora dose for the logit of rate of severe respiratory failure follows the following table

Model	Contrast coefficients for dose			
	Placebo	0.5 mg/kg	1.0 mg/kg	2.0 mg/kg
Emax	0.866	-0.264	-0.293	-0.308
sigEmax	0.663	0.300	-0.424	-0.539

Note: Candidate model of Emax model is based on the assumption of parameter of ED50=0.026 mg/kg. Candidate model of sigEmax mode is based on the assumption of parameters of ED50=0.606 mg/kg and h=4.3

- Step 3: Dose-finding step – Dose-Response Model Fitting

The procedure is the same as the step 3 of Session 8.6.1.

The proportion and 95% CI of patients who developed new onset persistent respiratory failure or new onset persistent renal failure or new onset persistent cardiovascular failure will be displayed and analyzed in the same way as rehospitalization for acute pancreatitis through Day 30. New onset is defined as organ failure that was not present at randomization. Persistent is defined as ≥48 hours. Respiratory failure, renal failure, and cardiovascular failure are defined by a score of ≥2 on the modified Marshall scoring system. Patients with a known medical history of chronic kidney disease will be excluded from the analysis of new onset renal failure.

8.7.9 Mortality by Day 30

Mortality is recorded at the Day 30 follow-up assessment. The proportion and 95% CI of patients who died by Day 30 will be displayed and analysed in the same way as the re-hospitalization for acute pancreatitis through Day 30. If the Day 30 vital status is unknown and the patient has been discharged, the patient will be treated as alive.

8.7.10 Change in Pain Score

Pain numeric rating scales are recorded through the Day 10 visit and at the Day 30 visit. The mean score at each visit and mean changes from baseline to each post-baseline visit of the pain numeric rating scale will be summarized by the treatment group.

8.8 Exploratory Endpoints

The development of infected pancreatic necrosis, and of sepsis will be summarized by the number of patients and treatment group.

Mean changes in total mGCSI-DD score from screening to discharge and to Day 30, IL-6 levels, and urine neutrophil gelatinase-associated lipocalin (NGAL) levels to each post-baseline visit will be summarized by the treatment groups.

The mean change in IL-6 levels in patients with a baseline level ≥150 pg/mL to the 72-hour visit will be summarized by the treatment group. The change in urine NGAL in patients with a baseline level ≥150 ng/mL to the 72-hour visit will be summarized by the treatment groups.

Shift tables will display the number of patients with baseline IL-6 levels >1000 pg/mL, 150-1000 pg/mL at 72 hours by the treatment groups.

8.9 Proof-of-Concept Composite Endpoint Analysis

Finkelstein-Schoenfeld Analysis

The proof-of-concept efficacy analysis uses a hierarchical combination applying the method of Finkelstein-Schoenfeld (Finkelstein 1999) to the following:

1. All-cause mortality at Day 30 Visit, and
2. Severe respiratory failure through the Day 30 Visit, and
3. Pancreatic necrosis ≥30% through the Day 30 Visit, and
4. Rehospitalization for AP or pancreatic necrosis/necrotizing pancreatitis through the Day 30 Visit, and
5. Time to solid food tolerance within 30 Days of the SFISD

The test score will be computed using a pairwise ranking procedure. The test statistic will be based on the sum of these scores and was stratified by the eCRF recorded sex (male or female) and then the stratification factor of the risk for organ failure (higher or lower) based on eCRF data.

Within each stratum, each subject will be compared to every other subject in a pair-wise manner. The pair-wise comparison assigned a +1 to the "better" subject and a -1 to the "worse" subject. All-cause mortality will be given higher priority in the calculation and only when two given subjects are alive at Day 30 when evaluating on all-cause mortality, then persistent severe respiratory failure, pancreatic necrosis >30%, rehospitalization for AP, and time to solid food tolerance would be used in the comparison in sequence.

- If one subject is alive and the other is not, the live subject receives a +1 and the deceased one a -1.
- If both subjects died, then the comparison is a tie.
- If both subjects are alive, then the comparison evaluates persistent severe respiratory failure to assign scores. The subject with persistent severe respiratory failure receives a -1 while the other without persistent severe respiratory failure receives +1.
- If both patients have, or do not have, persistent severe respiratory failure, then the comparison evaluates pancreatic necrosis ≥30%. The subject with pancreatic necrosis ≥30% receives a -1 while the other without pancreatic necrosis 30% receives +1.

- If both subjects have, or do not have, pancreatic necrosis ≥30%, then the comparison evaluates rehospitalization for acute pancreatitis. The subject with rehospitalization for acute pancreatitis receives a -1 while the other without rehospitalization for acute pancreatitis receives +1.
- If both subjects have, or do not have, rehospitalization for acute pancreatitis, the comparison uses time to solid food tolerance to assign scores. The subject with the longer time to solid food tolerance receives a -1 while the other with shorter time to solid food tolerance receives +1.

No imputation will be done for missing cases for the proof-of-concept composite efficacy analysis based on the Finkelstein-Schoenfeld method. In case when one subject is censored and all-cause mortality, persistent severe respiratory failure, pancreatic necrosis ≥30%, rehospitalization for AP, or time to solid food tolerance are unknown, the unknown status will be used in calculating the score and the score will be considered 0.

The proposed test is a score test based on the sum of the scores for the treated group. The test statistic can be written as

$$T = \sum_{k} \sum_{i \in A_k} D_i U_i$$

A_k is the set of the subjects in the kth strata and U_i is the score calculated within each stratum. D_i=1 for subjects in Auxora group and D_i=0 for subjects in placebo group. K=1,2,3,4 for four strata. The variance is written as

$$V = \sum_{k} \frac{m_k(n_k - m_k)}{n_k(n_k - 1)} (\sum_{i \in A_k} U_i^2)$$

M_k is the total number of Auxora subjects in the kth stratum. N_k is the total number of subjects in the kth stratum.

The hypothesis will be tested by comparing T/ $\sqrt{V}$.

Win Ratio and Confidence Interval

The win ratio method involves a comparison of each patient in an active treatment group with each patient in the placebo group to determine whether the active treatment patient had a better outcome (a "win"), a worse outcome (a "loss"), or an identical outcome (a "tie"). Each comparison begins with the death outcome and proceeds through the hierarchically ordered set of outcomes described below until the first outcome is identified for which the outcomes differ between the patients; this outcome is used to determine the "win" or "loss." The win ratio is the total number of wins divided by the total number of losses, and a win ratio >1 indicates better outcomes with active treatment.

Categories (a), (c), (e), (g), and (i) represent Auxora wins based on all-cause mortality, persistent severe respiratory failure, pancreatic necrosis >30%, rehospitalization for AP, and time to solid

food tolerance sequentially. Similarly, categories (b), (d), (f), (h), and (j) represent placebo wins. Category (k) represents ties, pairs where subjects were not able to be differentiated.

(a) Death on placebo, but alive on Auxora at Day 30
(b) Death on Auxora, but alive on placebo at Day 30
(k) Death on placebo, death on Auxora at Day 30. No further comparisons will be performed.

If alive on placebo and alive on Auxora at Day 30:
(c) Severe respiratory failure on placebo but not on Auxora
(d) Persistent severe respiratory failure on Auxora but not on placebo

If a tie for severe respiratory failure:
(e) Pancreatic necrosis ≥30% on placebo but not on Auxora
(f) Pancreatic necrosis ≥30% on Auxora but not on placebo

If a tie for pancreatic necrosis ≥30%:
(g) Rehospitalization for AP on placebo but not on Auxora
(h) Rehospitalization for AP on Auxora but not on placebo

If a tie for rehospitalization for AP:
(i) Earlier time to solid food tolerance on Auxora
(j) Earlier time to solid food tolerance on placebo

If a tie for rehospitalization for AP
(k) Tie for all outcomes

The overall win ratio will be calculated by adding (a) + (c) + (e) + (g) + (i) for all 4 strata and dividing it by the sum of (b) + (d) + (f) + (h) + (j) across all 4 strata.

The standard error of log (Win Ratio) can be calculated as:
SE (Log Win Ratio) = log (Win Ratio) / Z score from FS method
The confidence interval can be computed as follows:
95% CI for Win Ratio = exp (log (Win Ratio) +/- 1.96 * SE (Log Win Ratio))

The Win Ratio with 95% CI and p-value based on Finkelstein-Schoenfeld method will be used to compare each Auxora group versus placebo group.

The proof-of-concept analysis will perform a sensitivity analysis that will combine the subjects from the 1.25 mL/kg and 0.625 mL/kg groups into one combined group and combine the subjects from the 0.3125 mL/kg and placebo groups into a second combined group. The two combined groups will be compared using the Finkelstein-Schoenfeld method and Win Ratio method (Finkelstein 1999). This grouping method assumes that the 1.25 mL/kg and 0.625 mL/kg groups have the similar treatment effect on persistent severe respiratory failure. If this assumption is not valid after the primary analysis of the time to solid food tolerance after the database lock, the grouping method may be adjusted.

8.10 Safety Endpoint Analyses

The safety evaluation will be purely descriptive using descriptive statistics (N, mean, SD, median minimum and maximum) or frequency tables where appropriate using the MITT analysis set, unless otherwise specified. No imputation will be made in case of missing values.

8.10.1 Adverse Events

Adverse events (AEs) will be coded using MedDRA dictionary. If an incomplete or missing onset date was collected for an AE, the imputation method of missing data specified in Section 8.1.5 will be applied. Treatment-emergent AEs (TEAEs) are defined as AEs that started or worsened in severity on or after the SFISD. The relationship to study treatment for each AE will be classified as ‘Unrelated’, ‘Unlikely Related’, ‘Possibly Related’, ‘Probable Related’, ‘Definite Related’. AEs classified as ‘Possibly Related’, ‘Probable Related’, or ‘Definite Related’’ will be analyzed as ‘Related’ in the AE summaries. Data listings, patient narratives, etc. will present the relationship to study treatment as collected on the eCRF.

8.10.1.1 Tabulations of TEAEs

The frequency and incidence of TEAEs will be summarized by System Organ Class (SOC) and Preferred Term (PT) by treatment and then by overall treatment. The actual version of the MedDRA coding dictionary used will be noted in the clinical study report. The SOC is sorted by alphabetic order; then within SOC, PT is sorted by descending counts under Auxora Total column, then descending counts under placebo column, then alphabetic order for PTs with the same count.
Each of the summaries will be done at the subject level. Multiple occurrences of the same event within a subject will be counted once in the summaries by SOC and PT.

All AEs for each subject, including multiple occurrences of the same event, will be presented in full in a comprehensive listing including subject number, treatment, severity, seriousness, action taken, outcome, relationship to treatment, onset/stop date, and duration. The data listings for serious TEAEs and TEAEs leading to discontinuation of study treatment will be generated as well.

8.10.1.2 Adverse Event Overview

An overview of AEs will be presented by the actual treatment received overall and consisting of the number and percentage of patients experiencing at least one event for the following AE categories:

- Any TEAEs
- TEAEs related to study treatment
- Serious TEAEs
- Serious TEAEs related to study treatment
- TEAEs leading to discontinuation of study treatment
- TEAEs leading to death

8.10.1.3 Adverse Event by SOC and PT

The following summaries of AEs will be generated:

- Incidence of TEAEs
- Incidence of TEAEs related to study treatment
- Incidence of serious TEAEs
- Incidence of serious TEAEs related to study treatment
- Incidence of TEAEs leading to discontinuation of study treatment
- Incidence of TEAEs leading to death
- Incidence of severe TEAEs

8.10.2 Vital Signs

Vital signs will include temperature, systolic and diastolic blood pressure, respiratory rate (breaths per minute), heart rate (beats per minute) and QTc (milliseconds). Vital signs will be examined at each scheduled visit and time point. Clinically significant, treatment-emergent findings will be reported as AEs. In addition, vital signs results will be flagged as potentially clinically significant (PCS) if they meet the criteria which are defined below:

- Pulse rate >130 bpm
- Pulse rate <45 bpm
- Diastolic blood pressure >100 mmHg
- Systolic blood pressure >155 mmHg
- Systolic blood pressure <80 mmHg
- Respiration rate >25 breaths/min
- Body temperature ≥39 °C
- QTc >500 milliseconds

The number and percent of patients meeting each PCS criterion at each scheduled visit and time point will be summarized by the actual treatment and then by the overall treatment.

8.10.3 Laboratory Analyses

Local laboratory test results will be used in the summary. CBC with differential, platelets, serum chemistries, and serum procalcitonin will be summarized.

Laboratory values by visit will be summarized as boxplots. The lower and upper reference range values for lab tests will be used to grade the lab results to low, normal, and high categories. Shift tables will display:

- Shift from baseline category to the worst category
- Shift from baseline category to the last category

8.10.4 Arterial Blood Gas

If an arterial blood gas (ABG) has been performed as supportive care, the time of the draw and following results will be listed: the pH, PaO_2, $PaCO_2$, SaO_2 and FiO_2.

8.10.5 SpO_2/FiO_2

SpO_2 and FiO_2 parameters, and imputed P/F ratios will be listed.

8.11 PK Assessments

PK analysis will be performed as a separate analysis.

8.12 Subgroup Analyses

Subgroup analyses will be performed to explore how time to solid food tolerance through Day 30 visit and proof-of-concept endpoint are influenced by baseline variables and to evaluate the treatment effect at different levels of each of these variables. The Kaplan-Meier analysis, Cox model, Finkelstein-Schoenfeld Method, and Win Ratio will be performed by subgroup levels of the baseline variables listed below:

- Age (<50 vs ≥50)
- Race (White vs Asian vs Other)
- Sex (Male vs Female)
- Etiology (Alcohol vs Other)
- SIRS (Two criteria vs > Two criteria)
- Risk For Organ Failure (High vs Low as determined by values entered in eCRF)
- Hypoxemia (imputed P/F ≤360 vs >360)
- HCT (High: HCT ≥44% for men or ≥40% for women vs Low: HCT <44% for men or <40% for women)
- Balthazar Score on Screening CECT by central reading (A or B or C, vs D or E)
- WBC (>12,000 mm^3 or < 4,000 mm^3 vs ≤12,000 mm^3 and ≥4,000 mm^3)
- Patients enrolled in India vs. in the United States of America

To increase the number of subjects in each comparison group of the subgroup analysis, the subgroup analysis will combine the subjects from the 1.25 mL/kg and 0.625 mL/kg groups into one pooled Auxora group. This pooled Auxora group will be compared with the placebo group. This grouping method assumes that the 1.25 mL/kg and 0.625 mL/kg groups have the similar treatment effect. If this assumption is not valid after the primary analysis of the time to solid food tolerance, the grouping method will be adjusted.

9. INTERIM ANALYSIS

9.1 Interim Safety Analyses

Scheduled periodic review meetings will be conducted by an independent data monitoring committee (IDMC) overseeing the development program of Auxora. An IDMC charter will govern the IDMC and will describe the scope of responsibilities of the IDMC. If the IDMC

recommends alteration of the dosing regimen because of safety issues, the FDA and other regulatory agencies will be notified as appropriate. The IDMC responsibilities include protecting the safety of the study patients and making recommendations to CalciMedica concerning the conduct of the study.

10. VALIDATION

The tables, figures, and listings (TFLs) planned in this SAP will be programmed using SAS software version 9.4 (or above). The TFLs will be quality checked by the statistics team at using SAS software version 9.4 (or above).

11. PROGRAMMING AND DATA PRESENTATION CONVENTIONS

Listings will be presented in treatment, subject, visit (where applicable) and date (where applicable) order. Listings will be produced (landscape in MS Word) using PROC REPORT.

Summary tabulations will be presented by treatment group (and overall if appropriate), scheduled visit order (if appropriate). Continuous data summaries will present (unless stated otherwise) number of observations, mean, standard deviation, median, minimum, and maximum. Categorical data summaries will present the number of observations and the corresponding percentage.

12. SUMMARY OF CHANGES TO STATISTICAL ANALYSIS PLAN VERSION 1.0

Table 3. Summary of changes from statistical analysis plan version 1.0

#	**Nature of change from Statistical Analysis Plan 1.0**	**Rationale**
1	N/A	

13. SUMMARY OF CHANGES TO PROTOCOL PLANNED ANALYSIS

Table 4. Summary of changes from protocol planned analysis

#	**Nature of change from Protocol Planned Analysis**	**Rationale**
1	The analysis of the secondary endpoints of length of stay in the ICU for patients admitted to the ICU.	Length of stay in the ICU will be integrated into length of hospital stay.

14. BIBLIOGRAPHY

1. Bretz F, Pinheiro JC, Branson M. Combining multiple comparisons and modeling techniques in dose-response studies. Biometrics 2005; 61:738–748.

2. Pinheiro J, Bornkamp B, Glimm E, Bretz F. Model-based dose finding under model uncertainty using general parametric models. Statistics in Medicine 2014; 33:1646-1661.

3. Finkelstein DM, Schoenfeld DA. Combining mortality and longitudinal measures in clinical trials. Stat Med. 1999 Jun 15;18(11):1341-54. doi: 10.1002/(sici)1097-0258(19990615)18:11<1341::aid-sim129>3.0.co;2-7. PMID: 10399200.
